# Whole genome protein microarrays for serum profiling of immunodominant antigens of *Bacillus anthracis*

**DOI:** 10.3389/fmicb.2015.00747

**Published:** 2015-08-13

**Authors:** Karen E. Kempsell, Stephen P. Kidd, Kuiama Lewandowski, Michael J. Elmore, Sue Charlton, Annemarie Yeates, Hannah Cuthbertson, Bassam Hallis, Daniel M. Altmann, Mitch Rogers, Pierre Wattiau, Rebecca J. Ingram, Tim Brooks, Richard Vipond

**Affiliations:** ^1^Public Health EnglandPorton Down, Salisbury, UK; ^2^Department of Medicine, University College London, Hammersmith HospitalLondon, UK; ^3^Department of Bacterial Diseases, CODA-CERVA (Veterinary and Agrochemical Research Centre)Brussels, Belgium; ^4^Centre for Infection and Immunity, School of Medicine, Dentistry and Biomedical Sciences, Queen's University BelfastBelfast, UK

**Keywords:** anthrax, microarray, immunology, protein, antibody, infection

## Abstract

A commercial *Bacillus anthracis* (Anthrax) whole genome protein microarray has been used to identify immunogenic Anthrax proteins (IAP) using sera from groups of donors with (a) confirmed *B. anthracis* naturally acquired cutaneous infection, (b) confirmed *B. anthracis* intravenous drug use-acquired infection, (c) occupational exposure in a wool-sorters factory, (d) humans and rabbits vaccinated with the UK Anthrax protein vaccine and compared to naïve unexposed controls. Anti-IAP responses were observed for both IgG and IgA in the challenged groups; however the anti-IAP IgG response was more evident in the vaccinated group and the anti-IAP IgA response more evident in the *B. anthracis*-infected groups. Infected individuals appeared somewhat suppressed for their general IgG response, compared with other challenged groups. Immunogenic protein antigens were identified in all groups, some of which were shared between groups whilst others were specific for individual groups. The toxin proteins were immunodominant in all vaccinated, infected or other challenged groups. However, a number of other chromosomally-located and plasmid encoded open reading frame proteins were also recognized by infected or exposed groups in comparison to controls. Some of these antigens e.g., BA4182 are not recognized by vaccinated individuals, suggesting that there are proteins more specifically expressed by live Anthrax spores *in vivo* that are not currently found in the UK licensed Anthrax Vaccine (AVP). These may perhaps be preferentially expressed during infection and represent expression of alternative pathways in the *B. anthracis* “infectome.” These may make highly attractive candidates for diagnostic and vaccine biomarker development as they may be more specifically associated with the infectious phase of the pathogen. A number of *B. anthracis* small hypothetical protein targets have been synthesized, tested in mouse immunogenicity studies and validated in parallel using human sera from the same study.

## Introduction

*Bacillus anthracis* is a large Gram-positive spore-forming, rod-shaped bacterium that is the etiological agent of the zoonotic disease Anthrax. Reservoirs for Anthrax are wild and domestic ruminants, most commonly sheep, goats, and cattle and their proximity to humans within an agricultural setting can cause infection and disease (Dixon et al., 1999). Disease may be contracted through either direct contact with infected animals, or within industrialized countries, through contact with animal by-products such as wool, skins, (Wattiau et al., 2009) or bone meal, where spores can survive in the environment for decades (Jernigan et al., 2001; Olano and Walker, 2011). This occurs when spores enter the body through breaks in the skin, via ingestion or by inhalation (Little and Ivins, 1999). Cutaneous anthrax is clearly identifiable by the eschar lesion which appears sometime after infection at the site of entry, which is usually self-limiting and treatable with antibiotic therapy (Nalin, 1999). However, intestinal and inhalational anthrax with severe atypical pneumonia are by far the most serious forms and can be rapidly fatal. After a critical turning point, these forms of the disease no longer respond to treatment and the patient succumbs to overwhelming septicaemia and toxic shock (Baillie and Read, 2001). This is due to release of various toxins by the Anthrax bacillus formed of edema factor (EF) or lethal factor (LF) and protective antigen (PA) (Liu et al., 2014), the genes for which are carried on a plasmid pXO1. LF and PA combine to form lethal toxin and EF and PA form edema toxin (Hanna, 1998; Little and Ivins, 1999), which are responsible for the systemic toxinogenic effects leading to cell death. Full virulence of *B. anthracis* requires additional proteins for capsule biosynthesis encoded on a second plasmid pXO2 (Mikesell et al., 1983; Turnbull, 1991). The toxin proteins are in general *B. anthracis* specific, whereas homologs of the capsule genes have been found reasonably commonly in other closely related species. The toxin proteins have as a consequence been under development by a number of commercial groups as sub-unit vaccine candidates for Anthrax infection (Brey, 2005; Splino et al., 2005; Comer and Peterson, 2009; Friedlander and Little, 2009; Altmann, 2015).

Although Anthrax is primarily a disease of the African and Asian sub-continents (Dixon et al., 1999) and infection is rarely encountered in the UK, Western Europe and the USA. It has made something of a re-emergence in recent years under unusual circumstances. In 2001 there was an outbreak in postal workers and other individuals in the USA exposed to letters contaminated with Anthrax spores (Jernigan et al., 2001, 2002; Dewan et al., 2002; Perkins et al., 2002). This outbreak lead to infection of 22 individuals with five deaths, whole-scale disruption of the US postal system for a number of months and was traced to an incident of deliberate release (Greene et al., 2002; Hadler, 2007). In addition, there have been a number of more recent infections in the UK and US associated with contaminated goat skins used in the manufacture and playing of drums (Anaraki et al., 2008; Mayo et al., 2010) and the intravenous use of contaminated heroin (Booth et al., 2010; Hicks et al., 2012; Price et al., 2012; Grunow et al., 2013; Meghji et al., 2013; Berger et al., 2014; Veitch et al., 2014). This recent outbreak has to date resulted in 54 infected individuals and 17 deaths. The breakdown of cases shows 48 confirmed cases and 13 deaths in Scotland (Ramsay et al., 2010; Palmateer et al., 2012; Booth et al., 2014), five confirmed cases and four deaths in England (Booth et al., 2010) and other cases in Europe (Holzmann et al., 2012; Grunow et al., 2013). Soft tissue infections caused by spore-forming bacteria in intravenous drug users are not uncommon (Dancer et al., 2002; Mcguigan et al., 2002; Murray-Lillibridge et al., 2006; Lavender and Mccarron, 2013; Ascough and Altmann, 2015), but Anthrax is rare. This cluster of cases is unusual as only one previous case has been documented in Norway in 2002 (Russell et al., 2013). This highlights the need for on-going vigilance for detection of the disease in the community and also for maintenance and development of methods for screening, surveillance and protection (Joseph and Read, 2010; Rao et al., 2010).

Testing for rare pathogens is conducted by specialist reference laboratories within the UK and tests include a combination of classical methods e.g., culture, molecular and serological assays^1^. Continued development of specific, rapid microbiological tests are a research feature of most specialist reference laboratories and on-going work ensures constant improvement of “in house” services. Responsibility for Anthrax testing and environmental surveillance within the UK lies with Public Health England (PHE) Porton and this establishment has a continuing interest in Anthrax biology research. PHE Porton also manufactures the UK licensed Anthrax vaccine (AVP; Anthrax Vaccine Precipitated) and undertakes Anthrax vaccine research and development. The diagnostic technologies group at PHE Porton supports the activities of both these diagnostic and vaccine development areas. We have been investigating a number of methods for detection and characterization of *B. anthracis* and for identification of candidate markers for further diagnostic and vaccine development.

To this end we have been researching the use of protein microarrays as a platform for immune-competent antigen discovery, using immune and control sera. This method has been used successfully previously by other groups (Felgner et al., 2009; Cretich et al., 2010; Schweitzer et al., 2010; Cruz-Fisher et al., 2011) and for Anthrax vaccine immune sera using colony blot (Kudva et al., 2005) and immune-proteomics (Liu et al., 2013). We have used a commercially sourced *B. anthracis* whole genome protein microarray [Protoarray™, Thermofisher (formerly Invitrogen)] for the screening of immune sera from a number of different human cohorts and samples from a rabbit model of Anthrax vaccination. These have been exposed to *B. anthracis* antigens, either via vaccination with the current UK Anthrax protein vaccine, natural or acquired infection. The human subjects are volunteer donors who have received the UK licensed anthrax vaccine, plus unvaccinated controls, occupationally exposed workers from a Belgian wool-sorters factory (suspected pulmonary exposure), naturally infected Turkish individuals (confirmed cutaneous anthrax) and individuals in a cohort of intravenous drug users (IVDU), tested either positive or negative for anthrax infection.

Our aim is to identify immunodominant protein biomarkers which can be used as candidates for diagnostic or vaccine development and development of reagents for further characterization of the UK AVP Anthrax protein vaccine. A comparison was made between infected, vaccinated and control groups to determine if there are immunodominant antigens expressed during natural infection that are not present in the current UK vaccine and/or specific for different groups. Due to the differing routes of infection that these samples represent it is likely that different antigen complements will be expressed or that arms of the immune system will recognize different anthrax antigen profiles as has been observed for different complex Anthrax vaccines (Brenneman et al., 2011). Identification of antigens unique to individuals exposed to Anthrax spores would help facilitate development of new or more rapid tests, particularly the development of antibody-based antigen capture technologies (Olano and Walker, 2011) or vaccine development (Soborg et al., 2009; Ascough et al., 2014). These new rapid methods may become useful in a clinical setting (Weile and Knabbe, 2009) where early diagnosis is crucial. Antigens shared between the vaccinated and exposed groups could be useful in characterization of the current vaccine and also for further vaccine candidate selection. The next generation of Anthrax vaccines will be based on a thorough knowledge of the interaction between the virulence factors of *B. anthracis* and the host immune response (Turnbull, 1991; Brey, 2005).

Here we present results of this pilot Anthrax immunodominant biomarker identification study. We have identified a number of statistically significant proteins which are either specific to individual groups i.e., naturally exposed, infected individuals or vaccinees, or are shared between the groups. Group-specific and shared biomarker profiles are outlined and these have revealed interesting information about the number and the type of antigens recognized within and between test groups. These data suggest that a number of protein antigens are expressed only during infection, as these are not recognized by vaccinated or control individuals. This also suggests there are a group of proteins expressed by *B. antracis* which are not currently present in the licensed UK AV vaccine, which would be of value in future diagnostic test and vaccine development. Detailed characterization of a few select antigens is also presented and provides an interesting insight into the transcriptome of virulent *B. anthracis* in a host infection environment.

## Materials and methods

### Immune and control sera

#### Anthrax infected, exposed, vaccinee, and control human sera

Vaccinee and control sera were provided by Dr. Sue Charlton of the Vaccine Research Group (PHE) and had previously been assayed for the presence of anti-Protective Antigen (PA), anti-Lethal Factor (LF), and anti-Edema Factor (EF) antibodies, using Enzyme Linked Immunosorbant Assays (ELISA). There were six naïve sera and six vaccinated sera of varying titers for anti-toxin antibodies (2 low, 2 medium, and 2 high), the samples were anonymized before receipt. Sera were sourced from known cutaneous Anthrax-infected individuals from Turkey and occupationally exposed individuals from a Belgian wool-working factory (Wattiau et al., 2009). Sera were also sourced from individuals suspected of Anthrax infection from the recent outbreak in intravenous drug users. These were confirmed Anthrax positive (AP IVDU) or confirmed negative (AN IVDU) cases, by either anti-PA and LF ELISA, *B. anthracis*-specific PCR or bacterial culture.

#### Rabbit live spore and AVP vaccine sera

Duplicate New Zealand White Rabbits were injected intramuscularly and sub-cutaneously on Day 0 with live *B. anthracis* Sterne strain spores (5 × 10^6^ Colony forming units (CFU)) or AVP vaccine, with a booster on Day 9. Control rabbits were vaccinated with saline solution only. A test bleed was taken from each animal 7 days (Day 17) after each exposure to the requisite vaccine/saline control. Rabbits were then given another boost on Days 21 and 30 with a second test bleed collected 7 days after the fourth immunization. This was repeated a further 5 times. Hyperimmune sera were prepared from each animal from a final bleed taken on Day 137 and used in hybridization experiments to *B. anthracis* ProtoArrays™.

### Assay methodology

#### Anthrax toxin human and rabbit serum ELISAs

The wells of a microtiter plate were coated with 100 μl of rPA or rLF (10 μg/ml) diluted in coating buffer (71627 Sigma) covered with a plate sealer and left for 60 ± 30 min at 37 ± 3°C in a shaking incubator. The coating buffer was removed and the plates washed with fresh wash buffer [1 × phosphate buffered saline (PBS, P5368 Sigma) 0.1% Tween 20 (P5927 Sigma)]. One hundred micro liters of blocking buffer [wash buffer + 5% fetal calf serum (FCS, B8655 Sigma)] were added to each well, the microtiter plate covered with a plate sealer and incubated for a further 60 min at 37°C in a shaking incubator. This was removed, washed three times with wash buffer for 1 min and then 100 μl of each serum sample added to the microtiter plate. The plate was covered with a plate sealer and incubated for 60 min at 37°C in a shaking incubator. Anti-IgG or IgA horseradish peroxidase conjugated antibodies were diluted 1 in 100 (Human) or 1 in 1000 (Rabbit) and equilibrated at room temperature prior to use. The sample was removed and the plate washed again three times with wash buffer. 100 μl of diluted HRP conjugated anti-Human IgG or IgA (anti-human IgG # VX628420, anti- human IgA # VX627421, both Invitrogen Life Technologies) or anti-rabbit (HRP conjugated anti-rabbit IgG # ab6759 or anti-rabbit IgA # ab8510, both Abcam, UK) was added to each well, the plate was covered with a plate sealer and incubated for 60 min at 37°C in a shaking incubator, then the conjugate was removed and the plate washed three times with wash buffer. Twelve milliliters of TMB substrate (T0440 Sigma) was incubated at room temperature for 60 min then 100 μl added to each well. The plate was covered with a plate sealer and this was left to incubate without shaking at room temperature for 5 min. One hundred microliters of TMB stop solution (S5814 Sigma) were added to each well and the plate read using a VersaMax™ ELISA Microplate reader at 450 nm. Data were quantified and exported for analysis using the Softmax Pro version 5.2 ELISA software.

#### Serum hybridization to anthrax whole genome Protoarrays

All parts of the protocol were conducted at 4°C. Invitrogen Anthrax ProtoArray slides [Protoarray™, Thermofisher^2^ (formerly Invitrogen)] were stored at −20°C, therefore prior to use arrays were allowed to equilibrate at room temperature to avoid excess condensation forming. ProtoArrays were therefore equilibrated at 3–5°C, then rehydrated with 1 × blocking buffer (50 mM Hepes pH 7.5, 200 mM NaCl, 0.08% Triton X-100, 25% Glycerol, 20 mM reduced Glutathione, 1.0 mM DTT, 1% Fetal Calf Serum (FCS), equilibrated to pH 7.5-8.0 with 10 mM NaOH), ensuring coverage of the array, in a sealed slide holder under constant agitation for 1 h. The slides were then aspirated and carefully washed three times with 1x PBST buffer [1x PBS (P5368 Sigma) and 0.1% Tween 20 (P5927 Sigma)] containing 1% FCS. The buffer was gently aspirated off and 15 mls of diluted serum added (1:500 dilution in PBST/0.1% Tween 20) and incubated at 4°C for 90 min under constant gentle agitation. The slides were then washed three times with 1x PBST buffer, then hybridized with two fluorescently labeled secondary antibodies at a 1/1000 dilution for 45 min, goat anti-human IgG-Cy3 (# ab6597 Abcam, UK) and goat anti-human IgA-Cy5 (# 109-175-011-JIR Jackson ImmunoResearch, West Grove, USA) or goat anti-rabbit IgG-Cy3 (# ab6939, Abcam, UK) and goat anti-rabbit IgA alpha chain-DyLight® 650 (# ab96978, Abcam, UK). The slides were washed three times with 1x PBST buffer and air dried under centrifugation for 5 min at 3000 rpm (Sorvall Legend RT). They were then scanned with a GenePix Pro 4200A microarray scanner (Molecular Devices, Germany). The fluorescent signals of each channel (Cy3 and Cy5) were quantified and then exported for further analysis using Bluefuse™ microarray quantification software (BlueGnome, UK).

### Data analysis

#### Statistical processing of ELISA data

Raw ELISA data were imported into Systat Software (Inc.) SigmaPlot for Windows version 12.0. Data points were plotted in graphical format with log_10_ transformed serum dilution values on the x-axis and untransformed extinction coefficients on the Y axis. These were then analyzed using three parameter logistic regression analyses according to the given formula below. The intersect corresponding to the midpoint of the slope of curve on the X axis i.e., X_0_ was taken as representation of the half maximal effective serum dilution value (ED_50_).

$$y=\frac{a}{1+{\left(\frac{x}{x_{0}}\right)}^{b}}$$

#### “R” analysis of distribution of anthrax Protoarray hybridization data

All *B. anthracis* Protoarray serum hybridization output data were imported into the statistical package “R”^3^ and visualized in histogram graphical format (using the hist base function: signal intensity on the x-axis vs. frequency on the y-axis). Raw data were then log_2_ transformed (using the log2 function) and re-plotted to investigate the distribution of the data. Data were then sorted into groups according to origin i.e., negative and positive controls and anthrax-specific features and overlaid on a plot of all transformed data points.

#### Analysis of anthrax Protoarray hybridization data using genespring GX 12.5

All *B. anthracis* Protoarray serum hybridization output data were then imported into the bioinformatics software Agilent GeneSpring 12.5 (GX 12.5) and downstream analytical processes conducted using the statistical analysis tools and other visualization features of this package. Imported raw data were normalized to the mean of the buffer only control features, then baseline transformed to the median of all samples. Normalized data were assigned to their respective groups, filtered on expression to remove any features with negative intensity values, then either analyzed using fold-change analysis or Kruskal Wallis One-Way analysis of variance [ANOVA (asymptotic *p* ≤ 0.05, no multiple testing correction) see below], using as baseline the negative control condition.

$$K=\left(N-1\right)\frac{\sum_{i=1}^{g}n_{i}{\left({\bar{r}}_{i\cdot }-\bar{r}\right)}^{2}}{{\sum_{i=1}^{g}\sum_{j=1}^{n_{i}}\left(r_{ij}-\bar{r}\right)}^{2}}$$

Individual group comparisons were analyzed using the non-parametric Mann–Whitney *U*-test (asymptotic *p* ≤ 0.05, fold change cut off ≥ 1.5, unpaired, no multiple testing correction)

$$U_{1}=n_{1}n_{2}+\frac{n_{1}\left(n_{1}+1\right)}{2}-R_{1}$$

All other analyses including unsupervised hierarchical cluster analysis and depictions of raw and processed data using other visual outputs e.g., Venn diagram comparisons, were conducted using other GX 12.5 functions using default settings.

### Identification and chemical synthesis of anthrax peptide antigens

Anthrax peptides identified from BLAST searches of online databases (see **Table 3**), were synthesized chemically at > 95% purity by Peptide Synthetics, Peptide Protein Research Ltd., UK^4^. These were re-suspended in sterile, purified water (or 10–20% DMSO/sH_2_O) at a concentration of 2 mg/ml prior to use. These were used to coat ELISA plates at a concentration of 2 μg/ml in coating buffer. Anti-peptide Ig ELISAs were conducted using the procedures outlined above for the anti-toxin ELISAs but substituting Pierce protein-free blocking buffer (Life technologies #37584).

## Results

### Determination of anti- anthrax toxin antibody responses and study group designation

All human sera were assayed for Anti-PA and LF toxin component IgG and where possible IgA reactivity by direct (immobilized antigen) ELISA using well-established protocols. These were used to confirm prior anti-toxin ELISA screening of cutaneous Anthrax patients, exposed woolworkers and vaccine sera using routine diagnostic methods and establish anti-toxin titers for IgA (See Supplementary Information S1). Anthrax positive IVDU (AP IVDU) and Anthrax negative IVDU (AN IVDU) sera were not retested due to insufficient sera remaining from routine clinical testing. Data given in Supplementary Information S1 for these latter patients is derived from routine diagnostic ELISA data. No data are given for anti-toxin IgA titers for these latter groups.

All human control sera were assigned to their respective test groups according to geographical recruitment/vaccination or exposure history and positive anti-toxin titers for the AVP vaccines (AVPV), Turkish cutaneous Anthrax patient (TCA) and Belgian wool-sorters (BWS) group. AP IVDU and AN IVDU were assigned to their respective groups based on prior anti-PA and anti-LF IgG results and/or Anthrax bacillus culture or PCR positivity/negativity (Ramsay et al., 2010). All rabbit sera were assayed for anti-PA and LF IgG titers using established methods, similar to those conducted for human sera, substituting anti-rabbit IgG HRP-conjugated secondary detection antibody. These were assigned to their respective groups on vaccination schedule history.

### Identification of immunogenic anthrax proteins using invitrogen anthrax whole genome expressed Protoarrays

All immune and control sera were dual hybridized to Anthrax whole genome ProtoArrays at a standard serum dilution of 1:500 using either Cy3-labeled anti-human or rabbit IgG and Cy5-labeled anti human or DyLight® 650-labeled anti rabbit IgA. The slides were scanned in both channels, the data quantified and exported for further analysis using the microarray software Bluefuse™. Cy3 IgG and Cy5/DyLight® 650 IgA data were exported and analyzed separately.

#### Evaluation, normalization, and analysis of Protoarray Ig-hybridization data using non-parametric statistical analyses

Anthrax whole expressed proteome (ProtoArray) slides were manufactured by Invitrogen and in addition to the 4916 *B. anthracis*—specific protein features, spotted in duplicate, the array contains a number of negative and control features. These comprise a series of negative controls (blank, buffer, empty, *n* = 6700 features) and positive controls mainly chemical or human biological in origin (Alexafluor markers, AntiBiotin, human IgG, Biotin, BSA, Calmodulin, CMK, GST, anti-Human IgG, Internal, Kinase, MAPKAP, V5Control). These, plus other additional features, i.e., two-fold dilution series of the Anthrax toxins PA, LF and EF, comprise the remaining array spots (*n* = 2668). Thus in total there are 19200 total features, arranged in 12 rows and 4 columns in a 20 × 20 sub-grid configuration.

**Figure 1:**
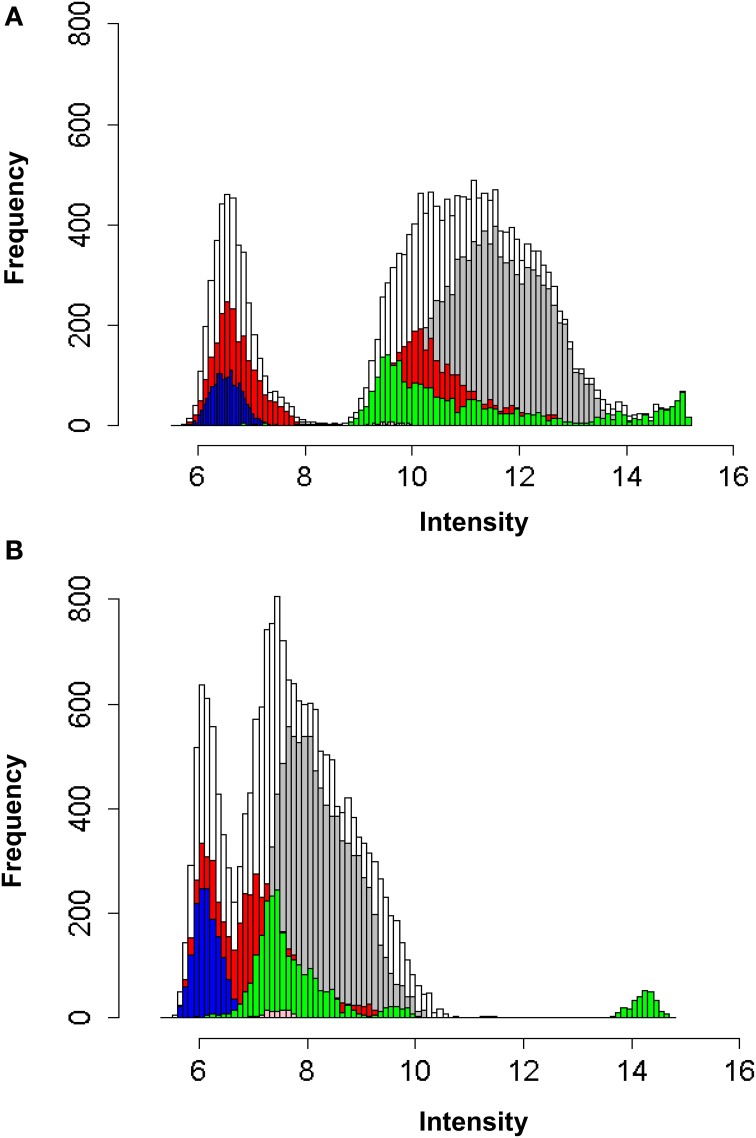
**(A)** Log_2_ frequency of distribution of fluorescence intensities of all protein, positive and negative control array entities using Cy3 labelled anti-human IgG antibody. **(B)** Log_2_ frequency of distribution of fluorescence intensities of all protein, positive and negative control array entities using Cy5 labelled anti-human IgA antibody. 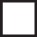 All combined data 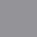 *B. anthracis* protein entities 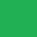 *H. sapiens* control protein entities (Alexa, AntiBiotin, Anti-human IgG, Biotin, BSA, Calmodulin, CMK, GST, Human IgG, Internal, Kinase, MAPKAP, V5Control) 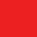 features labelled as ‘empty’ on the array 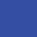 features labelled as ‘blank’ on the array 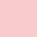 features labelled as ‘buffer’ on the array.

In order to investigate the frequency and intensity of distribution of the data and to inform selection of optimum normalization methods, all combined data were plotted in histogram format (Supplementary Information S2) and Figures 1A,B). Raw data for both IgG and IgA human serum hybridizations were found to not exhibit a normal distribution (Supplementary Informations S2 A,B). These were then Log_2_ transformed and found to exhibit a triphasic distribution (Supplementary Informations S2 C,D). Color coding of the different feature groups on the array (Supplementary Informations S2 E–J) revealed non-normal distribution in all groups, but particularly the negative and positive control groups. The reasons for these are not known, however some may be due to technical anomalies (i.e., high fluorescence intensity of the “empty” and “blank” controls), or due to specific (anti human IgG) or non-specific (MAPKAP) Ig binding to these features. However, these are not useful in analytical interrogation of the array and may have the effect of skewing standard default normalization and baseline transformation processes (e.g., to default 75th percentile and median of all samples). They also dominate downstream statistical analyses based on *p*-value ranked datasets. Although the *B. anthracis*-specific features also show a similar distribution pattern, this was less pronounced. It was therefore decided to filter the data to remove all extraneous controls with the exception of the “buffer” only and continue analysis using these and the *B. anthracis*-specific features (including toxins) only. Non-parametric statistical tools were used for all downstream analytical processes.

Sorted raw data (replicate data-points) for *B. anthracis*-specific features and buffer only, from both IgG and IgA ProtoArray hybridizations using sera from all human groups were imported into GX12.5, normalized to the average of buffer only control features, then baseline transformed to the median of all samples as described above. All entities were then filtered on expression and then analyzed using either the non-parametric Kruskal-Wallis One-Way ANOVA (no multiple testing correction and an asymptotic corrected *p*-value cut-off of *p* ≤ 0.05) or Mann–Whitney *U*-test analyses (unpaired, no multiple testing correction, at a fold-change cut off ≥1.5 and *p* ≤ 0.05). Data were then analyzed further and depicted graphically using other functions in GX 12.5.

#### Data statistical analysis of normalized human Protoarray Ig-hybridization data using non-parametric ANOVA

**Figure 2:**
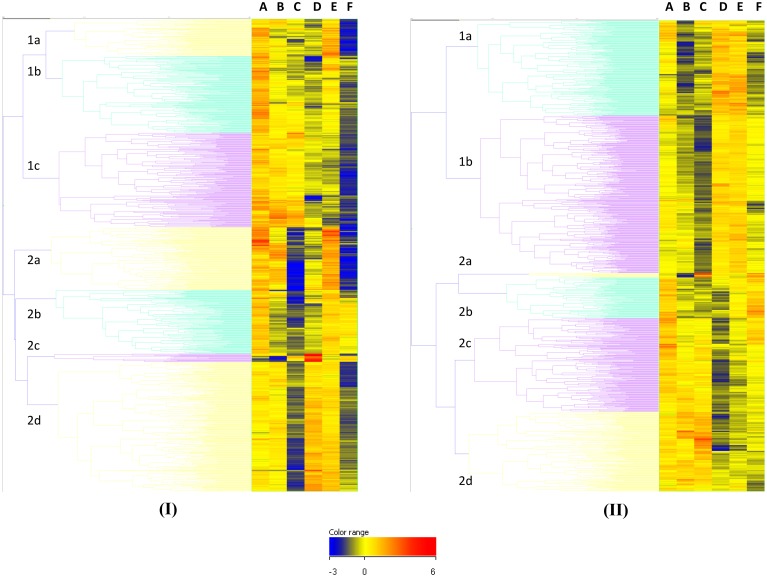
**Unsupervised Euclidean hierarchical cluster analysis of shared T1080 hits between IgG positive and IgA positive ANOVA entities**. **(A)**, IgG data set **(B)**, IgA data set.

All human IgG and IgA hybridization data were analyzed using Kruskal–Wallis One-Way ANOVA as described above, 6556 of 9706 (67.6%) features were positive for recognition by IgG and 1789 (18.4%) for IgA. The data were ranked on p value; the top ranked differentially recognized hit for IgG was EF (0.78125 ng/μl concentration, with the 0.360295 ng/μl concentration at position 5). These plus other EF features are strongly recognized by the AVPV group and not by the AP IVDU or the TCA groups. Other toxin features also figured strongly in the top 100 entities at various dilutions (data not shown), along with other *B. anthracis* proteins. The top ranked differentially recognized hit for IgA was not EF which appeared at position 15 (0.78125 ng/μl concentration). This feature again appeared to be more specifically recognized by the AVPV and also the BWS groups and not the AP IVDU, AN IVDU or the TCA groups. Other toxin dilution features did not feature strongly in the top ranked 100 for IgA. This may be due to the fact that these are recognized less strongly overall by IgA compared with IgG. However, there is clear differential recognition of the toxin components in the different groups and in particular the AVPV and AP IVDU and TCA groups for both IgG and IgA as seen in Figure 2. Cluster 2c in Figure 2I and cluster 2a in Figure 2II contain the majority of the toxin features and exhibit clear differential recognition between groups (discussed in further detail in Section Microarray Investigation of Human IgG and IgA Anti- Anthrax Toxin Features and Identification of *B. anthracis*-specific Hypothetical Proteins and Peptide ELISA Analysis of Human Sera below).

Of these statistically significant entity lists, 1080 (T1080) were shared between the IgG and IgA datasets [see Supplementary Information S3). Unsupervised Euclidean hierarchical cluster analyses for the T1080 feature list for IgG and IgA data are shown in Figure 2. Overall there is high IgG recognition of many protein entities by the naïve control group; most likely due to recognition of common shared eubacterial proteins from prior exposure. However unusually, recognition of the total number of proteins by control sera do appear to be in excess of those seen for all other groups. There appears to be particularly reduced recognition of protein features by the infected AP IVDU and TCA groups, which is most pronounced for IgG and less so for IgA. This is particularly evident in the TCA group which shows very restricted protein recognition repertoire for IgG. This is also evident in cluster analysis of the complete unfiltered IgG ANOVA dataset (data not shown). However, in the IgA dataset there is good recognition of proteins by all groups, yet the repertoire of proteins recognized is different. The recognition profiles of the AP IVDU, AN IVDU, and TCA are highly similar, whereas the AVPV and BWS profiles are alike. Using Hierarchical cluster analysis on both conditions and entities on the IgA ANOVA data, the AP IVDU, AN IVDU, TCA, and control groups co-cluster together, with the AP IVDU, AN IVDU in one sub-cluster and the TCA and control group in a second sub-cluster, whereas the AVPV and BWS associate separately (data not shown). This indicates similar recognition profiles between the AP IVDU, AN IVDU, and the TCA and control group. The profiles of proteins are clearly different between the clusters. Similar analyses using the IgG data (data not shown) revealed that the AP IVDU and TCA groups co-cluster together, with the remaining groups clustering separately. Thus highly similar recognition profiles for IgG are seen for the AP IVDU and the TCA groups with very restricted repertoires, the TCA group overall exhibiting the most restricted IgG protein repertoire recognition. IgG recognition of proteins in the infected groups appears depressed, whereas IgA recognition of proteins is less depressed but exhibits group-specific restricted repertoires, with some groups showing similarities.

#### Comparative non-parametric T-test statistical analysis of normalized human Protoarray Ig-hybridization data and identification of group specific antigens

To further delineate some of the differentially recognized features (DRF) between the control and test groups, non-parametric Mann–Whitney *U*-test analyses were conducted between the control and each of the individual test groups (Supplementary Information S4) for both IgG and IgA hybridization data.

These revealed for the IgG data (a) 8 entities DRF for the BWS, including the 3 lowest PA and 2 lowest LF concentration features, plus BA1930, BA0973 and BA2389 and 297 DRF for the control [Supplementary Information S4 Figure A (i)] (b) 4 entities DRF for the AN IVDU, including BA2377 (duplicate features), BA2389 and BA5591 and 1255 DRF for the control [Supplementary Information S4 Figure A (ii)] (c) 3 entities DRF for the AP IVDU including PA 200 ng/μl concentration and BA4182 (duplicate features) and 1990 DRF for the control [Supplementary Information S4 Figure A (iii)] (d) 27 entities DRF for the AVPV including 27/30 of the PA, LF, and EF concentrations (excluding the EF 200, PA 0.78125, and 0.390625 ng/μl concentrations) and 1582 DRF for the control [Supplementary Information S4 Figure A (iv)] (e) 13 entities DRF for the TCA including 9/10 of the LF concentrations and the top four concentrations of PA (200, 100, 50, and 25 ng/μl) and 6773 DRF for the control [Supplementary Information S4 Figure A (v)]. Supplementary Information S4 Figure A (vi) shows a comparison between the Anthrax positive groups of all statistically significant entities which bar 4 are all toxin component features. Remaining group-specific entities are given in Supplementary Information S4 Table 1.

Similar analyses for the IgA data revealed (a) 123 entities DRF for the BWS, with no positive toxin features and 737 DRF for the control [Supplementary Information S4 Figure B (i)] (b) 8 entities DRF for the AN IVDU and 321 DRF for the control [Supplementary Information S4 Figure B (ii)] (c) 18 entities for the AP IVDU, again including BA4182 [see Supplementary Information S4 Figure B (vii) and Supplementary Information S5] and PA (50 ng/μl concentration) and 437 DRF for the control [Supplementary Information S4 Figure B (iii)] (d) 80 entities DRF for the AVPV including EF (0.78125 ng/μl) and LF (0.390625 ng/μl) and 2378 DRF for the control [Supplementary Information S4 Figure B (iv)] (e) 3 entities for the TCA including BA2967 and BA3953 and 275 DRF for the control [Supplementary Information S4 Figure B (v)]. Supplementary Information S4 Figure B (vi) shows a comparison between the Anthrax positive groups of all statistically significant entities. Group-specific entities are given in Supplementary Information S4 Table 2.

**Figure 3:**
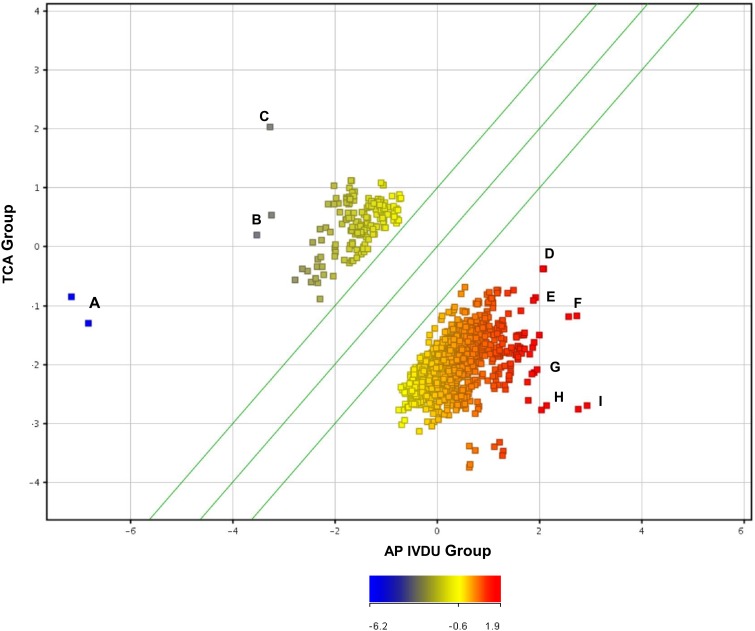
**Scatter plot depiction of statistically significant IgG recognized entities using a Mann–Whitney ***U***-test (unpaired, unequal variance, ***p*** < 0.05, fold change cut-off 1.5) between the IVDU Anthrax positive and the Turkish cutaneous Anthrax patient groups**. Highlighted immunogenic, differentially recognized protein entities A to I are given in detail in Supplementary Information S6 Table 2.

**Figure 4:**
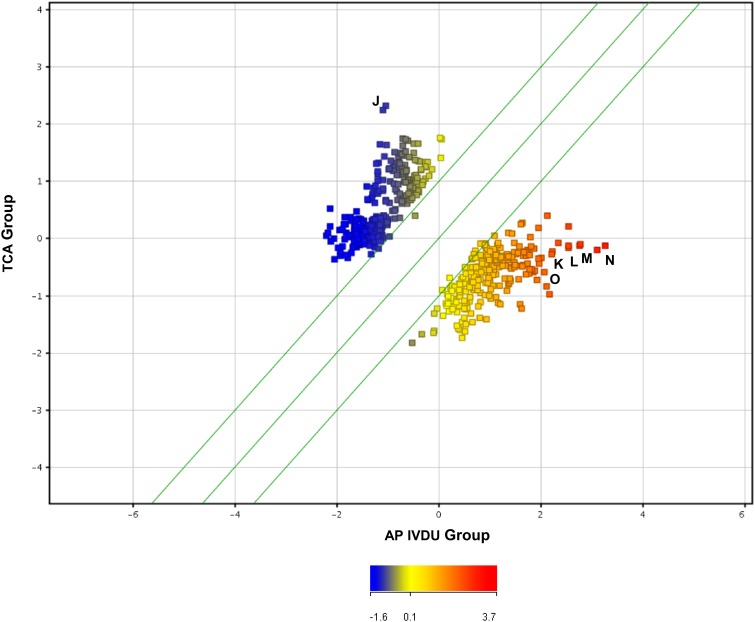
**Scatter plot depiction of statistically significant IgA recognized entities using a Mann–Witney ***U***-test (unpaired, unequal variance, ***p*** < 0.05, fold change cut-off 1.5) between the IVDU Anthrax positive and the Turkish cutaneous Anthrax patient groups**. Highlighted immunogenic, differentially recognized protein entities J–Q are given in detail in Supplementary Information S6 Table 2.

Similar analyses were conducted between the clinical infected TCA and AP IVDU groups to identify antigens which may be differentially recognized (*U*-test data outputs depicted in scatter plot graphical output in Figures 3, 4). One hundred and fourteen DRF for the TCA and 650 DRF for the AP IVDU were found between the groups for the IgG data and 268 DRF for the TCA and 244 DRF for the AP IVDU were found between the groups for the IgA data. However, many of these entities are also recognized by other groups. All entity lists were further interrogated on an individual entity basis for group-specific DRF which are outlined in detail in Supplementary Information S6 Tables 1, 2.

**Table 1:** *****B. anthracis***-specific and other partially conserved immunogenic protein entities identified by sequence comparison of all ***B. anthracis*** hypothetic proteins with database sequences using BLAST**.

**Gene name**	**Name**	**Peptide sequence/alignment to nearest neighbor**	**% Similarity**	**GroupIg positivity**	***p*-Value human IgG**	***p*-Value human IgA**
BA2157	Hypothetical protein (*B. anthracis*)	MSKNETVLLNQIEIVIEIFKNIQKVDPFYKDL MSKNETVLLNQIEIVIEILKNIQKEDPFYKDL	94	NA	NA	NA
BA2182	Hypothetical protein (*B. anthracis*)	MDYVENNRIHFQHTKRKLESFQLPFFFTEQYLI	0	NA	NA	NA
BA1695	Hypothetical protein (*B. anthracis*)	MLYEETIYHFDCI SFLSNECLGRIYRIEKNDEQAI MLYEETIYHFDRKSFLPHERLGRICEIEKNDEQAI	80	Turkish (IgA)	ND	1.16 × 10^−1^
BA0448	hypothetical protein (*B. anthracis*)	MKRIGINDKCIGCGAEVDDPECECEWRTCSCCGYPDCF VYEEGRYYHCKNCDHSTDPGHY	0	Belgian and Turkish (IgG) Turkish (IgA)	9.1 × 10^−3^	1.64 × 10^−2^
BA4898	Small, acid-soluble spore protein (*sspB*)	MARSTNKLAVPGAESALDQMKYEIAQEFGVQLGADAT MSRSTNKLAVPGAESALDQMKYEIAQEFGVQLGADAT ARANGSVGGEITKRLVSLAEQQLGGFQK ARANGSVGGEITKRLVSLAEQQLGGFQK	98	Turkish (IgA)	ND	4.02 × 10^−2^

**Table 2:** **IgG Anti-PA, LF and immunogenic peptide (from Table 1) ELISA data**.

**Antigen**	**C**			**AVPV**			**TCA**			**AP IVDU**		
	**EC_50_**	**Standard error**	***R***	**EC_50_**	**Standard error**	***R***	**EC_50_**	**Standard error**	***R***	**EC_50_**	**Standard error**	***R***
PA	NA	NA	NA	300.233	145.975	0.9311	NA	NA	NA	157.512	112.115	0.758
LF	NA	NA	NA	89.822	90.534	0.908	NA	NA	NA	69.577	62.239	0.915
BA2157	NA	NA	NA	NA	NA	NA	NA	NA	NA	NA	NA	NA
BA2182	NA	NA	NA	NA	NA	NA	NA	NA	NA	NA	NA	NA
BA1695	NA	NA	NA	NA	NA	NA	NA	NA	NA	NA	NA	NA
BA0448	NA	NA	NA	NA	NA	NA	NA	NA	NA	25.221	77.188	0.867
BA4898	NA	NA	NA	NA	NA	NA	NA	NA	NA	NA	NA	NA

*EC50 (χ_0_), standard error and R-values are given from three parameter logistic regression analysis*.

#### Microarray investigation of human IgG and igA anti- anthrax toxin features

**Figure 5:**
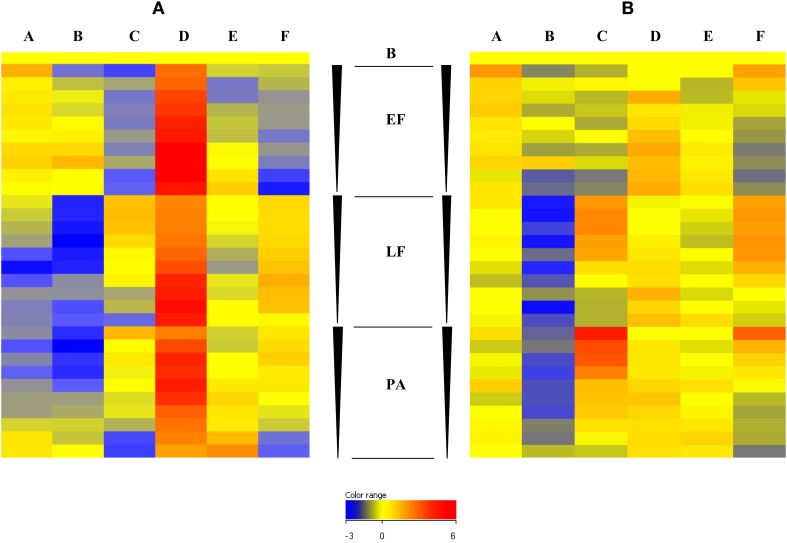
**Fluorescence intensity of ***B. anthracis*** anti-toxin IgG (I) and IgA (II) responses for edema factor (EF), Lethal factor (LF) and Protective antigen (PA) and buffer only control (B)**. A concentration gradient from 200 to 3.125 ng/μl was spotted on the array for each toxin protein, indicated by the block arrowheads, from highest (top) to lowest (bottom) concentration. **(A)** Negative controls **(B)** IVDU Anthrax negative **(C)** IVDU Anthrax positive **(D)** AVP vaccinees **(E)** Belgian Woolsorters **(F)** Turkish cutaneous Anthrax patients.

The Anthrax ProtoArray contained spotted features of two-fold dilutions of each of the toxin components PA, LF, and EF (concentration ranges from 200 to 0.390625 ng/μl i.e., 10 features per toxin subunit). These features were extracted and investigated separately (Figure 5) for both IgG and IgA to compare recognition of the toxins between different groups. This revealed for IgG (1) strong recognition of all dilutions of all three toxin components by the AVPV group, in a gradient with preferential recognition of the lowest toxin concentrations (2) reduced recognition of PA and LF by the AP IVDU and TCA groups (but very low recognition of EF), in a gradient with preferential recognition of the highest toxin concentrations (3) weaker recognition of all toxins for the BWS group, with a graded preference toward lower concentrations for EF and PA and a two-phase gradient of recognition for LF (4) some evidence of recognition of EF and PA by the control and AN IVDU groups and LF by the control group, but this is low and may represent background level recognition.

In addition for IgA (1) weak recognition of all dilutions of all three toxin components by the AVPV group, in a gradient with preferential recognition of the lowest toxin concentrations (2) strong recognition of PA and LF by the AP IVDU and TCA groups (with very low recognition of EF by the AP IVDU group, but recognition of EF at the higher concentrations for the TCA group), in a gradient with preferential recognition of the highest toxin concentrations (3) very weak recognition of all toxins for the BWS group, with a graded preference toward lower concentrations for EF, PA, and LF. There was some evidence of recognition of EF by the control and AN IVDU groups and some background recognition of PA and LF by the control group.

Thus, the infected and vaccinated groups demonstrate good recognition of two or more of the toxin components, with IgG recognition bias for AVP vaccinated individuals and IgA recognition bias for Anthrax infected individuals. The BWS group exhibited unbiased IgG and IgA recognition of toxin components.

### Comparative investigation of microarray human and rabbit igg and iga anti- anthrax toxin features

New Zealand white rabbits were vaccinated with *B. anthracis* Sterne live spore vaccine (LSV) or AVP protein vaccine according to the above protocol (see Section Rabbit Live Spore and AVP Vaccine Sera). All spore and AVP vaccinated rabbits gave good anti-PA and LF IgG titers (data not shown). Final bleed sera were hybridized to the Anthrax ProtoArray according to the same protocol for all human sera (see Section Serum Hybridization to Anthrax Whole Genome Protoarrays) and data processed and imported into GX 12.5 according to the protocols outlined above. Due to the small sample size full statistical processing was not conducted, however fold change analysis (FC cut-off >25) on normalized, baseline transformed IgG and IgA data revealed all toxin components to rank as the top hits (data not shown). A number of other Anthrax proteins were also detected above this threshold, however none were found to be shared with the human datasets. Fold-change values of the toxin components and relative comparison of the fold-change difference between spore and vaccinated rabbit data are given in Supplementary Information S7.

**Figure 6:**
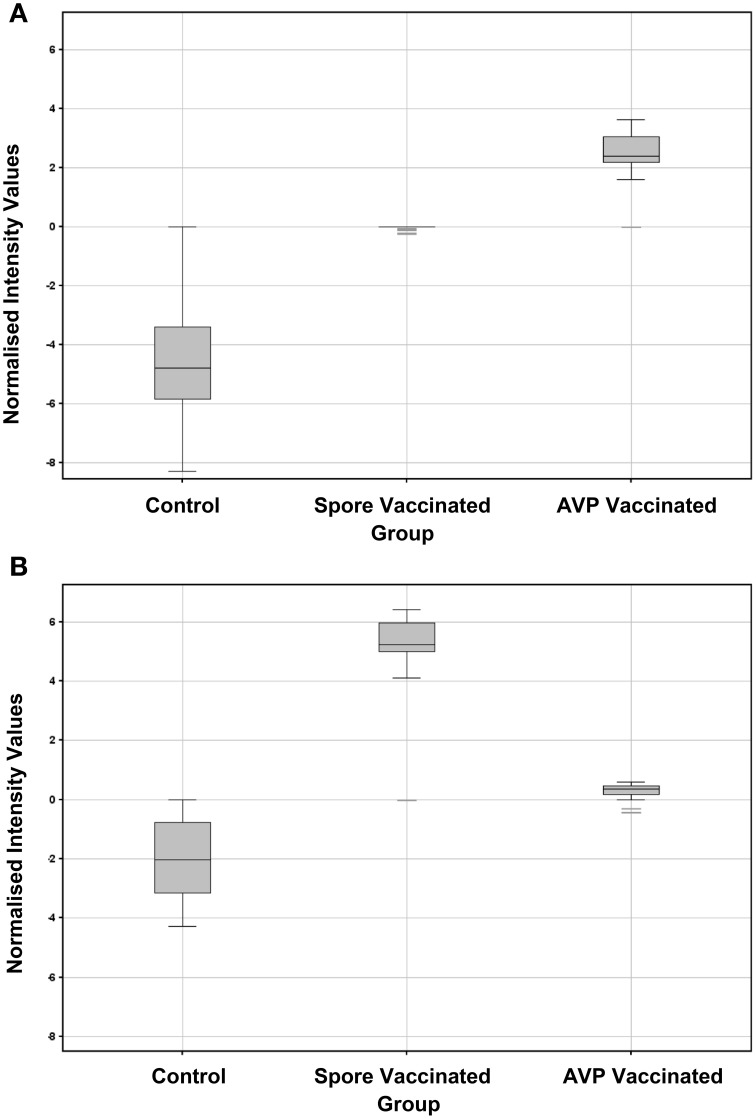
**Box plot graphs of combined array anti-toxin responses across all toxin concentrations for the rabbit spore and AVP vaccinated and control groups (A) IgG (B) IgA**.

**Figure 7:**
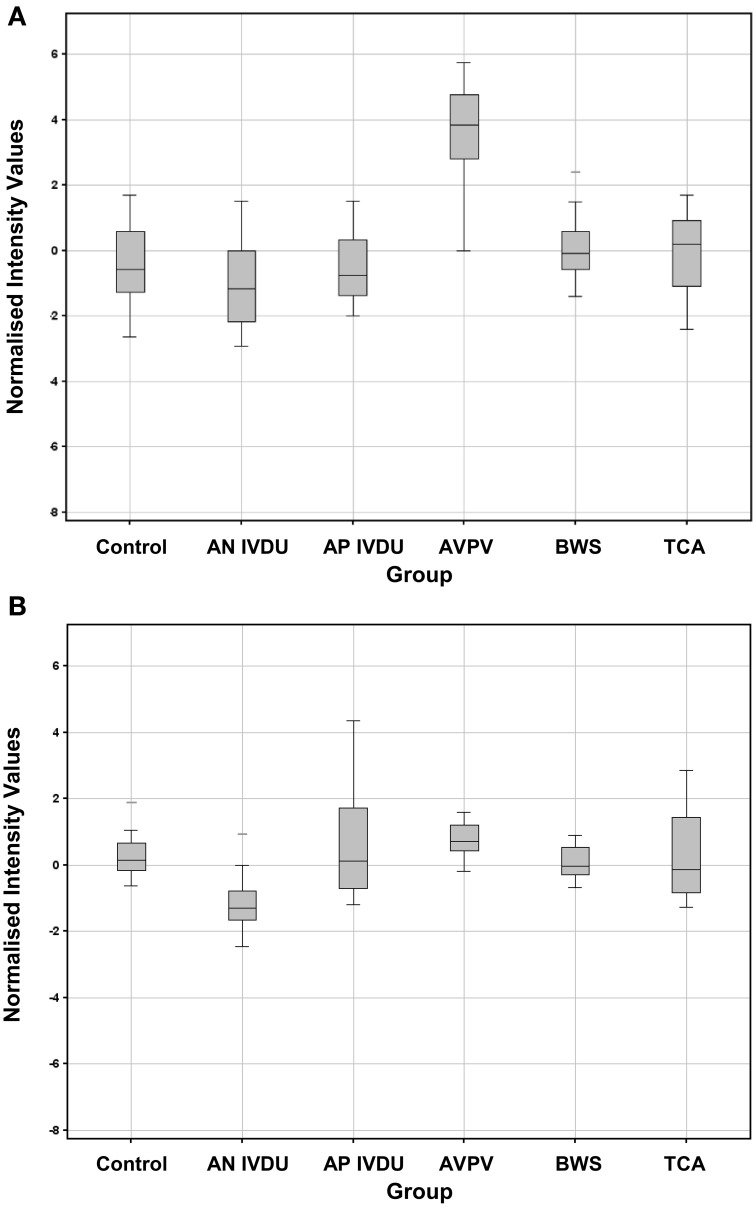
**Box plot graphs of combined array anti-toxin responses across all toxin concentrations for each of the human test and control groups (A) IgG (B) IgA**.

Combined boxplot depictions for normalized toxin data outputs (for all toxin components and concentrations) are given in Figure 6. For comparison analogous human data outputs are given in Figure 7. Good reactivity to all toxin component dilutions was seen in each of the rabbit vaccination challenge groups for both IgG and IgA, when compared to the unvaccinated control. It is apparent that AVP vaccinated rabbit's exhibit a higher anti-toxin IgG response than for LSV vaccinated rabbits, which in turn is above that of the control saline vaccinated rabbits. Conversely, LSV vaccinated rabbits exhibit a higher IgA anti-toxin response than AVP vaccinated rabbits which in turn is above that of the control saline vaccinated rabbits. This observation is paralleled in the human sera data, where the AVPV group exhibits a higher anti-toxin IgG response than the naturally infected AP IVDU and TCA groups, which conversely exhibit a higher anti-toxin IgA response than the AVPV group. The lower threshold for the AP IVDU and TCA group IgA data appears somewhat reduced compared with that for the AVPV IgG response, due to a lack of anti-EF response in these groups, whereas recognition of all toxin component dilutions by IgA is reduced overall in the AVPV group. Thus, live spores through natural infection appear to elicit an IgA biased response, in contrast to that generated through AVP protein vaccination, which exhibits IgG bias. The BWS group exhibit similarities to the AVPV group, perhaps suggesting primary exposure to protein antigen not live spores.

### Identification of *B. anthracis*-specific hypothetical proteins and peptide elisa analysis of human sera

Many of the proteins identified in this study although highly immunogenic and/or exhibiting some specificity for individual groups, were found to either not exhibit *B. anthracis* specificity using BLAST interrogation of online bioinformatics databases or were too large for on-going analysis, without complex cloning, expression and purification. We wished to identify Anthrax specific peptides which exhibited *B. anthracis* species-specificity and were also of a size amenable for chemical synthesis for downstream testing for either vaccination or diagnostic purposes. To this end all annotated *B. anthracis* “hypothetical” and “conserved hypothetical” proteins identified from online databases were compared to all deposited protein and nucleic acid sequences using the BLASTP and tBLASTn^5^ functions of online database search algorithms (data not shown). Five proteins were selected from these analyses, two of which exhibit complete sequence specificity for *B. anthracis*, BA2182 and BA0448, two which exhibit partial sequence specificity BA2157 and BA1695 and one control protein exhibiting extensive sequence conservation with closely related bacillus species BA4898 (Table 3). Only BA0448, BA1695 and BA4898 are represented on the Anthrax ProtoArray. All these peptides were chemically synthesized and then used to screen control and immune sera from the AVPV, AP IVDU, and TCA groups, using established ELISA protocols in comparison to PA and LF as positive controls.

**Table 3:** **IgA Anti-PA, LF, and immunogenic peptide (from Table 1) ELISA data**.

**Antigen**	**C**			**AVPV**			**TCA**			**AP IVDU**		
	**EC_50_**	**Standard error**	***R***	**EC_50_**	**Standard error**	***R***	**EC_50_**	**Standard error**	***R***	**EC_50_**	**Standard error**	***R***
**PA**	2.257	24.704	0.827	17.425	49.059	0.867	22.196	53.151	0.837	338.341	450.497	0.652
**LF**	NA	NA	NA	15.581	36.649	0.911	0.859	9.311	0.868	49.165	72.961	0.889
**BA2157**	NA	NA	NA	14.40	75.286	0.772	121.926	152.2116	0.749	109.596	55.105	0.968
**BA2182**	NA	NA	NA	NA	NA	NA	60.135	105.842	0.769	43.687	61.736	0.967
**BA1695**	NA	NA	NA	24.163	103.353	0.778	182.563	275.875	0.629	3.96	14.627	0.955
**BA0448**	NA	NA	NA	18.274	58.081	0.846	100.593	109.026	0.795	86.747	48.249	0.967
**BA4898**	NA	NA	NA	8.1610	49.5106	0.8073	34.8602	65.8659	0.8348	6.6059	27.3280	0.9064

*EC50 (χ_0_), standard error and R-values are given from three parameter logistic regression analysis*.

It can be seen that none of the five peptides were recognized by IgG in the AVPV and TCA groups and only BA0448 was recognized by the AP IVDU group. LF and PA were recognized at high titer in the AVPV and AP IVDU groups, however there was some response to LF and PA in the TCA group, but no three parameter logistic regression curve could be fitted to this data. *R*-values indicate greater consistency of recognition of LF in the AP IVDU group and that BA0448 exhibits better efficiency of detection than PA, whereas equivalent consistency of recognition of PA and LF is seen in the AVPV group. All 5 peptides were recognized by IgA in the AP IVDU and TCA groups and 4 of 5 were recognized by the AVPV group, with the exception of BA2182. *R*-values indicate again greater consistency of recognition of LF in the AP IVDU group and that all peptides exhibit better efficiency of detection than either LF or PA, whereas recognition of PA and the peptides appear less consistent than LF in the AVPV group. Consistent recognition of either toxin or peptides was not observed in the TCA group, indicating some variability between individual recognition profiles for these antigens. Thus the peptides may offer improved diagnostic detection capability in the AP IVDU group, particularly BA0448.

These peptides were also tested in mouse vaccination studies, however none proved immunogenic in an adjuvant-augmented prime, dual boost vaccination strategy (data not shown), indicating that individually these peptides are not inherently immunogenic as synthetic peptides i.e., in the absence of either other proteins in a complex protein vaccine (i.e., AVP), or in an infection setting.

## Discussion

Sera sourced from a number of suspected and/or confirmed Anthrax infected, UK AVP vaccinated or negative control groups were assessed for their anti-PA and anti-LF Ig responses by ELISA, for IgG and where possible IgA, to confirm or exclude prior Anthrax antigen exposure. Similar confirmatory analyses were conducted for sera from a rabbit vaccination model using the veterinary *B. anthracis* Sterne strain live spore or UK AVP vaccines. All immune and control sera were then hybridized to the Invitrogen Anthrax ProtoArray.

Combined data outputs from these hybridization experiments were evaluated by plotting untransformed and transformed distribution curves and found to exhibit a non-normal (triphasic) distribution. These distribution curves were found in the main to be influenced by high background values from “empty” and “blank” negative controls and the diverse range of “positive control” features, unrelated to the *B. anthracis* protein feature content of the array. High intensity values from these controls had the effect of biasing on-going data analyses both with normal data normalization procedures and when interrogating ranked data sets for statistically significant features. This necessitated the removal of the *B. anthracis* protein features and “buffer” only controls, which were then analyzed separately. Filtered data sets were then imported into the bioinformatics software GX 12.5 using normal procedures and henceforth analyzed using non-parametric data analysis tools.

Kruskal Wallis non-parametric ANOVA analyses revealed a large number of proteins recognized by IgG (67.6%) across all the human groups and fewer for IgA (18.4%). This is confirmed from the distribution curve data (Figure 1), where we see that there is a higher frequency of low intensity IgA- recognized protein features than those observed for the IgG-data. Thus, overall more proteins are recognized by IgG than IgA across all the human groups. However, this seems to be found mainly in the control group, which appear to exhibit recognition of a very large number of protein features compared with the other immune groups. *U*-test analyses between the control and all other groups confirmed this observation. This is somewhat unexpected and while it would be perhaps expected that unvaccinated/challenged individuals would exhibit recognition of a proportion of the proteins represented on the array due to immune memory to cross-reactive, conserved bacterial proteins, the large number of these recognition events is surprising. However, similar results have been observed for a previous study on *Bulkholderia pseudomallei* infection (Felgner et al., 2009), with normal uninfected control sera exhibiting cross-reactive recognition of bacterial species-specific proteins. These were previously suggested to be due to prior exposure to the organism; however it may be in the main due to recognition of conserved bacterial protein epitopes from other unrelated bacterial infections. This requires further investigation.

Another striking observation was the apparent reduced protein repertoire recognition by IgG of many proteins on the array for the AP IVDU and TCA infected groups, particularly for the latter. However, there is some recognition of the toxin and other protein features by IgG, along with a limited number of other proteins. This is an interesting observation and is supported by previous studies which suggest that *B. anthracis* spores, capsule and toxin target immune cells to dismantle the immune system (Baldari et al., 2006; Oliva et al., 2008; Hu and Leppla, 2009; Jelacic et al., 2014). In particular LF toxin and *B. anthracis* spores may specifically target B-cells (Fang et al., 2006; Lightfoot et al., 2014; Sahay et al., 2014). B-cells may also be implicated as the primary cell type involved in dissemination of the bacillus from the lung during inhalational Anthrax (Rayamajhi et al., 2012). Quinn and co-workers showed that a significant proportion of cutaneous Anthrax patients with presumed long-term chronic infection showed no memory B-cell IgG response to PA, whereas acutely infected inhalational Anthrax patients retained B-cell anti-PA IgG immune memory (Quinn et al., 2004). This may imply that the memory B-cell response is abrogated by Anthrax infection, particularly during chronic infection and this is supported by the results presented in this study.

This effect was less pronounced for IgA in both the AP IVDU or TCA groups and this does not appear to be due to intravenous drug use in the former group, as this effect is not seen in the AN IVDU group. The anti-toxin response is more pronounced for IgA in these infected groups. These perhaps constitute a *de novo* response to the infectious agent, after prior infection and subsequent to an apparent near eradication of the memory IgG response (although there is a moderate anti-toxin IgG response in these groups also). Despite evidence of this anti-Anthrax humoral response, this is not protective and appears insufficient to remediate on-going clinical infection, as these patients were confirmed to be infected on sample collection (by culture) and many exhibited severe disease in the AP IVDU group. Many of these IVDU succumbed to the disease and those in the TCA group required antimicrobial therapy.

A number of proteins were recognized by both IgG and IgA (T1080) and cluster analyses revealed group-specific recognition of proteins by both IgG and IgA; however recognition of some proteins was shared between groups. This also showed differential recognition of the toxin components between the groups. Cluster analyses revealed similarities between the recognition profiles of key groups i.e., the AP IVDA and TCA groups co-clustered together on the basis of IgG recognition profiles, whereas the remaining groups clustered together. The view for IgA was somewhat different. In both analyses the AP IVDU and TCA groups always segregate together as do the AVPV and BWS groups, indicating broadly similar recognition profiles. This may be taken to imply a common route of exposure. The AVPV and BWS groups being highly similar have perhaps received exposure through cutaneous contact to protein, whereas in the AP IDVU and TCA groups, exposure is to Anthrax bacilli. This appears to drive very different immune recognition responses, which is dominated by the IgG response in AVP vaccines and by IgA in individuals exposed to live spores.

Mann–Whitney *U*-test analyses of the control versus all other groups revealed a large number of proteins recognized by the control group in comparison to the test groups. Again, as discussed above the recognition of such a large number of proteins by this group is unexplained and further work is required to investigate the origin of this phenomenon. However, there was consistency between the control and all other groups for both IgG and IgA protein recognition profiles. Differentially recognized proteins in the test groups compared to the control comprised mainly different dilution features of the toxin components and a limited number of other protein features. Some of these are group specific. Many of these identified additional proteins were not found to be *B. anthracis*-specific with homologs in other closely related bacterial species. However, some are of interest biologically and warrant further investigation with regard to their specific role in infection and/or their use in on-going vaccine or diagnostic test development. One protein in particular BA1482 (See Supplementary Information S5) or *pdhC* (pyruvate dehydrogenase complex E2 component, dihydrolipoyllysine-residue acetyltransferase) is of particular note and is strongly and specifically recognized by IgG in the AP IVDU group and by IgA in the AP IVDU and TCA groups. This protein may be highly expressed during the infectious process and recognized *de novo* by the humoral antibody immune response. Components of the pyruvate dehydrogenase complex have been shown to be involved in pathogenicity in other bacterial species such as *Mycobacterium tuberculosis*, particularly in latency and persistence (Bryk et al., 2008). The E1 component subunit α of the pyruvate dehydrogenase (PDH) complex (BA4184), has also been identified as a significant, immunogenic *B. anthracis* protein previously by other workers (Liu et al., 2013). This would suggest that similarly *pdhC* may be involved in pathogenicity during Anthrax infection.

There is also clear differential recognition of protein features between the two “infected” groups, perhaps indicating dissimilar disease presentation and/or progression pathways and thus differential protein/pathway expression on the part of the infectious agent in response to differing environmental stress/stimuli. These may perhaps constitute proteins from differentially regulated pathogenicity “regulons.” Like other bacteria *B. anthracis* is found to differentially regulate its transcriptional and hence, by inference, its protein repertoire in response to external environmental stimuli (Carlson et al., 2009). Some of these e.g., BA3828 and BA0391 are transcription factors which are differentially regulated between the two groups. The former is specific to the TCA group and the latter the AP IVDU group. These merit further follow up as they may be of significance in pathogenicity and involved in development of the different clinical disease presentations observed in patients. Similar studies have been conducted previously however the complement of proteins presented in this study do not share antigen features in common (Kudva et al., 2005; Pflughoeft et al., 2014).

Good recognition of PA and LF was observed for all the vaccinated and infected/challenged groups, however recognition of EF was only observed in the AVPV group for IgG and weakly by the BWS and AN IVDU groups. Some weak recognition of EF was seen with the control group. IgA recognition of EF was seen mainly with the AVPV and TCA and more weakly for the BWS and AN IVDU groups. Again, some weak IgA recognition of EF was seen with the control group. Thus, despite the fact that edema is a common feature of disease in the TCA and particularly the AP IVDU group, little significant antibody response is seen to this toxin component in the infected groups. The IgG anti-toxin response is more marked in the AVPV group, although some IgA reactivity is also seen, however the predominant anti-toxin response in the AP IVDU and TCA infected groups is with IgA. These observations are confirmed from comparative rabbit live spore and AVP vaccination model data. The anti-toxin response in AVP vaccinated rabbits is biased toward IgG, whereas the anti-toxin response in spore vaccinated rabbits is biased toward IgA. Regulation of the IgA response although not fully understood may be via invariant natural killer T (iNKT) (MacLeod et al., 2013; Zeng et al., 2013) or gamma delta T cells (Fujihashi et al., 1996; Tezuka et al., 2011). This may perhaps imply involvement of these cell types during active *B. anthracis* infection. The means by which live spore challenge elicits this IgA-biased response strongly merits further investigation.

Although immunogenic proteins other than the toxin components were identified in this study, some of which appear to be specifically recognized by certain groups, none appeared to be *B. anthracis* specific and of a size amenable to chemical synthesis for further analysis. We therefore investigated the repertoire of conserved hypothetical and hypothetical proteins represented in the *B. anthracis* genome using the database comparison tool BLAST. Five proteins were identified of a size amenable to chemical synthesis, two of which exhibited *B. anthracis* specificity, two exhibiting partial protein sequence similarity and one exhibiting sequence conservation with other closely related bacillus species. These were used in ELISAs to determine their antibody reactivity with the control, AVPV, TCA, and AP IVDU groups in comparison to PA and LF as positive controls. Only BA0448 exhibited IgG reactivity in the AP IVDU group. The R value for this antigen was less than that observed for LF and greater than that for PA, indicating that this peptide and LF would be more useful for diagnostic detection in the AP IVDU group than PA for IgG. However, all five peptides exhibited good reactivity for IgA for the AP IVDU and TCA groups and four of five for the AVPV group. This indicates that all five are recognized by infected individuals, suggesting that they are expressed during infection and four of five are recognized by vaccinees indicating that they are present in the UK AVP vaccine. The *R*-values for the AP IVDU group were higher than that for either PA or LF, indicating that all five peptides are recognized with good specificity by this infected group and would be useful in a diagnostic context. All peptides exhibited poorer specificity for the TCA and also the AVPV groups than the toxin components, based on *R*-values. Therefore, four of the five proteins appear to be expressed both during vegetative growth *in vitro* and during infection; whereas BA2182 is not recognized by the AVPV group so may be more specifically expressed during infection only.

Little is known about the function of these peptides other than for BA4898 which is a spore associated protein and is associated with the exosporium. The expression profile or the function of the other four hypothetical proteins either *in vitro* or *vivo* is unknown; however their recognition by immune sera would suggest they are actively expressed by *B. anthracis* cells. There are a very large number of (1) conserved hypothetical and (2) hypothetical proteins in the *B. anthracis* genome and pXO1 and pXO2 plasmids. In this study we observed that across all groups (1) 51.13% of the genomic and 63.64% of the plasmid conserved hypothetical proteins and (2) 52.74% of the genomic and 64.66% of the plasmid hypothetical proteins were recognized as statistically significant by IgG binding. Whereas, (1) 27.57% of the genomic and 18.18% of the plasmid conserved hypothetical proteins, and (2) 19.19% of the genomic and 10.53% of the plasmid hypothetical proteins were recognized by IgA of these entities featured on the array (data not shown). This demonstrates that a large number of these may be expressed *in vitro* and *in vivo* and are recognized by both IgG and IgA. They also exhibit broadly group specific recognition profiles. It also parallels the expression distribution data which show a narrower range of recognition of proteins by IgA than for IgG. These peptides may be of great value in differential diagnosis of Anthrax infection, however as they were found not to be immunogenic in a mouse vaccination study their use as vaccine or vaccine components is uncertain. Perhaps in combination vaccines with other proteins or adjuvants they may be immunogenic, but they proved non-immunogenic as single peptide entities in this study.

In summary, the Anthrax ProtoArray has identified many immunogenic proteins in both vaccinated and Anthrax challenged groups. However, overall recognition of Anthrax proteins was somewhat more reduced than expected in the challenged groups when compared to naive controls. Of the proteins recognized by the vaccinated and Anthrax challenged groups, the toxins proved the most immune-dominant both for IgG and IgA recognition. Other proteins were also recognized in challenged groups which were not recognized by controls, some of which exhibited group specificity. These may be due to differing recognition related to specific infection or challenge routes or by differential expression of protein “infectome” by the Anthrax bacillus in response to different environmental stimuli. Further work is required to determine the underlying immunopathological basis for the IgA biased protein recognition and also the apparent IgG immunosuppression observed in the infected groups, and to determine the function of the identified proteins in bacterial pathogenesis and their potential utility as diagnostic or vaccine targets.

### Conflict of interest statement

The Associate Editor Diane Williamson declares that, despite having co-hosted a Frontiers Research Topic with author Bassam Hallis, the review process was handled objectively. The authors declare that the research was conducted in the absence of any commercial or financial relationships that could be construed as a potential conflict of interest.

## Footnotes

^1^https://www.gov.uk/government/collections/rare-and-imported-pathogens-laboratory-ripl

^2^http://www.thermofisher.com/en/about-us/invitrogen.html

^3^http://www.R-project.org/

^4^http://www.peptidesynthetics.co.uk/

^5^http://blast.ncbi.nlm.nih.gov/Blast.cgi
