# Supplementary material for: Whole genome protein microarrays for serum profiling of immunodominant antigens of Bacillus anthracis
Source: Front Microbiol. 2015 Aug 13;6:747. doi: 10.3389/fmicb.2015.00747 (PMC4534840; doi:10.3389/fmicb.2015.00747)
Supplement: Supplementary file 1 [file DataSheet1.DOCX]

**Supplementary Information S1- All human sera primary screen**

ND – Not detected NA – Not available

| **Antigen** | **Anti-Toxin IgG ELISA (EC_50_)** | | | | | |
| --- | --- | --- | --- | --- | --- | --- |
| **Group** | **Control** | | | | | |
| **Donor Number** | 24 | 49 | 53 | 54 | 382 | 383 |
| **LF** | ND | ND | ND | ND | ND | ND |
| **PA** | ND | ND | ND | ND | ND | ND |
| **Group** | **Vaccinees** | | | | | |
| **Donor Number** | 371 | 411 | 412 | 494 | 721 | 722 |
| **LF** | ND | 241.6 | 32.91 | 64.08 | 64.65 | 230.3 |
| **PA** | 53.48 | 386.0 | 68.82 | 162.7 | 193.2 | 486.0 |
| **Group** | **Belgian Wool Sorters** | | | | | |
| **Donor Number** | 9 | 18 | 29 | 45 | 57 | 59 |
| **LF** | ND | 6.74 | 80.24 | ND | ND | 4.855 |
| **PA** | 23.77 | 7.453 | ND | 1.185 | ND | ND |
| **Group** | **Turkish Cutaneous Anthrax Patients** | | | | | |
| **Donor Number** | 1 | 2 | 3 | 4 | 5 | 6 |
| **LF** | 45.29 | 28.98 | 55.35 | 68.09 | 400.8 | 172.9 |
| **PA** | 4.311 | 12.75 | 12.62 | 10.06 | 59.85 | 46.82 |

| **Antigen** | **Anti-Toxin IgG ELISA (EC_50_)** | | | | | |
| --- | --- | --- | --- | --- | --- | --- |
| **Group** | **Intravenous Drug Users Anthrax Negative** | | | | | |
| **Donor Number** | 1253 | 2425 | 4307 | 5211 | 5338 | 7670 |
| **LF** | ND | 3 | ND | ND | ND | ND |
| **PA** | ND | ND | ND | ND | ND | ND |
| **Group** | **Intravenous Drug Users Anthrax Positive** | | | | | |
| **Donor Number** | 0061 | 0180 | 0261 | 0335 | 0336 | 0450 |
| **LF** | 360 | 5 | >30 | 15 | 1 | 162 |
| **PA** | 104 | 48 | 168 | 39 | ND | 188 |

| **Antigen** | **Anti-Toxin IgA ELISA (EC_50_)** | | | | | |
| --- | --- | --- | --- | --- | --- | --- |
| **Group** | **Control** | | | | | |
| **Donor Number** | 24 | 49 | 53 | 54 | 382 | 383 |
| **LF** | ND | ND | ND | ND | ND | ND |
| **PA** | ND | ND | ND | ND | 15.61 | 4.68 |
| **Group** | **Vaccinees** | | | | | |
| **Donor Number** | 371 | 411 | 412 | 494 | 721 | 722 |
| **LF** | ND | ND | ND | ND | ND | ND |
| **PA** | ND | 97.8 | 4.913 | 15.57 | ND | ND |
| **Group** | **Belgian Wool Sorters** | | | | | |
| **Donor Number** | 9 | 18 | 29 | 45 | 57 | 59 |
| **LF** | 5.875 | 10.86 | ND | ND | 5.401 | 13.82 |
| **PA** | ND | ND | ND | ND | 5.091 | ND |
| **Group** | **Turkish Cutaneous Anthrax Patients** | | | | | |
| **Donor Number** | 1 | 2 | 3 | 4 | 5 | 6 |
| **LF** | 11.15 | ND | 4.239 | 2.510 | 39.77 | 13.06 |
| **PA** | ND | 21.11 | 1.486 | 13.43 | 6.118 | ND |

| **Antigen** | **Anti-Toxin IgA ELISA (EC_50_)** | | | | | |
| --- | --- | --- | --- | --- | --- | --- |
| **Group** | **Intravenous Drug Users Anthrax Negative** | | | | | |
| **Donor Number** | 1253 | 2425 | 4307 | 5211 | 5338 | 7670 |
| **LF** | NA | NA | NA | NA | NA | NA |
| **PA** | NA | NA | NA | NA | NA | NA |
| **Group** | **Intravenous Drug Users Anthrax Positive** | | | | | |
| **Donor Number** | 0061 | 0180 | 0261 | 0335 | 0336 | 0450 |
| **LF** | NA | NA | NA | NA | NA | NA |
| **PA** | NA | NA | NA | NA | NA | NA |
