# Supplementary material for: Whole genome protein microarrays for serum profiling of immunodominant antigens of Bacillus anthracis
Source: Front Microbiol. 2015 Aug 13;6:747. doi: 10.3389/fmicb.2015.00747 (PMC4534840; doi:10.3389/fmicb.2015.00747)
Supplement: Supplementary file 2 [file DataSheet2.DOCX]

**Supplementary Information S2**

There is evident non-normal distribution in each of the untransformed IgG and IgA data sets (Figures A and B). The data were therefore transformed to a log_2_ scale (Figures C & D). The distributions have a non-parametric triphasic distribution. Further investigations revealed that the majority of the values at the lower intensity could be accounted for by “negative” controls (Empty/Blank/Buffer – red in Figures E and F) or “positive” or human-protein specific controls (green in Figures G and H). The *Bacillus anthracis* specific spots are more normally distributed (shown in blue in Figures I and J), however they still exhibit a bi-modal distribution. In order to prevent over-normalisation due to this apparent skewing of these data sets, *B. anthracis* -specific protein entity data points and the buffer control features were separated from the other array data. These were then normalised using the mean of the buffer feature intensities and analysed independently of the other feature [Empty/blank/human protein/other positive control] data.

**A**


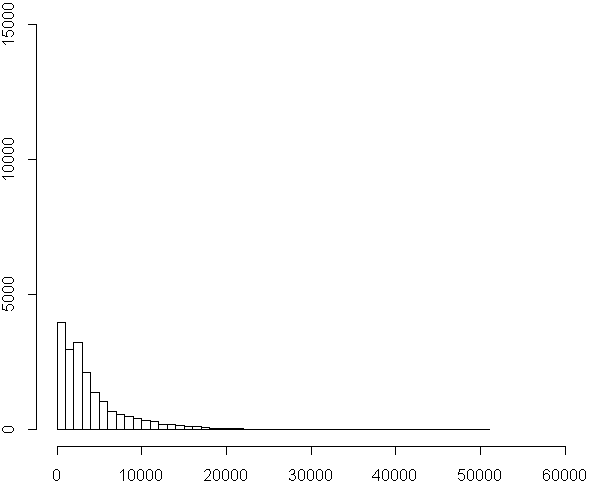


**Intensity**

**Frequency**

**B**


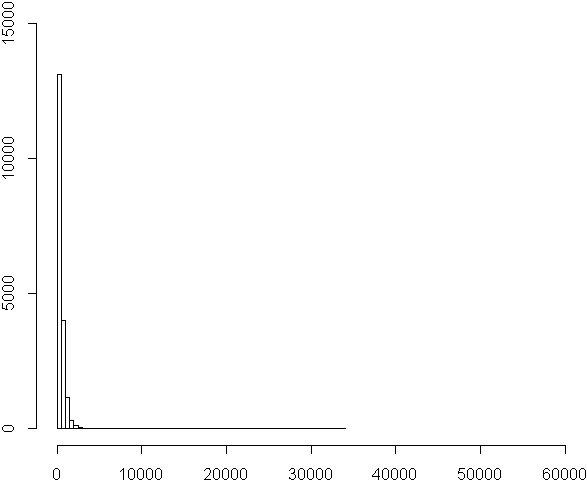


**Intensity**

**Frequency**

**Frequency**

**Intensity**

**C**


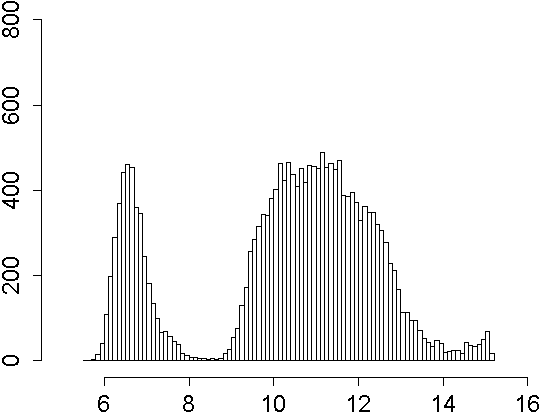


**Frequency**

**Intensity**

**D**


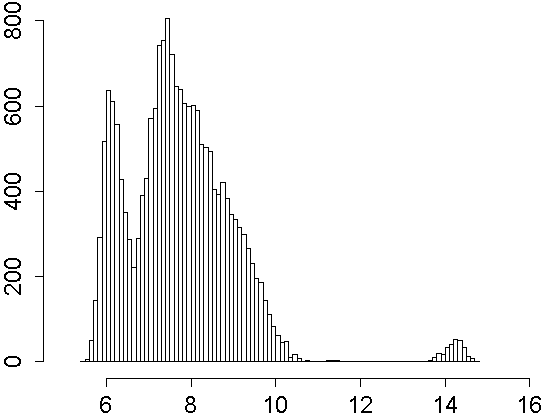


**Intensity**

**Frequency**

**E**


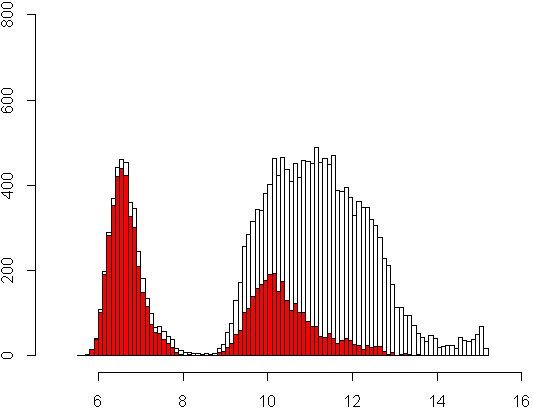


**Intensity**

**Frequency**

**F**


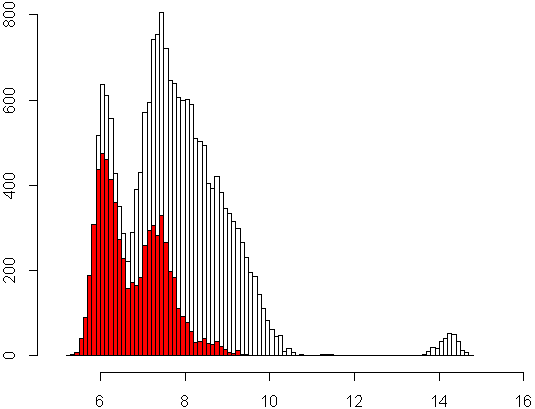


**Intensity**

**Frequency**

**G**


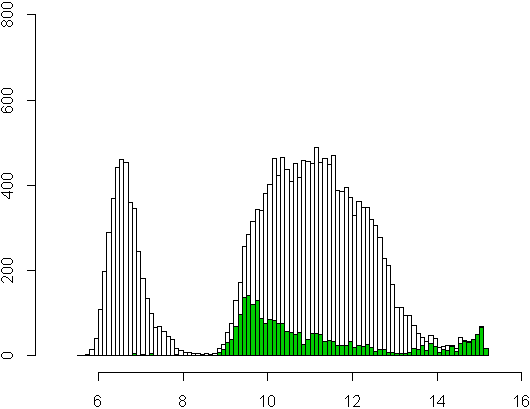


**Intensity**

**Frequency**

**H**


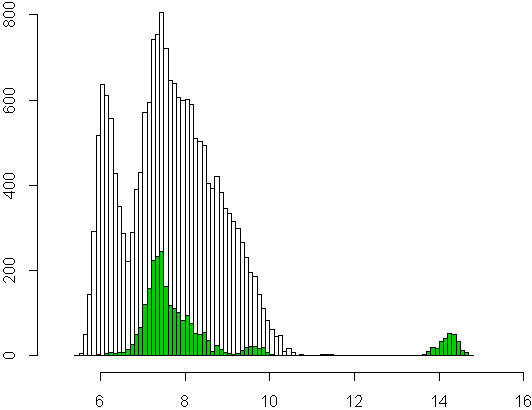


**Frequency**

**Intensity**

**I**


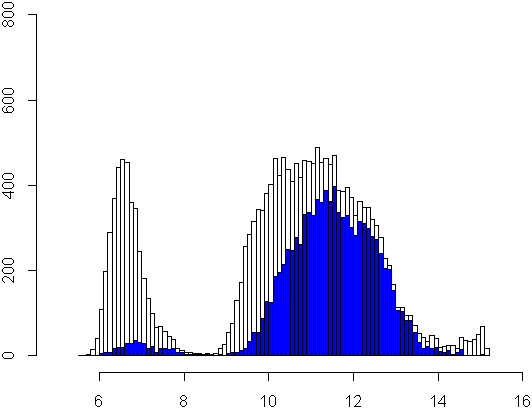


**Intensity**

**Frequency**

**J**


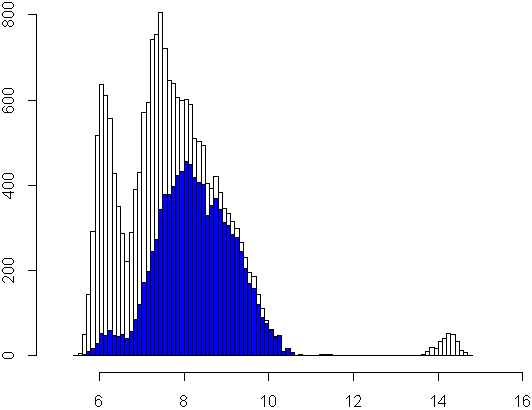


**Intensity**

**Frequency**

**Appendix I – R code used to generate histogram figures**

Background – IgG and IgA are dataframes consisting of 44 columns and 19200 rows. Columns 1:2 are probe NAME and probe ID, 39-44 are grid coordinates for the array, columns 3:38 are IgG raw intensity values from 6 sample groups (6 samples per group = 36): Control group (C), Belgian wool-sorters (B), naturally-infected Turkish (T), Vaccinees (V), Positive Heroin Users (P), Negative Heroin Users (N).Each row represents a separate spot on the array.

Values were averaged across each row before plotting.

######################################################

# repeat using the EF|LF|PA as well as the Ba

# for paper - remove legends and increase font of axes

#IgG data - watch the breaks

#07Apr15

# main hist

hist(apply(log2(IgG_PN[grep("", IgG_PN$NAME),][,3:38]), 1, mean), freq=T, xlim=c(5,17),ylim=c(0,950), breaks=100, main="", xlab="", ylab="",cex.axis=1.5)

#IgA data - watch the breaks

#07Apr15

# main hist

hist(apply(log2(IgA_PN[grep("", IgA_PN$NAME),][,3:38]), 1, mean), freq=T, xlim=c(5,17),ylim=c(0,950), breaks=100, main="", xlab="", ylab="",cex.axis=1.5)

#IgG data - watch the breaks

#07Apr15

# main hist

hist(apply(log2(IgG_PN[grep("", IgG_PN$NAME),][,3:38]), 1, mean), freq=T, xlim=c(5,17),ylim=c(0,950), breaks=100, main="", xlab="", ylab="",cex.axis=1.5)

# add "Ba|EF|LF|PA" - grey

hist(apply(log2(IgG_PN[grep("Ba|EF|LF|PA", IgG_PN$NAME),][,3:38]), 1, mean), ylim=c(0,950), breaks=100, add=T, col="grey")

# add Empty - red

hist(apply(log2(IgG_PN[grep("Empty", IgG_PN$NAME),][,3:38]), 1, mean), ylim=c(0,950), breaks=100, add=T, col="red")

# add Blank - Blue

hist(apply(log2(IgG_PN[grep("Blank", IgG_PN$NAME),][,3:38]), 1, mean), ylim=c(0,950), breaks=30, add=T, col="blue")

# add Positive Controls - Green

hist(apply(log2(IgG_PN[grep("Alexa|AntiBiotin|Anti-human-IgG|Biotin|BSA|Calmodulin|CMK|GST|HumanIgG|Internal|Kinase|MAPKAP|V5Control", IgG_PN$NAME),][,3:38]), 1, mean), xlim=c(0,16), ylim=c(0,600), breaks=100, add=T, col="green")

# add Buffer - Pink

hist(apply(log2(IgG_PN[grep("Buffer", IgG_PN$NAME),][,3:38]), 1, mean), ylim=c(0,950), breaks=20, add=T, col="pink")

#IgA data - watch the breaks

#07Apr15

# main hist

hist(apply(log2(IgA_PN[grep("", IgA_PN$NAME),][,3:38]), 1, mean), freq=T, xlim=c(5,17),ylim=c(0,950), breaks=100, main="", xlab="", ylab="", cex.axis=1.5)

# add "Ba" - grey

hist(apply(log2(IgA_PN[grep("Ba|EF|LF|PA", IgA_PN$NAME),][,3:26]), 1, mean), ylim=c(0,950), breaks=80, add=T, col="grey")

# add Empty - red

hist(apply(log2(IgA_PN[grep("Empty", IgA_PN$NAME),][,3:38]), 1, mean), ylim=c(0,950), breaks=50, add=T, col="red")

# add Blank - Blue

hist(apply(log2(IgA_PN[grep("Blank", IgA_PN$NAME),][,3:38]), 1, mean), ylim=c(0,950), breaks=20, add=T, col="blue")

# add Positive Controls - Green

hist(apply(log2(IgA_PN[grep("Alexa|AntiBiotin|Anti-human-IgG|Biotin|BSA|Calmodulin|CMK|GST|HumanIgG|Internal|Kinase|MAPKAP|V5Control", IgA_PN$NAME),][,3:38]), 1, mean), xlim=c(0,16), ylim=c(0,600), breaks=80, add=T, col="green")

# add Buffer - Pink

hist(apply(log2(IgA_PN[grep("Buffer", IgG_PN$NAME),][,3:38]), 1, mean), ylim=c(0,950), breaks=10, add=T, col="pink")

#untransformed data

# IgG

hist(apply((IgG_PN[grep("", IgG_PN$NAME),][,3:38]), 1, mean), freq=T, breaks=60, xlim=c(0,60000),ylim=c(0,15000),main="", xlab="")

#IgA

hist(apply((IgA_PN[grep("", IgA_PN$NAME),][,3:38]), 1, mean), freq=T, breaks=60, xlim=c(0,60000),ylim=c(0,15000),main="", xlab="")

par(mfrow=c(1,2))

#transformed data

hist(apply(log2(IgG_PN[grep("", IgG_PN$NAME),][,3:38]), 1, mean), freq=T, breaks=100, xlim=c(6,15),ylim=c(0,950),main="", xlab="")

hist(apply(log2_PN(IgA[grep("", IgA_PN$NAME),][,3:38]), 1, mean), freq=T, breaks=100, xlim=c(6,15),ylim=c(0,950),main="", xlab="")

par(mfrow=c(1,1))

#transformed IgG with empty blank buffer overlaid

hist(apply(log2(IgG_PN[grep("", IgG_PN$NAME),][,3:38]), 1, mean), freq=T, breaks=100, xlim=c(5,17),ylim=c(0,950),main="", xlab="")

hist(apply(log2(IgG_PN[grep("Empty|Blank|Buffer", IgG_PN$NAME),][,3:38]), 1, mean), freq=T, breaks=100, xlim=c(6,15),ylim=c(0,500),main="", xlab="", col=2, add=T)

#transformed IgA with empty blank buffer overlaid

hist(apply(log2(IgA_PN[grep("", IgA_PN$NAME),][,3:38]), 1, mean), freq=T, breaks=100, xlim=c(5,17),ylim=c(0,950),main="", xlab="")

hist(apply(log2(IgA_PN[grep("Empty|Blank|Buffer", IgA_PN$NAME),][,3:26]), 1, mean), freq=T, breaks=50, xlim=c(6,15),ylim=c(0,500),main="", xlab="", col=2, add=T)

#transformed IgG with positive controls overlaid

hist(apply(log2(IgG_PN[grep("", IgG_PN$NAME),][,3:38]), 1, mean), freq=T, breaks=100, xlim=c(5,17),ylim=c(0,950),main="", xlab="")

hist(apply(log2(IgG_PN[grep("Alexa|AntiBiotin|Anti-human-IgG|Biotin|BSA|Calmodulin|CMK|GST|HumanIgG|Internal|Kinase|MAPKAP|V5Control", IgG_PN$NAME),][,3:38]), 1, mean), freq=T, breaks=100, xlim=c(5,17),ylim=c(0,950),main="", xlab="", col=3, add=T)

#transformed IgA with positive controls overlaid

hist(apply(log2(IgA_PN[grep("", IgA_PN$NAME),][,3:38]), 1, mean), freq=T, breaks=100, xlim=c(5,17),ylim=c(0,950),main="", xlab="")

hist(apply(log2(IgA_PN[grep("Alexa|AntiBiotin|Anti-human-IgG|Biotin|BSA|Calmodulin|CMK|GST|HumanIgG|Internal|Kinase|MAPKAP|V5Control", IgA_PN$NAME),][,3:38]), 1, mean), freq=T, breaks=100, xlim=c(5,17),ylim=c(0,950),main="", xlab="", col=3, add=T)

#transformed IgG with Ba|EF|LF|PA overlaid

hist(apply(log2(IgG_PN[grep("", IgG_PN$NAME),][,3:38]), 1, mean), freq=T, breaks=100, xlim=c(5,17),ylim=c(0,950),main="", xlab="")

hist(apply(log2(IgG_PN[grep("Ba|EF|LF|PA", IgG_PN$NAME),][,3:38]), 1, mean), freq=T, breaks=100, xlim=c(5,17),ylim=c(0,950),main="", xlab="", col=4, add=T)

#transformed IgA with positive controls overlaid

hist(apply(log2(IgA_PN[grep("", IgA_PN$NAME),][,3:38]), 1, mean), freq=T, breaks=100, xlim=c(5,17),ylim=c(0,950),main="", xlab="")

hist(apply(log2(IgA_PN[grep("Ba|EF|LF|PA", IgA_PN$NAME),][,3:38]), 1, mean), freq=T, breaks=50, xlim=c(5,17),ylim=c(0,950),main="", xlab="", col=4, add=T)

par(mfrow=c(1,1))

#################################################
