# Supplementary material for: Whole genome protein microarrays for serum profiling of immunodominant antigens of Bacillus anthracis
Source: Front Microbiol. 2015 Aug 13;6:747. doi: 10.3389/fmicb.2015.00747 (PMC4534840; doi:10.3389/fmicb.2015.00747)
Supplement: Supplementary file 3 [file DataSheet3.DOCX]

**Supplementary Information S3:** (a) Venn Diagram of Unique and Shared Features between Anti-IgG and IgA Recognised Anthrax Proteins (b) T1080 IgG feature List Ranked on Corrected P value (p < 0.05) (c) T1080 IgA feature List Ranked on Corrected P value (p < 0.05)

**A**


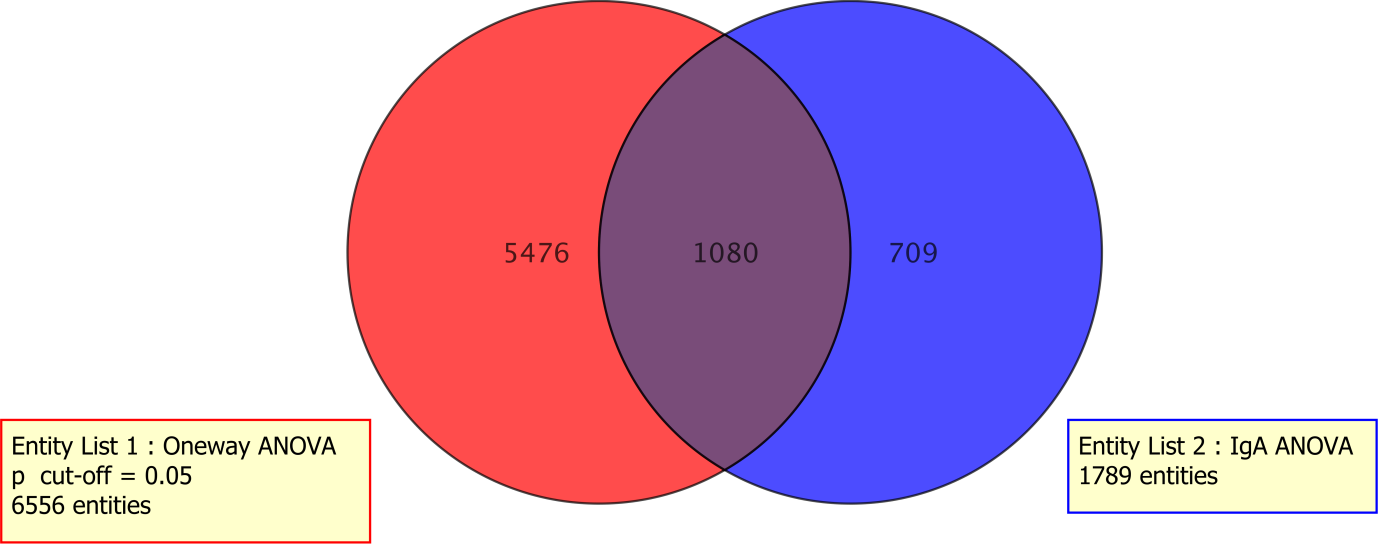


**B**

| **ID** | **p value** | **Protein Feature Name** |
| --- | --- | --- |
| Anthrax008~B06R18C17 | 2.59E-10 | EF_0.78125n~N/A |
| Anthrax008~B40R02C07 | 1.13E-09 | Ba~MGC:BA1458~BA696442~RFU:0 |
| Anthrax008~B06R18C19 | 1.13E-08 | EF_0.390625n~N/A |
| Anthrax008~B38R01C19 | 7.68E-08 | Ba~MGC:BA1881~BA696656~RFU:0 |
| Anthrax008~B34R02C18 | 2.02E-07 | Ba~MGC:BA4205~BA697783~RFU:2.76 |
| Anthrax008~B47R02C15 | 6.65E-07 | Ba~MGC:BA2752~BA697068~RFU:0 |
| Anthrax008~B14R18C17 | 1.52E-06 | LF_0.78125n~N/A |
| Anthrax008~B48R14C14 | 2.96E-06 | Ba~MGC:BA0007~BA698722~RFU:256.08 |
| Anthrax008~B48R02C07 | 2.76E-06 | Ba~MGC:BA0639~BA696061~RFU:4.6 |
| Anthrax008~B22R18C05 | 2.05E-06 | PA_50n~N/A |
| Anthrax008~B06R18C13 | 2.80E-06 | EF_3.125n~N/A |
| Anthrax008~B22R18C07 | 3.30E-06 | PA_25n~N/A |
| Anthrax008~B45R03C01 | 3.22E-06 | Ba~MGC:BA3842~BA697593~RFU:49942.78 |
| Anthrax008~B14R18C19 | 3.69E-06 | LF_0.390625n~N/A |
| Anthrax008~B48R02C12 | 4.31E-06 | Ba~MGC:BA1458~BA696442~RFU:22.99 |
| Anthrax008~B30R01C19 | 4.62E-06 | Ba~MGC:BA3349~BA697346~RFU:23.91 |
| Anthrax008~B12R06C03 | 4.37E-06 | Ba~MGC:BA0472~BA695986~RFU:60024.83 |
| Anthrax008~B14R01C17 | 5.43E-06 | Ba~MGC:BA4715~BA698051~RFU:0 |
| Anthrax008~B48R14C13 | 6.37E-06 | Ba~MGC:BA0007~BA698722~RFU:595.14 |
| Anthrax008~B40R02C09 | 8.65E-06 | Ba~MGC:BA4769~BA698080~RFU:0 |
| Anthrax008~B34R02C17 | 9.30E-06 | Ba~MGC:BA4205~BA697783~RFU:0 |
| Anthrax008~B38R08C11 | 1.71E-05 | Ba~MGC:BA2384~BA13716~RFU:0 |
| Anthrax008~B22R01C17 | 1.73E-05 | Ba~MGC:BA4774~BA698082~RFU:0 |
| Anthrax008~B04R03C20 | 1.90E-05 | Ba~MGC:BA0129~BA695800~RFU:60011.04 |
| Anthrax008~B06R18C07 | 1.95E-05 | EF_25n~N/A |
| Anthrax008~B13R05C14 | 2.66E-05 | Ba~MGC:BA0943~BA696210~RFU:58423.47 |
| Anthrax008~B30R01C17 | 2.43E-05 | Ba~MGC:BA0216~BA695848~RFU:0 |
| Anthrax008~B48R02C08 | 2.41E-05 | Ba~MGC:BA0639~BA696061~RFU:0 |
| Anthrax008~B22R18C03 | 2.29E-05 | PA_100n~N/A |
| Anthrax008~B46R08C15 | 2.54E-05 | Ba~MGC:BA1571~BA14664~RFU:0 |
| Anthrax008~B45R03C02 | 2.64E-05 | Ba~MGC:BA3842~BA697593~RFU:25666.67 |
| Anthrax008~B08R03C19 | 2.98E-05 | Ba~MGC:BA5350~BA698379~RFU:60038.63 |
| Anthrax008~B24R01C19 | 3.27E-05 | Ba~MGC:BA1534~BA696478~RFU:0 |
| Anthrax008~B48R08C06 | 3.20E-05 | Ba~MGC:BA2500~BA14596~RFU:0 |
| Anthrax008~B06R05C20 | 3.22E-05 | Ba~MGC:BA4061~BA697717~RFU:60035.87 |
| Anthrax008~B24R08C14 | 3.78E-05 | Ba~MGC:BA2441~BA15578~RFU:19.32 |
| Anthrax008~B29R14C15 | 3.80E-05 | Ba~MGC:BA2554~BA696979~RFU:4297.57 |
| Anthrax008~B06R05C19 | 3.73E-05 | Ba~MGC:BA4061~BA697717~RFU:60042.31 |
| Anthrax008~B32R08C19 | 3.79E-05 | Ba~MGC:BA2440~BA15228~RFU:0 |
| Anthrax008~B27R04C20 | 3.95E-05 | Ba~MGC:BA5718~BA698567~RFU:60042.31 |
| Anthrax008~B06R08C15 | 4.43E-05 | Ba~MGC:BA2716~BA15164~RFU:3.68 |
| Anthrax008~B14R18C11 | 4.43E-05 | LF_6.25n~N/A |
| Anthrax008~B38R04C02 | 4.72E-05 | Ba~MGC:BA1897~BA696662~RFU:59866.63 |
| Anthrax008~B27R04C19 | 4.77E-05 | Ba~MGC:BA5718~BA698567~RFU:60029.43 |
| Anthrax008~B13R05C13 | 5.00E-05 | Ba~MGC:BA0943~BA696210~RFU:46536.06 |
| Anthrax008~B18R02C12 | 5.68E-05 | Ba~MGC:BA0462~BA695982~RFU:60079.1 |
| Anthrax008~B46R02C17 | 5.79E-05 | Ba~MGC:BA3953~BA697653~RFU:54808.68 |
| Anthrax008~B18R02C11 | 6.48E-05 | Ba~MGC:BA0462~BA695982~RFU:60069.9 |
| Anthrax008~B40R02C08 | 5.98E-05 | Ba~MGC:BA1458~BA696442~RFU:5.52 |
| Anthrax008~B37R02C17 | 5.29E-05 | Ba~MGC:BA3828~BA697587~RFU:0 |
| Anthrax008~B48R08C19 | 6.42E-05 | Ba~MGC:BA1929~BA14640~RFU:0 |
| Anthrax008~B43R08C11 | 5.65E-05 | Ba~MGC:BA1166~BA14740~RFU:21911.3 |
| Anthrax008~B48R01C17 | 5.95E-05 | Ba~MGC:BA1797~BA696613~RFU:0 |
| Anthrax008~B38R04C01 | 6.40E-05 | Ba~MGC:BA1897~BA696662~RFU:59977.92 |
| Anthrax008~B32R08C09 | 6.31E-05 | Ba~MGC:BA2612~BA14310~RFU:0 |
| Anthrax008~B10R13C10 | 5.42E-05 | Ba~MGC:BA5061~BA5061~RFU:14127.32 |
| Anthrax008~B19R08C05 | 6.43E-05 | Ba~MGC:BA1499~BA15288~RFU:6117.55 |
| Anthrax008~B26R03C17 | 6.71E-05 | Ba~MGC:BA1531~BA696476~RFU:59986.2 |
| Anthrax008~B35R14C03 | 6.86E-05 | Ba~MGC:BXA0085~BA698611~RFU:15662.37 |
| Anthrax008~B24R08C15 | 7.26E-05 | Ba~MGC:BA2756~BA15130~RFU:0 |
| Anthrax008~B08R03C20 | 7.15E-05 | Ba~MGC:BA5350~BA698379~RFU:60071.74 |
| Anthrax008~B35R14C05 | 7.06E-05 | Ba~MGC:BA2916~BA697144~RFU:35849.89 |
| Anthrax008~B16R04C03 | 7.47E-05 | Ba~MGC:BA3555~BA697455~RFU:60042.31 |
| Anthrax008~B21R04C11 | 7.65E-05 | Ba~MGC:BA4778~BA698083~RFU:60030.35 |
| Anthrax008~B32R08C15 | 7.76E-05 | Ba~MGC:BA2750~BA15686~RFU:0 |
| Anthrax008~B11R07C06 | 8.00E-05 | Ba~MGC:BA4385~BA697886~RFU:14023.18 |
| Anthrax008~B47R03C01 | 8.14E-05 | Ba~MGC:BA4825~BA698107~RFU:60057.03 |
| Anthrax008~B16R04C04 | 8.36E-05 | Ba~MGC:BA3555~BA697455~RFU:59896.06 |
| Anthrax008~B26R03C18 | 8.28E-05 | Ba~MGC:BA1531~BA696476~RFU:60058.87 |
| Anthrax008~B32R01C18 | 8.62E-05 | Ba~MGC:BA3461~BA697403~RFU:0 |
| Anthrax008~B48R08C18 | 8.54E-05 | Ba~MGC:BA1788~BA14066~RFU:13.8 |
| Anthrax008~B11R08C14 | 8.95E-05 | Ba~MGC:BA1032~BA14952~RFU:2930.46 |
| Anthrax008~B33R08C14 | 9.42E-05 | Ba~MGC:BA1084~BA14368~RFU:2107.3 |
| Anthrax008~B43R01C17 | 9.58E-05 | Ba~MGC:BA0669~BA696075~RFU:40776.82 |
| Anthrax008~B40R01C20 | 9.80E-05 | Ba~MGC:BA1502~BA696462~RFU:0 |
| Anthrax008~B03R08C10 | 9.98E-05 | Ba~MGC:BA1054~BA14686~RFU:5529.8 |
| Anthrax008~B06R08C17 | 1.16E-04 | Ba~MGC:BA2563~BA13218~RFU:0 |
| Anthrax008~B32R01C20 | 1.12E-04 | Ba~MGC:BA3088~BA697240~RFU:0 |
| Anthrax008~B32R01C19 | 1.11E-04 | Ba~MGC:BA3088~BA697240~RFU:7.36 |
| Anthrax008~B43R01C18 | 1.16E-04 | Ba~MGC:BA0669~BA696075~RFU:41430.62 |
| Anthrax008~B44R05C04 | 1.17E-04 | Ba~MGC:BA0135~BA695802~RFU:59994.48 |
| Anthrax008~B03R05C15 | 1.13E-04 | Ba~MGC:BA4303~BA697832~RFU:60059.79 |
| Anthrax008~B42R06C07 | 1.19E-04 | Ba~MGC:BA3985~BA697675~RFU:0 |
| Anthrax008~B08R08C17 | 1.21E-04 | Ba~MGC:BA2287~BA14950~RFU:4802.24 |
| Anthrax008~B34R12C09 | 1.24E-04 | Ba~MGC:BA4649~BA13315~RFU:21499.26 |
| Anthrax008~B06R18C05 | 1.28E-04 | EF_50n~N/A |
| Anthrax008~B43R03C08 | 1.30E-04 | Ba~MGC:BA4084~BA697724~RFU:60053.35 |
| Anthrax008~B03R05C16 | 1.30E-04 | Ba~MGC:BA4303~BA697832~RFU:60068.06 |
| Anthrax008~B06R08C16 | 1.33E-04 | Ba~MGC:BA2716~BA15164~RFU:0 |
| Anthrax008~B03R08C09 | 1.40E-04 | Ba~MGC:BA1054~BA14686~RFU:5399.19 |
| Anthrax008~B34R13C08 | 1.36E-04 | Ba~MGC:BA4761~BA4761~RFU:23427.15 |
| Anthrax008~B42R05C17 | 1.37E-04 | Ba~MGC:BA5737~BA698575~RFU:60077.26 |
| Anthrax008~B35R04C13 | 1.35E-04 | Ba~MGC:BA5574~BA698492~RFU:59736.02 |
| Anthrax008~B14R08C19 | 1.39E-04 | Ba~MGC:BA2716~BA15164~RFU:11462.47 |
| Anthrax008~B38R11C19 | 1.39E-04 | Ba~MGC:BA0134~BA12564~RFU:16967.1 |
| Anthrax008~B38R08C09 | 1.46E-04 | Ba~MGC:BA2376~BA14746~RFU:0 |
| Anthrax008~B22R11C09 | 1.45E-04 | Ba~MGC:BA0094~BA0094~RFU:51357.62 |
| Anthrax008~B14R05C15 | 1.48E-04 | Ba~MGC:BA1373~BA696403~RFU:0 |
| Anthrax008~B47R03C07 | 1.55E-04 | Ba~MGC:BA0324~BA695907~RFU:0 |
| Anthrax008~B22R18C13 | 1.54E-04 | PA_3.125n~N/A |
| Anthrax008~B46R01C17 | 1.62E-04 | Ba~MGC:BA0749~BA698739~RFU:0 |
| Anthrax008~B42R05C18 | 1.57E-04 | Ba~MGC:BA5737~BA698575~RFU:60109.46 |
| Anthrax008~B35R05C01 | 1.62E-04 | Ba~MGC:BA1837~BA696637~RFU:59951.25 |
| Anthrax008~B39R02C15 | 1.60E-04 | Ba~MGC:BA1563~BA696496~RFU:17.17 |
| Anthrax008~B21R04C12 | 1.51E-04 | Ba~MGC:BA4778~BA698083~RFU:59861.11 |
| Anthrax008~B35R04C14 | 1.61E-04 | Ba~MGC:BA5574~BA698492~RFU:42716.15 |
| Anthrax008~B34R12C20 | 1.53E-04 | Ba~MGC:BA1851~BA13495~RFU:36729.61 |
| Anthrax008~B38R11C20 | 1.67E-04 | Ba~MGC:BA0134~BA12564~RFU:15288.98 |
| Anthrax008~B38R01C17 | 1.70E-04 | Ba~MGC:BA1569~BA696501~RFU:37.71 |
| Anthrax008~B43R14C11 | 1.77E-04 | Ba~MGC:BA5637~BA698522~RFU:0 |
| Anthrax008~B22R18C18 | 1.71E-04 | PA_0.78125n~N/A |
| Anthrax008~B35R05C02 | 1.75E-04 | Ba~MGC:BA1837~BA696637~RFU:60025.75 |
| Anthrax008~B03R03C17 | 1.77E-04 | Ba~MGC:BA2377~BA696904~RFU:60080.94 |
| Anthrax008~B26R04C06 | 1.77E-04 | Ba~MGC:BA0116~BA695794~RFU:60076.34 |
| Anthrax008~B22R18C01 | 1.89E-04 | PA_200n~N/A |
| Anthrax008~B37R14C15 | 1.91E-04 | Ba~MGC:BA3036~BA13665~RFU:15184.55 |
| Anthrax008~B34R13C03 | 1.90E-04 | Ba~MGC:BA5051~BA5051~RFU:2767.66 |
| Anthrax008~B34R12C15 | 1.90E-04 | Ba~MGC:BXB0086~BA13595~RFU:13389.13 |
| Anthrax008~B03R03C18 | 1.84E-04 | Ba~MGC:BA2377~BA696904~RFU:60051.51 |
| Anthrax008~B33R08C13 | 2.03E-04 | Ba~MGC:BA1084~BA14368~RFU:2203.15 |
| Anthrax008~B26R04C05 | 2.06E-04 | Ba~MGC:BA0116~BA695794~RFU:60056.11 |
| Anthrax008~B38R02C09 | 2.05E-04 | Ba~MGC:BA1329~BA696384~RFU:0 |
| Anthrax008~B18R15C07 | 1.95E-04 | Ba~MGC:BA1809~BA14846~RFU:60074.5 |
| Anthrax008~B34R12C16 | 1.98E-04 | Ba~MGC:BXB0086~BA13595~RFU:16061.52 |
| Anthrax008~B32R08C11 | 2.05E-04 | Ba~MGC:BA2541~BA15044~RFU:2894.13 |
| Anthrax008~B35R14C06 | 1.97E-04 | Ba~MGC:BA2916~BA697144~RFU:33222.04 |
| Anthrax008~B37R16C19 | 2.05E-04 | Ba~MGC:BA5534~BA698473~RFU:14529.33 |
| Anthrax008~B24R01C18 | 2.11E-04 | Ba~MGC:BA5458~BA698440~RFU:0 |
| Anthrax008~B48R08C13 | 2.12E-04 | Ba~MGC:BA1580~BA14210~RFU:0 |
| Anthrax008~B33R05C12 | 2.22E-04 | Ba~MGC:BA4819~BA698103~RFU:59986.2 |
| Anthrax008~B18R10C05 | 2.10E-04 | Ba~MGC:BA4120~BA15438~RFU:0 |
| Anthrax008~B46R02C20 | 2.20E-04 | Ba~MGC:BA0113~BA695793~RFU:60046.91 |
| Anthrax008~B46R02C19 | 2.14E-04 | Ba~MGC:BA0113~BA695793~RFU:59969.65 |
| Anthrax008~B48R08C09 | 2.17E-04 | Ba~MGC:BA2691~BA14402~RFU:2.76 |
| Anthrax008~B47R01C20 | 2.21E-04 | Ba~MGC:BA4022~BA697695~RFU:19238.91 |
| Anthrax008~B38R08C17 | 2.21E-04 | Ba~MGC:BA2152~BA15662~RFU:0 |
| Anthrax008~B14R08C17 | 2.25E-04 | Ba~MGC:BA2707~BA14216~RFU:0 |
| Anthrax008~B07R03C03 | 2.26E-04 | Ba~MGC:BA4536~BA697963~RFU:0 |
| Anthrax008~B14R08C15 | 2.28E-04 | Ba~MGC:BA2441~BA15578~RFU:0 |
| Anthrax008~B04R16C01 | 2.30E-04 | Ba~MGC:BA1484~BA696453~RFU:46380.61 |
| Anthrax008~B19R08C15 | 2.35E-04 | Ba~MGC:BA0986~BA14300~RFU:24148.27 |
| Anthrax008~B46R03C16 | 2.37E-04 | Ba~MGC:BA1143~BA696293~RFU:59965.97 |
| Anthrax008~B32R02C06 | 2.37E-04 | Ba~MGC:BA3903~BA697627~RFU:0 |
| Anthrax008~B21R14C14 | 2.46E-04 | Ba~MGC:BA0114~BA13534~RFU:60118.65 |
| Anthrax008~B18R15C11 | 2.47E-04 | Ba~MGC:BA5276~BA16385~RFU:28578.92 |
| Anthrax008~B03R08C07 | 2.53E-04 | Ba~MGC:BA1167~BA15724~RFU:7369.39 |
| Anthrax008~B47R03C02 | 2.55E-04 | Ba~MGC:BA4825~BA698107~RFU:60025.75 |
| Anthrax008~B34R13C04 | 2.55E-04 | Ba~MGC:BA5051~BA5051~RFU:2511.04 |
| Anthrax008~B06R18C01 | 2.59E-04 | EF_200n~N/A |
| Anthrax008~B17R05C10 | 2.67E-04 | Ba~MGC:BA1349~BA696395~RFU:59866.63 |
| Anthrax008~B34R12C18 | 2.74E-04 | Ba~MGC:BA3948~BA3948~RFU:19295.44 |
| Anthrax008~B46R08C10 | 2.78E-04 | Ba~MGC:BA2668~BA16150~RFU:8.28 |
| Anthrax008~B38R08C18 | 2.79E-04 | Ba~MGC:BA2152~BA15662~RFU:17.48 |
| Anthrax008~B14R08C20 | 2.81E-04 | Ba~MGC:BA2716~BA15164~RFU:12843.08 |
| Anthrax008~B11R06C14 | 2.85E-04 | Ba~MGC:BA3105~BA697252~RFU:19001.1 |
| Anthrax008~B08R07C11 | 2.84E-04 | Ba~MGC:BA2814~BA697100~RFU:0 |
| Anthrax008~B04R16C02 | 3.01E-04 | Ba~MGC:BA1484~BA696453~RFU:44928.26 |
| Anthrax008~B01R02C17 | 2.97E-04 | Ba~MGC:BA3818~BA697579~RFU:0 |
| Anthrax008~B40R08C09 | 3.01E-04 | Ba~MGC:BA2148~BA14864~RFU:0 |
| Anthrax008~B46R02C18 | 2.93E-04 | Ba~MGC:BA3953~BA697653~RFU:54380.98 |
| Anthrax008~B17R08C09 | 2.96E-04 | Ba~MGC:BA1284~BA13456~RFU:23719.6 |
| Anthrax008~B48R01C18 | 3.02E-04 | Ba~MGC:BA1797~BA696613~RFU:1.43 |
| Anthrax008~B24R08C11 | 3.06E-04 | Ba~MGC:BA2718~BA14642~RFU:.92 |
| Anthrax008~B34R12C11 | 3.18E-04 | Ba~MGC:BA2804~BA13475~RFU:40688.01 |
| Anthrax008~B36R04C13 | 3.16E-04 | Ba~MGC:BA4398~BA697893~RFU:59904.34 |
| Anthrax008~B19R03C06 | 3.14E-04 | Ba~MGC:BA3390~BA697369~RFU:60044.15 |
| Anthrax008~B34R09C18 | 3.15E-04 | Ba~MGC:BA4394~BA15216~RFU:11074.39 |
| Anthrax008~B03R02C06 | 3.34E-04 | Ba~MGC:BA0836~BA696157~RFU:60077.26 |
| Anthrax008~B15R15C04 | 3.49E-04 | Ba~MGC:BA5255~BA698333~RFU:1571.01 |
| Anthrax008~B09R08C10 | 3.50E-04 | Ba~MGC:BA1035~BA14328~RFU:29076.53 |
| Anthrax008~B30R08C11 | 3.60E-04 | Ba~MGC:BA2626~BA14344~RFU:3.68 |
| Anthrax008~B21R12C15 | 3.66E-04 | Ba~MGC:BA2616~BA12661~RFU:19308.31 |
| Anthrax008~B40R02C04 | 3.63E-04 | Ba~MGC:BA2987~BA697183~RFU:4.29 |
| Anthrax008~B24R08C19 | 3.65E-04 | Ba~MGC:BA2217~BA15926~RFU:0 |
| Anthrax008~B31R02C14 | 3.58E-04 | Ba~MGC:BA2318~BA696876~RFU:60050.59 |
| Anthrax008~B34R12C06 | 3.63E-04 | Ba~MGC:BA2525~BA13449~RFU:3186.17 |
| Anthrax008~B40R04C17 | 3.70E-04 | Ba~MGC:BA4612~BA698002~RFU:59985.28 |
| Anthrax008~B29R12C19 | 3.75E-04 | Ba~MGC:BA3810~BA3810~RFU:2020.79 |
| Anthrax008~B11R15C04 | 3.82E-04 | Ba~MGC:BA2655~BA698784~RFU:60002.76 |
| Anthrax008~B17R05C07 | 3.82E-04 | Ba~MGC:BA1554~BA696488~RFU:59902.5 |
| Anthrax008~B03R15C06 | 3.88E-04 | Ba~MGC:BA5407~BA698413~RFU:59883.19 |
| Anthrax008~B47R02C19 | 3.87E-04 | Ba~MGC:BA4270~BA697814~RFU:60080.02 |
| Anthrax008~B11R08C20 | 3.91E-04 | Ba~MGC:BA1258~BA16082~RFU:1915.93 |
| Anthrax008~B11R08C19 | 4.08E-04 | Ba~MGC:BA1258~BA16082~RFU:1697.94 |
| Anthrax008~B37R02C08 | 4.09E-04 | Ba~MGC:BA0637~BA696060~RFU:60082.78 |
| Anthrax008~B01R02C15 | 4.04E-04 | Ba~MGC:BA4480~BA697934~RFU:0 |
| Anthrax008~B34R12C07 | 4.06E-04 | Ba~MGC:BA3659~BA3659~RFU:23880.61 |
| Anthrax008~B20R03C11 | 4.07E-04 | Ba~MGC:BA0143.1~BA695807~RFU:9072.96 |
| Anthrax008~B24R02C04 | 4.09E-04 | Ba~MGC:BA3580~BA697470~RFU:0 |
| Anthrax008~B47R02C20 | 4.09E-04 | Ba~MGC:BA4270~BA697814~RFU:60119.57 |
| Anthrax008~B19R08C16 | 4.12E-04 | Ba~MGC:BA0986~BA14300~RFU:20819.74 |
| Anthrax008~B09R08C17 | 4.47E-04 | Ba~MGC:BA0932~BA13718~RFU:8854.08 |
| Anthrax008~B09R08C18 | 4.46E-04 | Ba~MGC:BA0932~BA13718~RFU:9515.02 |
| Anthrax008~B34R12C14 | 4.47E-04 | Ba~MGC:BXB0075~BA14189~RFU:15527.9 |
| Anthrax008~B42R06C10 | 4.51E-04 | Ba~MGC:BA4372~BA697879~RFU:2666.67 |
| Anthrax008~B33R08C10 | 4.49E-04 | Ba~MGC:BA1292~BA13966~RFU:45517.88 |
| Anthrax008~B03R02C05 | 4.45E-04 | Ba~MGC:BA0836~BA696157~RFU:60080.94 |
| Anthrax008~B25R05C02 | 4.70E-04 | Ba~MGC:BA4501~BA697944~RFU:54045.78 |
| Anthrax008~B16R06C01 | 4.71E-04 | Ba~MGC:BA2800~BA697093~RFU:15036.79 |
| Anthrax008~B42R06C09 | 4.64E-04 | Ba~MGC:BA4372~BA697879~RFU:17110.16 |
| Anthrax008~B01R05C19 | 4.71E-04 | Ba~MGC:BA5054~BA698226~RFU:60123.25 |
| Anthrax008~B46R06C04 | 4.63E-04 | Ba~MGC:BA1418~BA696420~RFU:60046.91 |
| Anthrax008~B15R04C06 | 4.70E-04 | Ba~MGC:BA4750~BA698066~RFU:60090.14 |
| Anthrax008~B14R06C10 | 4.78E-04 | Ba~MGC:BA4429~BA697908~RFU:60084.62 |
| Anthrax008~B46R03C15 | 4.78E-04 | Ba~MGC:BA1143~BA696293~RFU:59986.2 |
| Anthrax008~B03R06C16 | 4.86E-04 | Ba~MGC:BA1482~BA696452~RFU:20244.67 |
| Anthrax008~B14R18C09 | 4.94E-04 | LF_12.5n~N/A |
| Anthrax008~B06R08C19 | 4.98E-04 | Ba~MGC:BA2311~BA13470~RFU:0 |
| Anthrax008~B40R04C18 | 4.97E-04 | Ba~MGC:BA4612~BA698002~RFU:60011.96 |
| Anthrax008~B44R08C17 | 5.02E-04 | Ba~MGC:BA0957~BA14220~RFU:32018.6 |
| Anthrax008~B45R06C11 | 5.17E-04 | Ba~MGC:BA3605~BA697480~RFU:59988.96 |
| Anthrax008~B15R05C20 | 5.12E-04 | Ba~MGC:BA3566~BA697461~RFU:60079.1 |
| Anthrax008~B34R09C19 | 5.16E-04 | Ba~MGC:BA4246~BA15458~RFU:2040.06 |
| Anthrax008~B32R12C11 | 5.15E-04 | Ba~MGC:BA2091~BA14006~RFU:20261.22 |
| Anthrax008~B19R03C05 | 5.14E-04 | Ba~MGC:BA3390~BA697369~RFU:60040.47 |
| Anthrax008~B20R03C12 | 5.16E-04 | Ba~MGC:BA0143.1~BA695807~RFU:60031.27 |
| Anthrax008~B32R02C10 | 5.22E-04 | Ba~MGC:BA2119~BA696770~RFU:1.43 |
| Anthrax008~B17R05C09 | 5.26E-04 | Ba~MGC:BA1349~BA696395~RFU:59900.66 |
| Anthrax008~B14R06C09 | 5.30E-04 | Ba~MGC:BA4429~BA697908~RFU:60070.82 |
| Anthrax008~B01R05C20 | 5.28E-04 | Ba~MGC:BA5054~BA698226~RFU:60116.81 |
| Anthrax008~B26R12C02 | 5.34E-04 | Ba~MGC:BA4677~BA16600~RFU:1209.53 |
| Anthrax008~B32R08C13 | 5.39E-04 | Ba~MGC:BA2738~BA16084~RFU:0 |
| Anthrax008~B21R15C04 | 5.41E-04 | Ba~MGC:BA3844~BA697595~RFU:2366.24 |
| Anthrax008~B48R08C10 | 5.47E-04 | Ba~MGC:BA2691~BA14402~RFU:7.15 |
| Anthrax008~B37R14C16 | 5.46E-04 | Ba~MGC:BA3036~BA13665~RFU:19659.51 |
| Anthrax008~B32R08C12 | 5.58E-04 | Ba~MGC:BA2541~BA15044~RFU:2547.93 |
| Anthrax008~B30R11C10 | 5.66E-04 | Ba~MGC:BA0042~BA12881~RFU:9092.16 |
| Anthrax008~B35R12C17 | 5.66E-04 | Ba~MGC:BA5442~BA5442~RFU:80.94 |
| Anthrax008~B38R02C07 | 5.69E-04 | Ba~MGC:BA2795~BA697090~RFU:0 |
| Anthrax008~B31R02C13 | 5.75E-04 | Ba~MGC:BA2318~BA696876~RFU:60072.66 |
| Anthrax008~B43R15C03 | 5.81E-04 | Ba~MGC:BA0958~BA14302~RFU:60127.85 |
| Anthrax008~B47R02C12 | 5.88E-04 | Ba~MGC:BA0688~BA696084~RFU:41575.11 |
| Anthrax008~B11R06C13 | 5.89E-04 | Ba~MGC:BA3105~BA697252~RFU:22154.51 |
| Anthrax008~B14R08C09 | 5.96E-04 | Ba~MGC:BA2562~BA15500~RFU:0 |
| Anthrax008~B10R03C14 | 6.04E-04 | Ba~MGC:BA0032~BA695744~RFU:60017.48 |
| Anthrax008~B46R04C07 | 6.12E-04 | Ba~MGC:BA3966~BA697663~RFU:60003.68 |
| Anthrax008~B33R12C12 | 6.31E-04 | Ba~MGC:BA0483~BA0483~RFU:21314.74 |
| Anthrax008~B45R06C12 | 6.36E-04 | Ba~MGC:BA3605~BA697480~RFU:60015.64 |
| Anthrax008~B24R02C05 | 6.49E-04 | Ba~MGC:BA3391~BA697370~RFU:0 |
| Anthrax008~B19R05C13 | 6.77E-04 | Ba~MGC:BA3819~BA697580~RFU:60058.87 |
| Anthrax008~B28R02C06 | 6.77E-04 | Ba~MGC:BA4121~BA697740~RFU:60056.11 |
| Anthrax008~B08R05C20 | 6.87E-04 | Ba~MGC:BA0030~BA695743~RFU:60084.62 |
| Anthrax008~B01R08C19 | 6.88E-04 | Ba~MGC:BA1122~BA13778~RFU:4638.52 |
| Anthrax008~B13R03C05 | 6.97E-04 | Ba~MGC:BA2884~BA697133~RFU:0 |
| Anthrax008~B35R08C11 | 7.05E-04 | Ba~MGC:BA1380~BA14512~RFU:213.16 |
| Anthrax008~B46R08C05 | 7.18E-04 | Ba~MGC:BA2389~BA15040~RFU:8.28 |
| Anthrax008~B35R15C08 | 7.25E-04 | Ba~MGC:BA2808~BA697095~RFU:24611.85 |
| Anthrax008~B09R05C03 | 7.28E-04 | Ba~MGC:BA3379~BA697365~RFU:0 |
| Anthrax008~B46R08C08 | 7.32E-04 | Ba~MGC:BA2187~BA14758~RFU:6.44 |
| Anthrax008~B33R09C20 | 7.35E-04 | Ba~MGC:BA3763~BA13228~RFU:5527.04 |
| Anthrax008~B14R08C11 | 7.43E-04 | Ba~MGC:BA2495~BA12808~RFU:0 |
| Anthrax008~B25R05C01 | 7.51E-04 | Ba~MGC:BA4501~BA697944~RFU:48716.74 |
| Anthrax008~B38R08C15 | 7.56E-04 | Ba~MGC:BA1623~BA14238~RFU:0 |
| Anthrax008~B09R05C04 | 7.64E-04 | Ba~MGC:BA3379~BA697365~RFU:59587.01 |
| Anthrax008~B30R08C09 | 7.66E-04 | Ba~MGC:BA2611~BA15182~RFU:0 |
| Anthrax008~B21R14C18 | 7.75E-04 | Ba~MGC:BA2358~BA696897~RFU:6370.49 |
| Anthrax008~B22R08C07 | 7.78E-04 | Ba~MGC:BA2353~BA15830~RFU:0 |
| Anthrax008~B18R11C03 | 7.74E-04 | Ba~MGC:BXA0006~BA15174~RFU:1947.2 |
| Anthrax008~B34R09C17 | 7.83E-04 | Ba~MGC:BA4394~BA15216~RFU:10878.4 |
| Anthrax008~B43R15C04 | 7.85E-04 | Ba~MGC:BA0958~BA14302~RFU:60142.57 |
| Anthrax008~B46R04C08 | 7.90E-04 | Ba~MGC:BA3966~BA697663~RFU:59984.36 |
| Anthrax008~B44R08C18 | 8.00E-04 | Ba~MGC:BA0957~BA14220~RFU:32220.31 |
| Anthrax008~B25R08C20 | 8.02E-04 | Ba~MGC:BA0973~BA13544~RFU:1552.61 |
| Anthrax008~B13R15C17 | 8.05E-04 | Ba~MGC:BA3114~BA697256~RFU:6816.59 |
| Anthrax008~B28R02C05 | 8.10E-04 | Ba~MGC:BA4121~BA697740~RFU:59957.69 |
| Anthrax008~B09R07C03 | 8.15E-04 | Ba~MGC:BA1073~BA696261~RFU:4496.87 |
| Anthrax008~B21R14C13 | 8.30E-04 | Ba~MGC:BA0114~BA13534~RFU:60113.13 |
| Anthrax008~B26R12C01 | 8.32E-04 | Ba~MGC:BA4677~BA16600~RFU:1204.01 |
| Anthrax008~B16R06C02 | 8.29E-04 | Ba~MGC:BA2800~BA697093~RFU:16905.58 |
| Anthrax008~B03R08C18 | 8.40E-04 | Ba~MGC:BA1406~BA14404~RFU:7791.13 |
| Anthrax008~B41R11C17 | 8.49E-04 | Ba~MGC:BA2041~BA17206~RFU:1869.81 |
| Anthrax008~B30R05C10 | 8.55E-04 | Ba~MGC:BA0120~BA695796~RFU:7712.47 |
| Anthrax008~B08R05C19 | 8.53E-04 | Ba~MGC:BA0030~BA695743~RFU:60048.75 |
| Anthrax008~B27R14C11 | 8.62E-04 | Ba~MGC:BA2439~BA696936~RFU:0 |
| Anthrax008~B36R05C15 | 8.63E-04 | Ba~MGC:BA0111~BA695792~RFU:59981.6 |
| Anthrax008~B34R12C08 | 8.73E-04 | Ba~MGC:BA3659~BA3659~RFU:25648.45 |
| Anthrax008~B32R08C17 | 8.82E-04 | Ba~MGC:BA2401~BA15434~RFU:0 |
| Anthrax008~B09R08C09 | 8.99E-04 | Ba~MGC:BA1035~BA14328~RFU:30194.08 |
| Anthrax008~B16R04C01 | 9.13E-04 | Ba~MGC:BA3795~BA697568~RFU:60089.22 |
| Anthrax008~B37R02C07 | 9.23E-04 | Ba~MGC:BA0637~BA696060~RFU:60070.82 |
| Anthrax008~B40R02C03 | 9.25E-04 | Ba~MGC:BA2987~BA697183~RFU:0 |
| Anthrax008~B21R16C15 | 9.33E-04 | Ba~MGC:BA1075~BA1075~RFU:9720.38 |
| Anthrax008~B14R05C04 | 9.41E-04 | Ba~MGC:BA3560~BA697458~RFU:54722.22 |
| Anthrax008~B28R03C04 | 9.40E-04 | Ba~MGC:BA3943~BA697648~RFU:57293.97 |
| Anthrax008~B36R05C16 | 9.50E-04 | Ba~MGC:BA0111~BA695792~RFU:59936.53 |
| Anthrax008~B09R07C04 | 9.54E-04 | Ba~MGC:BA1073~BA696261~RFU:4006.62 |
| Anthrax008~B30R05C09 | 9.65E-04 | Ba~MGC:BA0120~BA695796~RFU:59755.33 |
| Anthrax008~B43R03C02 | 9.71E-04 | Ba~MGC:BA0131~BA695801~RFU:59978.84 |
| Anthrax008~B19R08C13 | 9.79E-04 | Ba~MGC:BA1447~BA15140~RFU:4335.91 |
| Anthrax008~B13R07C04 | 9.91E-04 | Ba~MGC:BA4929~BA698169~RFU:60007.36 |
| Anthrax008~B30R08C15 | 9.88E-04 | Ba~MGC:BA2745~BA14364~RFU:7.36 |
| Anthrax008~B10R03C13 | 9.93E-04 | Ba~MGC:BA0032~BA695744~RFU:59962.29 |
| Anthrax008~B21R11C14 | 9.94E-04 | Ba~MGC:BA2196~BA16432~RFU:17327.61 |
| Anthrax008~B08R07C18 | 0.001011179 | Ba~MGC:BA0887~BA696183~RFU:2103.57 |
| Anthrax008~B38R11C08 | 0.001011442 | Ba~MGC:BXB0046~BA16018~RFU:7743.92 |
| Anthrax008~B48R08C17 | 0.00103559 | Ba~MGC:BA1788~BA14066~RFU:0 |
| Anthrax008~B01R08C14 | 0.001045534 | Ba~MGC:BA1444~BA13502~RFU:6623.44 |
| Anthrax008~B03R08C17 | 0.001065396 | Ba~MGC:BA1406~BA14404~RFU:7805.37 |
| Anthrax008~B40R12C01 | 0.001062505 | Ba~MGC:BA1175~BA13074~RFU:29784.77 |
| Anthrax008~B34R10C13 | 0.001063942 | Ba~MGC:BXB0096~BA14624~RFU:8107.3 |
| Anthrax008~B17R05C08 | 0.00107721 | Ba~MGC:BA1554~BA696488~RFU:59917.22 |
| Anthrax008~B09R08C13 | 0.001077035 | Ba~MGC:BA0814~BA13152~RFU:6248.93 |
| Anthrax008~B13R07C03 | 0.001076784 | Ba~MGC:BA4929~BA698169~RFU:60003.68 |
| Anthrax008~B47R04C18 | 0.001088864 | Ba~MGC:BA5076~BA698241~RFU:60071.74 |
| Anthrax008~B33R09C19 | 0.001092042 | Ba~MGC:BA3763~BA13228~RFU:7178.99 |
| Anthrax008~B19R05C14 | 0.00108462 | Ba~MGC:BA3819~BA697580~RFU:60069.9 |
| Anthrax008~B16R04C02 | 0.001086572 | Ba~MGC:BA3795~BA697568~RFU:60060.71 |
| Anthrax008~B06R02C05 | 0.001099141 | Ba~MGC:BA3358~BA697353~RFU:0 |
| Anthrax008~B11R06C16 | 0.0011012 | Ba~MGC:BA2354~BA696895~RFU:2833.89 |
| Anthrax008~B34R13C06 | 0.001149329 | Ba~MGC:BA2644~BA2644~RFU:4700.15 |
| Anthrax008~B42R04C14 | 0.001146842 | Ba~MGC:BA0733~BA696108~RFU:60058.87 |
| Anthrax008~B48R04C19 | 0.001151932 | Ba~MGC:BA2188~BA696803~RFU:29303.29 |
| Anthrax008~B43R03C01 | 0.001146493 | Ba~MGC:BA0131~BA695801~RFU:60051.51 |
| Anthrax008~B40R02C05 | 0.00116405 | Ba~MGC:BA1044~BA696245~RFU:2.76 |
| Anthrax008~B48R02C05 | 0.001161606 | Ba~MGC:BA0169~BA695824~RFU:0 |
| Anthrax008~B14R18C05 | 0.001173046 | LF_50n~N/A |
| Anthrax008~B22R14C14 | 0.001183631 | Ba~MGC:BA1434~BA15844~RFU:60102.1 |
| Anthrax008~B35R12C18 | 0.001182421 | Ba~MGC:BA5442~BA5442~RFU:90.14 |
| Anthrax008~B01R05C03 | 0.001181026 | Ba~MGC:BA2933~BA697154~RFU:60090.14 |
| Anthrax008~B10R04C01 | 0.001191296 | Ba~MGC:BA2856~BA697117~RFU:60004.6 |
| Anthrax008~B23R02C17 | 0.001207118 | Ba~MGC:BA0314~BA695900~RFU:60062.55 |
| Anthrax008~B45R03C03 | 0.001206202 | Ba~MGC:BA3990~BA697676~RFU:0 |
| Anthrax008~B14R08C07 | 0.001208815 | Ba~MGC:BA2555~BA14438~RFU:0 |
| Anthrax008~B08R08C13 | 0.001219377 | Ba~MGC:BA2454~BA15700~RFU:0 |
| Anthrax008~B14R18C07 | 0.001223595 | LF_25n~N/A |
| Anthrax008~B11R07C16 | 0.001244089 | Ba~MGC:BA0012~BA695730~RFU:21383.37 |
| Anthrax008~B44R08C20 | 0.001248906 | Ba~MGC:BA0881~BA16218~RFU:22632.33 |
| Anthrax008~B40R08C19 | 0.001251966 | Ba~MGC:BA2448~BA14908~RFU:0 |
| Anthrax008~B09R14C20 | 0.001258693 | Ba~MGC:BA4553~BA697972~RFU:30623.75 |
| Anthrax008~B43R02C08 | 0.001262572 | Ba~MGC:BA4529~BA697959~RFU:60090.14 |
| Anthrax008~B08R01C19 | 0.001270811 | Ba~MGC:BA5096~BA698252~RFU:0 |
| Anthrax008~B29R10C17 | 0.001316262 | Ba~MGC:BA5194~BA14092~RFU:2443.49 |
| Anthrax008~B11R06C15 | 0.001313871 | Ba~MGC:BA2354~BA696895~RFU:3828.18 |
| Anthrax008~B04R04C04 | 0.001297737 | Ba~MGC:BA2778~BA697080~RFU:59989.88 |
| Anthrax008~B13R06C03 | 0.001317846 | Ba~MGC:BA4035~BA697703~RFU:0 |
| Anthrax008~B23R02C18 | 0.001285392 | Ba~MGC:BA0314~BA695900~RFU:60057.95 |
| Anthrax008~B03R14C15 | 0.001311883 | Ba~MGC:BA0953~BA696215~RFU:59959.53 |
| Anthrax008~B33R16C17 | 0.001301249 | Ba~MGC:BA0509~BA0509~RFU:6341.92 |
| Anthrax008~B28R03C03 | 0.00131936 | Ba~MGC:BA3943~BA697648~RFU:52321.89 |
| Anthrax008~B40R04C02 | 0.001283455 | Ba~MGC:BA5214~BA698314~RFU:59964.13 |
| Anthrax008~B40R08C13 | 0.001306136 | Ba~MGC:BA1645~BA14486~RFU:0 |
| Anthrax008~B21R03C05 | 0.001288615 | Ba~MGC:BA3106~BA697253~RFU:2.86 |
| Anthrax008~B34R10C14 | 0.001353047 | Ba~MGC:BXB0096~BA14624~RFU:7580.83 |
| Anthrax008~B12R02C20 | 0.001350667 | Ba~MGC:BA0331~BA695912~RFU:60075.42 |
| Anthrax008~B15R04C05 | 0.001350914 | Ba~MGC:BA4750~BA698066~RFU:60111.3 |
| Anthrax008~B34R02C20 | 0.001338398 | Ba~MGC:BA0110~BA695791~RFU:60051.51 |
| Anthrax008~B22R18C16 | 0.001349761 | PA_1.5625n~N/A |
| Anthrax008~B38R11C07 | 0.001355618 | Ba~MGC:BXB0046~BA16018~RFU:9136.31 |
| Anthrax008~B40R04C01 | 0.001378746 | Ba~MGC:BA5214~BA698314~RFU:60031.27 |
| Anthrax008~B31R15C09 | 0.001375233 | Ba~MGC:BA1445.1~BA696434~RFU:10131.53 |
| Anthrax008~B12R05C07 | 0.001378109 | Ba~MGC:BA0644~BA696063~RFU:47228.9 |
| Anthrax008~B38R08C05 | 0.001372069 | Ba~MGC:BA2695~BA14318~RFU:0 |
| Anthrax008~B10R04C15 | 0.001383944 | Ba~MGC:BA4510~BA698819~RFU:59990.8 |
| Anthrax008~B35R10C16 | 0.001390654 | Ba~MGC:BXA0101~BA16174~RFU:963.02 |
| Anthrax008~B01R05C04 | 0.001401312 | Ba~MGC:BA2933~BA697154~RFU:42981.4 |
| Anthrax008~B35R08C12 | 0.001410821 | Ba~MGC:BA1380~BA14512~RFU:291.85 |
| Anthrax008~B10R03C18 | 0.001423533 | Ba~MGC:BA3608~BA697481~RFU:57984.73 |
| Anthrax008~B19R03C18 | 0.001438899 | Ba~MGC:BA3811~BA697575~RFU:60030.35 |
| Anthrax008~B06R02C07 | 0.00143686 | Ba~MGC:BA1048~BA696249~RFU:25.75 |
| Anthrax008~B24R01C17 | 0.001431206 | Ba~MGC:BA5458~BA698440~RFU:5.52 |
| Anthrax008~B03R14C16 | 0.001463369 | Ba~MGC:BA0953~BA696215~RFU:59990.8 |
| Anthrax008~B06R02C08 | 0.001460087 | Ba~MGC:BA1048~BA696249~RFU:12.88 |
| Anthrax008~B03R15C05 | 0.001486114 | Ba~MGC:BA5407~BA698413~RFU:59933.77 |
| Anthrax008~B46R14C13 | 0.001493669 | Ba~MGC:BXB0110~BA16022~RFU:57240.34 |
| Anthrax008~B42R04C13 | 0.001502237 | Ba~MGC:BA0733~BA696108~RFU:60073.58 |
| Anthrax008~B34R09C20 | 0.001506551 | Ba~MGC:BA4246~BA15458~RFU:2034.33 |
| Anthrax008~B02R14C14 | 0.001524222 | Ba~MGC:BA0494~BA695992~RFU:36798.28 |
| Anthrax008~B17R08C20 | 0.001525958 | Ba~MGC:BA1437~BA14262~RFU:845.29 |
| Anthrax008~B24R16C03 | 0.001547119 | Ba~MGC:BA0272~BA695876~RFU:28806.87 |
| Anthrax008~B24R02C07 | 0.001556088 | Ba~MGC:BA1311~BA696374~RFU:0 |
| Anthrax008~B11R07C15 | 0.001569689 | Ba~MGC:BA0012~BA695730~RFU:20316.41 |
| Anthrax008~B21R11C07 | 0.001667085 | Ba~MGC:BA5135~BA16026~RFU:4062.95 |
| Anthrax008~B19R08C14 | 0.001666695 | Ba~MGC:BA1447~BA15140~RFU:5582.23 |
| Anthrax008~B29R12C20 | 0.001660497 | Ba~MGC:BA3810~BA3810~RFU:1877.3 |
| Anthrax008~B13R15C18 | 0.001662346 | Ba~MGC:BA3114~BA697256~RFU:7277.41 |
| Anthrax008~B10R06C05 | 0.00166418 | Ba~MGC:BA4334~BA697854~RFU:59798.57 |
| Anthrax008~B30R08C19 | 0.001652717 | Ba~MGC:BA2402~BA14206~RFU:0 |
| Anthrax008~B36R04C14 | 0.001699159 | Ba~MGC:BA4398~BA697893~RFU:59893.3 |
| Anthrax008~B07R06C10 | 0.001709133 | Ba~MGC:BA4235~BA697796~RFU:45772.63 |
| Anthrax008~B27R08C08 | 0.001736375 | Ba~MGC:BA1366~BA15280~RFU:8425.31 |
| Anthrax008~B21R11C13 | 0.001733404 | Ba~MGC:BA2196~BA16432~RFU:16281.83 |
| Anthrax008~B46R08C07 | 0.001740843 | Ba~MGC:BA2187~BA14758~RFU:0 |
| Anthrax008~B46R05C11 | 0.001734937 | Ba~MGC:BA4477~BA697933~RFU:60006.44 |
| Anthrax008~B12R05C15 | 0.00176516 | Ba~MGC:BA3491~BA697419~RFU:60019.32 |
| Anthrax008~B07R06C09 | 0.001778479 | Ba~MGC:BA4235~BA697796~RFU:47299.48 |
| Anthrax008~B47R04C17 | 0.001775931 | Ba~MGC:BA5076~BA698241~RFU:60054.27 |
| Anthrax008~B08R08C07 | 0.001785375 | Ba~MGC:BA2276~BA14756~RFU:0 |
| Anthrax008~B37R05C02 | 0.001791533 | Ba~MGC:BA1969~BA696698~RFU:60053.35 |
| Anthrax008~B02R10C07 | 0.001856069 | Ba~MGC:BA4164~BA13424~RFU:10626.61 |
| Anthrax008~B19R06C09 | 0.001856219 | Ba~MGC:BA1216~BA696323~RFU:60073.58 |
| Anthrax008~B09R08C05 | 0.001862033 | Ba~MGC:BA0995~BA15772~RFU:7423.46 |
| Anthrax008~B35R03C17 | 0.001866538 | Ba~MGC:BA2459~BA696947~RFU:51541.57 |
| Anthrax008~B03R03C08 | 0.00187959 | Ba~MGC:BA1411~BA696418~RFU:60070.82 |
| Anthrax008~B14R05C03 | 0.001878682 | Ba~MGC:BA3560~BA697458~RFU:59675.31 |
| Anthrax008~B30R08C16 | 0.001885908 | Ba~MGC:BA2745~BA14364~RFU:0 |
| Anthrax008~B20R03C10 | 0.001893774 | Ba~MGC:BA3981~BA697674~RFU:4057.22 |
| Anthrax008~B21R15C03 | 0.00190552 | Ba~MGC:BA3844~BA697595~RFU:2498.16 |
| Anthrax008~B14R02C12 | 0.001902478 | Ba~MGC:BA1877~BA696654~RFU:0 |
| Anthrax008~B15R05C06 | 0.001915111 | Ba~MGC:BA5614~BA698509~RFU:55366.24 |
| Anthrax008~B46R08C09 | 0.001924553 | Ba~MGC:BA2668~BA16150~RFU:0 |
| Anthrax008~B39R12C17 | 0.001920368 | Ba~MGC:BA3522~BA13214~RFU:28101.55 |
| Anthrax008~B43R02C07 | 0.001933641 | Ba~MGC:BA4529~BA697959~RFU:60108.54 |
| Anthrax008~B09R14C13 | 0.001938763 | Ba~MGC:BA2986~BA15032~RFU:60067.14 |
| Anthrax008~B33R10C04 | 0.001949728 | Ba~MGC:BA3669~BA13592~RFU:29312.91 |
| Anthrax008~B01R06C15 | 0.001958999 | Ba~MGC:BA0246~BA695859~RFU:3115.34 |
| Anthrax008~B12R05C16 | 0.001969091 | Ba~MGC:BA3491~BA697419~RFU:60045.07 |
| Anthrax008~B45R05C05 | 0.001980705 | Ba~MGC:BA2021~BA696721~RFU:60049.67 |
| Anthrax008~B22R01C19 | 0.001980931 | Ba~MGC:BA3321~BA697332~RFU:0 |
| Anthrax008~B38R05C19 | 0.002002484 | Ba~MGC:BA0073~BA695767~RFU:60081.86 |
| Anthrax008~B35R15C13 | 0.002004818 | Ba~MGC:BA3849~BA16486~RFU:26422.92 |
| Anthrax008~B12R05C14 | 0.002018669 | Ba~MGC:BA0594~BA696039~RFU:60000 |
| Anthrax008~B12R05C08 | 0.00203394 | Ba~MGC:BA0644~BA696063~RFU:60058.87 |
| Anthrax008~B35R03C18 | 0.0020296 | Ba~MGC:BA2459~BA696947~RFU:51363.13 |
| Anthrax008~B35R16C13 | 0.002045414 | Ba~MGC:BA1474~BA1474~RFU:16942.6 |
| Anthrax008~B41R05C09 | 0.002064155 | Ba~MGC:BA0766~BA696124~RFU:0 |
| Anthrax008~B19R03C17 | 0.002070903 | Ba~MGC:BA3811~BA697575~RFU:60018.4 |
| Anthrax008~B43R02C18 | 0.002072753 | Ba~MGC:BA1861~BA696647~RFU:60081.86 |
| Anthrax008~B04R02C02 | 0.002093281 | Ba~MGC:BA0183~BA695833~RFU:60040.47 |
| Anthrax008~B45R05C06 | 0.002100171 | Ba~MGC:BA2021~BA696721~RFU:60045.07 |
| Anthrax008~B26R12C17 | 0.002094674 | Ba~MGC:BXB0112~BA13579~RFU:36387.97 |
| Anthrax008~B33R13C20 | 0.002093688 | Ba~MGC:BXA0005~BA698578~RFU:566.59 |
| Anthrax008~B22R08C17 | 0.002095475 | Ba~MGC:BA2519~BA14282~RFU:0 |
| Anthrax008~B13R05C20 | 0.002110037 | Ba~MGC:BA5551~BA698480~RFU:59901.58 |
| Anthrax008~B10R03C17 | 0.002131084 | Ba~MGC:BA3608~BA697481~RFU:51420.6 |
| Anthrax008~B08R14C09 | 0.002139073 | Ba~MGC:BA1869~BA13433~RFU:47793.41 |
| Anthrax008~B44R14C14 | 0.002155437 | Ba~MGC:BA3827~BA3827~RFU:2241.54 |
| Anthrax008~B34R02C19 | 0.002163312 | Ba~MGC:BA0110~BA695791~RFU:60056.11 |
| Anthrax008~B01R08C20 | 0.00218696 | Ba~MGC:BA1122~BA13778~RFU:5151.65 |
| Anthrax008~B09R03C10 | 0.002184836 | Ba~MGC:BA2066~BA696743~RFU:60097.5 |
| Anthrax008~B05R15C06 | 0.002217268 | Ba~MGC:BXA0061~BA698600~RFU:36938.93 |
| Anthrax008~B48R08C05 | 0.002218955 | Ba~MGC:BA2500~BA14596~RFU:6.44 |
| Anthrax008~B17R11C07 | 0.002242992 | Ba~MGC:BA5115~BA13562~RFU:28595.47 |
| Anthrax008~B32R02C08 | 0.002274904 | Ba~MGC:BA2386~BA698779~RFU:1.43 |
| Anthrax008~B22R07C19 | 0.002286128 | Ba~MGC:BA0871~BA696174~RFU:577.63 |
| Anthrax008~B40R13C01 | 0.002306882 | Ba~MGC:BA0609~BA16683~RFU:547.28 |
| Anthrax008~B01R15C08 | 0.002312861 | Ba~MGC:BA2129~BA696776~RFU:1243.56 |
| Anthrax008~B47R02C11 | 0.002312592 | Ba~MGC:BA0688~BA696084~RFU:41440.63 |
| Anthrax008~B36R06C16 | 0.002326356 | Ba~MGC:BA2591~BA698782~RFU:59971.49 |
| Anthrax008~B20R06C06 | 0.002327545 | Ba~MGC:BA4817~BA698102~RFU:60052.43 |
| Anthrax008~B06R06C08 | 0.002335293 | Ba~MGC:BA0224~BA695852~RFU:25363.38 |
| Anthrax008~B11R07C03 | 0.002366194 | Ba~MGC:BA1602~BA696518~RFU:21300.59 |
| Anthrax008~B03R08C14 | 0.002377654 | Ba~MGC:BA0947~BA13982~RFU:16959.16 |
| Anthrax008~B06R08C09 | 0.002378071 | Ba~MGC:BA2717~BA14920~RFU:0 |
| Anthrax008~B18R13C15 | 0.002394265 | Ba~MGC:BA1828~BA1828~RFU:21293.23 |
| Anthrax008~B29R02C05 | 0.002391408 | Ba~MGC:BA0857~BA696165~RFU:60097.5 |
| Anthrax008~B15R15C03 | 0.002412469 | Ba~MGC:BA5255~BA698333~RFU:1949.04 |
| Anthrax008~B38R01C18 | 0.002412751 | Ba~MGC:BA1569~BA696501~RFU:0 |
| Anthrax008~B14R13C15 | 0.002428411 | Ba~MGC:BA3136~BA3136~RFU:857.25 |
| Anthrax008~B30R02C08 | 0.002423744 | Ba~MGC:BA4850~BA698120~RFU:7.15 |
| Anthrax008~B12R05C13 | 0.002439546 | Ba~MGC:BA0594~BA696039~RFU:59916.3 |
| Anthrax008~B04R03C12 | 0.002453489 | Ba~MGC:BA3238~BA697300~RFU:60010.12 |
| Anthrax008~B35R15C03 | 0.002531783 | Ba~MGC:BA4840~BA14142~RFU:27525.75 |
| Anthrax008~B33R13C19 | 0.002536089 | Ba~MGC:BXA0005~BA698578~RFU:513.59 |
| Anthrax008~B29R12C12 | 0.00253606 | Ba~MGC:BA4711~BA4711~RFU:13929.9 |
| Anthrax008~B45R05C08 | 0.00253633 | Ba~MGC:BA4342~BA697862~RFU:41464.31 |
| Anthrax008~B02R03C05 | 0.00253911 | Ba~MGC:BA1733~BA696583~RFU:0 |
| Anthrax008~B22R08C06 | 0.002516156 | Ba~MGC:BA2375~BA15418~RFU:0 |
| Anthrax008~B10R06C06 | 0.002507015 | Ba~MGC:BA4334~BA697854~RFU:59923.66 |
| Anthrax008~B45R05C07 | 0.002499611 | Ba~MGC:BA4342~BA697862~RFU:44838.12 |
| Anthrax008~B01R06C16 | 0.00250653 | Ba~MGC:BA0246~BA695859~RFU:3460.26 |
| Anthrax008~B26R11C11 | 0.002559137 | Ba~MGC:BXA0011~BA17108~RFU:3519.31 |
| Anthrax008~B35R11C08 | 0.002567638 | Ba~MGC:BA5104~BA16116~RFU:2057.58 |
| Anthrax008~B29R10C18 | 0.002576601 | Ba~MGC:BA5194~BA14092~RFU:2419.17 |
| Anthrax008~B09R03C09 | 0.002585531 | Ba~MGC:BA2066~BA696743~RFU:60090.14 |
| Anthrax008~B39R10C15 | 0.002594144 | Ba~MGC:BA5086~BA15868~RFU:3803.35 |
| Anthrax008~B37R05C01 | 0.002613151 | Ba~MGC:BA1969~BA696698~RFU:60074.5 |
| Anthrax008~B03R03C07 | 0.002624898 | Ba~MGC:BA1411~BA696418~RFU:60064.39 |
| Anthrax008~B35R15C01 | 0.002631399 | Ba~MGC:BA5537~BA698474~RFU:57526.67 |
| Anthrax008~B04R02C01 | 0.002659399 | Ba~MGC:BA0183~BA695833~RFU:60056.11 |
| Anthrax008~B13R05C19 | 0.002655827 | Ba~MGC:BA5551~BA698480~RFU:59918.14 |
| Anthrax008~B22R01C20 | 0.002684107 | Ba~MGC:BA3321~BA697332~RFU:16.56 |
| Anthrax008~B44R04C05 | 0.002710585 | Ba~MGC:BA3339~BA697342~RFU:60005.52 |
| Anthrax008~B13R04C10 | 0.002723115 | Ba~MGC:BA2840~BA697109~RFU:43751.07 |
| Anthrax008~B05R12C18 | 0.002737074 | Ba~MGC:BA2660~BA13147~RFU:32860.56 |
| Anthrax008~B12R02C17 | 0.002754894 | Ba~MGC:BA0010~BA695728~RFU:59988.96 |
| Anthrax008~B42R14C10 | 0.002771825 | Ba~MGC:BA1313~BA696375~RFU:166.48 |
| Anthrax008~B40R13C16 | 0.002766796 | Ba~MGC:BA5245~BA5245~RFU:3056.48 |
| Anthrax008~B38R05C20 | 0.002813398 | Ba~MGC:BA0073~BA695767~RFU:60102.1 |
| Anthrax008~B08R14C10 | 0.002812983 | Ba~MGC:BA1869~BA13433~RFU:49749.82 |
| Anthrax008~B15R12C05 | 0.002789418 | Ba~MGC:BA3327~BA13237~RFU:22152.32 |
| Anthrax008~B22R13C15 | 0.002807721 | Ba~MGC:BA1784~BA1784~RFU:679.54 |
| Anthrax008~B17R11C08 | 0.002807186 | Ba~MGC:BA5115~BA13562~RFU:31201.25 |
| Anthrax008~B48R02C03 | 0.002797255 | Ba~MGC:BA0185~BA695835~RFU:0 |
| Anthrax008~B24R16C04 | 0.002825277 | Ba~MGC:BA0272~BA695876~RFU:32422.03 |
| Anthrax008~B06R03C19 | 0.002820521 | Ba~MGC:BA4674~BA698028~RFU:60022.08 |
| Anthrax008~B36R12C14 | 0.002847177 | Ba~MGC:BXB0002~BA13943~RFU:32771.34 |
| Anthrax008~B34R10C03 | 0.002846589 | Ba~MGC:BA4701~BA14946~RFU:2677.52 |
| Anthrax008~B29R12C11 | 0.002870482 | Ba~MGC:BA4711~BA4711~RFU:20902.72 |
| Anthrax008~B16R01C17 | 0.002878991 | Ba~MGC:BA3871~BA697606~RFU:0 |
| Anthrax008~B03R14C20 | 0.002876526 | Ba~MGC:BA5467~BA698444~RFU:25080.02 |
| Anthrax008~B40R05C12 | 0.002904441 | Ba~MGC:BA1144~BA696294~RFU:23055.79 |
| Anthrax008~B04R03C11 | 0.002975514 | Ba~MGC:BA3238~BA697300~RFU:60009.2 |
| Anthrax008~B02R10C08 | 0.002970895 | Ba~MGC:BA4164~BA13424~RFU:12176.23 |
| Anthrax008~B19R06C13 | 0.002999732 | Ba~MGC:BA0596~BA696041~RFU:706.72 |
| Anthrax008~B43R08C19 | 0.003041614 | Ba~MGC:BA1381~BA14660~RFU:7571.74 |
| Anthrax008~B10R04C02 | 0.003039003 | Ba~MGC:BA2856~BA697117~RFU:59950.33 |
| Anthrax008~B11R07C04 | 0.00308989 | Ba~MGC:BA1602~BA696518~RFU:20615.16 |
| Anthrax008~B44R10C04 | 0.003095368 | Ba~MGC:BA4268~BA13626~RFU:11748.53 |
| Anthrax008~B11R02C11 | 0.003106492 | Ba~MGC:BA2597~BA696997~RFU:1399.93 |
| Anthrax008~B40R02C06 | 0.003222529 | Ba~MGC:BA1044~BA696245~RFU:0 |
| Anthrax008~B24R08C12 | 0.003222987 | Ba~MGC:BA2718~BA14642~RFU:0 |
| Anthrax008~B14R13C16 | 0.003237565 | Ba~MGC:BA3136~BA3136~RFU:871.04 |
| Anthrax008~B11R13C08 | 0.003236662 | Ba~MGC:BA4191~BA14889~RFU:361.48 |
| Anthrax008~B20R06C05 | 0.003262616 | Ba~MGC:BA4817~BA698102~RFU:60056.11 |
| Anthrax008~B04R03C03 | 0.003255269 | Ba~MGC:BA2919~BA697146~RFU:0 |
| Anthrax008~B05R12C17 | 0.003264789 | Ba~MGC:BA2660~BA13147~RFU:43265.27 |
| Anthrax008~B06R03C20 | 0.003351162 | Ba~MGC:BA4674~BA698028~RFU:60034.03 |
| Anthrax008~B44R04C06 | 0.003369938 | Ba~MGC:BA3339~BA697342~RFU:59990.8 |
| Anthrax008~B20R03C09 | 0.003452117 | Ba~MGC:BA3981~BA697674~RFU:44579.4 |
| Anthrax008~B30R13C09 | 0.00349405 | Ba~MGC:BA4672~BA4672~RFU:421.27 |
| Anthrax008~B01R04C19 | 0.003484801 | Ba~MGC:BA3490~BA697418~RFU:53469.46 |
| Anthrax008~B38R02C20 | 0.003485798 | Ba~MGC:BA4410~BA697899~RFU:41204.58 |
| Anthrax008~B29R13C19 | 0.003500651 | Ba~MGC:BA4000~BA4000~RFU:810.34 |
| Anthrax008~B11R11C07 | 0.003505015 | Ba~MGC:BA5428~BA13268~RFU:22809.05 |
| Anthrax008~B26R12C18 | 0.003543144 | Ba~MGC:BXB0112~BA13579~RFU:35964.86 |
| Anthrax008~B15R05C05 | 0.003536283 | Ba~MGC:BA5614~BA698509~RFU:54459.23 |
| Anthrax008~B39R02C04 | 0.003587666 | Ba~MGC:BA0205~BA695845~RFU:60055.19 |
| Anthrax008~B02R04C15 | 0.003600123 | Ba~MGC:BA1170~BA696303~RFU:60090.14 |
| Anthrax008~B23R12C16 | 0.003627869 | Ba~MGC:BA3231~BA3231~RFU:6947.07 |
| Anthrax008~B06R06C07 | 0.00362557 | Ba~MGC:BA0224~BA695852~RFU:48825.46 |
| Anthrax008~B36R03C11 | 0.003650953 | Ba~MGC:BA0101~BA695788~RFU:53407.73 |
| Anthrax008~B32R02C07 | 0.003673479 | Ba~MGC:BA2386~BA698779~RFU:0 |
| Anthrax008~B40R04C10 | 0.003708437 | Ba~MGC:BA5102~BA698255~RFU:59925.5 |
| Anthrax008~B43R07C17 | 0.003723272 | Ba~MGC:BA2153~BA696787~RFU:2152.32 |
| Anthrax008~B06R06C02 | 0.003738256 | Ba~MGC:BA2412~BA696922~RFU:39087.27 |
| Anthrax008~B13R05C03 | 0.003734084 | Ba~MGC:BA0136~BA695803~RFU:60028.51 |
| Anthrax008~B39R02C03 | 0.003747946 | Ba~MGC:BA0205~BA695845~RFU:60036.79 |
| Anthrax008~B46R04C18 | 0.003755483 | Ba~MGC:BA1585~BA696509~RFU:60068.06 |
| Anthrax008~B01R15C07 | 0.003783922 | Ba~MGC:BA2129~BA696776~RFU:1078.92 |
| Anthrax008~B34R13C02 | 0.003801249 | Ba~MGC:BA3258~BA3258~RFU:4172.19 |
| Anthrax008~B31R10C15 | 0.003810671 | Ba~MGC:BA5137~BA13404~RFU:14716.7 |
| Anthrax008~B40R05C11 | 0.003831487 | Ba~MGC:BA1144~BA696294~RFU:59882.27 |
| Anthrax008~B40R13C15 | 0.003844591 | Ba~MGC:BA5245~BA5245~RFU:3054.64 |
| Anthrax008~B46R05C08 | 0.003851306 | Ba~MGC:BA3212~BA698801~RFU:60084.62 |
| Anthrax008~B01R14C19 | 0.003937871 | Ba~MGC:BA4440~BA697915~RFU:58899.01 |
| Anthrax008~B35R15C06 | 0.003985787 | Ba~MGC:BA5566~BA698487~RFU:34958.61 |
| Anthrax008~B16R12C04 | 0.004011071 | Ba~MGC:BA0512~BA0512~RFU:51947.2 |
| Anthrax008~B01R14C20 | 0.004034062 | Ba~MGC:BA4440~BA697915~RFU:60042.31 |
| Anthrax008~B38R08C19 | 0.004095373 | Ba~MGC:BA2172~BA14044~RFU:0 |
| Anthrax008~B11R10C01 | 0.004156338 | Ba~MGC:BA3951~BA13826~RFU:9915.59 |
| Anthrax008~B44R04C11 | 0.00422718 | Ba~MGC:BA4624~BA698008~RFU:57336.28 |
| Anthrax008~B19R07C08 | 0.004209491 | Ba~MGC:BA3915~BA697633~RFU:60053.35 |
| Anthrax008~B38R08C04 | 0.004221016 | Ba~MGC:BA0882~BA696181~RFU:1713.58 |
| Anthrax008~B01R04C20 | 0.004225955 | Ba~MGC:BA3490~BA697418~RFU:51710.82 |
| Anthrax008~B08R08C18 | 0.004237764 | Ba~MGC:BA2287~BA14950~RFU:4739.7 |
| Anthrax008~B17R05C03 | 0.004270429 | Ba~MGC:BA2053~BA696739~RFU:59744.3 |
| Anthrax008~B37R13C12 | 0.004282711 | Ba~MGC:BA1114~BA1114~RFU:1080.11 |
| Anthrax008~B33R05C19 | 0.004355948 | Ba~MGC:BA0296~BA695887~RFU:0 |
| Anthrax008~B09R14C17 | 0.004388588 | Ba~MGC:BA3474~BA698807~RFU:45449.21 |
| Anthrax008~B09R04C20 | 0.004432288 | Ba~MGC:BA3420~BA697385~RFU:33341.92 |
| Anthrax008~B48R15C10 | 0.004427858 | Ba~MGC:BA5179~BA698293~RFU:0 |
| Anthrax008~B14R18C03 | 0.004442348 | LF_100n~N/A |
| Anthrax008~B03R14C19 | 0.004450624 | Ba~MGC:BA5467~BA698444~RFU:29990.8 |
| Anthrax008~B39R10C19 | 0.004468667 | Ba~MGC:BA5227~BA12796~RFU:24222.77 |
| Anthrax008~B16R12C17 | 0.004485278 | Ba~MGC:BA0808~BA15883~RFU:17473.33 |
| Anthrax008~B34R13C05 | 0.004488158 | Ba~MGC:BA2644~BA2644~RFU:4941.13 |
| Anthrax008~B17R06C10 | 0.004518944 | Ba~MGC:BA0728~BA696104~RFU:55333.89 |
| Anthrax008~B41R03C05 | 0.004525032 | Ba~MGC:BA4815~BA698100~RFU:60049.67 |
| Anthrax008~B48R05C11 | 0.004539107 | Ba~MGC:BA0049~BA695754~RFU:60009.2 |
| Anthrax008~B46R06C02 | 0.004623286 | Ba~MGC:BA4394.1~BA697891~RFU:60037.71 |
| Anthrax008~B09R14C18 | 0.004708608 | Ba~MGC:BA3474~BA698807~RFU:43772.53 |
| Anthrax008~B07R03C20 | 0.004709004 | Ba~MGC:BA5648~BA698529~RFU:60111.3 |
| Anthrax008~B23R02C09 | 0.004755355 | Ba~MGC:BA1287~BA696366~RFU:34466.52 |
| Anthrax008~B13R05C16 | 0.004804494 | Ba~MGC:BA4537~BA697964~RFU:59097.68 |
| Anthrax008~B17R05C04 | 0.004876766 | Ba~MGC:BA2053~BA696739~RFU:59902.5 |
| Anthrax008~B31R14C11 | 0.004871578 | Ba~MGC:BA0231~BA17167~RFU:3989.15 |
| Anthrax008~B06R06C01 | 0.00494223 | Ba~MGC:BA2412~BA696922~RFU:32964.5 |
| Anthrax008~B35R07C08 | 0.004955438 | Ba~MGC:BA5120~BA698263~RFU:5694.44 |
| Anthrax008~B12R12C07 | 0.004987704 | Ba~MGC:BA3694~BA13523~RFU:56933.41 |
| Anthrax008~B39R02C16 | 0.005065673 | Ba~MGC:BA1563~BA696496~RFU:0 |
| Anthrax008~B35R10C15 | 0.005154527 | Ba~MGC:BXA0101~BA16174~RFU:1066.96 |
| Anthrax008~B29R04C14 | 0.005174632 | Ba~MGC:BA4797~BA698091~RFU:60063.47 |
| Anthrax008~B27R01C19 | 0.005222473 | Ba~MGC:BA4890~BA698144~RFU:60057.95 |
| Anthrax008~B31R10C16 | 0.005214896 | Ba~MGC:BA5137~BA13404~RFU:13550.4 |
| Anthrax008~B09R10C19 | 0.005236541 | Ba~MGC:BXA0044~BA15108~RFU:7217.45 |
| Anthrax008~B13R04C09 | 0.005261642 | Ba~MGC:BA2840~BA697109~RFU:59535.5 |
| Anthrax008~B29R10C15 | 0.005264923 | Ba~MGC:BA5144~BA14378~RFU:1456.37 |
| Anthrax008~B06R02C11 | 0.005279756 | Ba~MGC:BA3918~BA697634~RFU:0 |
| Anthrax008~B04R05C09 | 0.005302266 | Ba~MGC:BA5142~BA698274~RFU:59931.94 |
| Anthrax008~B09R04C19 | 0.00534848 | Ba~MGC:BA3420~BA697385~RFU:31703.86 |
| Anthrax008~B32R01C17 | 0.005406235 | Ba~MGC:BA3461~BA697403~RFU:15.64 |
| Anthrax008~B25R08C16 | 0.005414198 | Ba~MGC:BA1463~BA15132~RFU:9004.29 |
| Anthrax008~B02R10C11 | 0.005398068 | Ba~MGC:BA4500~BA15484~RFU:2075.97 |
| Anthrax008~B19R05C02 | 0.005459866 | Ba~MGC:BA0386~BA695946~RFU:60083.7 |
| Anthrax008~B41R03C06 | 0.005451345 | Ba~MGC:BA4815~BA698100~RFU:60030.35 |
| Anthrax008~B13R11C08 | 0.005476275 | Ba~MGC:BA5269~BA15278~RFU:1283.11 |
| Anthrax008~B34R12C10 | 0.005506606 | Ba~MGC:BA4649~BA13315~RFU:21108.73 |
| Anthrax008~B40R05C17 | 0.005574236 | Ba~MGC:BA0125~BA695797~RFU:60022.99 |
| Anthrax008~B40R09C20 | 0.005575897 | Ba~MGC:BA5086~BA15868~RFU:3811.63 |
| Anthrax008~B12R02C19 | 0.005576686 | Ba~MGC:BA0331~BA695912~RFU:59991.72 |
| Anthrax008~B45R11C05 | 0.005578426 | Ba~MGC:BA4348~BA14478~RFU:7379.51 |
| Anthrax008~B19R07C06 | 0.005588058 | Ba~MGC:BA3076~BA697235~RFU:60086.46 |
| Anthrax008~B39R06C08 | 0.005633438 | Ba~MGC:BA1195~BA696314~RFU:60104.86 |
| Anthrax008~B08R03C18 | 0.00565351 | Ba~MGC:BA2363~BA696898~RFU:60037.71 |
| Anthrax008~B41R02C10 | 0.005645246 | Ba~MGC:BA4759~BA698073~RFU:60005.52 |
| Anthrax008~B18R03C13 | 0.005669165 | Ba~MGC:BA4236~BA697797~RFU:60028.51 |
| Anthrax008~B40R08C04 | 0.005672794 | Ba~MGC:BA3859~BA697600~RFU:1862.58 |
| Anthrax008~B40R05C18 | 0.005718071 | Ba~MGC:BA0125~BA695797~RFU:60043.23 |
| Anthrax008~B34R06C09 | 0.005784508 | Ba~MGC:BA4650~BA698017~RFU:45762.51 |
| Anthrax008~B04R05C10 | 0.005796592 | Ba~MGC:BA5142~BA698274~RFU:59982.52 |
| Anthrax008~B33R05C20 | 0.00589647 | Ba~MGC:BA0296~BA695887~RFU:.92 |
| Anthrax008~B07R03C19 | 0.005900234 | Ba~MGC:BA5648~BA698529~RFU:60090.14 |
| Anthrax008~B36R03C12 | 0.005943086 | Ba~MGC:BA0101~BA695788~RFU:17557.94 |
| Anthrax008~B19R07C07 | 0.006008634 | Ba~MGC:BA3915~BA697633~RFU:60052.43 |
| Anthrax008~B12R02C18 | 0.0060011 | Ba~MGC:BA0010~BA695728~RFU:59961.37 |
| Anthrax008~B35R15C19 | 0.006050407 | Ba~MGC:BA3893~BA15359~RFU:10336.64 |
| Anthrax008~B27R02C04 | 0.006088565 | Ba~MGC:BA0060~BA695759~RFU:6933.41 |
| Anthrax008~B02R08C13 | 0.006085572 | Ba~MGC:BA1729~BA13066~RFU:29492.27 |
| Anthrax008~B41R06C05 | 0.006075995 | Ba~MGC:BA0420~BA695963~RFU:54978.54 |
| Anthrax008~B26R11C05 | 0.006116194 | Ba~MGC:BA5712~BA15596~RFU:4752.58 |
| Anthrax008~B24R15C11 | 0.006170519 | Ba~MGC:BA0774~BA17122~RFU:611.66 |
| Anthrax008~B30R01C18 | 0.006189297 | Ba~MGC:BA0216~BA695848~RFU:27.59 |
| Anthrax008~B19R07C05 | 0.006209084 | Ba~MGC:BA3076~BA697235~RFU:60095.66 |
| Anthrax008~B27R02C03 | 0.006268402 | Ba~MGC:BA0060~BA695759~RFU:57568.98 |
| Anthrax008~B30R08C12 | 0.00629803 | Ba~MGC:BA2626~BA14344~RFU:0 |
| Anthrax008~B41R03C16 | 0.006333324 | Ba~MGC:BA1573~BA696503~RFU:59964.13 |
| Anthrax008~B41R02C09 | 0.006385423 | Ba~MGC:BA4759~BA698073~RFU:60048.75 |
| Anthrax008~B35R15C07 | 0.006426378 | Ba~MGC:BA2808~BA697095~RFU:23794.15 |
| Anthrax008~B11R04C13 | 0.006534512 | Ba~MGC:BA0038.1~BA695747~RFU:60038.63 |
| Anthrax008~B41R03C15 | 0.00657465 | Ba~MGC:BA1573~BA696503~RFU:59946.65 |
| Anthrax008~B10R04C08 | 0.006572524 | Ba~MGC:BA4014~BA697691~RFU:7282.01 |
| Anthrax008~B13R11C07 | 0.006593708 | Ba~MGC:BA5269~BA15278~RFU:1225.17 |
| Anthrax008~B04R03C14 | 0.006608144 | Ba~MGC:BA2227~BA696825~RFU:60054.27 |
| Anthrax008~B48R11C03 | 0.006703781 | Ba~MGC:BXB0093~BA14790~RFU:5978.54 |
| Anthrax008~B04R03C13 | 0.006701101 | Ba~MGC:BA2227~BA696825~RFU:60025.75 |
| Anthrax008~B34R06C19 | 0.006722247 | Ba~MGC:BA3422~BA697387~RFU:28450.15 |
| Anthrax008~B29R10C16 | 0.006726698 | Ba~MGC:BA5144~BA14378~RFU:1198.86 |
| Anthrax008~B08R08C19 | 0.006739724 | Ba~MGC:BA2342~BA14170~RFU:24840.88 |
| Anthrax008~B48R13C14 | 0.006756596 | Ba~MGC:BA4933~BA4933~RFU:1121.6 |
| Anthrax008~B36R06C15 | 0.006819393 | Ba~MGC:BA2591~BA698782~RFU:59977.01 |
| Anthrax008~B09R05C16 | 0.006813165 | Ba~MGC:BA2945~BA697162~RFU:59958.61 |
| Anthrax008~B48R02C09 | 0.006813935 | Ba~MGC:BA5002~BA698203~RFU:0 |
| Anthrax008~B41R05C03 | 0.006865163 | Ba~MGC:BA4256~BA697807~RFU:0 |
| Anthrax008~B28R10C04 | 0.006858992 | Ba~MGC:BA4310~BA14784~RFU:2983.81 |
| Anthrax008~B16R01C20 | 0.006939853 | Ba~MGC:BA2960~BA697172~RFU:7.36 |
| Anthrax008~B22R01C18 | 0.006928705 | Ba~MGC:BA4774~BA698082~RFU:4.6 |
| Anthrax008~B12R12C06 | 0.006934844 | Ba~MGC:BA3589~BA13916~RFU:60091.06 |
| Anthrax008~B48R15C05 | 0.006976125 | Ba~MGC:BA1712~BA696568~RFU:1146.06 |
| Anthrax008~B14R18C01 | 0.007051143 | LF_200n~N/A |
| Anthrax008~B15R01C17 | 0.007120448 | Ba~MGC:BA5355~BA698384~RFU:46489.27 |
| Anthrax008~B48R13C10 | 0.007114652 | Ba~MGC:BA5327~BA15265~RFU:241.77 |
| Anthrax008~B15R12C08 | 0.007153201 | Ba~MGC:BA4392~BA4392~RFU:36179.18 |
| Anthrax008~B39R06C19 | 0.007191129 | Ba~MGC:BA1707~BA696567~RFU:0 |
| Anthrax008~B29R04C13 | 0.007185864 | Ba~MGC:BA4797~BA698091~RFU:60047.83 |
| Anthrax008~B37R03C02 | 0.007278794 | Ba~MGC:BA3303~BA697325~RFU:35273.25 |
| Anthrax008~B18R10C07 | 0.007294468 | Ba~MGC:BA4437~BA13780~RFU:6479.03 |
| Anthrax008~B12R12C05 | 0.007333547 | Ba~MGC:BA3589~BA13916~RFU:60082.78 |
| Anthrax008~B17R14C09 | 0.007398373 | Ba~MGC:BA4681~BA698031~RFU:6215.05 |
| Anthrax008~B24R06C09 | 0.007454983 | Ba~MGC:BA4593~BA697989~RFU:60070.82 |
| Anthrax008~B48R09C20 | 0.007468057 | Ba~MGC:BA5287~BA14606~RFU:1370.53 |
| Anthrax008~B30R13C10 | 0.007539292 | Ba~MGC:BA4672~BA4672~RFU:403.79 |
| Anthrax008~B13R05C15 | 0.007564219 | Ba~MGC:BA4537~BA697964~RFU:58757.36 |
| Anthrax008~B31R13C06 | 0.007624525 | Ba~MGC:BA4768~BA14360~RFU:32960.82 |
| Anthrax008~B44R09C20 | 0.007618603 | Ba~MGC:BA4514~BA14256~RFU:2855.51 |
| Anthrax008~B39R06C07 | 0.007665818 | Ba~MGC:BA1195~BA696314~RFU:60093.82 |
| Anthrax008~B45R03C16 | 0.007679645 | Ba~MGC:BA3713~BA697527~RFU:10553.65 |
| Anthrax008~B43R05C15 | 0.007735203 | Ba~MGC:BA1370~BA696402~RFU:60037.71 |
| Anthrax008~B43R05C16 | 0.00787001 | Ba~MGC:BA1370~BA696402~RFU:60046.91 |
| Anthrax008~B34R05C09 | 0.007863192 | Ba~MGC:BA2236~BA696832~RFU:60091.98 |
| Anthrax008~B23R02C10 | 0.007852813 | Ba~MGC:BA1287~BA696366~RFU:37940.58 |
| Anthrax008~B46R08C11 | 0.007922605 | Ba~MGC:BA2693~BA14168~RFU:0 |
| Anthrax008~B46R04C01 | 0.007930984 | Ba~MGC:BA2031~BA696728~RFU:59977.01 |
| Anthrax008~B24R06C10 | 0.007954494 | Ba~MGC:BA4593~BA697989~RFU:60067.14 |
| Anthrax008~B34R06C10 | 0.007983905 | Ba~MGC:BA4650~BA698017~RFU:47959.9 |
| Anthrax008~B09R05C15 | 0.008142537 | Ba~MGC:BA2945~BA697162~RFU:60024.83 |
| Anthrax008~B34R02C01 | 0.008175082 | Ba~MGC:BA1429~BA696429~RFU:60096.58 |
| Anthrax008~B38R02C19 | 0.008209291 | Ba~MGC:BA4410~BA697899~RFU:37918.45 |
| Anthrax008~B34R06C20 | 0.008222604 | Ba~MGC:BA3422~BA697387~RFU:32632.45 |
| Anthrax008~B11R04C14 | 0.008259825 | Ba~MGC:BA0038.1~BA695747~RFU:60033.11 |
| Anthrax008~B11R04C01 | 0.008385656 | Ba~MGC:BA3761~BA697553~RFU:60024.83 |
| Anthrax008~B30R04C17 | 0.008473558 | Ba~MGC:BA2458~BA696946~RFU:60032.19 |
| Anthrax008~B34R02C02 | 0.008506717 | Ba~MGC:BA1429~BA696429~RFU:60107.62 |
| Anthrax008~B30R04C18 | 0.008559359 | Ba~MGC:BA2458~BA696946~RFU:60020.24 |
| Anthrax008~B23R15C11 | 0.008572882 | Ba~MGC:BA5651~BA15670~RFU:3124.54 |
| Anthrax008~B14R11C18 | 0.008638538 | Ba~MGC:BA0906~BA0906~RFU:11519.31 |
| Anthrax008~B24R15C13 | 0.008687075 | Ba~MGC:BA1420~BA16549~RFU:1005.33 |
| Anthrax008~B42R05C10 | 0.008703903 | Ba~MGC:BA1244~BA696342~RFU:34291.76 |
| Anthrax008~B39R06C20 | 0.008719452 | Ba~MGC:BA1707~BA696567~RFU:60075.42 |
| Anthrax008~B48R13C09 | 0.008750785 | Ba~MGC:BA5327~BA15265~RFU:250.36 |
| Anthrax008~B04R02C08 | 0.008837215 | Ba~MGC:BA2446~BA696939~RFU:59982.52 |
| Anthrax008~B35R03C05 | 0.008849533 | Ba~MGC:BA1994~BA696707~RFU:60029.43 |
| Anthrax008~B24R08C01 | 0.008983457 | Ba~MGC:BA5395~BA698404~RFU:3032.9 |
| Anthrax008~B28R02C12 | 0.009010113 | Ba~MGC:BA3336~BA697340~RFU:60019.32 |
| Anthrax008~B47R07C06 | 0.009013331 | Ba~MGC:BA3537~BA697446~RFU:60133.37 |
| Anthrax008~B02R03C14 | 0.009058555 | Ba~MGC:BA3504~BA697427~RFU:35928.99 |
| Anthrax008~B18R10C15 | 0.009097679 | Ba~MGC:BXB0074~BA15994~RFU:5473.53 |
| Anthrax008~B35R14C19 | 0.009085429 | Ba~MGC:BA2088~BA696753~RFU:60044.15 |
| Anthrax008~B38R05C03 | 0.009100114 | Ba~MGC:BA0448~BA695977~RFU:0 |
| Anthrax008~B40R12C02 | 0.00922723 | Ba~MGC:BA1175~BA13074~RFU:33841.98 |
| Anthrax008~B30R10C10 | 0.009250724 | Ba~MGC:BA4615~BA13702~RFU:0 |
| Anthrax008~B05R02C12 | 0.009277382 | Ba~MGC:BA0127~BA695799~RFU:50786.42 |
| Anthrax008~B03R05C04 | 0.009273155 | Ba~MGC:BA5141~BA698273~RFU:60067.14 |
| Anthrax008~B28R03C08 | 0.009474958 | Ba~MGC:BA3813~BA697576~RFU:59972.41 |
| Anthrax008~B30R07C11 | 0.009596068 | Ba~MGC:BA0080~BA695773~RFU:2510.12 |
| Anthrax008~B15R03C07 | 0.009616436 | Ba~MGC:BA3167~BA697271~RFU:0 |
| Anthrax008~B19R05C01 | 0.009688562 | Ba~MGC:BA0386~BA695946~RFU:60071.74 |
| Anthrax008~B38R04C04 | 0.009675922 | Ba~MGC:BA4963~BA698183~RFU:22895.57 |
| Anthrax008~B24R04C12 | 0.009663382 | Ba~MGC:BA4162~BA697762~RFU:59972.41 |
| Anthrax008~B05R05C12 | 0.009727884 | Ba~MGC:BA1672~BA696552~RFU:58077.63 |
| Anthrax008~B31R10C18 | 0.009718542 | Ba~MGC:BA5208~BA13390~RFU:4523.55 |
| Anthrax008~B48R11C19 | 0.009762101 | Ba~MGC:BA1644~BA12445~RFU:13971.39 |
| Anthrax008~B39R12C18 | 0.009803086 | Ba~MGC:BA3522~BA13214~RFU:25054.36 |
| Anthrax008~B12R14C08 | 0.009809166 | Ba~MGC:BA4091~BA697729~RFU:336.64 |
| Anthrax008~B48R10C04 | 0.009851879 | Ba~MGC:BA5228~BA14098~RFU:1417.74 |
| Anthrax008~B47R07C05 | 0.00990545 | Ba~MGC:BA3537~BA697446~RFU:60128.77 |
| Anthrax008~B35R03C06 | 0.009953404 | Ba~MGC:BA1994~BA696707~RFU:60004.6 |
| Anthrax008~B18R10C11 | 0.009995593 | Ba~MGC:BA4420~BA13552~RFU:6812.91 |
| Anthrax008~B47R01C17 | 0.010000781 | Ba~MGC:BA4023~BA697696~RFU:31791.13 |
| Anthrax008~B26R05C11 | 0.010029202 | Ba~MGC:BA1629~BA696530~RFU:60098.42 |
| Anthrax008~B32R13C12 | 0.01005747 | Ba~MGC:BA4399~BA4399~RFU:635.58 |
| Anthrax008~B16R12C18 | 0.010214135 | Ba~MGC:BA0808~BA15883~RFU:18887.97 |
| Anthrax008~B16R08C05 | 0.010303021 | Ba~MGC:BA2532~BA13688~RFU:0 |
| Anthrax008~B08R14C06 | 0.010450903 | Ba~MGC:BXB0014~BA15568~RFU:47525.04 |
| Anthrax008~B48R10C03 | 0.010447752 | Ba~MGC:BA5228~BA14098~RFU:1227.01 |
| Anthrax008~B27R01C20 | 0.010440626 | Ba~MGC:BA4890~BA698144~RFU:60068.06 |
| Anthrax008~B33R02C18 | 0.010517001 | Ba~MGC:BA4562~BA697977~RFU:60089.22 |
| Anthrax008~B33R04C08 | 0.010603447 | Ba~MGC:BA0188~BA695838~RFU:59913.54 |
| Anthrax008~B19R05C17 | 0.010638337 | Ba~MGC:BA5081~BA698243~RFU:60068.98 |
| Anthrax008~B28R11C06 | 0.010667414 | Ba~MGC:BA5721~BA13676~RFU:7098.97 |
| Anthrax008~B48R05C02 | 0.010689238 | Ba~MGC:BA4798~BA698092~RFU:60010.12 |
| Anthrax008~B08R02C02 | 0.01077733 | Ba~MGC:BA2383~BA696907~RFU:0 |
| Anthrax008~B14R14C02 | 0.010770557 | Ba~MGC:BA2769~BA14444~RFU:60093.82 |
| Anthrax008~B07R02C02 | 0.010729735 | Ba~MGC:BA5195~BA698304~RFU:22632.45 |
| Anthrax008~B28R02C11 | 0.010773947 | Ba~MGC:BA3336~BA697340~RFU:59982.52 |
| Anthrax008~B16R14C15 | 0.010761607 | Ba~MGC:BA2529~BA698781~RFU:59528.15 |
| Anthrax008~B01R14C14 | 0.010813751 | Ba~MGC:BA4424~BA14350~RFU:54093.08 |
| Anthrax008~B02R03C13 | 0.010866883 | Ba~MGC:BA3504~BA697427~RFU:39208.98 |
| Anthrax008~B30R07C12 | 0.010947033 | Ba~MGC:BA0080~BA695773~RFU:2485.28 |
| Anthrax008~B36R05C20 | 0.010948435 | Ba~MGC:BA2145~BA696784~RFU:60045.07 |
| Anthrax008~B11R04C02 | 0.01101379 | Ba~MGC:BA3761~BA697553~RFU:60008.28 |
| Anthrax008~B33R02C17 | 0.011024226 | Ba~MGC:BA4562~BA697977~RFU:60066.23 |
| Anthrax008~B20R10C09 | 0.011042282 | Ba~MGC:BA4373~BA15668~RFU:805.44 |
| Anthrax008~B45R07C09 | 0.011060641 | Ba~MGC:BA4309~BA697836~RFU:862.77 |
| Anthrax008~B32R02C04 | 0.011122973 | Ba~MGC:BA2114~BA696768~RFU:11.96 |
| Anthrax008~B05R02C11 | 0.011234907 | Ba~MGC:BA0127~BA695799~RFU:57351.91 |
| Anthrax008~B40R02C20 | 0.011278737 | Ba~MGC:BA4608~BA697998~RFU:60021.16 |
| Anthrax008~B47R06C15 | 0.011264096 | Ba~MGC:BA3513~BA697432~RFU:60108.54 |
| Anthrax008~B46R05C09 | 0.011381558 | Ba~MGC:BA2207~BA696814~RFU:60071.74 |
| Anthrax008~B48R05C01 | 0.011367278 | Ba~MGC:BA4798~BA698092~RFU:60098.42 |
| Anthrax008~B48R07C18 | 0.011584505 | Ba~MGC:BA3373~BA697364~RFU:1482.12 |
| Anthrax008~B01R07C04 | 0.011675942 | Ba~MGC:BA5248~BA698328~RFU:60114.05 |
| Anthrax008~B14R03C14 | 0.011739009 | Ba~MGC:BA0259~BA695868~RFU:60005.52 |
| Anthrax008~B14R06C05 | 0.011795743 | Ba~MGC:BA1949~BA696689~RFU:59977.01 |
| Anthrax008~B28R11C12 | 0.011821052 | Ba~MGC:BXA0035~BA17214~RFU:8199.04 |
| Anthrax008~B35R13C19 | 0.011898504 | Ba~MGC:BA0230~BA0230~RFU:59958.61 |
| Anthrax008~B25R01C17 | 0.011951892 | Ba~MGC:BA0834~BA696155~RFU:4139.07 |
| Anthrax008~B26R11C12 | 0.011976875 | Ba~MGC:BXA0011~BA17108~RFU:2858.37 |
| Anthrax008~B04R02C07 | 0.012058473 | Ba~MGC:BA2446~BA696939~RFU:60000 |
| Anthrax008~B33R07C07 | 0.01216048 | Ba~MGC:BA5693~BA698551~RFU:27377.67 |
| Anthrax008~B38R04C03 | 0.012232676 | Ba~MGC:BA4963~BA698183~RFU:59862.03 |
| Anthrax008~B47R06C16 | 0.012246322 | Ba~MGC:BA3513~BA697432~RFU:60111.3 |
| Anthrax008~B08R07C13 | 0.012259798 | Ba~MGC:BA0107~BA695790~RFU:0 |
| Anthrax008~B39R07C12 | 0.012332029 | Ba~MGC:BA5367~BA698392~RFU:865.53 |
| Anthrax008~B13R06C14 | 0.012384466 | Ba~MGC:BA5527~BA698827~RFU:44553.65 |
| Anthrax008~B15R01C18 | 0.012377403 | Ba~MGC:BA5355~BA698384~RFU:41705.29 |
| Anthrax008~B07R02C01 | 0.012356824 | Ba~MGC:BA5195~BA698304~RFU:22192.79 |
| Anthrax008~B01R14C13 | 0.012478609 | Ba~MGC:BA4424~BA14350~RFU:60088.3 |
| Anthrax008~B48R09C19 | 0.012553345 | Ba~MGC:BA5287~BA14606~RFU:1864.42 |
| Anthrax008~B48R07C17 | 0.012578905 | Ba~MGC:BA3373~BA697364~RFU:1537.9 |
| Anthrax008~B02R07C05 | 0.012667482 | Ba~MGC:BA2175~BA696798~RFU:33755.52 |
| Anthrax008~B12R10C14 | 0.012745124 | Ba~MGC:BXA0188~BA13750~RFU:7405.26 |
| Anthrax008~B06R05C03 | 0.012818597 | Ba~MGC:BA5318~BA698363~RFU:46396.25 |
| Anthrax008~B15R04C02 | 0.012873126 | Ba~MGC:BA1275~BA696360~RFU:49965.67 |
| Anthrax008~B39R07C11 | 0.012931898 | Ba~MGC:BA5367~BA698392~RFU:1015.45 |
| Anthrax008~B44R10C10 | 0.012957031 | Ba~MGC:BA4073~BA14818~RFU:35279.62 |
| Anthrax008~B34R05C10 | 0.013101162 | Ba~MGC:BA2236~BA696832~RFU:60093.82 |
| Anthrax008~B10R04C07 | 0.013091773 | Ba~MGC:BA4014~BA697691~RFU:7782.38 |
| Anthrax008~B39R04C09 | 0.013278771 | Ba~MGC:BA1462~BA696444~RFU:60058.87 |
| Anthrax008~B40R02C19 | 0.013352162 | Ba~MGC:BA4608~BA697998~RFU:59971.49 |
| Anthrax008~B13R06C13 | 0.013380903 | Ba~MGC:BA5527~BA698827~RFU:43010.01 |
| Anthrax008~B26R05C12 | 0.013393131 | Ba~MGC:BA1629~BA696530~RFU:44928.47 |
| Anthrax008~B38R04C09 | 0.013450132 | Ba~MGC:BA3620~BA697486~RFU:2110.01 |
| Anthrax008~B40R02C11 | 0.013442333 | Ba~MGC:BA4338~BA697858~RFU:0 |
| Anthrax008~B02R07C06 | 0.013468186 | Ba~MGC:BA2175~BA696798~RFU:28119.94 |
| Anthrax008~B34R02C05 | 0.013501208 | Ba~MGC:BA0067~BA695763~RFU:34237.49 |
| Anthrax008~B03R04C20 | 0.013576872 | Ba~MGC:BA0017~BA695735~RFU:60016.56 |
| Anthrax008~B28R10C08 | 0.013635348 | Ba~MGC:BA3487~BA15100~RFU:10675.13 |
| Anthrax008~B35R03C19 | 0.013700764 | Ba~MGC:BA3764~BA697554~RFU:59870.31 |
| Anthrax008~B46R04C02 | 0.013902 | Ba~MGC:BA2031~BA696728~RFU:59933.77 |
| Anthrax008~B40R01C19 | 0.013988948 | Ba~MGC:BA1502~BA696462~RFU:11.44 |
| Anthrax008~B30R02C07 | 0.014034094 | Ba~MGC:BA4850~BA698120~RFU:0 |
| Anthrax008~B15R10C20 | 0.014148283 | Ba~MGC:BA5056~BA15666~RFU:958.51 |
| Anthrax008~B35R03C20 | 0.014148274 | Ba~MGC:BA3764~BA697554~RFU:59898.82 |
| Anthrax008~B39R02C19 | 0.014269007 | Ba~MGC:BA1932~BA696681~RFU:60027.59 |
| Anthrax008~B34R12C05 | 0.014247401 | Ba~MGC:BA2525~BA13449~RFU:2987.49 |
| Anthrax008~B18R13C18 | 0.014257049 | Ba~MGC:BA3905~BA3905~RFU:60124.17 |
| Anthrax008~B13R05C17 | 0.014489918 | Ba~MGC:BA2038.1~BA696732~RFU:59818.8 |
| Anthrax008~B14R03C10 | 0.014526038 | Ba~MGC:BA2434~BA696932~RFU:29215.42 |
| Anthrax008~B25R11C05 | 0.014552746 | Ba~MGC:BA5069~BA14834~RFU:2000 |
| Anthrax008~B21R03C06 | 0.014675865 | Ba~MGC:BA3106~BA697253~RFU:0 |
| Anthrax008~B36R06C03 | 0.014676326 | Ba~MGC:BA1093~BA696272~RFU:48055.79 |
| Anthrax008~B02R04C16 | 0.014651174 | Ba~MGC:BA1170~BA696303~RFU:60091.98 |
| Anthrax008~B03R04C19 | 0.014733402 | Ba~MGC:BA0017~BA695735~RFU:60011.96 |
| Anthrax008~B31R02C08 | 0.014830146 | Ba~MGC:BA3089~BA697241~RFU:60101.18 |
| Anthrax008~B45R07C10 | 0.014930744 | Ba~MGC:BA4309~BA697836~RFU:959.94 |
| Anthrax008~B15R07C05 | 0.015041069 | Ba~MGC:BA4168~BA697763~RFU:60115.89 |
| Anthrax008~B02R02C08 | 0.015045176 | Ba~MGC:BA4692~BA698039~RFU:6000.74 |
| Anthrax008~B02R06C09 | 0.015271971 | Ba~MGC:BA2509~BA696962~RFU:60088.3 |
| Anthrax008~B02R06C10 | 0.01526349 | Ba~MGC:BA2509~BA696962~RFU:60094.74 |
| Anthrax008~B18R03C19 | 0.015268573 | Ba~MGC:BA0517~BA696006~RFU:59965.97 |
| Anthrax008~B27R05C19 | 0.015224281 | Ba~MGC:BA1615~BA696524~RFU:59626.61 |
| Anthrax008~B32R09C20 | 0.015311159 | Ba~MGC:BA5137~BA13404~RFU:6499.26 |
| Anthrax008~B35R16C16 | 0.015310712 | Ba~MGC:BA4953~BA17395~RFU:19850.99 |
| Anthrax008~B03R03C02 | 0.015340249 | Ba~MGC:BA2995~BA697189~RFU:60054.27 |
| Anthrax008~B39R05C19 | 0.015363765 | Ba~MGC:BA4264~BA697811~RFU:59979.76 |
| Anthrax008~B36R12C13 | 0.015396291 | Ba~MGC:BXB0002~BA13943~RFU:32204.75 |
| Anthrax008~B32R09C19 | 0.01547219 | Ba~MGC:BA5137~BA13404~RFU:6396.25 |
| Anthrax008~B18R13C17 | 0.01551452 | Ba~MGC:BA3905~BA3905~RFU:55903.24 |
| Anthrax008~B04R07C07 | 0.015688566 | Ba~MGC:BA5412~BA698416~RFU:33875.09 |
| Anthrax008~B27R05C20 | 0.01589478 | Ba~MGC:BA1615~BA696524~RFU:36403.43 |
| Anthrax008~B48R04C01 | 0.015976368 | Ba~MGC:BA3621~BA697487~RFU:60099.34 |
| Anthrax008~B12R10C13 | 0.015968397 | Ba~MGC:BXA0188~BA13750~RFU:6674.03 |
| Anthrax008~B46R06C01 | 0.016010089 | Ba~MGC:BA4394.1~BA697891~RFU:60026.67 |
| Anthrax008~B48R04C02 | 0.016097823 | Ba~MGC:BA3621~BA697487~RFU:60025.75 |
| Anthrax008~B14R06C06 | 0.016130704 | Ba~MGC:BA1949~BA696689~RFU:60005.52 |
| Anthrax008~B04R10C06 | 0.0161437 | Ba~MGC:BA4548~BA15688~RFU:3514.53 |
| Anthrax008~B46R05C14 | 0.016077638 | Ba~MGC:BA1891~BA696659~RFU:60086.46 |
| Anthrax008~B33R02C13 | 0.01610571 | Ba~MGC:BA1538~BA696481~RFU:60014.72 |
| Anthrax008~B36R05C19 | 0.01638147 | Ba~MGC:BA2145~BA696784~RFU:59972.41 |
| Anthrax008~B09R05C06 | 0.016538749 | Ba~MGC:BA4525~BA697956~RFU:59927.34 |
| Anthrax008~B16R09C17 | 0.016781459 | Ba~MGC:BA4971~BA13890~RFU:3117.18 |
| Anthrax008~B39R04C10 | 0.01711701 | Ba~MGC:BA1462~BA696444~RFU:60087.38 |
| Anthrax008~B18R10C16 | 0.017114855 | Ba~MGC:BXB0074~BA15994~RFU:6068.67 |
| Anthrax008~B45R02C13 | 0.017196951 | Ba~MGC:BA2692~BA697044~RFU:60036.79 |
| Anthrax008~B18R03C20 | 0.017215965 | Ba~MGC:BA0517~BA696006~RFU:60043.23 |
| Anthrax008~B14R03C09 | 0.017230533 | Ba~MGC:BA2434~BA696932~RFU:27050.22 |
| Anthrax008~B30R08C13 | 0.017607981 | Ba~MGC:BA2717~BA14920~RFU:2.86 |
| Anthrax008~B17R06C08 | 0.017659131 | Ba~MGC:BA0678~BA696079~RFU:35628.22 |
| Anthrax008~B27R11C08 | 0.01773507 | Ba~MGC:BA5077~BA16056~RFU:2026.31 |
| Anthrax008~B28R04C16 | 0.01784349 | Ba~MGC:BA0366~BA695932~RFU:60043.23 |
| Anthrax008~B01R07C03 | 0.017917436 | Ba~MGC:BA5248~BA698328~RFU:60097.5 |
| Anthrax008~B30R13C17 | 0.017967263 | Ba~MGC:BA2252~BA14248~RFU:60108.54 |
| Anthrax008~B37R03C01 | 0.017955061 | Ba~MGC:BA3303~BA697325~RFU:108.73 |
| Anthrax008~B39R12C09 | 0.018223517 | Ba~MGC:BA3897~BA3897~RFU:32684.88 |
| Anthrax008~B15R04C07 | 0.018257473 | Ba~MGC:BA1182~BA696306~RFU:60078.18 |
| Anthrax008~B45R03C04 | 0.018313635 | Ba~MGC:BA3990~BA697676~RFU:13.8 |
| Anthrax008~B32R02C03 | 0.018514259 | Ba~MGC:BA2114~BA696768~RFU:0 |
| Anthrax008~B46R05C13 | 0.018622251 | Ba~MGC:BA1891~BA696659~RFU:60054.27 |
| Anthrax008~B13R04C02 | 0.018790513 | Ba~MGC:BA0461~BA695981~RFU:60032.19 |
| Anthrax008~B45R02C14 | 0.018817607 | Ba~MGC:BA2692~BA697044~RFU:60065.31 |
| Anthrax008~B22R06C04 | 0.018871248 | Ba~MGC:BA2224~BA696823~RFU:60068.06 |
| Anthrax008~B31R02C07 | 0.018879067 | Ba~MGC:BA3089~BA697241~RFU:60115.89 |
| Anthrax008~B16R09C18 | 0.018918626 | Ba~MGC:BA4971~BA13890~RFU:2877.12 |
| Anthrax008~B02R02C07 | 0.019007275 | Ba~MGC:BA4692~BA698039~RFU:6424.76 |
| Anthrax008~B45R02C17 | 0.019051358 | Ba~MGC:BA4886~BA698141~RFU:0 |
| Anthrax008~B07R14C19 | 0.019092513 | Ba~MGC:BA4104~BA697734~RFU:60111.3 |
| Anthrax008~B43R03C06 | 0.01916557 | Ba~MGC:BA4679~BA698029~RFU:60022.08 |
| Anthrax008~B13R07C06 | 0.019331263 | Ba~MGC:BA5584~BA698498~RFU:38677.34 |
| Anthrax008~B25R10C04 | 0.019447938 | Ba~MGC:BA3489~BA14022~RFU:17178.07 |
| Anthrax008~B10R04C09 | 0.01949002 | Ba~MGC:BA2931~BA697152~RFU:59927.34 |
| Anthrax008~B11R14C14 | 0.019513214 | Ba~MGC:BA4213~BA697786~RFU:60095.66 |
| Anthrax008~B24R07C20 | 0.019610884 | Ba~MGC:BA4690~BA698038~RFU:1811.07 |
| Anthrax008~B07R11C03 | 0.019663617 | Ba~MGC:BA4995~BA14866~RFU:1606.88 |
| Anthrax008~B33R04C14 | 0.019682854 | Ba~MGC:BA1194~BA696313~RFU:60008.28 |
| Anthrax008~B44R02C07 | 0.019711848 | Ba~MGC:BA3990~BA697676~RFU:60080.02 |
| Anthrax008~B17R06C07 | 0.019888954 | Ba~MGC:BA0678~BA696079~RFU:56475.35 |
| Anthrax008~B14R02C11 | 0.01995411 | Ba~MGC:BA1877~BA696654~RFU:34.33 |
| Anthrax008~B07R14C20 | 0.020073188 | Ba~MGC:BA4104~BA697734~RFU:60123.25 |
| Anthrax008~B46R05C10 | 0.020355392 | Ba~MGC:BA2207~BA696814~RFU:60054.27 |
| Anthrax008~B01R01C20 | 0.02060482 | Ba~MGC:BA3092~BA697243~RFU:60121.41 |
| Anthrax008~B11R14C13 | 0.02060379 | Ba~MGC:BA4213~BA697786~RFU:60108.54 |
| Anthrax008~B08R07C14 | 0.020746483 | Ba~MGC:BA0107~BA695790~RFU:10.12 |
| Anthrax008~B16R14C16 | 0.020942159 | Ba~MGC:BA2529~BA698781~RFU:60091.06 |
| Anthrax008~B11R03C04 | 0.021255333 | Ba~MGC:BA2450~BA696942~RFU:60041.39 |
| Anthrax008~B09R05C05 | 0.021249605 | Ba~MGC:BA4525~BA697956~RFU:0 |
| Anthrax008~B30R02C11 | 0.021502184 | Ba~MGC:BA0170~BA695825~RFU:0 |
| Anthrax008~B13R14C14 | 0.021486389 | Ba~MGC:BA3529~BA697440~RFU:48961.55 |
| Anthrax008~B01R06C17 | 0.021471964 | Ba~MGC:BA3974~BA697669~RFU:60119.57 |
| Anthrax008~B14R03C13 | 0.021927437 | Ba~MGC:BA0259~BA695868~RFU:60032.19 |
| Anthrax008~B19R03C10 | 0.022048822 | Ba~MGC:BA1820~BA696626~RFU:60061.63 |
| Anthrax008~B14R02C07 | 0.022160215 | Ba~MGC:BA0946~BA696212~RFU:0 |
| Anthrax008~B44R11C17 | 0.022167288 | Ba~MGC:BA4182~BA16750~RFU:29185.98 |
| Anthrax008~B14R06C19 | 0.022195358 | Ba~MGC:BA0631.1~BA696057~RFU:57388.7 |
| Anthrax008~B45R06C18 | 0.022286378 | Ba~MGC:BA4565.1~BA697979~RFU:59958.61 |
| Anthrax008~B11R03C09 | 0.022348002 | Ba~MGC:BA1226~BA696329~RFU:49217.26 |
| Anthrax008~B22R06C03 | 0.022377264 | Ba~MGC:BA2224~BA696823~RFU:60049.67 |
| Anthrax008~B36R06C04 | 0.022516007 | Ba~MGC:BA1093~BA696272~RFU:31201.72 |
| Anthrax008~B44R02C08 | 0.022678522 | Ba~MGC:BA3990~BA697676~RFU:60068.98 |
| Anthrax008~B28R06C02 | 0.02270183 | Ba~MGC:BA4526~BA697957~RFU:60089.22 |
| Anthrax008~B23R08C03 | 0.022742108 | Ba~MGC:BA4307~BA697835~RFU:2684.88 |
| Anthrax008~B14R08C05 | 0.02290452 | Ba~MGC:BA2357~BA13670~RFU:0 |
| Anthrax008~B18R14C12 | 0.02295872 | Ba~MGC:BA5477~BA698825~RFU:95.85 |
| Anthrax008~B27R05C06 | 0.022938108 | Ba~MGC:BA1138~BA696290~RFU:60036.79 |
| Anthrax008~B46R05C20 | 0.02317331 | Ba~MGC:BA2016~BA696718~RFU:30826.9 |
| Anthrax008~B18R03C14 | 0.023178315 | Ba~MGC:BA4236~BA697797~RFU:60043.23 |
| Anthrax008~B46R02C03 | 0.02313954 | Ba~MGC:BA1013~BA696235~RFU:0 |
| Anthrax008~B07R05C06 | 0.023255005 | Ba~MGC:BA2844~BA697112~RFU:60077.26 |
| Anthrax008~B13R07C05 | 0.02343452 | Ba~MGC:BA5584~BA698498~RFU:41192.05 |
| Anthrax008~B27R07C06 | 0.02353458 | Ba~MGC:BA5298~BA698353~RFU:13165.95 |
| Anthrax008~B22R02C19 | 0.023739837 | Ba~MGC:BA0731~BA696106~RFU:59756.25 |
| Anthrax008~B43R03C09 | 0.023877429 | Ba~MGC:BA5641~BA698525~RFU:60050.59 |
| Anthrax008~B18R12C14 | 0.024115847 | Ba~MGC:BXA0119~BA13500~RFU:33982.71 |
| Anthrax008~B28R04C15 | 0.024154063 | Ba~MGC:BA0366~BA695932~RFU:60004.6 |
| Anthrax008~B32R02C09 | 0.024194902 | Ba~MGC:BA2119~BA696770~RFU:0 |
| Anthrax008~B22R13C19 | 0.024222283 | Ba~MGC:BA5281~BA13591~RFU:60093.82 |
| Anthrax008~B38R04C10 | 0.024291232 | Ba~MGC:BA3620~BA697486~RFU:60047.83 |
| Anthrax008~B10R04C14 | 0.02428637 | Ba~MGC:BA0715~BA696096~RFU:0 |
| Anthrax008~B34R04C15 | 0.024571363 | Ba~MGC:BA2569~BA696986~RFU:60112.21 |
| Anthrax008~B14R06C03 | 0.024656458 | Ba~MGC:BA4664~BA698024~RFU:59945.73 |
| Anthrax008~B34R04C05 | 0.024706889 | Ba~MGC:BA3500~BA697424~RFU:30730.32 |
| Anthrax008~B01R06C18 | 0.024708018 | Ba~MGC:BA3974~BA697669~RFU:60122.33 |
| Anthrax008~B28R06C01 | 0.024774345 | Ba~MGC:BA4526~BA697957~RFU:60096.58 |
| Anthrax008~B39R03C04 | 0.025072707 | Ba~MGC:BA1488~BA696456~RFU:60069.9 |
| Anthrax008~B11R03C10 | 0.025111383 | Ba~MGC:BA1226~BA696329~RFU:52805.37 |
| Anthrax008~B27R07C05 | 0.02513034 | Ba~MGC:BA5298~BA698353~RFU:12893.67 |
| Anthrax008~B13R04C01 | 0.025095517 | Ba~MGC:BA0461~BA695981~RFU:60038.63 |
| Anthrax008~B33R04C13 | 0.025087569 | Ba~MGC:BA1194~BA696313~RFU:59951.25 |
| Anthrax008~B11R03C03 | 0.025026174 | Ba~MGC:BA2450~BA696942~RFU:60040.47 |
| Anthrax008~B12R14C07 | 0.025304431 | Ba~MGC:BA4091~BA697729~RFU:211.55 |
| Anthrax008~B36R02C10 | 0.025551762 | Ba~MGC:BA3641~BA697496~RFU:60074.5 |
| Anthrax008~B10R04C13 | 0.025555829 | Ba~MGC:BA0715~BA696096~RFU:54276.11 |
| Anthrax008~B22R08C09 | 0.025629712 | Ba~MGC:BA2431~BA13438~RFU:0 |
| Anthrax008~B02R03C18 | 0.025979673 | Ba~MGC:BA1227~BA696330~RFU:60099.34 |
| Anthrax008~B36R02C12 | 0.026045272 | Ba~MGC:BA3921~BA697635~RFU:60044.15 |
| Anthrax008~B10R14C18 | 0.026046284 | Ba~MGC:BA2878~BA15454~RFU:60069.9 |
| Anthrax008~B40R03C13 | 0.026528457 | Ba~MGC:BA5351~BA698380~RFU:59990.8 |
| Anthrax008~B35R04C15 | 0.026750963 | Ba~MGC:BA5569~BA698488~RFU:58593.64 |
| Anthrax008~B34R04C06 | 0.02684226 | Ba~MGC:BA3500~BA697424~RFU:28423.47 |
| Anthrax008~B25R02C17 | 0.026816884 | Ba~MGC:BA5136~BA698270~RFU:28145.92 |
| Anthrax008~B19R04C12 | 0.02715566 | Ba~MGC:BA2717.1~BA697053~RFU:57585.54 |
| Anthrax008~B22R13C20 | 0.027156806 | Ba~MGC:BA5281~BA13591~RFU:60102.1 |
| Anthrax008~B27R11C06 | 0.027139576 | Ba~MGC:BA5290~BA14230~RFU:34543.78 |
| Anthrax008~B03R04C13 | 0.02728595 | Ba~MGC:BA4299~BA697831~RFU:60047.83 |
| Anthrax008~B46R04C03 | 0.027585186 | Ba~MGC:BA2002~BA696712~RFU:59953.09 |
| Anthrax008~B35R15C04 | 0.027681058 | Ba~MGC:BA4840~BA14142~RFU:27473.33 |
| Anthrax008~B26R10C19 | 0.027840998 | Ba~MGC:BXA0196~BA14076~RFU:8632.27 |
| Anthrax008~B30R13C18 | 0.027839573 | Ba~MGC:BA2252~BA14248~RFU:60117.73 |
| Anthrax008~B48R05C18 | 0.027890433 | Ba~MGC:BA0063~BA695761~RFU:60034.95 |
| Anthrax008~B24R15C12 | 0.027969705 | Ba~MGC:BA0774~BA17122~RFU:541.76 |
| Anthrax008~B24R02C03 | 0.028126266 | Ba~MGC:BA3580~BA697470~RFU:27.18 |
| Anthrax008~B19R02C08 | 0.028141523 | Ba~MGC:BA5062~BA698231~RFU:60045.07 |
| Anthrax008~B34R04C20 | 0.028135605 | Ba~MGC:BA3458~BA697402~RFU:60055.19 |
| Anthrax008~B34R04C16 | 0.02819607 | Ba~MGC:BA2569~BA696986~RFU:59707.51 |
| Anthrax008~B09R02C15 | 0.028233586 | Ba~MGC:BA1181~BA696305~RFU:0 |
| Anthrax008~B31R13C05 | 0.028315717 | Ba~MGC:BA4768~BA14360~RFU:33860.38 |
| Anthrax008~B10R10C06 | 0.028549206 | Ba~MGC:BA4124~BA13292~RFU:10150.21 |
| Anthrax008~B18R14C11 | 0.02880785 | Ba~MGC:BA5477~BA698825~RFU:165.56 |
| Anthrax008~B27R14C10 | 0.02880976 | Ba~MGC:BA1240~BA696339~RFU:43044.52 |
| Anthrax008~B08R06C11 | 0.028961271 | Ba~MGC:BA0376~BA695938~RFU:60117.73 |
| Anthrax008~B19R03C13 | 0.028951723 | Ba~MGC:BA1721~BA696574~RFU:60053.35 |
| Anthrax008~B43R05C08 | 0.028953286 | Ba~MGC:BA1719~BA696573~RFU:60016.56 |
| Anthrax008~B14R06C04 | 0.02918268 | Ba~MGC:BA4664~BA698024~RFU:59853.75 |
| Anthrax008~B35R06C08 | 0.029390216 | Ba~MGC:BA2551~BA696977~RFU:60076.34 |
| Anthrax008~B14R10C09 | 0.029410321 | Ba~MGC:BA5311~BA14666~RFU:5215.23 |
| Anthrax008~B32R13C11 | 0.029551355 | Ba~MGC:BA4399~BA4399~RFU:580.39 |
| Anthrax008~B10R06C16 | 0.029605916 | Ba~MGC:BA4896~BA698150~RFU:22297.57 |
| Anthrax008~B19R03C09 | 0.029582191 | Ba~MGC:BA1820~BA696626~RFU:60067.14 |
| Anthrax008~B29R05C20 | 0.02973725 | Ba~MGC:BA0503~BA695998~RFU:60079.1 |
| Anthrax008~B14R02C05 | 0.02984106 | Ba~MGC:BA4383~BA697885~RFU:0 |
| Anthrax008~B27R11C05 | 0.0299693 | Ba~MGC:BA5290~BA14230~RFU:21699.57 |
| Anthrax008~B03R04C14 | 0.030405704 | Ba~MGC:BA4299~BA697831~RFU:60029.43 |
| Anthrax008~B02R03C01 | 0.030494738 | Ba~MGC:BA4359~BA697872~RFU:43159.49 |
| Anthrax008~B44R06C10 | 0.03064369 | Ba~MGC:BA5601~BA698504~RFU:59962.29 |
| Anthrax008~B14R05C05 | 0.030638056 | Ba~MGC:BA5452~BA698438~RFU:59654.16 |
| Anthrax008~B42R03C13 | 0.030703917 | Ba~MGC:BA3654~BA697501~RFU:60005.52 |
| Anthrax008~B19R04C15 | 0.030804262 | Ba~MGC:BA2305~BA696869~RFU:60035.87 |
| Anthrax008~B11R15C05 | 0.030796664 | Ba~MGC:BA0072~BA695766~RFU:59988.96 |
| Anthrax008~B08R06C07 | 0.030814802 | Ba~MGC:BA3628~BA697489~RFU:60095.66 |
| Anthrax008~B35R14C12 | 0.0313771 | Ba~MGC:BA1674~BA696554~RFU:0 |
| Anthrax008~B35R04C16 | 0.031410594 | Ba~MGC:BA5569~BA698488~RFU:59855.59 |
| Anthrax008~B35R06C07 | 0.031744808 | Ba~MGC:BA2551~BA696977~RFU:60070.82 |
| Anthrax008~B13R06C08 | 0.031778738 | Ba~MGC:BA3845~BA697596~RFU:50802.06 |
| Anthrax008~B46R04C17 | 0.031761754 | Ba~MGC:BA1585~BA696509~RFU:60043.23 |
| Anthrax008~B14R02C10 | 0.031733237 | Ba~MGC:BA0438~BA695972~RFU:8.58 |
| Anthrax008~B44R06C09 | 0.03179833 | Ba~MGC:BA5601~BA698504~RFU:59976.09 |
| Anthrax008~B36R11C07 | 0.0317153 | Ba~MGC:BXA0026~BA15896~RFU:4456.4 |
| Anthrax008~B47R07C03 | 0.031973194 | Ba~MGC:BA4556.1~BA697973~RFU:60104.86 |
| Anthrax008~B39R03C03 | 0.03208176 | Ba~MGC:BA1488~BA696456~RFU:60059.79 |
| Anthrax008~B02R03C17 | 0.032119136 | Ba~MGC:BA1227~BA696330~RFU:60089.22 |
| Anthrax008~B05R02C01 | 0.03216563 | Ba~MGC:BA4869~BA698132~RFU:29094.92 |
| Anthrax008~B33R02C14 | 0.032237235 | Ba~MGC:BA1538~BA696481~RFU:60023.91 |
| Anthrax008~B46R06C17 | 0.032418624 | Ba~MGC:BA1829~BA698766~RFU:60108.54 |
| Anthrax008~B10R14C17 | 0.032440964 | Ba~MGC:BA2878~BA15454~RFU:60045.99 |
| Anthrax008~B13R04C12 | 0.032489583 | Ba~MGC:BA1485~BA696454~RFU:24301.86 |
| Anthrax008~B04R06C10 | 0.03267671 | Ba~MGC:BA0842~BA696159~RFU:59986.2 |
| Anthrax008~B43R04C06 | 0.032790143 | Ba~MGC:BA0744.1~BA696113~RFU:59847.31 |
| Anthrax008~B44R11C18 | 0.033203453 | Ba~MGC:BA4182~BA16750~RFU:25233.19 |
| Anthrax008~B19R04C17 | 0.033372924 | Ba~MGC:BA4673~BA698027~RFU:59973.33 |
| Anthrax008~B05R02C02 | 0.033448275 | Ba~MGC:BA4869~BA698132~RFU:29176.78 |
| Anthrax008~B11R04C16 | 0.03343709 | Ba~MGC:BA0119~BA695795~RFU:59971.49 |
| Anthrax008~B40R06C11 | 0.0335626 | Ba~MGC:BA5121~BA698264~RFU:0 |
| Anthrax008~B32R02C14 | 0.03360758 | Ba~MGC:BA3425~BA697389~RFU:60006.44 |
| Anthrax008~B36R02C11 | 0.033587545 | Ba~MGC:BA3921~BA697635~RFU:60064.39 |
| Anthrax008~B02R06C05 | 0.033804953 | Ba~MGC:BA2136~BA696779~RFU:36301.51 |
| Anthrax008~B27R04C13 | 0.03394503 | Ba~MGC:BA4154~BA697758~RFU:60021.16 |
| Anthrax008~B43R04C05 | 0.034078225 | Ba~MGC:BA0744.1~BA696113~RFU:59805.92 |
| Anthrax008~B04R06C09 | 0.034071226 | Ba~MGC:BA0842~BA696159~RFU:59946.65 |
| Anthrax008~B22R08C15 | 0.03421185 | Ba~MGC:BA2350~BA16176~RFU:0 |
| Anthrax008~B21R06C14 | 0.034293678 | Ba~MGC:BA4867~BA698130~RFU:60075.42 |
| Anthrax008~B26R03C08 | 0.03427269 | Ba~MGC:BA5284~BA698346~RFU:60088.3 |
| Anthrax008~B48R10C11 | 0.0345343 | Ba~MGC:BA4678~BA15900~RFU:1159.86 |
| Anthrax008~B32R10C07 | 0.034669988 | Ba~MGC:BA4524~BA16238~RFU:4443.49 |
| Anthrax008~B08R06C12 | 0.034653965 | Ba~MGC:BA0376~BA695938~RFU:60113.13 |
| Anthrax008~B11R15C06 | 0.034727536 | Ba~MGC:BA0072~BA695766~RFU:59992.64 |
| Anthrax008~B43R03C10 | 0.03492711 | Ba~MGC:BA5641~BA698525~RFU:60055.19 |
| Anthrax008~B24R14C03 | 0.035077516 | Ba~MGC:BA0309~BA695897~RFU:60102.1 |
| Anthrax008~B01R03C04 | 0.035298686 | Ba~MGC:BA2841~BA697110~RFU:60119.57 |
| Anthrax008~B42R04C10 | 0.035251956 | Ba~MGC:BA1600~BA696516~RFU:60023.91 |
| Anthrax008~B40R04C16 | 0.035320774 | Ba~MGC:BA4760~BA698074~RFU:59944.81 |
| Anthrax008~B43R07C02 | 0.035429694 | Ba~MGC:BA0086~BA695778~RFU:6129.51 |
| Anthrax008~B02R06C06 | 0.0356153 | Ba~MGC:BA2136~BA696779~RFU:40465.42 |
| Anthrax008~B40R04C15 | 0.035939716 | Ba~MGC:BA4760~BA698074~RFU:59832.6 |
| Anthrax008~B16R06C03 | 0.035911735 | Ba~MGC:BA4100~BA697733~RFU:60077.26 |
| Anthrax008~B01R06C11 | 0.03629598 | Ba~MGC:BA1188~BA696311~RFU:60045.07 |
| Anthrax008~B30R06C09 | 0.03639358 | Ba~MGC:BA4952~BA698181~RFU:4473.88 |
| Anthrax008~B19R04C16 | 0.03645089 | Ba~MGC:BA2305~BA696869~RFU:59968.73 |
| Anthrax008~B16R06C04 | 0.03667807 | Ba~MGC:BA4100~BA697733~RFU:60046.91 |
| Anthrax008~B48R06C03 | 0.03688636 | Ba~MGC:BA1526~BA696475~RFU:0 |
| Anthrax008~B32R10C08 | 0.037030462 | Ba~MGC:BA4524~BA16238~RFU:4280.4 |
| Anthrax008~B27R06C08 | 0.03702799 | Ba~MGC:BA0100~BA695787~RFU:60075.42 |
| Anthrax008~B46R10C06 | 0.037508186 | Ba~MGC:BA4779~BA13646~RFU:68.98 |
| Anthrax008~B37R06C08 | 0.037695773 | Ba~MGC:BA4148~BA697754~RFU:2582.78 |
| Anthrax008~B27R11C07 | 0.037835646 | Ba~MGC:BA5077~BA16056~RFU:2159.68 |
| Anthrax008~B10R06C15 | 0.037847973 | Ba~MGC:BA4896~BA698150~RFU:22277.41 |
| Anthrax008~B19R05C18 | 0.037936144 | Ba~MGC:BA5081~BA698243~RFU:60079.1 |
| Anthrax008~B02R05C03 | 0.037959464 | Ba~MGC:BA1469~BA696446~RFU:60058.87 |
| Anthrax008~B24R14C04 | 0.03821498 | Ba~MGC:BA0309~BA695897~RFU:60107.62 |
| Anthrax008~B30R02C03 | 0.038206927 | Ba~MGC:BA2240~BA696835~RFU:0 |
| Anthrax008~B14R04C14 | 0.038640477 | Ba~MGC:BA1582~BA696508~RFU:60024.83 |
| Anthrax008~B48R06C04 | 0.03870685 | Ba~MGC:BA1526~BA696475~RFU:26153.08 |
| Anthrax008~B12R07C06 | 0.03951885 | Ba~MGC:BA1323~BA696383~RFU:60094.74 |
| Anthrax008~B10R07C04 | 0.0395762 | Ba~MGC:BA0280~BA695880~RFU:59841.8 |
| Anthrax008~B19R05C20 | 0.03980635 | Ba~MGC:BA0016~BA695734~RFU:60070.82 |
| Anthrax008~B28R03C07 | 0.04004624 | Ba~MGC:BA3813~BA697576~RFU:59974.25 |
| Anthrax008~B21R05C14 | 0.04091053 | Ba~MGC:BA5515~BA698465~RFU:60077.26 |
| Anthrax008~B02R05C04 | 0.04089691 | Ba~MGC:BA1469~BA696446~RFU:3095.85 |
| Anthrax008~B23R05C08 | 0.041068107 | Ba~MGC:BA3842~BA697593~RFU:59890.54 |
| Anthrax008~B11R06C06 | 0.04126265 | Ba~MGC:BA3754~BA697551~RFU:6959.94 |
| Anthrax008~B46R06C19 | 0.04146461 | Ba~MGC:BA4823~BA698105~RFU:23904.15 |
| Anthrax008~B12R07C05 | 0.041462123 | Ba~MGC:BA1323~BA696383~RFU:60071.74 |
| Anthrax008~B27R04C14 | 0.04145572 | Ba~MGC:BA4154~BA697758~RFU:60050.59 |
| Anthrax008~B15R03C06 | 0.041609317 | Ba~MGC:BA2917~BA697145~RFU:60106.7 |
| Anthrax008~B16R08C19 | 0.04160069 | Ba~MGC:BA2344~BA13830~RFU:60021.16 |
| Anthrax008~B38R05C17 | 0.04173926 | Ba~MGC:BA2252.1~BA696844~RFU:60052.43 |
| Anthrax008~B36R11C08 | 0.041897032 | Ba~MGC:BXA0026~BA15896~RFU:4711.18 |
| Anthrax008~B16R02C06 | 0.042194795 | Ba~MGC:BA2253~BA696845~RFU:0 |
| Anthrax008~B15R04C08 | 0.042228963 | Ba~MGC:BA1182~BA696306~RFU:60054.27 |
| Anthrax008~B10R04C10 | 0.04219976 | Ba~MGC:BA2931~BA697152~RFU:59951.25 |
| Anthrax008~B24R02C09 | 0.0427272 | Ba~MGC:BA1242~BA696341~RFU:0 |
| Anthrax008~B14R04C13 | 0.04293129 | Ba~MGC:BA1582~BA696508~RFU:53111.66 |
| Anthrax008~B09R02C04 | 0.042939357 | Ba~MGC:BA0197~BA695841~RFU:60091.06 |
| Anthrax008~B04R06C05 | 0.0429756 | Ba~MGC:BA4573~BA697982~RFU:59795.81 |
| Anthrax008~B11R04C15 | 0.043035105 | Ba~MGC:BA0119~BA695795~RFU:59978.84 |
| Anthrax008~B39R05C09 | 0.043204412 | Ba~MGC:BA1487~BA696455~RFU:60084.62 |
| Anthrax008~B13R06C07 | 0.043378886 | Ba~MGC:BA3845~BA697596~RFU:37391.46 |
| Anthrax008~B27R14C09 | 0.043433014 | Ba~MGC:BA1240~BA696339~RFU:41547.09 |
| Anthrax008~B48R06C13 | 0.04469231 | Ba~MGC:BA0048~BA695753~RFU:60050.59 |
| Anthrax008~B34R04C01 | 0.04482289 | Ba~MGC:BA3861~BA697601~RFU:60086.46 |
| Anthrax008~B08R08C20 | 0.044789825 | Ba~MGC:BA2342~BA14170~RFU:26489.15 |
| Anthrax008~B44R06C08 | 0.04502615 | Ba~MGC:BA0760~BA696120~RFU:59971.49 |
| Anthrax008~B31R06C06 | 0.04523571 | Ba~MGC:BA0684~BA696081~RFU:60052.43 |
| Anthrax008~B14R05C06 | 0.045316245 | Ba~MGC:BA5452~BA698438~RFU:59999.08 |
| Anthrax008~B21R02C18 | 0.045564085 | Ba~MGC:BA5724~BA698571~RFU:60103.94 |
| Anthrax008~B35R07C03 | 0.04563115 | Ba~MGC:BA4051~BA697710~RFU:60088.3 |
| Anthrax008~B09R06C05 | 0.045949176 | Ba~MGC:BA2625~BA697012~RFU:59617.37 |
| Anthrax008~B22R05C07 | 0.04594975 | Ba~MGC:BA2880~BA697129~RFU:60039.55 |
| Anthrax008~B44R06C07 | 0.046039328 | Ba~MGC:BA0760~BA696120~RFU:0 |
| Anthrax008~B38R05C09 | 0.04608207 | Ba~MGC:BA1472~BA696447~RFU:60038.63 |
| Anthrax008~B40R08C05 | 0.04618536 | Ba~MGC:BA2178~BA16164~RFU:0 |
| Anthrax008~B31R06C05 | 0.046144705 | Ba~MGC:BA0684~BA696081~RFU:60047.83 |
| Anthrax008~B35R07C04 | 0.046291094 | Ba~MGC:BA4051~BA697710~RFU:60110.38 |
| Anthrax008~B40R05C08 | 0.046442952 | Ba~MGC:BA1564~BA696497~RFU:60015.64 |
| Anthrax008~B01R03C03 | 0.046992972 | Ba~MGC:BA2841~BA697110~RFU:60125.09 |
| Anthrax008~B37R02C15 | 0.047112804 | Ba~MGC:BA2122~BA696772~RFU:0 |
| Anthrax008~B08R04C09 | 0.047074035 | Ba~MGC:BA2765~BA697074~RFU:60106.7 |
| Anthrax008~B28R04C06 | 0.047511548 | Ba~MGC:BA1729.1~BA696579~RFU:59204.38 |
| Anthrax008~B46R04C04 | 0.04753722 | Ba~MGC:BA2002~BA696712~RFU:60019.32 |
| Anthrax008~B34R07C01 | 0.047542818 | Ba~MGC:BA4193~BA697776~RFU:60115.89 |
| Anthrax008~B47R03C03 | 0.047651894 | Ba~MGC:BA2279~BA696856~RFU:60062.55 |
| Anthrax008~B10R07C03 | 0.04774222 | Ba~MGC:BA0280~BA695880~RFU:59875.83 |
| Anthrax008~B04R06C06 | 0.04787291 | Ba~MGC:BA4573~BA697982~RFU:59919.98 |
| Anthrax008~B46R05C19 | 0.048343826 | Ba~MGC:BA2016~BA696718~RFU:46804.01 |
| Anthrax008~B15R06C16 | 0.048492987 | Ba~MGC:BA1213~BA696321~RFU:60099.34 |
| Anthrax008~B47R07C04 | 0.048585508 | Ba~MGC:BA4556.1~BA697973~RFU:60101.18 |
| Anthrax008~B34R04C19 | 0.048692375 | Ba~MGC:BA3458~BA697402~RFU:60048.75 |
| Anthrax008~B10R02C09 | 0.04883156 | Ba~MGC:BA5361.1~BA698389~RFU:60034.03 |
| Anthrax008~B34R04C02 | 0.048839033 | Ba~MGC:BA3861~BA697601~RFU:60092.9 |
| Anthrax008~B21R02C17 | 0.048972875 | Ba~MGC:BA5724~BA698571~RFU:60092.9 |
| Anthrax008~B47R03C04 | 0.049115874 | Ba~MGC:BA2279~BA696856~RFU:60083.7 |
| Anthrax008~B36R10C08 | 0.049909074 | Ba~MGC:BA3475~BA13908~RFU:10081.55 |

**C**

| **ID** | **p value** | **Protein Feature Name** |
| --- | --- | --- |
| Anthrax008~B28R03C08 | 1.69E-07 | Ba~MGC:BA3813~BA697576~RFU:59972.41 |
| Anthrax008~B40R01C20 | 3.18E-07 | Ba~MGC:BA1502~BA696462~RFU:0 |
| Anthrax008~B24R02C05 | 6.58E-07 | Ba~MGC:BA3391~BA697370~RFU:0 |
| Anthrax008~B14R08C15 | 6.74E-07 | Ba~MGC:BA2441~BA15578~RFU:0 |
| Anthrax008~B14R08C20 | 1.81E-06 | Ba~MGC:BA2716~BA15164~RFU:12843.08 |
| Anthrax008~B01R05C03 | 2.03E-06 | Ba~MGC:BA2933~BA697154~RFU:60090.14 |
| Anthrax008~B16R06C04 | 2.29E-06 | Ba~MGC:BA4100~BA697733~RFU:60046.91 |
| Anthrax008~B04R03C03 | 2.30E-06 | Ba~MGC:BA2919~BA697146~RFU:0 |
| Anthrax008~B01R05C04 | 2.38E-06 | Ba~MGC:BA2933~BA697154~RFU:42981.4 |
| Anthrax008~B32R01C19 | 3.30E-06 | Ba~MGC:BA3088~BA697240~RFU:7.36 |
| Anthrax008~B38R04C02 | 3.46E-06 | Ba~MGC:BA1897~BA696662~RFU:59866.63 |
| Anthrax008~B47R04C17 | 3.51E-06 | Ba~MGC:BA5076~BA698241~RFU:60054.27 |
| Anthrax008~B16R06C03 | 3.64E-06 | Ba~MGC:BA4100~BA697733~RFU:60077.26 |
| Anthrax008~B15R03C07 | 4.65E-06 | Ba~MGC:BA3167~BA697271~RFU:0 |
| Anthrax008~B36R06C16 | 5.23E-06 | Ba~MGC:BA2591~BA698782~RFU:59971.49 |
| Anthrax008~B45R03C04 | 6.06E-06 | Ba~MGC:BA3990~BA697676~RFU:13.8 |
| Anthrax008~B24R01C19 | 6.18E-06 | Ba~MGC:BA1534~BA696478~RFU:0 |
| Anthrax008~B32R08C11 | 6.85E-06 | Ba~MGC:BA2541~BA15044~RFU:2894.13 |
| Anthrax008~B32R01C18 | 8.42E-06 | Ba~MGC:BA3461~BA697403~RFU:0 |
| Anthrax008~B36R06C15 | 1.00E-05 | Ba~MGC:BA2591~BA698782~RFU:59977.01 |
| Anthrax008~B32R08C15 | 1.02E-05 | Ba~MGC:BA2750~BA15686~RFU:0 |
| Anthrax008~B01R05C19 | 1.06E-05 | Ba~MGC:BA5054~BA698226~RFU:60123.25 |
| Anthrax008~B13R07C03 | 1.21E-05 | Ba~MGC:BA4929~BA698169~RFU:60003.68 |
| Anthrax008~B13R03C05 | 1.38E-05 | Ba~MGC:BA2884~BA697133~RFU:0 |
| Anthrax008~B13R06C03 | 1.39E-05 | Ba~MGC:BA4035~BA697703~RFU:0 |
| Anthrax008~B19R04C16 | 1.57E-05 | Ba~MGC:BA2305~BA696869~RFU:59968.73 |
| Anthrax008~B20R03C11 | 1.59E-05 | Ba~MGC:BA0143.1~BA695807~RFU:9072.96 |
| Anthrax008~B32R02C08 | 1.64E-05 | Ba~MGC:BA2386~BA698779~RFU:1.43 |
| Anthrax008~B40R02C09 | 1.66E-05 | Ba~MGC:BA4769~BA698080~RFU:0 |
| Anthrax008~B48R08C19 | 1.83E-05 | Ba~MGC:BA1929~BA14640~RFU:0 |
| Anthrax008~B32R08C17 | 1.83E-05 | Ba~MGC:BA2401~BA15434~RFU:0 |
| Anthrax008~B24R02C09 | 1.92E-05 | Ba~MGC:BA1242~BA696341~RFU:0 |
| Anthrax008~B14R08C17 | 1.99E-05 | Ba~MGC:BA2707~BA14216~RFU:0 |
| Anthrax008~B47R04C18 | 2.05E-05 | Ba~MGC:BA5076~BA698241~RFU:60071.74 |
| Anthrax008~B28R02C06 | 2.46E-05 | Ba~MGC:BA4121~BA697740~RFU:60056.11 |
| Anthrax008~B38R04C01 | 2.49E-05 | Ba~MGC:BA1897~BA696662~RFU:59977.92 |
| Anthrax008~B13R07C04 | 2.57E-05 | Ba~MGC:BA4929~BA698169~RFU:60007.36 |
| Anthrax008~B44R11C18 | 2.57E-05 | Ba~MGC:BA4182~BA16750~RFU:25233.19 |
| Anthrax008~B24R01C18 | 2.64E-05 | Ba~MGC:BA5458~BA698440~RFU:0 |
| Anthrax008~B19R04C15 | 2.81E-05 | Ba~MGC:BA2305~BA696869~RFU:60035.87 |
| Anthrax008~B48R14C14 | 3.11E-05 | Ba~MGC:BA0007~BA698722~RFU:256.08 |
| Anthrax008~B32R02C10 | 3.15E-05 | Ba~MGC:BA2119~BA696770~RFU:1.43 |
| Anthrax008~B01R05C20 | 3.33E-05 | Ba~MGC:BA5054~BA698226~RFU:60116.81 |
| Anthrax008~B06R18C13 | 3.41E-05 | EF_3.125n~N/A |
| Anthrax008~B08R07C14 | 3.54E-05 | Ba~MGC:BA0107~BA695790~RFU:10.12 |
| Anthrax008~B21R04C12 | 3.55E-05 | Ba~MGC:BA4778~BA698083~RFU:59861.11 |
| Anthrax008~B06R02C07 | 3.77E-05 | Ba~MGC:BA1048~BA696249~RFU:25.75 |
| Anthrax008~B44R11C17 | 3.86E-05 | Ba~MGC:BA4182~BA16750~RFU:29185.98 |
| Anthrax008~B06R18C17 | 4.17E-05 | EF_0.78125n~N/A |
| Anthrax008~B32R08C12 | 4.18E-05 | Ba~MGC:BA2541~BA15044~RFU:2547.93 |
| Anthrax008~B32R02C07 | 4.18E-05 | Ba~MGC:BA2386~BA698779~RFU:0 |
| Anthrax008~B24R02C07 | 4.19E-05 | Ba~MGC:BA1311~BA696374~RFU:0 |
| Anthrax008~B38R01C19 | 4.53E-05 | Ba~MGC:BA1881~BA696656~RFU:0 |
| Anthrax008~B19R07C06 | 4.96E-05 | Ba~MGC:BA3076~BA697235~RFU:60086.46 |
| Anthrax008~B22R01C19 | 5.24E-05 | Ba~MGC:BA3321~BA697332~RFU:0 |
| Anthrax008~B46R02C17 | 5.36E-05 | Ba~MGC:BA3953~BA697653~RFU:54808.68 |
| Anthrax008~B19R05C17 | 5.50E-05 | Ba~MGC:BA5081~BA698243~RFU:60068.98 |
| Anthrax008~B06R18C19 | 5.65E-05 | EF_0.390625n~N/A |
| Anthrax008~B01R02C15 | 5.71E-05 | Ba~MGC:BA4480~BA697934~RFU:0 |
| Anthrax008~B32R02C03 | 5.83E-05 | Ba~MGC:BA2114~BA696768~RFU:0 |
| Anthrax008~B16R08C19 | 5.93E-05 | Ba~MGC:BA2344~BA13830~RFU:60021.16 |
| Anthrax008~B02R03C05 | 6.10E-05 | Ba~MGC:BA1733~BA696583~RFU:0 |
| Anthrax008~B32R08C13 | 6.13E-05 | Ba~MGC:BA2738~BA16084~RFU:0 |
| Anthrax008~B32R01C20 | 6.29E-05 | Ba~MGC:BA3088~BA697240~RFU:0 |
| Anthrax008~B08R08C07 | 6.56E-05 | Ba~MGC:BA2276~BA14756~RFU:0 |
| Anthrax008~B48R01C17 | 6.91E-05 | Ba~MGC:BA1797~BA696613~RFU:0 |
| Anthrax008~B48R08C18 | 6.92E-05 | Ba~MGC:BA1788~BA14066~RFU:13.8 |
| Anthrax008~B46R02C03 | 7.08E-05 | Ba~MGC:BA1013~BA696235~RFU:0 |
| Anthrax008~B24R08C19 | 7.32E-05 | Ba~MGC:BA2217~BA15926~RFU:0 |
| Anthrax008~B30R02C07 | 7.42E-05 | Ba~MGC:BA4850~BA698120~RFU:0 |
| Anthrax008~B30R08C09 | 7.65E-05 | Ba~MGC:BA2611~BA15182~RFU:0 |
| Anthrax008~B48R02C08 | 7.66E-05 | Ba~MGC:BA0639~BA696061~RFU:0 |
| Anthrax008~B15R03C06 | 7.89E-05 | Ba~MGC:BA2917~BA697145~RFU:60106.7 |
| Anthrax008~B20R03C12 | 8.46E-05 | Ba~MGC:BA0143.1~BA695807~RFU:60031.27 |
| Anthrax008~B14R02C05 | 8.66E-05 | Ba~MGC:BA4383~BA697885~RFU:0 |
| Anthrax008~B01R02C17 | 8.67E-05 | Ba~MGC:BA3818~BA697579~RFU:0 |
| Anthrax008~B10R06C06 | 8.77E-05 | Ba~MGC:BA4334~BA697854~RFU:59923.66 |
| Anthrax008~B32R02C09 | 8.89E-05 | Ba~MGC:BA2119~BA696770~RFU:0 |
| Anthrax008~B06R08C09 | 8.97E-05 | Ba~MGC:BA2717~BA14920~RFU:0 |
| Anthrax008~B32R02C06 | 9.58E-05 | Ba~MGC:BA3903~BA697627~RFU:0 |
| Anthrax008~B46R06C19 | 9.86E-05 | Ba~MGC:BA4823~BA698105~RFU:23904.15 |
| Anthrax008~B48R14C13 | 9.97E-05 | Ba~MGC:BA0007~BA698722~RFU:595.14 |
| Anthrax008~B46R02C18 | 1.02E-04 | Ba~MGC:BA3953~BA697653~RFU:54380.98 |
| Anthrax008~B06R02C11 | 1.04E-04 | Ba~MGC:BA3918~BA697634~RFU:0 |
| Anthrax008~B10R06C05 | 1.07E-04 | Ba~MGC:BA4334~BA697854~RFU:59798.57 |
| Anthrax008~B17R05C08 | 1.19E-04 | Ba~MGC:BA1554~BA696488~RFU:59917.22 |
| Anthrax008~B30R08C19 | 1.28E-04 | Ba~MGC:BA2402~BA14206~RFU:0 |
| Anthrax008~B21R04C11 | 1.30E-04 | Ba~MGC:BA4778~BA698083~RFU:60030.35 |
| Anthrax008~B24R08C12 | 1.31E-04 | Ba~MGC:BA2718~BA14642~RFU:0 |
| Anthrax008~B30R02C03 | 1.38E-04 | Ba~MGC:BA2240~BA696835~RFU:0 |
| Anthrax008~B13R06C08 | 1.39E-04 | Ba~MGC:BA3845~BA697596~RFU:50802.06 |
| Anthrax008~B47R02C15 | 1.42E-04 | Ba~MGC:BA2752~BA697068~RFU:0 |
| Anthrax008~B16R04C02 | 1.49E-04 | Ba~MGC:BA3795~BA697568~RFU:60060.71 |
| Anthrax008~B28R03C07 | 1.53E-04 | Ba~MGC:BA3813~BA697576~RFU:59974.25 |
| Anthrax008~B35R08C12 | 1.65E-04 | Ba~MGC:BA1380~BA14512~RFU:291.85 |
| Anthrax008~B40R02C06 | 1.77E-04 | Ba~MGC:BA1044~BA696245~RFU:0 |
| Anthrax008~B40R02C05 | 1.89E-04 | Ba~MGC:BA1044~BA696245~RFU:2.76 |
| Anthrax008~B28R02C05 | 1.93E-04 | Ba~MGC:BA4121~BA697740~RFU:59957.69 |
| Anthrax008~B19R07C08 | 1.98E-04 | Ba~MGC:BA3915~BA697633~RFU:60053.35 |
| Anthrax008~B16R04C01 | 2.03E-04 | Ba~MGC:BA3795~BA697568~RFU:60089.22 |
| Anthrax008~B08R07C13 | 2.07E-04 | Ba~MGC:BA0107~BA695790~RFU:0 |
| Anthrax008~B30R08C12 | 2.12E-04 | Ba~MGC:BA2626~BA14344~RFU:0 |
| Anthrax008~B19R05C18 | 2.25E-04 | Ba~MGC:BA5081~BA698243~RFU:60079.1 |
| Anthrax008~B43R01C18 | 2.31E-04 | Ba~MGC:BA0669~BA696075~RFU:41430.62 |
| Anthrax008~B02R03C13 | 2.33E-04 | Ba~MGC:BA3504~BA697427~RFU:39208.98 |
| Anthrax008~B08R08C13 | 2.34E-04 | Ba~MGC:BA2454~BA15700~RFU:0 |
| Anthrax008~B09R05C04 | 2.54E-04 | Ba~MGC:BA3379~BA697365~RFU:59587.01 |
| Anthrax008~B11R06C14 | 2.56E-04 | Ba~MGC:BA3105~BA697252~RFU:19001.1 |
| Anthrax008~B22R18C01 | 2.66E-04 | PA_200n~N/A |
| Anthrax008~B32R02C04 | 2.83E-04 | Ba~MGC:BA2114~BA696768~RFU:11.96 |
| Anthrax008~B40R02C07 | 2.91E-04 | Ba~MGC:BA1458~BA696442~RFU:0 |
| Anthrax008~B17R05C07 | 2.92E-04 | Ba~MGC:BA1554~BA696488~RFU:59902.5 |
| Anthrax008~B13R05C14 | 2.96E-04 | Ba~MGC:BA0943~BA696210~RFU:58423.47 |
| Anthrax008~B48R08C10 | 3.03E-04 | Ba~MGC:BA2691~BA14402~RFU:7.15 |
| Anthrax008~B10R07C04 | 3.14E-04 | Ba~MGC:BA0280~BA695880~RFU:59841.8 |
| Anthrax008~B08R08C17 | 3.16E-04 | Ba~MGC:BA2287~BA14950~RFU:4802.24 |
| Anthrax008~B16R01C20 | 3.26E-04 | Ba~MGC:BA2960~BA697172~RFU:7.36 |
| Anthrax008~B13R06C07 | 3.29E-04 | Ba~MGC:BA3845~BA697596~RFU:37391.46 |
| Anthrax008~B34R12C11 | 3.31E-04 | Ba~MGC:BA2804~BA13475~RFU:40688.01 |
| Anthrax008~B09R02C15 | 3.34E-04 | Ba~MGC:BA1181~BA696305~RFU:0 |
| Anthrax008~B19R07C05 | 3.41E-04 | Ba~MGC:BA3076~BA697235~RFU:60095.66 |
| Anthrax008~B08R08C18 | 3.80E-04 | Ba~MGC:BA2287~BA14950~RFU:4739.7 |
| Anthrax008~B13R05C16 | 4.00E-04 | Ba~MGC:BA4537~BA697964~RFU:59097.68 |
| Anthrax008~B09R05C06 | 4.00E-04 | Ba~MGC:BA4525~BA697956~RFU:59927.34 |
| Anthrax008~B26R04C06 | 4.08E-04 | Ba~MGC:BA0116~BA695794~RFU:60076.34 |
| Anthrax008~B10R14C18 | 4.13E-04 | Ba~MGC:BA2878~BA15454~RFU:60069.9 |
| Anthrax008~B34R12C16 | 4.19E-04 | Ba~MGC:BXB0086~BA13595~RFU:16061.52 |
| Anthrax008~B35R03C05 | 4.20E-04 | Ba~MGC:BA1994~BA696707~RFU:60029.43 |
| Anthrax008~B19R07C07 | 4.23E-04 | Ba~MGC:BA3915~BA697633~RFU:60052.43 |
| Anthrax008~B35R03C19 | 4.26E-04 | Ba~MGC:BA3764~BA697554~RFU:59870.31 |
| Anthrax008~B45R02C17 | 4.34E-04 | Ba~MGC:BA4886~BA698141~RFU:0 |
| Anthrax008~B13R07C05 | 4.42E-04 | Ba~MGC:BA5584~BA698498~RFU:41192.05 |
| Anthrax008~B24R02C04 | 4.47E-04 | Ba~MGC:BA3580~BA697470~RFU:0 |
| Anthrax008~B34R05C10 | 4.51E-04 | Ba~MGC:BA2236~BA696832~RFU:60093.82 |
| Anthrax008~B35R03C06 | 4.70E-04 | Ba~MGC:BA1994~BA696707~RFU:60004.6 |
| Anthrax008~B31R02C14 | 4.71E-04 | Ba~MGC:BA2318~BA696876~RFU:60050.59 |
| Anthrax008~B01R04C19 | 4.74E-04 | Ba~MGC:BA3490~BA697418~RFU:53469.46 |
| Anthrax008~B03R14C19 | 4.81E-04 | Ba~MGC:BA5467~BA698444~RFU:29990.8 |
| Anthrax008~B30R02C08 | 4.86E-04 | Ba~MGC:BA4850~BA698120~RFU:7.15 |
| Anthrax008~B06R02C08 | 4.87E-04 | Ba~MGC:BA1048~BA696249~RFU:12.88 |
| Anthrax008~B48R08C17 | 4.93E-04 | Ba~MGC:BA1788~BA14066~RFU:0 |
| Anthrax008~B48R02C03 | 4.95E-04 | Ba~MGC:BA0185~BA695835~RFU:0 |
| Anthrax008~B03R03C07 | 4.96E-04 | Ba~MGC:BA1411~BA696418~RFU:60064.39 |
| Anthrax008~B17R06C08 | 4.97E-04 | Ba~MGC:BA0678~BA696079~RFU:35628.22 |
| Anthrax008~B38R04C09 | 5.06E-04 | Ba~MGC:BA3620~BA697486~RFU:2110.01 |
| Anthrax008~B19R02C08 | 5.09E-04 | Ba~MGC:BA5062~BA698231~RFU:60045.07 |
| Anthrax008~B14R06C09 | 5.55E-04 | Ba~MGC:BA4429~BA697908~RFU:60070.82 |
| Anthrax008~B07R14C19 | 5.56E-04 | Ba~MGC:BA4104~BA697734~RFU:60111.3 |
| Anthrax008~B13R07C06 | 5.66E-04 | Ba~MGC:BA5584~BA698498~RFU:38677.34 |
| Anthrax008~B35R03C20 | 5.69E-04 | Ba~MGC:BA3764~BA697554~RFU:59898.82 |
| Anthrax008~B32R01C17 | 5.70E-04 | Ba~MGC:BA3461~BA697403~RFU:15.64 |
| Anthrax008~B09R05C03 | 5.74E-04 | Ba~MGC:BA3379~BA697365~RFU:0 |
| Anthrax008~B14R03C14 | 5.77E-04 | Ba~MGC:BA0259~BA695868~RFU:60005.52 |
| Anthrax008~B13R05C15 | 6.05E-04 | Ba~MGC:BA4537~BA697964~RFU:58757.36 |
| Anthrax008~B34R13C06 | 6.06E-04 | Ba~MGC:BA2644~BA2644~RFU:4700.15 |
| Anthrax008~B47R03C01 | 6.08E-04 | Ba~MGC:BA4825~BA698107~RFU:60057.03 |
| Anthrax008~B47R06C15 | 6.10E-04 | Ba~MGC:BA3513~BA697432~RFU:60108.54 |
| Anthrax008~B40R02C08 | 6.10E-04 | Ba~MGC:BA1458~BA696442~RFU:5.52 |
| Anthrax008~B17R06C07 | 6.11E-04 | Ba~MGC:BA0678~BA696079~RFU:56475.35 |
| Anthrax008~B11R04C15 | 6.25E-04 | Ba~MGC:BA0119~BA695795~RFU:59978.84 |
| Anthrax008~B03R14C20 | 6.34E-04 | Ba~MGC:BA5467~BA698444~RFU:25080.02 |
| Anthrax008~B34R05C09 | 6.35E-04 | Ba~MGC:BA2236~BA696832~RFU:60091.98 |
| Anthrax008~B09R05C05 | 6.36E-04 | Ba~MGC:BA4525~BA697956~RFU:0 |
| Anthrax008~B02R03C14 | 6.38E-04 | Ba~MGC:BA3504~BA697427~RFU:35928.99 |
| Anthrax008~B40R02C11 | 6.42E-04 | Ba~MGC:BA4338~BA697858~RFU:0 |
| Anthrax008~B39R06C20 | 6.67E-04 | Ba~MGC:BA1707~BA696567~RFU:60075.42 |
| Anthrax008~B24R08C15 | 6.67E-04 | Ba~MGC:BA2756~BA15130~RFU:0 |
| Anthrax008~B38R04C03 | 6.73E-04 | Ba~MGC:BA4963~BA698183~RFU:59862.03 |
| Anthrax008~B30R08C15 | 6.88E-04 | Ba~MGC:BA2745~BA14364~RFU:7.36 |
| Anthrax008~B26R03C17 | 6.97E-04 | Ba~MGC:BA1531~BA696476~RFU:59986.2 |
| Anthrax008~B10R06C15 | 6.97E-04 | Ba~MGC:BA4896~BA698150~RFU:22277.41 |
| Anthrax008~B38R08C17 | 7.01E-04 | Ba~MGC:BA2152~BA15662~RFU:0 |
| Anthrax008~B24R08C14 | 7.11E-04 | Ba~MGC:BA2441~BA15578~RFU:19.32 |
| Anthrax008~B14R08C19 | 7.11E-04 | Ba~MGC:BA2716~BA15164~RFU:11462.47 |
| Anthrax008~B48R08C13 | 7.13E-04 | Ba~MGC:BA1580~BA14210~RFU:0 |
| Anthrax008~B26R03C18 | 7.69E-04 | Ba~MGC:BA1531~BA696476~RFU:60058.87 |
| Anthrax008~B19R03C10 | 7.82E-04 | Ba~MGC:BA1820~BA696626~RFU:60061.63 |
| Anthrax008~B17R11C08 | 7.82E-04 | Ba~MGC:BA5115~BA13562~RFU:31201.25 |
| Anthrax008~B10R07C03 | 7.85E-04 | Ba~MGC:BA0280~BA695880~RFU:59875.83 |
| Anthrax008~B36R06C03 | 8.00E-04 | Ba~MGC:BA1093~BA696272~RFU:48055.79 |
| Anthrax008~B10R10C06 | 8.11E-04 | Ba~MGC:BA4124~BA13292~RFU:10150.21 |
| Anthrax008~B08R05C20 | 8.24E-04 | Ba~MGC:BA0030~BA695743~RFU:60084.62 |
| Anthrax008~B06R06C02 | 8.63E-04 | Ba~MGC:BA2412~BA696922~RFU:39087.27 |
| Anthrax008~B46R06C17 | 8.76E-04 | Ba~MGC:BA1829~BA698766~RFU:60108.54 |
| Anthrax008~B46R02C20 | 9.08E-04 | Ba~MGC:BA0113~BA695793~RFU:60046.91 |
| Anthrax008~B14R08C05 | 9.21E-04 | Ba~MGC:BA2357~BA13670~RFU:0 |
| Anthrax008~B26R12C17 | 9.27E-04 | Ba~MGC:BXB0112~BA13579~RFU:36387.97 |
| Anthrax008~B36R04C14 | 9.46E-04 | Ba~MGC:BA4398~BA697893~RFU:59893.3 |
| Anthrax008~B16R01C17 | 9.52E-04 | Ba~MGC:BA3871~BA697606~RFU:0 |
| Anthrax008~B08R05C19 | 0.001007044 | Ba~MGC:BA0030~BA695743~RFU:60048.75 |
| Anthrax008~B11R04C16 | 0.001013878 | Ba~MGC:BA0119~BA695795~RFU:59971.49 |
| Anthrax008~B26R05C12 | 0.001019798 | Ba~MGC:BA1629~BA696530~RFU:44928.47 |
| Anthrax008~B07R14C20 | 0.001023347 | Ba~MGC:BA4104~BA697734~RFU:60123.25 |
| Anthrax008~B03R08C09 | 0.001023877 | Ba~MGC:BA1054~BA14686~RFU:5399.19 |
| Anthrax008~B08R07C11 | 0.001025394 | Ba~MGC:BA2814~BA697100~RFU:0 |
| Anthrax008~B26R04C05 | 0.001043289 | Ba~MGC:BA0116~BA695794~RFU:60056.11 |
| Anthrax008~B40R02C04 | 0.001044157 | Ba~MGC:BA2987~BA697183~RFU:4.29 |
| Anthrax008~B48R02C07 | 0.001044628 | Ba~MGC:BA0639~BA696061~RFU:4.6 |
| Anthrax008~B48R06C04 | 0.001053988 | Ba~MGC:BA1526~BA696475~RFU:26153.08 |
| Anthrax008~B38R02C07 | 0.001059139 | Ba~MGC:BA2795~BA697090~RFU:0 |
| Anthrax008~B34R02C17 | 0.001068834 | Ba~MGC:BA4205~BA697783~RFU:0 |
| Anthrax008~B10R06C16 | 0.001070178 | Ba~MGC:BA4896~BA698150~RFU:22297.57 |
| Anthrax008~B01R04C20 | 0.00109737 | Ba~MGC:BA3490~BA697418~RFU:51710.82 |
| Anthrax008~B14R01C17 | 0.001118315 | Ba~MGC:BA4715~BA698051~RFU:0 |
| Anthrax008~B43R02C18 | 0.001124217 | Ba~MGC:BA1861~BA696647~RFU:60081.86 |
| Anthrax008~B07R03C03 | 0.001138089 | Ba~MGC:BA4536~BA697963~RFU:0 |
| Anthrax008~B10R04C01 | 0.001140313 | Ba~MGC:BA2856~BA697117~RFU:60004.6 |
| Anthrax008~B37R05C02 | 0.001143819 | Ba~MGC:BA1969~BA696698~RFU:60053.35 |
| Anthrax008~B27R01C20 | 0.00115246 | Ba~MGC:BA4890~BA698144~RFU:60068.06 |
| Anthrax008~B14R18C17 | 0.00119719 | LF_0.78125n~N/A |
| Anthrax008~B06R06C08 | 0.001204616 | Ba~MGC:BA0224~BA695852~RFU:25363.38 |
| Anthrax008~B45R03C01 | 0.00123919 | Ba~MGC:BA3842~BA697593~RFU:49942.78 |
| Anthrax008~B30R08C13 | 0.001265331 | Ba~MGC:BA2717~BA14920~RFU:2.86 |
| Anthrax008~B06R08C19 | 0.001266341 | Ba~MGC:BA2311~BA13470~RFU:0 |
| Anthrax008~B06R08C17 | 0.00131238 | Ba~MGC:BA2563~BA13218~RFU:0 |
| Anthrax008~B14R03C13 | 0.001313109 | Ba~MGC:BA0259~BA695868~RFU:60032.19 |
| Anthrax008~B03R06C16 | 0.001320027 | Ba~MGC:BA1482~BA696452~RFU:20244.67 |
| Anthrax008~B47R03C07 | 0.001331187 | Ba~MGC:BA0324~BA695907~RFU:0 |
| Anthrax008~B40R05C18 | 0.001353691 | Ba~MGC:BA0125~BA695797~RFU:60043.23 |
| Anthrax008~B27R01C19 | 0.001381215 | Ba~MGC:BA4890~BA698144~RFU:60057.95 |
| Anthrax008~B39R06C19 | 0.001395636 | Ba~MGC:BA1707~BA696567~RFU:0 |
| Anthrax008~B14R02C11 | 0.001449285 | Ba~MGC:BA1877~BA696654~RFU:34.33 |
| Anthrax008~B16R12C18 | 0.001459741 | Ba~MGC:BA0808~BA15883~RFU:18887.97 |
| Anthrax008~B02R02C07 | 0.001477362 | Ba~MGC:BA4692~BA698039~RFU:6424.76 |
| Anthrax008~B10R14C17 | 0.001504455 | Ba~MGC:BA2878~BA15454~RFU:60045.99 |
| Anthrax008~B06R06C07 | 0.001514816 | Ba~MGC:BA0224~BA695852~RFU:48825.46 |
| Anthrax008~B42R04C14 | 0.001515942 | Ba~MGC:BA0733~BA696108~RFU:60058.87 |
| Anthrax008~B19R05C13 | 0.001534297 | Ba~MGC:BA3819~BA697580~RFU:60058.87 |
| Anthrax008~B10R04C02 | 0.001540768 | Ba~MGC:BA2856~BA697117~RFU:59950.33 |
| Anthrax008~B36R03C11 | 0.001555544 | Ba~MGC:BA0101~BA695788~RFU:53407.73 |
| Anthrax008~B40R04C02 | 0.001588664 | Ba~MGC:BA5214~BA698314~RFU:59964.13 |
| Anthrax008~B24R08C11 | 0.001607823 | Ba~MGC:BA2718~BA14642~RFU:.92 |
| Anthrax008~B46R08C15 | 0.00160999 | Ba~MGC:BA1571~BA14664~RFU:0 |
| Anthrax008~B47R03C02 | 0.001646312 | Ba~MGC:BA4825~BA698107~RFU:60025.75 |
| Anthrax008~B04R06C09 | 0.001671717 | Ba~MGC:BA0842~BA696159~RFU:59946.65 |
| Anthrax008~B08R08C19 | 0.001681898 | Ba~MGC:BA2342~BA14170~RFU:24840.88 |
| Anthrax008~B11R06C13 | 0.001702614 | Ba~MGC:BA3105~BA697252~RFU:22154.51 |
| Anthrax008~B39R02C16 | 0.001704684 | Ba~MGC:BA1563~BA696496~RFU:0 |
| Anthrax008~B14R18C07 | 0.001721434 | LF_25n~N/A |
| Anthrax008~B32R08C19 | 0.001721642 | Ba~MGC:BA2440~BA15228~RFU:0 |
| Anthrax008~B14R18C01 | 0.001745139 | LF_200n~N/A |
| Anthrax008~B16R12C17 | 0.001755094 | Ba~MGC:BA0808~BA15883~RFU:17473.33 |
| Anthrax008~B06R18C01 | 0.001770459 | EF_200n~N/A |
| Anthrax008~B40R05C17 | 0.001807096 | Ba~MGC:BA0125~BA695797~RFU:60022.99 |
| Anthrax008~B37R02C17 | 0.001809143 | Ba~MGC:BA3828~BA697587~RFU:0 |
| Anthrax008~B22R18C05 | 0.001831652 | PA_50n~N/A |
| Anthrax008~B22R05C07 | 0.00183541 | Ba~MGC:BA2880~BA697129~RFU:60039.55 |
| Anthrax008~B47R02C20 | 0.001839114 | Ba~MGC:BA4270~BA697814~RFU:60119.57 |
| Anthrax008~B14R18C05 | 0.001841026 | LF_50n~N/A |
| Anthrax008~B35R06C08 | 0.001848328 | Ba~MGC:BA2551~BA696977~RFU:60076.34 |
| Anthrax008~B13R06C14 | 0.001855091 | Ba~MGC:BA5527~BA698827~RFU:44553.65 |
| Anthrax008~B19R05C14 | 0.001863977 | Ba~MGC:BA3819~BA697580~RFU:60069.9 |
| Anthrax008~B16R04C04 | 0.001873577 | Ba~MGC:BA3555~BA697455~RFU:59896.06 |
| Anthrax008~B06R08C16 | 0.001888016 | Ba~MGC:BA2716~BA15164~RFU:0 |
| Anthrax008~B11R04C01 | 0.001911146 | Ba~MGC:BA3761~BA697553~RFU:60024.83 |
| Anthrax008~B19R03C13 | 0.001914718 | Ba~MGC:BA1721~BA696574~RFU:60053.35 |
| Anthrax008~B22R18C13 | 0.001945441 | PA_3.125n~N/A |
| Anthrax008~B45R05C06 | 0.001964907 | Ba~MGC:BA2021~BA696721~RFU:60045.07 |
| Anthrax008~B40R08C09 | 0.001970413 | Ba~MGC:BA2148~BA14864~RFU:0 |
| Anthrax008~B22R06C04 | 0.001970428 | Ba~MGC:BA2224~BA696823~RFU:60068.06 |
| Anthrax008~B03R15C06 | 0.002000414 | Ba~MGC:BA5407~BA698413~RFU:59883.19 |
| Anthrax008~B17R05C03 | 0.002001211 | Ba~MGC:BA2053~BA696739~RFU:59744.3 |
| Anthrax008~B06R08C15 | 0.002014063 | Ba~MGC:BA2716~BA15164~RFU:3.68 |
| Anthrax008~B40R04C01 | 0.002024661 | Ba~MGC:BA5214~BA698314~RFU:60031.27 |
| Anthrax008~B35R12C18 | 0.002043982 | Ba~MGC:BA5442~BA5442~RFU:90.14 |
| Anthrax008~B40R08C19 | 0.002049354 | Ba~MGC:BA2448~BA14908~RFU:0 |
| Anthrax008~B34R12C18 | 0.002076931 | Ba~MGC:BA3948~BA3948~RFU:19295.44 |
| Anthrax008~B26R05C11 | 0.002103941 | Ba~MGC:BA1629~BA696530~RFU:60098.42 |
| Anthrax008~B14R08C07 | 0.002117141 | Ba~MGC:BA2555~BA14438~RFU:0 |
| Anthrax008~B28R02C11 | 0.002121914 | Ba~MGC:BA3336~BA697340~RFU:59982.52 |
| Anthrax008~B14R18C03 | 0.002124399 | LF_100n~N/A |
| Anthrax008~B28R04C16 | 0.002191762 | Ba~MGC:BA0366~BA695932~RFU:60043.23 |
| Anthrax008~B27R11C08 | 0.002278246 | Ba~MGC:BA5077~BA16056~RFU:2026.31 |
| Anthrax008~B13R11C08 | 0.002291499 | Ba~MGC:BA5269~BA15278~RFU:1283.11 |
| Anthrax008~B03R03C08 | 0.002324332 | Ba~MGC:BA1411~BA696418~RFU:60070.82 |
| Anthrax008~B27R02C03 | 0.00232669 | Ba~MGC:BA0060~BA695759~RFU:57568.98 |
| Anthrax008~B45R06C11 | 0.002330664 | Ba~MGC:BA3605~BA697480~RFU:59988.96 |
| Anthrax008~B02R06C09 | 0.00233912 | Ba~MGC:BA2509~BA696962~RFU:60088.3 |
| Anthrax008~B40R02C03 | 0.002343178 | Ba~MGC:BA2987~BA697183~RFU:0 |
| Anthrax008~B36R03C12 | 0.002351721 | Ba~MGC:BA0101~BA695788~RFU:17557.94 |
| Anthrax008~B26R12C18 | 0.002357073 | Ba~MGC:BXB0112~BA13579~RFU:35964.86 |
| Anthrax008~B14R06C10 | 0.00243286 | Ba~MGC:BA4429~BA697908~RFU:60084.62 |
| Anthrax008~B35R12C17 | 0.002468385 | Ba~MGC:BA5442~BA5442~RFU:80.94 |
| Anthrax008~B07R03C19 | 0.002517445 | Ba~MGC:BA5648~BA698529~RFU:60090.14 |
| Anthrax008~B46R08C07 | 0.002536969 | Ba~MGC:BA2187~BA14758~RFU:0 |
| Anthrax008~B46R02C19 | 0.002582389 | Ba~MGC:BA0113~BA695793~RFU:59969.65 |
| Anthrax008~B06R05C19 | 0.002584174 | Ba~MGC:BA4061~BA697717~RFU:60042.31 |
| Anthrax008~B35R07C04 | 0.002596105 | Ba~MGC:BA4051~BA697710~RFU:60110.38 |
| Anthrax008~B42R04C13 | 0.00259825 | Ba~MGC:BA0733~BA696108~RFU:60073.58 |
| Anthrax008~B34R13C08 | 0.002612271 | Ba~MGC:BA4761~BA4761~RFU:23427.15 |
| Anthrax008~B46R08C05 | 0.00263977 | Ba~MGC:BA2389~BA15040~RFU:8.28 |
| Anthrax008~B13R06C13 | 0.00264453 | Ba~MGC:BA5527~BA698827~RFU:43010.01 |
| Anthrax008~B11R04C14 | 0.002700858 | Ba~MGC:BA0038.1~BA695747~RFU:60033.11 |
| Anthrax008~B36R04C13 | 0.002726799 | Ba~MGC:BA4398~BA697893~RFU:59904.34 |
| Anthrax008~B35R05C01 | 0.002732266 | Ba~MGC:BA1837~BA696637~RFU:59951.25 |
| Anthrax008~B17R05C04 | 0.002735085 | Ba~MGC:BA2053~BA696739~RFU:59902.5 |
| Anthrax008~B07R03C20 | 0.002740475 | Ba~MGC:BA5648~BA698529~RFU:60111.3 |
| Anthrax008~B35R14C12 | 0.002787742 | Ba~MGC:BA1674~BA696554~RFU:0 |
| Anthrax008~B01R07C03 | 0.002814494 | Ba~MGC:BA5248~BA698328~RFU:60097.5 |
| Anthrax008~B08R01C19 | 0.002826467 | Ba~MGC:BA5096~BA698252~RFU:0 |
| Anthrax008~B11R08C20 | 0.002889684 | Ba~MGC:BA1258~BA16082~RFU:1915.93 |
| Anthrax008~B14R05C04 | 0.002890532 | Ba~MGC:BA3560~BA697458~RFU:54722.22 |
| Anthrax008~B45R05C05 | 0.002920668 | Ba~MGC:BA2021~BA696721~RFU:60049.67 |
| Anthrax008~B45R06C12 | 0.002924637 | Ba~MGC:BA3605~BA697480~RFU:60015.64 |
| Anthrax008~B22R13C20 | 0.002937652 | Ba~MGC:BA5281~BA13591~RFU:60102.1 |
| Anthrax008~B12R12C07 | 0.00297067 | Ba~MGC:BA3694~BA13523~RFU:56933.41 |
| Anthrax008~B10R04C10 | 0.002983622 | Ba~MGC:BA2931~BA697152~RFU:59951.25 |
| Anthrax008~B23R05C08 | 0.002996786 | Ba~MGC:BA3842~BA697593~RFU:59890.54 |
| Anthrax008~B06R05C20 | 0.003048431 | Ba~MGC:BA4061~BA697717~RFU:60035.87 |
| Anthrax008~B43R14C11 | 0.003061012 | Ba~MGC:BA5637~BA698522~RFU:0 |
| Anthrax008~B22R08C07 | 0.00310081 | Ba~MGC:BA2353~BA15830~RFU:0 |
| Anthrax008~B02R02C08 | 0.003103419 | Ba~MGC:BA4692~BA698039~RFU:6000.74 |
| Anthrax008~B38R02C09 | 0.003115486 | Ba~MGC:BA1329~BA696384~RFU:0 |
| Anthrax008~B18R13C18 | 0.003124921 | Ba~MGC:BA3905~BA3905~RFU:60124.17 |
| Anthrax008~B01R03C03 | 0.003131329 | Ba~MGC:BA2841~BA697110~RFU:60125.09 |
| Anthrax008~B11R04C02 | 0.003190731 | Ba~MGC:BA3761~BA697553~RFU:60008.28 |
| Anthrax008~B06R06C01 | 0.00321762 | Ba~MGC:BA2412~BA696922~RFU:32964.5 |
| Anthrax008~B04R05C10 | 0.003252216 | Ba~MGC:BA5142~BA698274~RFU:59982.52 |
| Anthrax008~B35R05C02 | 0.003302852 | Ba~MGC:BA1837~BA696637~RFU:60025.75 |
| Anthrax008~B04R06C10 | 0.003318196 | Ba~MGC:BA0842~BA696159~RFU:59986.2 |
| Anthrax008~B25R08C16 | 0.003329502 | Ba~MGC:BA1463~BA15132~RFU:9004.29 |
| Anthrax008~B09R07C03 | 0.003345077 | Ba~MGC:BA1073~BA696261~RFU:4496.87 |
| Anthrax008~B37R05C01 | 0.003414595 | Ba~MGC:BA1969~BA696698~RFU:60074.5 |
| Anthrax008~B43R05C15 | 0.003435913 | Ba~MGC:BA1370~BA696402~RFU:60037.71 |
| Anthrax008~B08R06C11 | 0.003436413 | Ba~MGC:BA0376~BA695938~RFU:60117.73 |
| Anthrax008~B48R13C14 | 0.003449196 | Ba~MGC:BA4933~BA4933~RFU:1121.6 |
| Anthrax008~B11R06C06 | 0.003450322 | Ba~MGC:BA3754~BA697551~RFU:6959.94 |
| Anthrax008~B40R01C19 | 0.003464619 | Ba~MGC:BA1502~BA696462~RFU:11.44 |
| Anthrax008~B28R02C12 | 0.003469748 | Ba~MGC:BA3336~BA697340~RFU:60019.32 |
| Anthrax008~B25R05C01 | 0.003500409 | Ba~MGC:BA4501~BA697944~RFU:48716.74 |
| Anthrax008~B14R05C03 | 0.003547213 | Ba~MGC:BA3560~BA697458~RFU:59675.31 |
| Anthrax008~B27R04C19 | 0.003570221 | Ba~MGC:BA5718~BA698567~RFU:60029.43 |
| Anthrax008~B33R05C20 | 0.003596973 | Ba~MGC:BA0296~BA695887~RFU:.92 |
| Anthrax008~B47R06C16 | 0.003611461 | Ba~MGC:BA3513~BA697432~RFU:60111.3 |
| Anthrax008~B46R05C13 | 0.003624372 | Ba~MGC:BA1891~BA696659~RFU:60054.27 |
| Anthrax008~B43R01C17 | 0.003644068 | Ba~MGC:BA0669~BA696075~RFU:40776.82 |
| Anthrax008~B32R13C12 | 0.003733334 | Ba~MGC:BA4399~BA4399~RFU:635.58 |
| Anthrax008~B34R04C20 | 0.00374611 | Ba~MGC:BA3458~BA697402~RFU:60055.19 |
| Anthrax008~B35R07C03 | 0.003749722 | Ba~MGC:BA4051~BA697710~RFU:60088.3 |
| Anthrax008~B14R18C19 | 0.003762046 | LF_0.390625n~N/A |
| Anthrax008~B02R10C11 | 0.003840683 | Ba~MGC:BA4500~BA15484~RFU:2075.97 |
| Anthrax008~B25R05C02 | 0.003851544 | Ba~MGC:BA4501~BA697944~RFU:54045.78 |
| Anthrax008~B12R07C06 | 0.003862565 | Ba~MGC:BA1323~BA696383~RFU:60094.74 |
| Anthrax008~B35R03C18 | 0.003887266 | Ba~MGC:BA2459~BA696947~RFU:51363.13 |
| Anthrax008~B09R02C04 | 0.00389151 | Ba~MGC:BA0197~BA695841~RFU:60091.06 |
| Anthrax008~B14R06C06 | 0.003906703 | Ba~MGC:BA1949~BA696689~RFU:60005.52 |
| Anthrax008~B42R06C07 | 0.003910672 | Ba~MGC:BA3985~BA697675~RFU:0 |
| Anthrax008~B12R02C17 | 0.003915993 | Ba~MGC:BA0010~BA695728~RFU:59988.96 |
| Anthrax008~B38R05C09 | 0.003945754 | Ba~MGC:BA1472~BA696447~RFU:60038.63 |
| Anthrax008~B14R06C05 | 0.003955499 | Ba~MGC:BA1949~BA696689~RFU:59977.01 |
| Anthrax008~B22R08C09 | 0.003983812 | Ba~MGC:BA2431~BA13438~RFU:0 |
| Anthrax008~B38R05C20 | 0.003987229 | Ba~MGC:BA0073~BA695767~RFU:60102.1 |
| Anthrax008~B39R12C17 | 0.004103751 | Ba~MGC:BA3522~BA13214~RFU:28101.55 |
| Anthrax008~B19R03C09 | 0.004119256 | Ba~MGC:BA1820~BA696626~RFU:60067.14 |
| Anthrax008~B39R07C11 | 0.004136719 | Ba~MGC:BA5367~BA698392~RFU:1015.45 |
| Anthrax008~B08R03C20 | 0.004169385 | Ba~MGC:BA5350~BA698379~RFU:60071.74 |
| Anthrax008~B18R10C05 | 0.004228853 | Ba~MGC:BA4120~BA15438~RFU:0 |
| Anthrax008~B44R06C07 | 0.004270237 | Ba~MGC:BA0760~BA696120~RFU:0 |
| Anthrax008~B27R05C06 | 0.004280856 | Ba~MGC:BA1138~BA696290~RFU:60036.79 |
| Anthrax008~B26R12C01 | 0.004324394 | Ba~MGC:BA4677~BA16600~RFU:1204.01 |
| Anthrax008~B19R05C02 | 0.004456576 | Ba~MGC:BA0386~BA695946~RFU:60083.7 |
| Anthrax008~B46R08C10 | 0.004529309 | Ba~MGC:BA2668~BA16150~RFU:8.28 |
| Anthrax008~B31R02C13 | 0.004618873 | Ba~MGC:BA2318~BA696876~RFU:60072.66 |
| Anthrax008~B13R05C19 | 0.004630422 | Ba~MGC:BA5551~BA698480~RFU:59918.14 |
| Anthrax008~B43R15C04 | 0.004642567 | Ba~MGC:BA0958~BA14302~RFU:60142.57 |
| Anthrax008~B12R05C07 | 0.004657116 | Ba~MGC:BA0644~BA696063~RFU:47228.9 |
| Anthrax008~B12R02C18 | 0.004658672 | Ba~MGC:BA0010~BA695728~RFU:59961.37 |
| Anthrax008~B30R01C17 | 0.004730011 | Ba~MGC:BA0216~BA695848~RFU:0 |
| Anthrax008~B35R08C11 | 0.004779376 | Ba~MGC:BA1380~BA14512~RFU:213.16 |
| Anthrax008~B17R08C09 | 0.004827058 | Ba~MGC:BA1284~BA13456~RFU:23719.6 |
| Anthrax008~B38R08C09 | 0.004834516 | Ba~MGC:BA2376~BA14746~RFU:0 |
| Anthrax008~B35R15C08 | 0.00483529 | Ba~MGC:BA2808~BA697095~RFU:24611.85 |
| Anthrax008~B14R05C05 | 0.004880588 | Ba~MGC:BA5452~BA698438~RFU:59654.16 |
| Anthrax008~B03R08C18 | 0.004889424 | Ba~MGC:BA1406~BA14404~RFU:7791.13 |
| Anthrax008~B02R07C05 | 0.004921975 | Ba~MGC:BA2175~BA696798~RFU:33755.52 |
| Anthrax008~B16R04C03 | 0.004973537 | Ba~MGC:BA3555~BA697455~RFU:60042.31 |
| Anthrax008~B04R05C09 | 0.004976862 | Ba~MGC:BA5142~BA698274~RFU:59931.94 |
| Anthrax008~B39R02C15 | 0.005002196 | Ba~MGC:BA1563~BA696496~RFU:17.17 |
| Anthrax008~B46R08C09 | 0.005090879 | Ba~MGC:BA2668~BA16150~RFU:0 |
| Anthrax008~B36R05C15 | 0.005217128 | Ba~MGC:BA0111~BA695792~RFU:59981.6 |
| Anthrax008~B45R03C03 | 0.005233602 | Ba~MGC:BA3990~BA697676~RFU:0 |
| Anthrax008~B38R08C18 | 0.005259393 | Ba~MGC:BA2152~BA15662~RFU:17.48 |
| Anthrax008~B02R06C10 | 0.005267375 | Ba~MGC:BA2509~BA696962~RFU:60094.74 |
| Anthrax008~B48R13C09 | 0.005355016 | Ba~MGC:BA5327~BA15265~RFU:250.36 |
| Anthrax008~B44R06C08 | 0.005391025 | Ba~MGC:BA0760~BA696120~RFU:59971.49 |
| Anthrax008~B39R06C08 | 0.005403968 | Ba~MGC:BA1195~BA696314~RFU:60104.86 |
| Anthrax008~B01R07C04 | 0.005436772 | Ba~MGC:BA5248~BA698328~RFU:60114.05 |
| Anthrax008~B02R04C15 | 0.005495952 | Ba~MGC:BA1170~BA696303~RFU:60090.14 |
| Anthrax008~B35R16C16 | 0.005543116 | Ba~MGC:BA4953~BA17395~RFU:19850.99 |
| Anthrax008~B42R03C13 | 0.005583174 | Ba~MGC:BA3654~BA697501~RFU:60005.52 |
| Anthrax008~B37R03C01 | 0.005661097 | Ba~MGC:BA3303~BA697325~RFU:108.73 |
| Anthrax008~B21R06C14 | 0.005690443 | Ba~MGC:BA4867~BA698130~RFU:60075.42 |
| Anthrax008~B28R10C04 | 0.005692394 | Ba~MGC:BA4310~BA14784~RFU:2983.81 |
| Anthrax008~B45R03C02 | 0.005766544 | Ba~MGC:BA3842~BA697593~RFU:25666.67 |
| Anthrax008~B24R01C17 | 0.005812937 | Ba~MGC:BA5458~BA698440~RFU:5.52 |
| Anthrax008~B34R12C14 | 0.005913954 | Ba~MGC:BXB0075~BA14189~RFU:15527.9 |
| Anthrax008~B35R06C07 | 0.005963518 | Ba~MGC:BA2551~BA696977~RFU:60070.82 |
| Anthrax008~B35R10C16 | 0.005984999 | Ba~MGC:BXA0101~BA16174~RFU:963.02 |
| Anthrax008~B33R05C19 | 0.005998733 | Ba~MGC:BA0296~BA695887~RFU:0 |
| Anthrax008~B22R13C19 | 0.006014163 | Ba~MGC:BA5281~BA13591~RFU:60093.82 |
| Anthrax008~B12R02C19 | 0.006021614 | Ba~MGC:BA0331~BA695912~RFU:59991.72 |
| Anthrax008~B16R09C17 | 0.006117443 | Ba~MGC:BA4971~BA13890~RFU:3117.18 |
| Anthrax008~B44R08C17 | 0.006128544 | Ba~MGC:BA0957~BA14220~RFU:32018.6 |
| Anthrax008~B38R11C19 | 0.006151652 | Ba~MGC:BA0134~BA12564~RFU:16967.1 |
| Anthrax008~B41R02C09 | 0.006249772 | Ba~MGC:BA4759~BA698073~RFU:60048.75 |
| Anthrax008~B30R02C11 | 0.006374701 | Ba~MGC:BA0170~BA695825~RFU:0 |
| Anthrax008~B21R15C04 | 0.006383679 | Ba~MGC:BA3844~BA697595~RFU:2366.24 |
| Anthrax008~B46R01C17 | 0.006419383 | Ba~MGC:BA0749~BA698739~RFU:0 |
| Anthrax008~B03R02C06 | 0.006488435 | Ba~MGC:BA0836~BA696157~RFU:60077.26 |
| Anthrax008~B34R04C16 | 0.006580814 | Ba~MGC:BA2569~BA696986~RFU:59707.51 |
| Anthrax008~B40R05C08 | 0.006629437 | Ba~MGC:BA1564~BA696497~RFU:60015.64 |
| Anthrax008~B35R04C15 | 0.006650644 | Ba~MGC:BA5569~BA698488~RFU:58593.64 |
| Anthrax008~B34R12C06 | 0.006666292 | Ba~MGC:BA2525~BA13449~RFU:3186.17 |
| Anthrax008~B35R15C01 | 0.006669685 | Ba~MGC:BA5537~BA698474~RFU:57526.67 |
| Anthrax008~B38R08C15 | 0.006770507 | Ba~MGC:BA1623~BA14238~RFU:0 |
| Anthrax008~B12R07C05 | 0.006772768 | Ba~MGC:BA1323~BA696383~RFU:60071.74 |
| Anthrax008~B12R10C13 | 0.006777207 | Ba~MGC:BXA0188~BA13750~RFU:6674.03 |
| Anthrax008~B36R05C19 | 0.006842706 | Ba~MGC:BA2145~BA696784~RFU:59972.41 |
| Anthrax008~B28R04C15 | 0.00685308 | Ba~MGC:BA0366~BA695932~RFU:60004.6 |
| Anthrax008~B06R02C05 | 0.006942605 | Ba~MGC:BA3358~BA697353~RFU:0 |
| Anthrax008~B27R04C20 | 0.006978515 | Ba~MGC:BA5718~BA698567~RFU:60042.31 |
| Anthrax008~B24R02C03 | 0.007021103 | Ba~MGC:BA3580~BA697470~RFU:27.18 |
| Anthrax008~B35R15C06 | 0.007052521 | Ba~MGC:BA5566~BA698487~RFU:34958.61 |
| Anthrax008~B10R03C14 | 0.00713171 | Ba~MGC:BA0032~BA695744~RFU:60017.48 |
| Anthrax008~B27R02C04 | 0.00714692 | Ba~MGC:BA0060~BA695759~RFU:6933.41 |
| Anthrax008~B38R08C11 | 0.007190332 | Ba~MGC:BA2384~BA13716~RFU:0 |
| Anthrax008~B29R12C20 | 0.007211641 | Ba~MGC:BA3810~BA3810~RFU:1877.3 |
| Anthrax008~B46R06C02 | 0.007258681 | Ba~MGC:BA4394.1~BA697891~RFU:60037.71 |
| Anthrax008~B02R04C16 | 0.007273943 | Ba~MGC:BA1170~BA696303~RFU:60091.98 |
| Anthrax008~B03R03C17 | 0.007275405 | Ba~MGC:BA2377~BA696904~RFU:60080.94 |
| Anthrax008~B40R08C05 | 0.007310726 | Ba~MGC:BA2178~BA16164~RFU:0 |
| Anthrax008~B01R14C14 | 0.0074199 | Ba~MGC:BA4424~BA14350~RFU:54093.08 |
| Anthrax008~B13R05C20 | 0.007431936 | Ba~MGC:BA5551~BA698480~RFU:59901.58 |
| Anthrax008~B46R05C14 | 0.007452707 | Ba~MGC:BA1891~BA696659~RFU:60086.46 |
| Anthrax008~B17R11C07 | 0.007467348 | Ba~MGC:BA5115~BA13562~RFU:28595.47 |
| Anthrax008~B34R06C19 | 0.007480513 | Ba~MGC:BA3422~BA697387~RFU:28450.15 |
| Anthrax008~B34R12C20 | 0.00750991 | Ba~MGC:BA1851~BA13495~RFU:36729.61 |
| Anthrax008~B15R06C16 | 0.007537079 | Ba~MGC:BA1213~BA696321~RFU:60099.34 |
| Anthrax008~B31R06C06 | 0.007573515 | Ba~MGC:BA0684~BA696081~RFU:60052.43 |
| Anthrax008~B31R06C05 | 0.007599862 | Ba~MGC:BA0684~BA696081~RFU:60047.83 |
| Anthrax008~B35R07C08 | 0.007650141 | Ba~MGC:BA5120~BA698263~RFU:5694.44 |
| Anthrax008~B41R03C06 | 0.007671195 | Ba~MGC:BA4815~BA698100~RFU:60030.35 |
| Anthrax008~B35R15C03 | 0.007706956 | Ba~MGC:BA4840~BA14142~RFU:27525.75 |
| Anthrax008~B18R03C20 | 0.007718015 | Ba~MGC:BA0517~BA696006~RFU:60043.23 |
| Anthrax008~B27R11C05 | 0.007719838 | Ba~MGC:BA5290~BA14230~RFU:21699.57 |
| Anthrax008~B04R03C12 | 0.007756622 | Ba~MGC:BA3238~BA697300~RFU:60010.12 |
| Anthrax008~B38R05C19 | 0.007847616 | Ba~MGC:BA0073~BA695767~RFU:60081.86 |
| Anthrax008~B12R05C13 | 0.007939437 | Ba~MGC:BA0594~BA696039~RFU:59916.3 |
| Anthrax008~B15R15C03 | 0.007982559 | Ba~MGC:BA5255~BA698333~RFU:1949.04 |
| Anthrax008~B34R02C01 | 0.007994976 | Ba~MGC:BA1429~BA696429~RFU:60096.58 |
| Anthrax008~B04R03C11 | 0.008013982 | Ba~MGC:BA3238~BA697300~RFU:60009.2 |
| Anthrax008~B37R14C15 | 0.008020878 | Ba~MGC:BA3036~BA13665~RFU:15184.55 |
| Anthrax008~B11R08C19 | 0.008048893 | Ba~MGC:BA1258~BA16082~RFU:1697.94 |
| Anthrax008~B32R08C09 | 0.008069241 | Ba~MGC:BA2612~BA14310~RFU:0 |
| Anthrax008~B38R01C17 | 0.008079635 | Ba~MGC:BA1569~BA696501~RFU:37.71 |
| Anthrax008~B12R05C16 | 0.008100809 | Ba~MGC:BA3491~BA697419~RFU:60045.07 |
| Anthrax008~B22R18C03 | 0.008100876 | PA_100n~N/A |
| Anthrax008~B38R04C10 | 0.008138784 | Ba~MGC:BA3620~BA697486~RFU:60047.83 |
| Anthrax008~B44R08C18 | 0.008241587 | Ba~MGC:BA0957~BA14220~RFU:32220.31 |
| Anthrax008~B02R05C04 | 0.00841713 | Ba~MGC:BA1469~BA696446~RFU:3095.85 |
| Anthrax008~B34R12C09 | 0.008486942 | Ba~MGC:BA4649~BA13315~RFU:21499.26 |
| Anthrax008~B22R02C19 | 0.008509147 | Ba~MGC:BA0731~BA696106~RFU:59756.25 |
| Anthrax008~B13R05C13 | 0.008521573 | Ba~MGC:BA0943~BA696210~RFU:46536.06 |
| Anthrax008~B27R07C05 | 0.008615763 | Ba~MGC:BA5298~BA698353~RFU:12893.67 |
| Anthrax008~B15R04C06 | 0.008619195 | Ba~MGC:BA4750~BA698066~RFU:60090.14 |
| Anthrax008~B40R04C17 | 0.008621806 | Ba~MGC:BA4612~BA698002~RFU:59985.28 |
| Anthrax008~B10R04C09 | 0.008753081 | Ba~MGC:BA2931~BA697152~RFU:59927.34 |
| Anthrax008~B21R11C14 | 0.008783375 | Ba~MGC:BA2196~BA16432~RFU:17327.61 |
| Anthrax008~B34R12C08 | 0.008807385 | Ba~MGC:BA3659~BA3659~RFU:25648.45 |
| Anthrax008~B48R07C18 | 0.008836694 | Ba~MGC:BA3373~BA697364~RFU:1482.12 |
| Anthrax008~B01R14C13 | 0.008904105 | Ba~MGC:BA4424~BA14350~RFU:60088.3 |
| Anthrax008~B43R15C03 | 0.008923441 | Ba~MGC:BA0958~BA14302~RFU:60127.85 |
| Anthrax008~B34R13C03 | 0.008926593 | Ba~MGC:BA5051~BA5051~RFU:2767.66 |
| Anthrax008~B48R06C03 | 0.008927304 | Ba~MGC:BA1526~BA696475~RFU:0 |
| Anthrax008~B48R08C09 | 0.008984754 | Ba~MGC:BA2691~BA14402~RFU:2.76 |
| Anthrax008~B33R08C14 | 0.008992196 | Ba~MGC:BA1084~BA14368~RFU:2107.3 |
| Anthrax008~B03R08C10 | 0.009025132 | Ba~MGC:BA1054~BA14686~RFU:5529.8 |
| Anthrax008~B37R06C08 | 0.009103679 | Ba~MGC:BA4148~BA697754~RFU:2582.78 |
| Anthrax008~B22R06C03 | 0.009141613 | Ba~MGC:BA2224~BA696823~RFU:60049.67 |
| Anthrax008~B34R13C04 | 0.009147081 | Ba~MGC:BA5051~BA5051~RFU:2511.04 |
| Anthrax008~B41R03C15 | 0.009205353 | Ba~MGC:BA1573~BA696503~RFU:59946.65 |
| Anthrax008~B34R09C20 | 0.009262743 | Ba~MGC:BA4246~BA15458~RFU:2034.33 |
| Anthrax008~B37R02C15 | 0.009316296 | Ba~MGC:BA2122~BA696772~RFU:0 |
| Anthrax008~B11R06C16 | 0.009359703 | Ba~MGC:BA2354~BA696895~RFU:2833.89 |
| Anthrax008~B46R08C08 | 0.009387938 | Ba~MGC:BA2187~BA14758~RFU:6.44 |
| Anthrax008~B39R02C03 | 0.009433609 | Ba~MGC:BA0205~BA695845~RFU:60036.79 |
| Anthrax008~B39R02C19 | 0.009532355 | Ba~MGC:BA1932~BA696681~RFU:60027.59 |
| Anthrax008~B14R18C11 | 0.009646434 | LF_6.25n~N/A |
| Anthrax008~B30R08C16 | 0.009751371 | Ba~MGC:BA2745~BA14364~RFU:0 |
| Anthrax008~B30R13C10 | 0.009792053 | Ba~MGC:BA4672~BA4672~RFU:403.79 |
| Anthrax008~B10R04C14 | 0.009816027 | Ba~MGC:BA0715~BA696096~RFU:0 |
| Anthrax008~B15R05C05 | 0.009819086 | Ba~MGC:BA5614~BA698509~RFU:54459.23 |
| Anthrax008~B38R04C04 | 0.009901611 | Ba~MGC:BA4963~BA698183~RFU:22895.57 |
| Anthrax008~B19R08C16 | 0.009908559 | Ba~MGC:BA0986~BA14300~RFU:20819.74 |
| Anthrax008~B34R10C03 | 0.009920348 | Ba~MGC:BA4701~BA14946~RFU:2677.52 |
| Anthrax008~B27R11C07 | 0.009922973 | Ba~MGC:BA5077~BA16056~RFU:2159.68 |
| Anthrax008~B14R03C09 | 0.010028616 | Ba~MGC:BA2434~BA696932~RFU:27050.22 |
| Anthrax008~B48R05C01 | 0.010060043 | Ba~MGC:BA4798~BA698092~RFU:60098.42 |
| Anthrax008~B18R03C19 | 0.010199886 | Ba~MGC:BA0517~BA696006~RFU:59965.97 |
| Anthrax008~B05R02C02 | 0.010200369 | Ba~MGC:BA4869~BA698132~RFU:29176.78 |
| Anthrax008~B42R05C10 | 0.010219729 | Ba~MGC:BA1244~BA696342~RFU:34291.76 |
| Anthrax008~B47R03C03 | 0.010223615 | Ba~MGC:BA2279~BA696856~RFU:60062.55 |
| Anthrax008~B12R05C15 | 0.01033238 | Ba~MGC:BA3491~BA697419~RFU:60019.32 |
| Anthrax008~B41R03C16 | 0.010349287 | Ba~MGC:BA1573~BA696503~RFU:59964.13 |
| Anthrax008~B41R03C05 | 0.010455469 | Ba~MGC:BA4815~BA698100~RFU:60049.67 |
| Anthrax008~B48R02C09 | 0.010469839 | Ba~MGC:BA5002~BA698203~RFU:0 |
| Anthrax008~B43R05C16 | 0.010500189 | Ba~MGC:BA1370~BA696402~RFU:60046.91 |
| Anthrax008~B31R13C06 | 0.010542215 | Ba~MGC:BA4768~BA14360~RFU:32960.82 |
| Anthrax008~B48R05C02 | 0.010571034 | Ba~MGC:BA4798~BA698092~RFU:60010.12 |
| Anthrax008~B14R02C10 | 0.010587087 | Ba~MGC:BA0438~BA695972~RFU:8.58 |
| Anthrax008~B45R02C13 | 0.010590296 | Ba~MGC:BA2692~BA697044~RFU:60036.79 |
| Anthrax008~B09R14C17 | 0.010743173 | Ba~MGC:BA3474~BA698807~RFU:45449.21 |
| Anthrax008~B13R15C18 | 0.010766665 | Ba~MGC:BA3114~BA697256~RFU:7277.41 |
| Anthrax008~B34R02C18 | 0.010786511 | Ba~MGC:BA4205~BA697783~RFU:2.76 |
| Anthrax008~B12R05C14 | 0.010824358 | Ba~MGC:BA0594~BA696039~RFU:60000 |
| Anthrax008~B09R08C17 | 0.010832893 | Ba~MGC:BA0932~BA13718~RFU:8854.08 |
| Anthrax008~B48R02C05 | 0.01087701 | Ba~MGC:BA0169~BA695824~RFU:0 |
| Anthrax008~B10R04C13 | 0.010878486 | Ba~MGC:BA0715~BA696096~RFU:54276.11 |
| Anthrax008~B18R15C07 | 0.010883133 | Ba~MGC:BA1809~BA14846~RFU:60074.5 |
| Anthrax008~B40R13C01 | 0.010913746 | Ba~MGC:BA0609~BA16683~RFU:547.28 |
| Anthrax008~B15R05C06 | 0.010915653 | Ba~MGC:BA5614~BA698509~RFU:55366.24 |
| Anthrax008~B39R03C03 | 0.010978244 | Ba~MGC:BA1488~BA696456~RFU:60059.79 |
| Anthrax008~B34R04C15 | 0.011104988 | Ba~MGC:BA2569~BA696986~RFU:60112.21 |
| Anthrax008~B43R03C06 | 0.011134521 | Ba~MGC:BA4679~BA698029~RFU:60022.08 |
| Anthrax008~B48R01C18 | 0.01122733 | Ba~MGC:BA1797~BA696613~RFU:1.43 |
| Anthrax008~B30R04C18 | 0.011265372 | Ba~MGC:BA2458~BA696946~RFU:60020.24 |
| Anthrax008~B29R04C13 | 0.01129341 | Ba~MGC:BA4797~BA698091~RFU:60047.83 |
| Anthrax008~B35R15C19 | 0.011471635 | Ba~MGC:BA3893~BA15359~RFU:10336.64 |
| Anthrax008~B46R03C15 | 0.011482308 | Ba~MGC:BA1143~BA696293~RFU:59986.2 |
| Anthrax008~B08R08C20 | 0.011516156 | Ba~MGC:BA2342~BA14170~RFU:26489.15 |
| Anthrax008~B15R01C17 | 0.01160208 | Ba~MGC:BA5355~BA698384~RFU:46489.27 |
| Anthrax008~B45R05C07 | 0.011674101 | Ba~MGC:BA4342~BA697862~RFU:44838.12 |
| Anthrax008~B48R06C13 | 0.011702298 | Ba~MGC:BA0048~BA695753~RFU:60050.59 |
| Anthrax008~B36R02C10 | 0.011769615 | Ba~MGC:BA3641~BA697496~RFU:60074.5 |
| Anthrax008~B46R05C19 | 0.011808299 | Ba~MGC:BA2016~BA696718~RFU:46804.01 |
| Anthrax008~B22R08C17 | 0.011859457 | Ba~MGC:BA2519~BA14282~RFU:0 |
| Anthrax008~B18R10C16 | 0.011892395 | Ba~MGC:BXB0074~BA15994~RFU:6068.67 |
| Anthrax008~B44R05C04 | 0.011907928 | Ba~MGC:BA0135~BA695802~RFU:59994.48 |
| Anthrax008~B18R03C13 | 0.012002799 | Ba~MGC:BA4236~BA697797~RFU:60028.51 |
| Anthrax008~B44R04C05 | 0.012021464 | Ba~MGC:BA3339~BA697342~RFU:60005.52 |
| Anthrax008~B45R02C14 | 0.01207142 | Ba~MGC:BA2692~BA697044~RFU:60065.31 |
| Anthrax008~B10R03C17 | 0.012092246 | Ba~MGC:BA3608~BA697481~RFU:51420.6 |
| Anthrax008~B14R05C06 | 0.012131843 | Ba~MGC:BA5452~BA698438~RFU:59999.08 |
| Anthrax008~B01R15C08 | 0.01214208 | Ba~MGC:BA2129~BA696776~RFU:1243.56 |
| Anthrax008~B13R14C14 | 0.012160527 | Ba~MGC:BA3529~BA697440~RFU:48961.55 |
| Anthrax008~B43R08C11 | 0.012204178 | Ba~MGC:BA1166~BA14740~RFU:21911.3 |
| Anthrax008~B36R05C16 | 0.012260235 | Ba~MGC:BA0111~BA695792~RFU:59936.53 |
| Anthrax008~B10R03C13 | 0.012278783 | Ba~MGC:BA0032~BA695744~RFU:59962.29 |
| Anthrax008~B01R03C04 | 0.012317797 | Ba~MGC:BA2841~BA697110~RFU:60119.57 |
| Anthrax008~B01R08C20 | 0.012323537 | Ba~MGC:BA1122~BA13778~RFU:5151.65 |
| Anthrax008~B48R02C12 | 0.012407947 | Ba~MGC:BA1458~BA696442~RFU:22.99 |
| Anthrax008~B13R15C17 | 0.012432007 | Ba~MGC:BA3114~BA697256~RFU:6816.59 |
| Anthrax008~B33R02C14 | 0.01244053 | Ba~MGC:BA1538~BA696481~RFU:60023.91 |
| Anthrax008~B46R05C20 | 0.012489666 | Ba~MGC:BA2016~BA696718~RFU:30826.9 |
| Anthrax008~B35R14C19 | 0.012587508 | Ba~MGC:BA2088~BA696753~RFU:60044.15 |
| Anthrax008~B19R08C15 | 0.012605295 | Ba~MGC:BA0986~BA14300~RFU:24148.27 |
| Anthrax008~B27R11C06 | 0.012618568 | Ba~MGC:BA5290~BA14230~RFU:34543.78 |
| Anthrax008~B02R07C06 | 0.012711007 | Ba~MGC:BA2175~BA696798~RFU:28119.94 |
| Anthrax008~B46R08C11 | 0.012718359 | Ba~MGC:BA2693~BA14168~RFU:0 |
| Anthrax008~B02R10C08 | 0.012742742 | Ba~MGC:BA4164~BA13424~RFU:12176.23 |
| Anthrax008~B44R02C08 | 0.012789402 | Ba~MGC:BA3990~BA697676~RFU:60068.98 |
| Anthrax008~B06R03C20 | 0.01283294 | Ba~MGC:BA4674~BA698028~RFU:60034.03 |
| Anthrax008~B34R10C13 | 0.012873176 | Ba~MGC:BXB0096~BA14624~RFU:8107.3 |
| Anthrax008~B27R14C11 | 0.012887001 | Ba~MGC:BA2439~BA696936~RFU:0 |
| Anthrax008~B33R09C19 | 0.0129722 | Ba~MGC:BA3763~BA13228~RFU:7178.99 |
| Anthrax008~B34R04C01 | 0.012994321 | Ba~MGC:BA3861~BA697601~RFU:60086.46 |
| Anthrax008~B40R04C18 | 0.013005757 | Ba~MGC:BA4612~BA698002~RFU:60011.96 |
| Anthrax008~B12R05C08 | 0.013106204 | Ba~MGC:BA0644~BA696063~RFU:60058.87 |
| Anthrax008~B19R06C09 | 0.013131407 | Ba~MGC:BA1216~BA696323~RFU:60073.58 |
| Anthrax008~B13R04C09 | 0.013204696 | Ba~MGC:BA2840~BA697109~RFU:59535.5 |
| Anthrax008~B36R02C12 | 0.013216188 | Ba~MGC:BA3921~BA697635~RFU:60044.15 |
| Anthrax008~B34R04C02 | 0.013258079 | Ba~MGC:BA3861~BA697601~RFU:60092.9 |
| Anthrax008~B35R15C07 | 0.013283662 | Ba~MGC:BA2808~BA697095~RFU:23794.15 |
| Anthrax008~B21R03C06 | 0.013293259 | Ba~MGC:BA3106~BA697253~RFU:0 |
| Anthrax008~B05R02C11 | 0.01332652 | Ba~MGC:BA0127~BA695799~RFU:57351.91 |
| Anthrax008~B46R04C03 | 0.013336252 | Ba~MGC:BA2002~BA696712~RFU:59953.09 |
| Anthrax008~B03R03C18 | 0.013338339 | Ba~MGC:BA2377~BA696904~RFU:60051.51 |
| Anthrax008~B39R03C04 | 0.013363673 | Ba~MGC:BA1488~BA696456~RFU:60069.9 |
| Anthrax008~B18R10C15 | 0.013371964 | Ba~MGC:BXB0074~BA15994~RFU:5473.53 |
| Anthrax008~B07R06C09 | 0.01347142 | Ba~MGC:BA4235~BA697796~RFU:47299.48 |
| Anthrax008~B35R03C17 | 0.013483908 | Ba~MGC:BA2459~BA696947~RFU:51541.57 |
| Anthrax008~B11R07C04 | 0.013590493 | Ba~MGC:BA1602~BA696518~RFU:20615.16 |
| Anthrax008~B44R06C09 | 0.013674784 | Ba~MGC:BA5601~BA698504~RFU:59976.09 |
| Anthrax008~B14R13C16 | 0.013684765 | Ba~MGC:BA3136~BA3136~RFU:871.04 |
| Anthrax008~B46R06C01 | 0.01376082 | Ba~MGC:BA4394.1~BA697891~RFU:60026.67 |
| Anthrax008~B38R11C08 | 0.013832566 | Ba~MGC:BXB0046~BA16018~RFU:7743.92 |
| Anthrax008~B28R03C04 | 0.01385775 | Ba~MGC:BA3943~BA697648~RFU:57293.97 |
| Anthrax008~B07R06C10 | 0.013963998 | Ba~MGC:BA4235~BA697796~RFU:45772.63 |
| Anthrax008~B31R10C15 | 0.013989188 | Ba~MGC:BA5137~BA13404~RFU:14716.7 |
| Anthrax008~B40R05C11 | 0.013990535 | Ba~MGC:BA1144~BA696294~RFU:59882.27 |
| Anthrax008~B33R02C18 | 0.014080754 | Ba~MGC:BA4562~BA697977~RFU:60089.22 |
| Anthrax008~B15R04C05 | 0.014129214 | Ba~MGC:BA4750~BA698066~RFU:60111.3 |
| Anthrax008~B29R12C11 | 0.014162967 | Ba~MGC:BA4711~BA4711~RFU:20902.72 |
| Anthrax008~B37R02C07 | 0.014174348 | Ba~MGC:BA0637~BA696060~RFU:60070.82 |
| Anthrax008~B11R06C15 | 0.014190643 | Ba~MGC:BA2354~BA696895~RFU:3828.18 |
| Anthrax008~B33R08C10 | 0.014203443 | Ba~MGC:BA1292~BA13966~RFU:45517.88 |
| Anthrax008~B43R07C02 | 0.014299736 | Ba~MGC:BA0086~BA695778~RFU:6129.51 |
| Anthrax008~B32R09C19 | 0.014462268 | Ba~MGC:BA5137~BA13404~RFU:6396.25 |
| Anthrax008~B47R02C19 | 0.014597456 | Ba~MGC:BA4270~BA697814~RFU:60080.02 |
| Anthrax008~B36R06C04 | 0.014707104 | Ba~MGC:BA1093~BA696272~RFU:31201.72 |
| Anthrax008~B08R03C19 | 0.014725816 | Ba~MGC:BA5350~BA698379~RFU:60038.63 |
| Anthrax008~B18R14C12 | 0.014738047 | Ba~MGC:BA5477~BA698825~RFU:95.85 |
| Anthrax008~B33R05C12 | 0.014752081 | Ba~MGC:BA4819~BA698103~RFU:59986.2 |
| Anthrax008~B03R02C05 | 0.01484306 | Ba~MGC:BA0836~BA696157~RFU:60080.94 |
| Anthrax008~B14R08C11 | 0.014872531 | Ba~MGC:BA2495~BA12808~RFU:0 |
| Anthrax008~B35R04C16 | 0.01489835 | Ba~MGC:BA5569~BA698488~RFU:59855.59 |
| Anthrax008~B34R06C10 | 0.014908306 | Ba~MGC:BA4650~BA698017~RFU:47959.9 |
| Anthrax008~B20R06C05 | 0.014910336 | Ba~MGC:BA4817~BA698102~RFU:60056.11 |
| Anthrax008~B40R12C02 | 0.014930289 | Ba~MGC:BA1175~BA13074~RFU:33841.98 |
| Anthrax008~B11R08C14 | 0.014935114 | Ba~MGC:BA1032~BA14952~RFU:2930.46 |
| Anthrax008~B11R14C14 | 0.014977803 | Ba~MGC:BA4213~BA697786~RFU:60095.66 |
| Anthrax008~B09R14C18 | 0.014990157 | Ba~MGC:BA3474~BA698807~RFU:43772.53 |
| Anthrax008~B04R06C06 | 0.015038675 | Ba~MGC:BA4573~BA697982~RFU:59919.98 |
| Anthrax008~B43R03C02 | 0.015058593 | Ba~MGC:BA0131~BA695801~RFU:59978.84 |
| Anthrax008~B11R03C09 | 0.015110591 | Ba~MGC:BA1226~BA696329~RFU:49217.26 |
| Anthrax008~B21R12C15 | 0.015125708 | Ba~MGC:BA2616~BA12661~RFU:19308.31 |
| Anthrax008~B30R01C19 | 0.01514299 | Ba~MGC:BA3349~BA697346~RFU:23.91 |
| Anthrax008~B22R01C17 | 0.015241954 | Ba~MGC:BA4774~BA698082~RFU:0 |
| Anthrax008~B26R03C08 | 0.015303153 | Ba~MGC:BA5284~BA698346~RFU:60088.3 |
| Anthrax008~B11R07C03 | 0.015342877 | Ba~MGC:BA1602~BA696518~RFU:21300.59 |
| Anthrax008~B33R10C04 | 0.015436605 | Ba~MGC:BA3669~BA13592~RFU:29312.91 |
| Anthrax008~B10R04C08 | 0.015580146 | Ba~MGC:BA4014~BA697691~RFU:7282.01 |
| Anthrax008~B14R08C09 | 0.01562831 | Ba~MGC:BA2562~BA15500~RFU:0 |
| Anthrax008~B12R02C20 | 0.015706088 | Ba~MGC:BA0331~BA695912~RFU:60075.42 |
| Anthrax008~B30R13C18 | 0.015707256 | Ba~MGC:BA2252~BA14248~RFU:60117.73 |
| Anthrax008~B22R08C15 | 0.01575595 | Ba~MGC:BA2350~BA16176~RFU:0 |
| Anthrax008~B36R11C08 | 0.015769582 | Ba~MGC:BXA0026~BA15896~RFU:4711.18 |
| Anthrax008~B15R07C05 | 0.015794799 | Ba~MGC:BA4168~BA697763~RFU:60115.89 |
| Anthrax008~B34R07C01 | 0.01581045 | Ba~MGC:BA4193~BA697776~RFU:60115.89 |
| Anthrax008~B07R05C06 | 0.015827224 | Ba~MGC:BA2844~BA697112~RFU:60077.26 |
| Anthrax008~B48R04C02 | 0.015844094 | Ba~MGC:BA3621~BA697487~RFU:60025.75 |
| Anthrax008~B28R06C02 | 0.015947899 | Ba~MGC:BA4526~BA697957~RFU:60089.22 |
| Anthrax008~B26R12C02 | 0.015977465 | Ba~MGC:BA4677~BA16600~RFU:1209.53 |
| Anthrax008~B30R10C10 | 0.015997251 | Ba~MGC:BA4615~BA13702~RFU:0 |
| Anthrax008~B34R09C19 | 0.016015202 | Ba~MGC:BA4246~BA15458~RFU:2040.06 |
| Anthrax008~B14R02C12 | 0.016063962 | Ba~MGC:BA1877~BA696654~RFU:0 |
| Anthrax008~B04R02C02 | 0.01610063 | Ba~MGC:BA0183~BA695833~RFU:60040.47 |
| Anthrax008~B34R06C20 | 0.01623522 | Ba~MGC:BA3422~BA697387~RFU:32632.45 |
| Anthrax008~B19R03C18 | 0.016317053 | Ba~MGC:BA3811~BA697575~RFU:60030.35 |
| Anthrax008~B11R07C16 | 0.016383655 | Ba~MGC:BA0012~BA695730~RFU:21383.37 |
| Anthrax008~B27R08C08 | 0.016452145 | Ba~MGC:BA1366~BA15280~RFU:8425.31 |
| Anthrax008~B36R05C20 | 0.016465101 | Ba~MGC:BA2145~BA696784~RFU:60045.07 |
| Anthrax008~B43R04C05 | 0.016476592 | Ba~MGC:BA0744.1~BA696113~RFU:59805.92 |
| Anthrax008~B43R03C10 | 0.016504595 | Ba~MGC:BA5641~BA698525~RFU:60055.19 |
| Anthrax008~B40R02C20 | 0.016516207 | Ba~MGC:BA4608~BA697998~RFU:60021.16 |
| Anthrax008~B38R05C03 | 0.016599381 | Ba~MGC:BA0448~BA695977~RFU:0 |
| Anthrax008~B08R06C12 | 0.016831433 | Ba~MGC:BA0376~BA695938~RFU:60113.13 |
| Anthrax008~B45R06C18 | 0.016876575 | Ba~MGC:BA4565.1~BA697979~RFU:59958.61 |
| Anthrax008~B30R13C17 | 0.016943235 | Ba~MGC:BA2252~BA14248~RFU:60108.54 |
| Anthrax008~B31R02C07 | 0.017030887 | Ba~MGC:BA3089~BA697241~RFU:60115.89 |
| Anthrax008~B09R03C10 | 0.017036008 | Ba~MGC:BA2066~BA696743~RFU:60097.5 |
| Anthrax008~B22R18C07 | 0.017172985 | PA_25n~N/A |
| Anthrax008~B11R03C10 | 0.017181685 | Ba~MGC:BA1226~BA696329~RFU:52805.37 |
| Anthrax008~B04R02C01 | 0.017247802 | Ba~MGC:BA0183~BA695833~RFU:60056.11 |
| Anthrax008~B35R11C08 | 0.017276103 | Ba~MGC:BA5104~BA16116~RFU:2057.58 |
| Anthrax008~B30R04C17 | 0.017278533 | Ba~MGC:BA2458~BA696946~RFU:60032.19 |
| Anthrax008~B27R14C10 | 0.017287757 | Ba~MGC:BA1240~BA696339~RFU:43044.52 |
| Anthrax008~B35R16C13 | 0.017419847 | Ba~MGC:BA1474~BA1474~RFU:16942.6 |
| Anthrax008~B39R12C18 | 0.01762462 | Ba~MGC:BA3522~BA13214~RFU:25054.36 |
| Anthrax008~B22R11C09 | 0.017657217 | Ba~MGC:BA0094~BA0094~RFU:51357.62 |
| Anthrax008~B44R02C07 | 0.017659968 | Ba~MGC:BA3990~BA697676~RFU:60080.02 |
| Anthrax008~B42R05C18 | 0.017682426 | Ba~MGC:BA5737~BA698575~RFU:60109.46 |
| Anthrax008~B16R14C15 | 0.01771175 | Ba~MGC:BA2529~BA698781~RFU:59528.15 |
| Anthrax008~B48R04C01 | 0.017728781 | Ba~MGC:BA3621~BA697487~RFU:60099.34 |
| Anthrax008~B14R03C10 | 0.017764578 | Ba~MGC:BA2434~BA696932~RFU:29215.42 |
| Anthrax008~B02R03C18 | 0.017775606 | Ba~MGC:BA1227~BA696330~RFU:60099.34 |
| Anthrax008~B04R03C13 | 0.017884746 | Ba~MGC:BA2227~BA696825~RFU:60025.75 |
| Anthrax008~B30R08C11 | 0.017973317 | Ba~MGC:BA2626~BA14344~RFU:3.68 |
| Anthrax008~B09R08C10 | 0.018125825 | Ba~MGC:BA1035~BA14328~RFU:29076.53 |
| Anthrax008~B46R04C17 | 0.018144459 | Ba~MGC:BA1585~BA696509~RFU:60043.23 |
| Anthrax008~B04R06C05 | 0.018148849 | Ba~MGC:BA4573~BA697982~RFU:59795.81 |
| Anthrax008~B40R13C16 | 0.0181573 | Ba~MGC:BA5245~BA5245~RFU:3056.48 |
| Anthrax008~B48R07C17 | 0.01835525 | Ba~MGC:BA3373~BA697364~RFU:1537.9 |
| Anthrax008~B36R02C11 | 0.018367909 | Ba~MGC:BA3921~BA697635~RFU:60064.39 |
| Anthrax008~B44R04C11 | 0.0184189 | Ba~MGC:BA4624~BA698008~RFU:57336.28 |
| Anthrax008~B11R14C13 | 0.018419737 | Ba~MGC:BA4213~BA697786~RFU:60108.54 |
| Anthrax008~B18R15C11 | 0.018439254 | Ba~MGC:BA5276~BA16385~RFU:28578.92 |
| Anthrax008~B38R02C20 | 0.018473476 | Ba~MGC:BA4410~BA697899~RFU:41204.58 |
| Anthrax008~B34R12C07 | 0.018489107 | Ba~MGC:BA3659~BA3659~RFU:23880.61 |
| Anthrax008~B46R05C08 | 0.018561797 | Ba~MGC:BA3212~BA698801~RFU:60084.62 |
| Anthrax008~B46R05C10 | 0.018677548 | Ba~MGC:BA2207~BA696814~RFU:60054.27 |
| Anthrax008~B11R07C06 | 0.018683283 | Ba~MGC:BA4385~BA697886~RFU:14023.18 |
| Anthrax008~B40R02C19 | 0.018729912 | Ba~MGC:BA4608~BA697998~RFU:59971.49 |
| Anthrax008~B48R10C04 | 0.018778212 | Ba~MGC:BA5228~BA14098~RFU:1417.74 |
| Anthrax008~B05R02C01 | 0.018814035 | Ba~MGC:BA4869~BA698132~RFU:29094.92 |
| Anthrax008~B16R02C06 | 0.018918118 | Ba~MGC:BA2253~BA696845~RFU:0 |
| Anthrax008~B09R08C13 | 0.018997962 | Ba~MGC:BA0814~BA13152~RFU:6248.93 |
| Anthrax008~B02R03C17 | 0.019011421 | Ba~MGC:BA1227~BA696330~RFU:60089.22 |
| Anthrax008~B32R09C20 | 0.019042997 | Ba~MGC:BA5137~BA13404~RFU:6499.26 |
| Anthrax008~B11R15C04 | 0.01907044 | Ba~MGC:BA2655~BA698784~RFU:60002.76 |
| Anthrax008~B46R04C04 | 0.019148352 | Ba~MGC:BA2002~BA696712~RFU:60019.32 |
| Anthrax008~B30R13C09 | 0.019235965 | Ba~MGC:BA4672~BA4672~RFU:421.27 |
| Anthrax008~B46R03C16 | 0.019404845 | Ba~MGC:BA1143~BA696293~RFU:59965.97 |
| Anthrax008~B22R07C19 | 0.019445006 | Ba~MGC:BA0871~BA696174~RFU:577.63 |
| Anthrax008~B01R06C16 | 0.01951658 | Ba~MGC:BA0246~BA695859~RFU:3460.26 |
| Anthrax008~B19R03C17 | 0.019528698 | Ba~MGC:BA3811~BA697575~RFU:60018.4 |
| Anthrax008~B11R04C13 | 0.019583238 | Ba~MGC:BA0038.1~BA695747~RFU:60038.63 |
| Anthrax008~B27R07C06 | 0.019591622 | Ba~MGC:BA5298~BA698353~RFU:13165.95 |
| Anthrax008~B29R12C19 | 0.019603005 | Ba~MGC:BA3810~BA3810~RFU:2020.79 |
| Anthrax008~B12R10C14 | 0.01971049 | Ba~MGC:BXA0188~BA13750~RFU:7405.26 |
| Anthrax008~B21R15C03 | 0.01973937 | Ba~MGC:BA3844~BA697595~RFU:2498.16 |
| Anthrax008~B09R07C04 | 0.019765358 | Ba~MGC:BA1073~BA696261~RFU:4006.62 |
| Anthrax008~B19R04C17 | 0.019770943 | Ba~MGC:BA4673~BA698027~RFU:59973.33 |
| Anthrax008~B10R02C09 | 0.019924711 | Ba~MGC:BA5361.1~BA698389~RFU:60034.03 |
| Anthrax008~B07R02C02 | 0.019982759 | Ba~MGC:BA5195~BA698304~RFU:22632.45 |
| Anthrax008~B22R18C16 | 0.020119216 | PA_1.5625n~N/A |
| Anthrax008~B46R10C06 | 0.020176416 | Ba~MGC:BA4779~BA13646~RFU:68.98 |
| Anthrax008~B47R07C05 | 0.02022849 | Ba~MGC:BA3537~BA697446~RFU:60128.77 |
| Anthrax008~B14R13C15 | 0.020312736 | Ba~MGC:BA3136~BA3136~RFU:857.25 |
| Anthrax008~B24R15C11 | 0.020328684 | Ba~MGC:BA0774~BA17122~RFU:611.66 |
| Anthrax008~B24R15C13 | 0.020367332 | Ba~MGC:BA1420~BA16549~RFU:1005.33 |
| Anthrax008~B30R11C10 | 0.020368395 | Ba~MGC:BA0042~BA12881~RFU:9092.16 |
| Anthrax008~B33R04C14 | 0.02039343 | Ba~MGC:BA1194~BA696313~RFU:60008.28 |
| Anthrax008~B43R03C09 | 0.020525327 | Ba~MGC:BA5641~BA698525~RFU:60050.59 |
| Anthrax008~B46R04C18 | 0.020549577 | Ba~MGC:BA1585~BA696509~RFU:60068.06 |
| Anthrax008~B19R08C14 | 0.020699333 | Ba~MGC:BA1447~BA15140~RFU:5582.23 |
| Anthrax008~B48R10C11 | 0.020723 | Ba~MGC:BA4678~BA15900~RFU:1159.86 |
| Anthrax008~B40R04C15 | 0.020731736 | Ba~MGC:BA4760~BA698074~RFU:59832.6 |
| Anthrax008~B45R07C09 | 0.020781627 | Ba~MGC:BA4309~BA697836~RFU:862.77 |
| Anthrax008~B17R05C10 | 0.0208117 | Ba~MGC:BA1349~BA696395~RFU:59866.63 |
| Anthrax008~B29R14C15 | 0.020925237 | Ba~MGC:BA2554~BA696979~RFU:4297.57 |
| Anthrax008~B43R03C01 | 0.020951824 | Ba~MGC:BA0131~BA695801~RFU:60051.51 |
| Anthrax008~B38R08C05 | 0.020971859 | Ba~MGC:BA2695~BA14318~RFU:0 |
| Anthrax008~B39R07C12 | 0.021189868 | Ba~MGC:BA5367~BA698392~RFU:865.53 |
| Anthrax008~B39R02C04 | 0.021214684 | Ba~MGC:BA0205~BA695845~RFU:60055.19 |
| Anthrax008~B30R01C18 | 0.021264134 | Ba~MGC:BA0216~BA695848~RFU:27.59 |
| Anthrax008~B27R04C14 | 0.021277785 | Ba~MGC:BA4154~BA697758~RFU:60050.59 |
| Anthrax008~B16R06C02 | 0.021400683 | Ba~MGC:BA2800~BA697093~RFU:16905.58 |
| Anthrax008~B22R08C06 | 0.021400921 | Ba~MGC:BA2375~BA15418~RFU:0 |
| Anthrax008~B34R13C05 | 0.021423794 | Ba~MGC:BA2644~BA2644~RFU:4941.13 |
| Anthrax008~B21R03C05 | 0.021444116 | Ba~MGC:BA3106~BA697253~RFU:2.86 |
| Anthrax008~B35R14C05 | 0.021478023 | Ba~MGC:BA2916~BA697144~RFU:35849.89 |
| Anthrax008~B19R05C01 | 0.021492997 | Ba~MGC:BA0386~BA695946~RFU:60071.74 |
| Anthrax008~B29R13C19 | 0.021630371 | Ba~MGC:BA4000~BA4000~RFU:810.34 |
| Anthrax008~B03R04C20 | 0.021639405 | Ba~MGC:BA0017~BA695735~RFU:60016.56 |
| Anthrax008~B28R04C06 | 0.021707388 | Ba~MGC:BA1729.1~BA696579~RFU:59204.38 |
| Anthrax008~B40R09C20 | 0.021727074 | Ba~MGC:BA5086~BA15868~RFU:3811.63 |
| Anthrax008~B02R14C14 | 0.021849219 | Ba~MGC:BA0494~BA695992~RFU:36798.28 |
| Anthrax008~B35R15C04 | 0.0219144 | Ba~MGC:BA4840~BA14142~RFU:27473.33 |
| Anthrax008~B46R04C07 | 0.021956993 | Ba~MGC:BA3966~BA697663~RFU:60003.68 |
| Anthrax008~B16R12C04 | 0.022218397 | Ba~MGC:BA0512~BA0512~RFU:51947.2 |
| Anthrax008~B11R15C05 | 0.022303477 | Ba~MGC:BA0072~BA695766~RFU:59988.96 |
| Anthrax008~B30R07C11 | 0.022452502 | Ba~MGC:BA0080~BA695773~RFU:2510.12 |
| Anthrax008~B24R15C12 | 0.022518495 | Ba~MGC:BA0774~BA17122~RFU:541.76 |
| Anthrax008~B09R08C18 | 0.022717677 | Ba~MGC:BA0932~BA13718~RFU:9515.02 |
| Anthrax008~B17R05C09 | 0.02289245 | Ba~MGC:BA1349~BA696395~RFU:59900.66 |
| Anthrax008~B21R02C17 | 0.022893962 | Ba~MGC:BA5724~BA698571~RFU:60092.9 |
| Anthrax008~B19R08C13 | 0.02292617 | Ba~MGC:BA1447~BA15140~RFU:4335.91 |
| Anthrax008~B45R07C10 | 0.022962775 | Ba~MGC:BA4309~BA697836~RFU:959.94 |
| Anthrax008~B31R02C08 | 0.023071213 | Ba~MGC:BA3089~BA697241~RFU:60101.18 |
| Anthrax008~B43R07C17 | 0.023175536 | Ba~MGC:BA2153~BA696787~RFU:2152.32 |
| Anthrax008~B26R11C12 | 0.023229895 | Ba~MGC:BXA0011~BA17108~RFU:2858.37 |
| Anthrax008~B24R14C03 | 0.023286795 | Ba~MGC:BA0309~BA695897~RFU:60102.1 |
| Anthrax008~B03R08C14 | 0.02336744 | Ba~MGC:BA0947~BA13982~RFU:16959.16 |
| Anthrax008~B33R02C13 | 0.023625555 | Ba~MGC:BA1538~BA696481~RFU:60014.72 |
| Anthrax008~B41R02C10 | 0.023661023 | Ba~MGC:BA4759~BA698073~RFU:60005.52 |
| Anthrax008~B10R04C15 | 0.02370488 | Ba~MGC:BA4510~BA698819~RFU:59990.8 |
| Anthrax008~B38R02C19 | 0.023899829 | Ba~MGC:BA4410~BA697899~RFU:37918.45 |
| Anthrax008~B14R06C03 | 0.024137523 | Ba~MGC:BA4664~BA698024~RFU:59945.73 |
| Anthrax008~B16R08C05 | 0.024159161 | Ba~MGC:BA2532~BA13688~RFU:0 |
| Anthrax008~B01R06C11 | 0.024262069 | Ba~MGC:BA1188~BA696311~RFU:60045.07 |
| Anthrax008~B34R09C18 | 0.024336917 | Ba~MGC:BA4394~BA15216~RFU:11074.39 |
| Anthrax008~B41R05C09 | 0.0244611 | Ba~MGC:BA0766~BA696124~RFU:0 |
| Anthrax008~B38R05C17 | 0.024553437 | Ba~MGC:BA2252.1~BA696844~RFU:60052.43 |
| Anthrax008~B15R15C04 | 0.024670044 | Ba~MGC:BA5255~BA698333~RFU:1571.01 |
| Anthrax008~B33R04C13 | 0.02469639 | Ba~MGC:BA1194~BA696313~RFU:59951.25 |
| Anthrax008~B29R10C18 | 0.024725366 | Ba~MGC:BA5194~BA14092~RFU:2419.17 |
| Anthrax008~B14R04C13 | 0.024813546 | Ba~MGC:BA1582~BA696508~RFU:53111.66 |
| Anthrax008~B35R13C19 | 0.02482164 | Ba~MGC:BA0230~BA0230~RFU:59958.61 |
| Anthrax008~B03R14C16 | 0.02502004 | Ba~MGC:BA0953~BA696215~RFU:59990.8 |
| Anthrax008~B09R05C15 | 0.025084628 | Ba~MGC:BA2945~BA697162~RFU:60024.83 |
| Anthrax008~B11R13C08 | 0.025102874 | Ba~MGC:BA4191~BA14889~RFU:361.48 |
| Anthrax008~B15R04C08 | 0.025244668 | Ba~MGC:BA1182~BA696306~RFU:60054.27 |
| Anthrax008~B21R11C13 | 0.025258098 | Ba~MGC:BA2196~BA16432~RFU:16281.83 |
| Anthrax008~B01R08C14 | 0.025598172 | Ba~MGC:BA1444~BA13502~RFU:6623.44 |
| Anthrax008~B38R08C19 | 0.025615271 | Ba~MGC:BA2172~BA14044~RFU:0 |
| Anthrax008~B34R09C17 | 0.025661826 | Ba~MGC:BA4394~BA15216~RFU:10878.4 |
| Anthrax008~B29R12C12 | 0.025683686 | Ba~MGC:BA4711~BA4711~RFU:13929.9 |
| Anthrax008~B09R03C09 | 0.025817242 | Ba~MGC:BA2066~BA696743~RFU:60090.14 |
| Anthrax008~B04R03C14 | 0.025902724 | Ba~MGC:BA2227~BA696825~RFU:60054.27 |
| Anthrax008~B02R05C03 | 0.025939547 | Ba~MGC:BA1469~BA696446~RFU:60058.87 |
| Anthrax008~B01R15C07 | 0.026022507 | Ba~MGC:BA2129~BA696776~RFU:1078.92 |
| Anthrax008~B39R06C07 | 0.0260435 | Ba~MGC:BA1195~BA696314~RFU:60093.82 |
| Anthrax008~B06R03C19 | 0.026080195 | Ba~MGC:BA4674~BA698028~RFU:60022.08 |
| Anthrax008~B44R04C06 | 0.02617068 | Ba~MGC:BA3339~BA697342~RFU:59990.8 |
| Anthrax008~B18R03C14 | 0.026239103 | Ba~MGC:BA4236~BA697797~RFU:60043.23 |
| Anthrax008~B34R04C19 | 0.026474519 | Ba~MGC:BA3458~BA697402~RFU:60048.75 |
| Anthrax008~B05R12C17 | 0.026550438 | Ba~MGC:BA2660~BA13147~RFU:43265.27 |
| Anthrax008~B34R02C02 | 0.02657034 | Ba~MGC:BA1429~BA696429~RFU:60107.62 |
| Anthrax008~B15R12C08 | 0.026660139 | Ba~MGC:BA4392~BA4392~RFU:36179.18 |
| Anthrax008~B20R10C09 | 0.02699217 | Ba~MGC:BA4373~BA15668~RFU:805.44 |
| Anthrax008~B34R13C02 | 0.0270498 | Ba~MGC:BA3258~BA3258~RFU:4172.19 |
| Anthrax008~B24R07C20 | 0.027153088 | Ba~MGC:BA4690~BA698038~RFU:1811.07 |
| Anthrax008~B39R04C09 | 0.027169704 | Ba~MGC:BA1462~BA696444~RFU:60058.87 |
| Anthrax008~B35R10C15 | 0.027339388 | Ba~MGC:BXA0101~BA16174~RFU:1066.96 |
| Anthrax008~B26R11C05 | 0.027377877 | Ba~MGC:BA5712~BA15596~RFU:4752.58 |
| Anthrax008~B29R04C14 | 0.027408907 | Ba~MGC:BA4797~BA698091~RFU:60063.47 |
| Anthrax008~B48R09C19 | 0.02747376 | Ba~MGC:BA5287~BA14606~RFU:1864.42 |
| Anthrax008~B40R05C12 | 0.027545504 | Ba~MGC:BA1144~BA696294~RFU:23055.79 |
| Anthrax008~B27R04C13 | 0.027588231 | Ba~MGC:BA4154~BA697758~RFU:60021.16 |
| Anthrax008~B45R05C08 | 0.027635703 | Ba~MGC:BA4342~BA697862~RFU:41464.31 |
| Anthrax008~B01R06C18 | 0.02766939 | Ba~MGC:BA3974~BA697669~RFU:60122.33 |
| Anthrax008~B15R10C20 | 0.027679484 | Ba~MGC:BA5056~BA15666~RFU:958.51 |
| Anthrax008~B32R13C11 | 0.027751269 | Ba~MGC:BA4399~BA4399~RFU:580.39 |
| Anthrax008~B34R12C05 | 0.027859746 | Ba~MGC:BA2525~BA13449~RFU:2987.49 |
| Anthrax008~B22R18C18 | 0.027913718 | PA_0.78125n~N/A |
| Anthrax008~B34R04C05 | 0.028079115 | Ba~MGC:BA3500~BA697424~RFU:30730.32 |
| Anthrax008~B03R05C16 | 0.028152527 | Ba~MGC:BA4303~BA697832~RFU:60068.06 |
| Anthrax008~B48R11C03 | 0.028226946 | Ba~MGC:BXB0093~BA14790~RFU:5978.54 |
| Anthrax008~B11R02C11 | 0.028257469 | Ba~MGC:BA2597~BA696997~RFU:1399.93 |
| Anthrax008~B21R02C18 | 0.028273309 | Ba~MGC:BA5724~BA698571~RFU:60103.94 |
| Anthrax008~B39R04C10 | 0.02847532 | Ba~MGC:BA1462~BA696444~RFU:60087.38 |
| Anthrax008~B03R14C15 | 0.028555451 | Ba~MGC:BA0953~BA696215~RFU:59959.53 |
| Anthrax008~B30R07C12 | 0.028563095 | Ba~MGC:BA0080~BA695773~RFU:2485.28 |
| Anthrax008~B08R14C10 | 0.028613707 | Ba~MGC:BA1869~BA13433~RFU:49749.82 |
| Anthrax008~B03R08C07 | 0.028650349 | Ba~MGC:BA1167~BA15724~RFU:7369.39 |
| Anthrax008~B13R04C10 | 0.028750362 | Ba~MGC:BA2840~BA697109~RFU:43751.07 |
| Anthrax008~B40R12C01 | 0.028824959 | Ba~MGC:BA1175~BA13074~RFU:29784.77 |
| Anthrax008~B16R06C01 | 0.028880266 | Ba~MGC:BA2800~BA697093~RFU:15036.79 |
| Anthrax008~B04R16C01 | 0.028926857 | Ba~MGC:BA1484~BA696453~RFU:46380.61 |
| Anthrax008~B04R02C07 | 0.02901247 | Ba~MGC:BA2446~BA696939~RFU:60000 |
| Anthrax008~B29R10C15 | 0.029071422 | Ba~MGC:BA5144~BA14378~RFU:1456.37 |
| Anthrax008~B48R05C18 | 0.029218314 | Ba~MGC:BA0063~BA695761~RFU:60034.95 |
| Anthrax008~B33R07C07 | 0.02940408 | Ba~MGC:BA5693~BA698551~RFU:27377.67 |
| Anthrax008~B43R02C07 | 0.029465014 | Ba~MGC:BA4529~BA697959~RFU:60108.54 |
| Anthrax008~B14R18C09 | 0.029477911 | LF_12.5n~N/A |
| Anthrax008~B20R03C10 | 0.029530559 | Ba~MGC:BA3981~BA697674~RFU:4057.22 |
| Anthrax008~B31R13C05 | 0.02964512 | Ba~MGC:BA4768~BA14360~RFU:33860.38 |
| Anthrax008~B12R06C03 | 0.029765332 | Ba~MGC:BA0472~BA695986~RFU:60024.83 |
| Anthrax008~B37R02C08 | 0.029824348 | Ba~MGC:BA0637~BA696060~RFU:60082.78 |
| Anthrax008~B14R02C07 | 0.029922405 | Ba~MGC:BA0946~BA696212~RFU:0 |
| Anthrax008~B44R06C10 | 0.029948449 | Ba~MGC:BA5601~BA698504~RFU:59962.29 |
| Anthrax008~B04R03C20 | 0.030002788 | Ba~MGC:BA0129~BA695800~RFU:60011.04 |
| Anthrax008~B34R04C06 | 0.030036166 | Ba~MGC:BA3500~BA697424~RFU:28423.47 |
| Anthrax008~B05R15C06 | 0.03009288 | Ba~MGC:BXA0061~BA698600~RFU:36938.93 |
| Anthrax008~B38R11C07 | 0.030146254 | Ba~MGC:BXB0046~BA16018~RFU:9136.31 |
| Anthrax008~B29R05C20 | 0.030200876 | Ba~MGC:BA0503~BA695998~RFU:60079.1 |
| Anthrax008~B46R04C01 | 0.0302096 | Ba~MGC:BA2031~BA696728~RFU:59977.01 |
| Anthrax008~B35R04C13 | 0.030274248 | Ba~MGC:BA5574~BA698492~RFU:59736.02 |
| Anthrax008~B25R11C05 | 0.030279357 | Ba~MGC:BA5069~BA14834~RFU:2000 |
| Anthrax008~B27R05C20 | 0.030292079 | Ba~MGC:BA1615~BA696524~RFU:36403.43 |
| Anthrax008~B07R11C03 | 0.030525077 | Ba~MGC:BA4995~BA14866~RFU:1606.88 |
| Anthrax008~B46R14C13 | 0.030658953 | Ba~MGC:BXB0110~BA16022~RFU:57240.34 |
| Anthrax008~B28R11C06 | 0.030806154 | Ba~MGC:BA5721~BA13676~RFU:7098.97 |
| Anthrax008~B23R02C10 | 0.030818785 | Ba~MGC:BA1287~BA696366~RFU:37940.58 |
| Anthrax008~B12R14C07 | 0.030901458 | Ba~MGC:BA4091~BA697729~RFU:211.55 |
| Anthrax008~B34R12C15 | 0.031027468 | Ba~MGC:BXB0086~BA13595~RFU:13389.13 |
| Anthrax008~B14R06C04 | 0.031080196 | Ba~MGC:BA4664~BA698024~RFU:59853.75 |
| Anthrax008~B22R01C18 | 0.031160764 | Ba~MGC:BA4774~BA698082~RFU:4.6 |
| Anthrax008~B30R05C10 | 0.03118199 | Ba~MGC:BA0120~BA695796~RFU:7712.47 |
| Anthrax008~B12R12C06 | 0.031198708 | Ba~MGC:BA3589~BA13916~RFU:60091.06 |
| Anthrax008~B43R04C06 | 0.03135937 | Ba~MGC:BA0744.1~BA696113~RFU:59847.31 |
| Anthrax008~B38R08C04 | 0.031439703 | Ba~MGC:BA0882~BA696181~RFU:1713.58 |
| Anthrax008~B19R04C12 | 0.031478047 | Ba~MGC:BA2717.1~BA697053~RFU:57585.54 |
| Anthrax008~B31R14C11 | 0.031480435 | Ba~MGC:BA0231~BA17167~RFU:3989.15 |
| Anthrax008~B18R10C11 | 0.03149146 | Ba~MGC:BA4420~BA13552~RFU:6812.91 |
| Anthrax008~B45R11C05 | 0.03188459 | Ba~MGC:BA4348~BA14478~RFU:7379.51 |
| Anthrax008~B47R02C12 | 0.032000426 | Ba~MGC:BA0688~BA696084~RFU:41575.11 |
| Anthrax008~B13R05C03 | 0.032020263 | Ba~MGC:BA0136~BA695803~RFU:60028.51 |
| Anthrax008~B24R06C09 | 0.032398857 | Ba~MGC:BA4593~BA697989~RFU:60070.82 |
| Anthrax008~B22R13C15 | 0.032408565 | Ba~MGC:BA1784~BA1784~RFU:679.54 |
| Anthrax008~B34R12C10 | 0.032548398 | Ba~MGC:BA4649~BA13315~RFU:21108.73 |
| Anthrax008~B11R07C15 | 0.032716185 | Ba~MGC:BA0012~BA695730~RFU:20316.41 |
| Anthrax008~B05R12C18 | 0.03278035 | Ba~MGC:BA2660~BA13147~RFU:32860.56 |
| Anthrax008~B04R02C08 | 0.03287723 | Ba~MGC:BA2446~BA696939~RFU:59982.52 |
| Anthrax008~B04R10C06 | 0.032878276 | Ba~MGC:BA4548~BA15688~RFU:3514.53 |
| Anthrax008~B46R04C08 | 0.032899283 | Ba~MGC:BA3966~BA697663~RFU:59984.36 |
| Anthrax008~B23R15C11 | 0.032901034 | Ba~MGC:BA5651~BA15670~RFU:3124.54 |
| Anthrax008~B02R08C13 | 0.032906912 | Ba~MGC:BA1729~BA13066~RFU:29492.27 |
| Anthrax008~B37R13C12 | 0.03292535 | Ba~MGC:BA1114~BA1114~RFU:1080.11 |
| Anthrax008~B18R13C17 | 0.03292787 | Ba~MGC:BA3905~BA3905~RFU:55903.24 |
| Anthrax008~B18R13C15 | 0.03294085 | Ba~MGC:BA1828~BA1828~RFU:21293.23 |
| Anthrax008~B41R05C03 | 0.03298468 | Ba~MGC:BA4256~BA697807~RFU:0 |
| Anthrax008~B33R02C17 | 0.033297718 | Ba~MGC:BA4562~BA697977~RFU:60066.23 |
| Anthrax008~B46R05C09 | 0.033496257 | Ba~MGC:BA2207~BA696814~RFU:60071.74 |
| Anthrax008~B08R04C09 | 0.03349914 | Ba~MGC:BA2765~BA697074~RFU:60106.7 |
| Anthrax008~B03R05C15 | 0.03374132 | Ba~MGC:BA4303~BA697832~RFU:60059.79 |
| Anthrax008~B32R02C14 | 0.03399669 | Ba~MGC:BA3425~BA697389~RFU:60006.44 |
| Anthrax008~B09R08C09 | 0.03415021 | Ba~MGC:BA1035~BA14328~RFU:30194.08 |
| Anthrax008~B29R02C05 | 0.034242895 | Ba~MGC:BA0857~BA696165~RFU:60097.5 |
| Anthrax008~B40R03C13 | 0.034384817 | Ba~MGC:BA5351~BA698380~RFU:59990.8 |
| Anthrax008~B38R01C18 | 0.034407936 | Ba~MGC:BA1569~BA696501~RFU:0 |
| Anthrax008~B46R05C11 | 0.034408145 | Ba~MGC:BA4477~BA697933~RFU:60006.44 |
| Anthrax008~B21R11C07 | 0.03446216 | Ba~MGC:BA5135~BA16026~RFU:4062.95 |
| Anthrax008~B42R06C10 | 0.0345048 | Ba~MGC:BA4372~BA697879~RFU:2666.67 |
| Anthrax008~B19R03C06 | 0.03455396 | Ba~MGC:BA3390~BA697369~RFU:60044.15 |
| Anthrax008~B09R10C19 | 0.034703564 | Ba~MGC:BXA0044~BA15108~RFU:7217.45 |
| Anthrax008~B02R10C07 | 0.034725476 | Ba~MGC:BA4164~BA13424~RFU:10626.61 |
| Anthrax008~B47R07C04 | 0.03499411 | Ba~MGC:BA4556.1~BA697973~RFU:60101.18 |
| Anthrax008~B15R01C18 | 0.035055906 | Ba~MGC:BA5355~BA698384~RFU:41705.29 |
| Anthrax008~B44R10C04 | 0.035171635 | Ba~MGC:BA4268~BA13626~RFU:11748.53 |
| Anthrax008~B41R11C17 | 0.035205316 | Ba~MGC:BA2041~BA17206~RFU:1869.81 |
| Anthrax008~B03R04C19 | 0.03529401 | Ba~MGC:BA0017~BA695735~RFU:60011.96 |
| Anthrax008~B13R11C07 | 0.035417706 | Ba~MGC:BA5269~BA15278~RFU:1225.17 |
| Anthrax008~B37R14C16 | 0.03541793 | Ba~MGC:BA3036~BA13665~RFU:19659.51 |
| Anthrax008~B08R06C07 | 0.035452377 | Ba~MGC:BA3628~BA697489~RFU:60095.66 |
| Anthrax008~B22R01C20 | 0.035457976 | Ba~MGC:BA3321~BA697332~RFU:16.56 |
| Anthrax008~B07R02C01 | 0.035495248 | Ba~MGC:BA5195~BA698304~RFU:22192.79 |
| Anthrax008~B10R04C07 | 0.03570087 | Ba~MGC:BA4014~BA697691~RFU:7782.38 |
| Anthrax008~B48R11C19 | 0.03575485 | Ba~MGC:BA1644~BA12445~RFU:13971.39 |
| Anthrax008~B33R04C08 | 0.03582271 | Ba~MGC:BA0188~BA695838~RFU:59913.54 |
| Anthrax008~B36R11C07 | 0.035827614 | Ba~MGC:BXA0026~BA15896~RFU:4456.4 |
| Anthrax008~B21R14C14 | 0.035843715 | Ba~MGC:BA0114~BA13534~RFU:60118.65 |
| Anthrax008~B47R03C04 | 0.03599536 | Ba~MGC:BA2279~BA696856~RFU:60083.7 |
| Anthrax008~B44R08C20 | 0.0360386 | Ba~MGC:BA0881~BA16218~RFU:22632.33 |
| Anthrax008~B48R09C20 | 0.036077265 | Ba~MGC:BA5287~BA14606~RFU:1370.53 |
| Anthrax008~B45R03C16 | 0.036185123 | Ba~MGC:BA3713~BA697527~RFU:10553.65 |
| Anthrax008~B40R08C13 | 0.036380727 | Ba~MGC:BA1645~BA14486~RFU:0 |
| Anthrax008~B27R06C08 | 0.036450915 | Ba~MGC:BA0100~BA695787~RFU:60075.42 |
| Anthrax008~B40R04C16 | 0.036477704 | Ba~MGC:BA4760~BA698074~RFU:59944.81 |
| Anthrax008~B34R02C19 | 0.03648668 | Ba~MGC:BA0110~BA695791~RFU:60056.11 |
| Anthrax008~B27R14C09 | 0.036504533 | Ba~MGC:BA1240~BA696339~RFU:41547.09 |
| Anthrax008~B44R09C20 | 0.036569774 | Ba~MGC:BA4514~BA14256~RFU:2855.51 |
| Anthrax008~B34R02C20 | 0.036589414 | Ba~MGC:BA0110~BA695791~RFU:60051.51 |
| Anthrax008~B48R08C05 | 0.036632378 | Ba~MGC:BA2500~BA14596~RFU:6.44 |
| Anthrax008~B36R12C13 | 0.036636967 | Ba~MGC:BXB0002~BA13943~RFU:32204.75 |
| Anthrax008~B02R06C06 | 0.036762774 | Ba~MGC:BA2136~BA696779~RFU:40465.42 |
| Anthrax008~B43R02C08 | 0.03678088 | Ba~MGC:BA4529~BA697959~RFU:60090.14 |
| Anthrax008~B33R08C13 | 0.03679375 | Ba~MGC:BA1084~BA14368~RFU:2203.15 |
| Anthrax008~B23R02C17 | 0.03685326 | Ba~MGC:BA0314~BA695900~RFU:60062.55 |
| Anthrax008~B17R14C09 | 0.036981698 | Ba~MGC:BA4681~BA698031~RFU:6215.05 |
| Anthrax008~B03R15C05 | 0.03698991 | Ba~MGC:BA5407~BA698413~RFU:59933.77 |
| Anthrax008~B11R15C06 | 0.037003007 | Ba~MGC:BA0072~BA695766~RFU:59992.64 |
| Anthrax008~B47R01C17 | 0.037149 | Ba~MGC:BA4023~BA697696~RFU:31791.13 |
| Anthrax008~B24R14C04 | 0.037223633 | Ba~MGC:BA0309~BA695897~RFU:60107.62 |
| Anthrax008~B06R18C07 | 0.037228364 | EF_25n~N/A |
| Anthrax008~B01R06C15 | 0.03731789 | Ba~MGC:BA0246~BA695859~RFU:3115.34 |
| Anthrax008~B30R05C09 | 0.037328035 | Ba~MGC:BA0120~BA695796~RFU:59755.33 |
| Anthrax008~B47R01C20 | 0.037442576 | Ba~MGC:BA4022~BA697695~RFU:19238.91 |
| Anthrax008~B40R08C04 | 0.037559964 | Ba~MGC:BA3859~BA697600~RFU:1862.58 |
| Anthrax008~B31R15C09 | 0.03781694 | Ba~MGC:BA1445.1~BA696434~RFU:10131.53 |
| Anthrax008~B40R04C10 | 0.037831258 | Ba~MGC:BA5102~BA698255~RFU:59925.5 |
| Anthrax008~B19R08C05 | 0.03790073 | Ba~MGC:BA1499~BA15288~RFU:6117.55 |
| Anthrax008~B33R12C12 | 0.0379015 | Ba~MGC:BA0483~BA0483~RFU:21314.74 |
| Anthrax008~B48R13C10 | 0.03792478 | Ba~MGC:BA5327~BA15265~RFU:241.77 |
| Anthrax008~B06R18C05 | 0.03804239 | EF_50n~N/A |
| Anthrax008~B34R02C05 | 0.03813004 | Ba~MGC:BA0067~BA695763~RFU:34237.49 |
| Anthrax008~B24R08C01 | 0.038166765 | Ba~MGC:BA5395~BA698404~RFU:3032.9 |
| Anthrax008~B48R04C19 | 0.03818213 | Ba~MGC:BA2188~BA696803~RFU:29303.29 |
| Anthrax008~B17R06C10 | 0.038353942 | Ba~MGC:BA0728~BA696104~RFU:55333.89 |
| Anthrax008~B37R16C19 | 0.03840986 | Ba~MGC:BA5534~BA698473~RFU:14529.33 |
| Anthrax008~B23R12C16 | 0.038479306 | Ba~MGC:BA3231~BA3231~RFU:6947.07 |
| Anthrax008~B09R05C16 | 0.038511258 | Ba~MGC:BA2945~BA697162~RFU:59958.61 |
| Anthrax008~B09R14C20 | 0.038522217 | Ba~MGC:BA4553~BA697972~RFU:30623.75 |
| Anthrax008~B37R03C02 | 0.03860019 | Ba~MGC:BA3303~BA697325~RFU:35273.25 |
| Anthrax008~B08R07C18 | 0.03864383 | Ba~MGC:BA0887~BA696183~RFU:2103.57 |
| Anthrax008~B12R12C05 | 0.038881678 | Ba~MGC:BA3589~BA13916~RFU:60082.78 |
| Anthrax008~B14R11C18 | 0.038901173 | Ba~MGC:BA0906~BA0906~RFU:11519.31 |
| Anthrax008~B06R05C03 | 0.03899117 | Ba~MGC:BA5318~BA698363~RFU:46396.25 |
| Anthrax008~B12R14C08 | 0.039039608 | Ba~MGC:BA4091~BA697729~RFU:336.64 |
| Anthrax008~B11R11C07 | 0.03938578 | Ba~MGC:BA5428~BA13268~RFU:22809.05 |
| Anthrax008~B03R03C02 | 0.0395444 | Ba~MGC:BA2995~BA697189~RFU:60054.27 |
| Anthrax008~B47R07C06 | 0.03975219 | Ba~MGC:BA3537~BA697446~RFU:60133.37 |
| Anthrax008~B15R04C02 | 0.039791744 | Ba~MGC:BA1275~BA696360~RFU:49965.67 |
| Anthrax008~B42R14C10 | 0.039980084 | Ba~MGC:BA1313~BA696375~RFU:166.48 |
| Anthrax008~B17R08C20 | 0.04013237 | Ba~MGC:BA1437~BA14262~RFU:845.29 |
| Anthrax008~B46R04C02 | 0.04022623 | Ba~MGC:BA2031~BA696728~RFU:59933.77 |
| Anthrax008~B46R06C04 | 0.040320717 | Ba~MGC:BA1418~BA696420~RFU:60046.91 |
| Anthrax008~B35R14C03 | 0.040337738 | Ba~MGC:BXA0085~BA698611~RFU:15662.37 |
| Anthrax008~B27R05C19 | 0.040437113 | Ba~MGC:BA1615~BA696524~RFU:59626.61 |
| Anthrax008~B13R04C02 | 0.040455054 | Ba~MGC:BA0461~BA695981~RFU:60032.19 |
| Anthrax008~B33R13C19 | 0.0404746 | Ba~MGC:BXA0005~BA698578~RFU:513.59 |
| Anthrax008~B15R05C20 | 0.040498618 | Ba~MGC:BA3566~BA697461~RFU:60079.1 |
| Anthrax008~B21R14C13 | 0.04056972 | Ba~MGC:BA0114~BA13534~RFU:60113.13 |
| Anthrax008~B18R10C07 | 0.04062726 | Ba~MGC:BA4437~BA13780~RFU:6479.03 |
| Anthrax008~B15R04C07 | 0.040749114 | Ba~MGC:BA1182~BA696306~RFU:60078.18 |
| Anthrax008~B09R08C05 | 0.040791918 | Ba~MGC:BA0995~BA15772~RFU:7423.46 |
| Anthrax008~B32R10C08 | 0.04083115 | Ba~MGC:BA4524~BA16238~RFU:4280.4 |
| Anthrax008~B43R05C08 | 0.040841345 | Ba~MGC:BA1719~BA696573~RFU:60016.56 |
| Anthrax008~B10R13C10 | 0.040880572 | Ba~MGC:BA5061~BA5061~RFU:14127.32 |
| Anthrax008~B19R06C13 | 0.040886108 | Ba~MGC:BA0596~BA696041~RFU:706.72 |
| Anthrax008~B26R11C11 | 0.04091286 | Ba~MGC:BXA0011~BA17108~RFU:3519.31 |
| Anthrax008~B23R02C18 | 0.041070085 | Ba~MGC:BA0314~BA695900~RFU:60057.95 |
| Anthrax008~B21R16C15 | 0.041071385 | Ba~MGC:BA1075~BA1075~RFU:9720.38 |
| Anthrax008~B19R03C05 | 0.041087735 | Ba~MGC:BA3390~BA697369~RFU:60040.47 |
| Anthrax008~B33R16C17 | 0.041178606 | Ba~MGC:BA0509~BA0509~RFU:6341.92 |
| Anthrax008~B21R14C18 | 0.04120507 | Ba~MGC:BA2358~BA696897~RFU:6370.49 |
| Anthrax008~B42R05C17 | 0.041241694 | Ba~MGC:BA5737~BA698575~RFU:60077.26 |
| Anthrax008~B16R14C16 | 0.041401017 | Ba~MGC:BA2529~BA698781~RFU:60091.06 |
| Anthrax008~B11R03C03 | 0.04173854 | Ba~MGC:BA2450~BA696942~RFU:60040.47 |
| Anthrax008~B34R10C14 | 0.041759655 | Ba~MGC:BXB0096~BA14624~RFU:7580.83 |
| Anthrax008~B04R04C04 | 0.041807193 | Ba~MGC:BA2778~BA697080~RFU:59989.88 |
| Anthrax008~B33R09C20 | 0.04191656 | Ba~MGC:BA3763~BA13228~RFU:5527.04 |
| Anthrax008~B48R15C10 | 0.04195295 | Ba~MGC:BA5179~BA698293~RFU:0 |
| Anthrax008~B09R14C13 | 0.04196346 | Ba~MGC:BA2986~BA15032~RFU:60067.14 |
| Anthrax008~B08R14C09 | 0.042077295 | Ba~MGC:BA1869~BA13433~RFU:47793.41 |
| Anthrax008~B40R06C11 | 0.04210461 | Ba~MGC:BA5121~BA698264~RFU:0 |
| Anthrax008~B26R10C19 | 0.042331245 | Ba~MGC:BXA0196~BA14076~RFU:8632.27 |
| Anthrax008~B23R08C03 | 0.04233243 | Ba~MGC:BA4307~BA697835~RFU:2684.88 |
| Anthrax008~B28R03C03 | 0.042334985 | Ba~MGC:BA3943~BA697648~RFU:52321.89 |
| Anthrax008~B33R13C20 | 0.04251769 | Ba~MGC:BXA0005~BA698578~RFU:566.59 |
| Anthrax008~B03R08C17 | 0.042659063 | Ba~MGC:BA1406~BA14404~RFU:7805.37 |
| Anthrax008~B28R06C01 | 0.042759374 | Ba~MGC:BA4526~BA697957~RFU:60096.58 |
| Anthrax008~B34R06C09 | 0.042767495 | Ba~MGC:BA4650~BA698017~RFU:45762.51 |
| Anthrax008~B18R14C11 | 0.042801797 | Ba~MGC:BA5477~BA698825~RFU:165.56 |
| Anthrax008~B35R14C06 | 0.042876415 | Ba~MGC:BA2916~BA697144~RFU:33222.04 |
| Anthrax008~B04R07C07 | 0.04299707 | Ba~MGC:BA5412~BA698416~RFU:33875.09 |
| Anthrax008~B01R06C17 | 0.04301678 | Ba~MGC:BA3974~BA697669~RFU:60119.57 |
| Anthrax008~B24R16C04 | 0.043042656 | Ba~MGC:BA0272~BA695876~RFU:32422.03 |
| Anthrax008~B05R02C12 | 0.043058652 | Ba~MGC:BA0127~BA695799~RFU:50786.42 |
| Anthrax008~B11R03C04 | 0.04309465 | Ba~MGC:BA2450~BA696942~RFU:60041.39 |
| Anthrax008~B23R02C09 | 0.043167606 | Ba~MGC:BA1287~BA696366~RFU:34466.52 |
| Anthrax008~B20R03C09 | 0.043222424 | Ba~MGC:BA3981~BA697674~RFU:44579.4 |
| Anthrax008~B39R12C09 | 0.04335558 | Ba~MGC:BA3897~BA3897~RFU:32684.88 |
| Anthrax008~B03R04C14 | 0.043362007 | Ba~MGC:BA4299~BA697831~RFU:60029.43 |
| Anthrax008~B20R06C06 | 0.04338142 | Ba~MGC:BA4817~BA698102~RFU:60052.43 |
| Anthrax008~B03R05C04 | 0.04363032 | Ba~MGC:BA5141~BA698273~RFU:60067.14 |
| Anthrax008~B08R14C06 | 0.043645732 | Ba~MGC:BXB0014~BA15568~RFU:47525.04 |
| Anthrax008~B14R14C02 | 0.043649476 | Ba~MGC:BA2769~BA14444~RFU:60093.82 |
| Anthrax008~B39R05C19 | 0.04380515 | Ba~MGC:BA4264~BA697811~RFU:59979.76 |
| Anthrax008~B25R10C04 | 0.04389747 | Ba~MGC:BA3489~BA14022~RFU:17178.07 |
| Anthrax008~B47R02C11 | 0.044234853 | Ba~MGC:BA0688~BA696084~RFU:41440.63 |
| Anthrax008~B47R07C03 | 0.044329513 | Ba~MGC:BA4556.1~BA697973~RFU:60104.86 |
| Anthrax008~B48R05C11 | 0.044349674 | Ba~MGC:BA0049~BA695754~RFU:60009.2 |
| Anthrax008~B39R10C19 | 0.044399753 | Ba~MGC:BA5227~BA12796~RFU:24222.77 |
| Anthrax008~B01R14C19 | 0.044406787 | Ba~MGC:BA4440~BA697915~RFU:58899.01 |
| Anthrax008~B42R06C09 | 0.0444639 | Ba~MGC:BA4372~BA697879~RFU:17110.16 |
| Anthrax008~B28R11C12 | 0.044659164 | Ba~MGC:BXA0035~BA17214~RFU:8199.04 |
| Anthrax008~B13R04C01 | 0.044761218 | Ba~MGC:BA0461~BA695981~RFU:60038.63 |
| Anthrax008~B48R15C05 | 0.044775058 | Ba~MGC:BA1712~BA696568~RFU:1146.06 |
| Anthrax008~B19R05C20 | 0.04487749 | Ba~MGC:BA0016~BA695734~RFU:60070.82 |
| Anthrax008~B18R12C14 | 0.04500201 | Ba~MGC:BXA0119~BA13500~RFU:33982.71 |
| Anthrax008~B25R08C20 | 0.04516428 | Ba~MGC:BA0973~BA13544~RFU:1552.61 |
| Anthrax008~B09R04C19 | 0.04520215 | Ba~MGC:BA3420~BA697385~RFU:31703.86 |
| Anthrax008~B44R10C10 | 0.045241337 | Ba~MGC:BA4073~BA14818~RFU:35279.62 |
| Anthrax008~B02R06C05 | 0.04526088 | Ba~MGC:BA2136~BA696779~RFU:36301.51 |
| Anthrax008~B01R08C19 | 0.045418784 | Ba~MGC:BA1122~BA13778~RFU:4638.52 |
| Anthrax008~B31R10C18 | 0.04557197 | Ba~MGC:BA5208~BA13390~RFU:4523.55 |
| Anthrax008~B43R03C08 | 0.045578424 | Ba~MGC:BA4084~BA697724~RFU:60053.35 |
| Anthrax008~B29R10C17 | 0.045688804 | Ba~MGC:BA5194~BA14092~RFU:2443.49 |
| Anthrax008~B36R12C14 | 0.045695413 | Ba~MGC:BXB0002~BA13943~RFU:32771.34 |
| Anthrax008~B31R10C16 | 0.04583505 | Ba~MGC:BA5137~BA13404~RFU:13550.4 |
| Anthrax008~B48R08C06 | 0.045938116 | Ba~MGC:BA2500~BA14596~RFU:0 |
| Anthrax008~B01R01C20 | 0.046226174 | Ba~MGC:BA3092~BA697243~RFU:60121.41 |
| Anthrax008~B48R10C03 | 0.04645406 | Ba~MGC:BA5228~BA14098~RFU:1227.01 |
| Anthrax008~B24R16C03 | 0.046456277 | Ba~MGC:BA0272~BA695876~RFU:28806.87 |
| Anthrax008~B24R06C10 | 0.046624605 | Ba~MGC:BA4593~BA697989~RFU:60067.14 |
| Anthrax008~B29R10C16 | 0.046758097 | Ba~MGC:BA5144~BA14378~RFU:1198.86 |
| Anthrax008~B18R02C11 | 0.04689081 | Ba~MGC:BA0462~BA695982~RFU:60069.9 |
| Anthrax008~B01R14C20 | 0.04698714 | Ba~MGC:BA4440~BA697915~RFU:60042.31 |
| Anthrax008~B32R10C07 | 0.047004454 | Ba~MGC:BA4524~BA16238~RFU:4443.49 |
| Anthrax008~B16R09C18 | 0.047120757 | Ba~MGC:BA4971~BA13890~RFU:2877.12 |
| Anthrax008~B28R10C08 | 0.04729873 | Ba~MGC:BA3487~BA15100~RFU:10675.13 |
| Anthrax008~B03R04C13 | 0.047369577 | Ba~MGC:BA4299~BA697831~RFU:60047.83 |
| Anthrax008~B02R03C01 | 0.04749552 | Ba~MGC:BA4359~BA697872~RFU:43159.49 |
| Anthrax008~B14R05C15 | 0.04749925 | Ba~MGC:BA1373~BA696403~RFU:0 |
| Anthrax008~B24R04C12 | 0.047575783 | Ba~MGC:BA4162~BA697762~RFU:59972.41 |
| Anthrax008~B32R12C11 | 0.047618054 | Ba~MGC:BA2091~BA14006~RFU:20261.22 |
| Anthrax008~B13R04C12 | 0.04769753 | Ba~MGC:BA1485~BA696454~RFU:24301.86 |
| Anthrax008~B35R04C14 | 0.047732912 | Ba~MGC:BA5574~BA698492~RFU:42716.15 |
| Anthrax008~B08R03C18 | 0.047813877 | Ba~MGC:BA2363~BA696898~RFU:60037.71 |
| Anthrax008~B39R10C15 | 0.047839522 | Ba~MGC:BA5086~BA15868~RFU:3803.35 |
| Anthrax008~B09R04C20 | 0.04785985 | Ba~MGC:BA3420~BA697385~RFU:33341.92 |
| Anthrax008~B22R14C14 | 0.047878742 | Ba~MGC:BA1434~BA15844~RFU:60102.1 |
| Anthrax008~B36R10C08 | 0.0480084 | Ba~MGC:BA3475~BA13908~RFU:10081.55 |
| Anthrax008~B11R10C01 | 0.048118167 | Ba~MGC:BA3951~BA13826~RFU:9915.59 |
| Anthrax008~B25R01C17 | 0.048141763 | Ba~MGC:BA0834~BA696155~RFU:4139.07 |
| Anthrax008~B35R15C13 | 0.048197813 | Ba~MGC:BA3849~BA16486~RFU:26422.92 |
| Anthrax008~B10R03C18 | 0.048205387 | Ba~MGC:BA3608~BA697481~RFU:57984.73 |
| Anthrax008~B14R06C19 | 0.04824575 | Ba~MGC:BA0631.1~BA696057~RFU:57388.7 |
| Anthrax008~B15R12C05 | 0.04824601 | Ba~MGC:BA3327~BA13237~RFU:22152.32 |
| Anthrax008~B04R16C02 | 0.048336856 | Ba~MGC:BA1484~BA696453~RFU:44928.26 |
| Anthrax008~B30R06C09 | 0.048389092 | Ba~MGC:BA4952~BA698181~RFU:4473.88 |
| Anthrax008~B42R04C10 | 0.048452903 | Ba~MGC:BA1600~BA696516~RFU:60023.91 |
| Anthrax008~B44R14C14 | 0.048558123 | Ba~MGC:BA3827~BA3827~RFU:2241.54 |
| Anthrax008~B38R11C20 | 0.04873423 | Ba~MGC:BA0134~BA12564~RFU:15288.98 |
| Anthrax008~B21R05C14 | 0.04875782 | Ba~MGC:BA5515~BA698465~RFU:60077.26 |
| Anthrax008~B14R04C14 | 0.048848614 | Ba~MGC:BA1582~BA696508~RFU:60024.83 |
| Anthrax008~B39R05C09 | 0.04893439 | Ba~MGC:BA1487~BA696455~RFU:60084.62 |
| Anthrax008~B40R13C15 | 0.049011424 | Ba~MGC:BA5245~BA5245~RFU:3054.64 |
| Anthrax008~B08R02C02 | 0.04901647 | Ba~MGC:BA2383~BA696907~RFU:0 |
| Anthrax008~B25R02C17 | 0.049220756 | Ba~MGC:BA5136~BA698270~RFU:28145.92 |
| Anthrax008~B13R05C17 | 0.049344733 | Ba~MGC:BA2038.1~BA696732~RFU:59818.8 |
| Anthrax008~B43R08C19 | 0.049391482 | Ba~MGC:BA1381~BA14660~RFU:7571.74 |
| Anthrax008~B09R06C05 | 0.04941597 | Ba~MGC:BA2625~BA697012~RFU:59617.37 |
| Anthrax008~B18R11C03 | 0.049426466 | Ba~MGC:BXA0006~BA15174~RFU:1947.2 |
| Anthrax008~B14R10C09 | 0.049426883 | Ba~MGC:BA5311~BA14666~RFU:5215.23 |
| Anthrax008~B05R05C12 | 0.049609236 | Ba~MGC:BA1672~BA696552~RFU:58077.63 |
| Anthrax008~B18R02C12 | 0.04984608 | Ba~MGC:BA0462~BA695982~RFU:60079.1 |
| Anthrax008~B41R06C05 | 0.049867637 | Ba~MGC:BA0420~BA695963~RFU:54978.54 |
