# Supplementary material for: Whole genome protein microarrays for serum profiling of immunodominant antigens of Bacillus anthracis
Source: Front Microbiol. 2015 Aug 13;6:747. doi: 10.3389/fmicb.2015.00747 (PMC4534840; doi:10.3389/fmicb.2015.00747)
Supplement: Supplementary file 4 [file DataSheet4.DOCX]

Supplementary Information S4  **(A)**

**(i) (ii) (iii) (iv)**


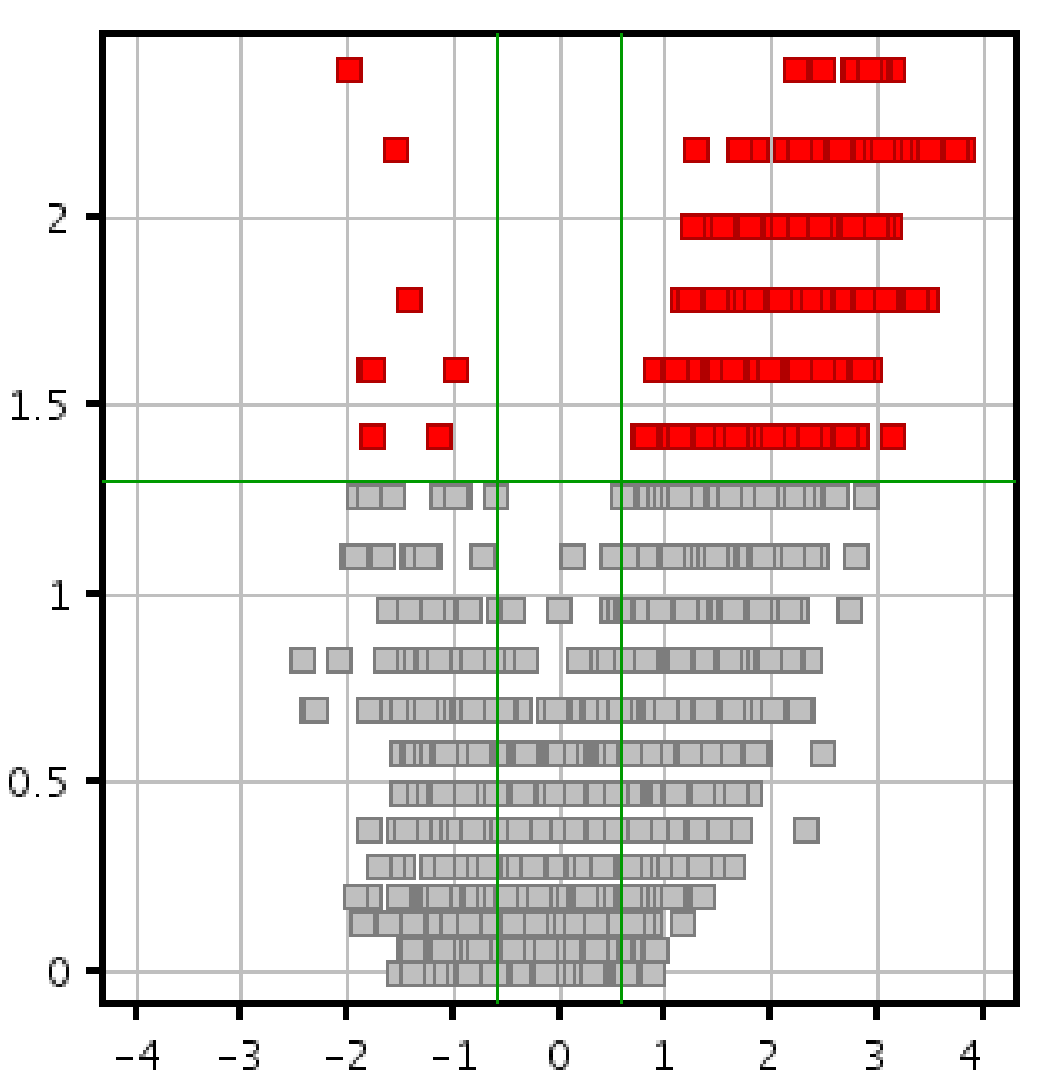

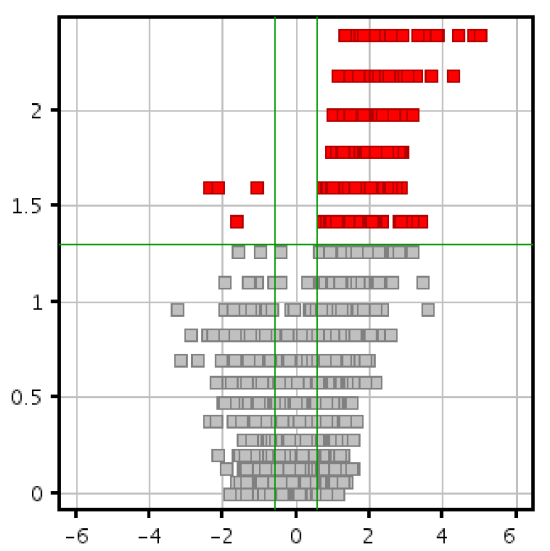

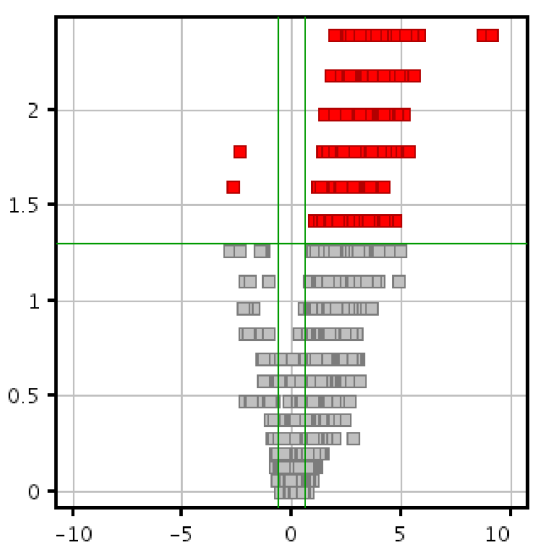

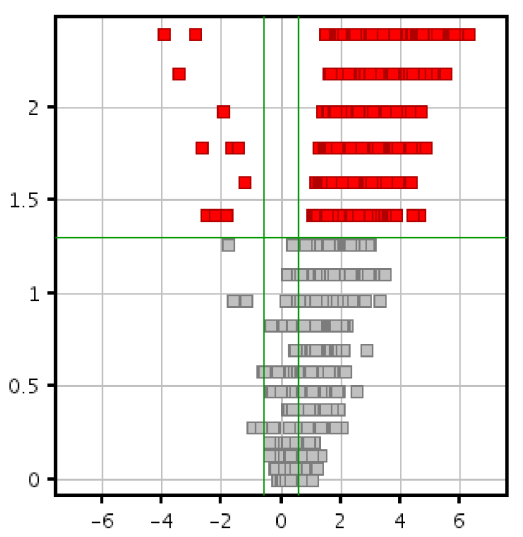


(e)

(b)

(c)

(a)

**(v) (vi)**

(d)

(a)


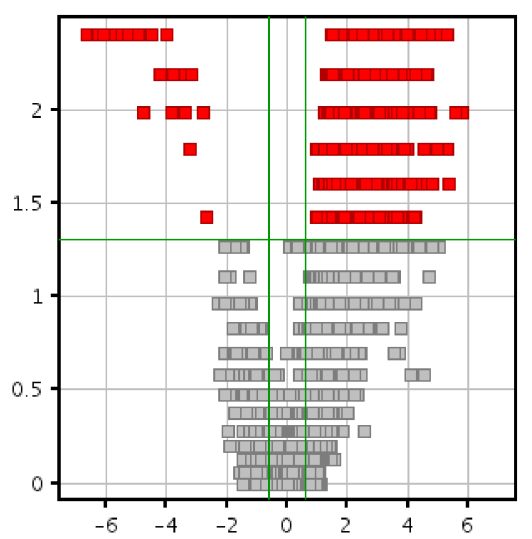


(c)

(d)

(b)


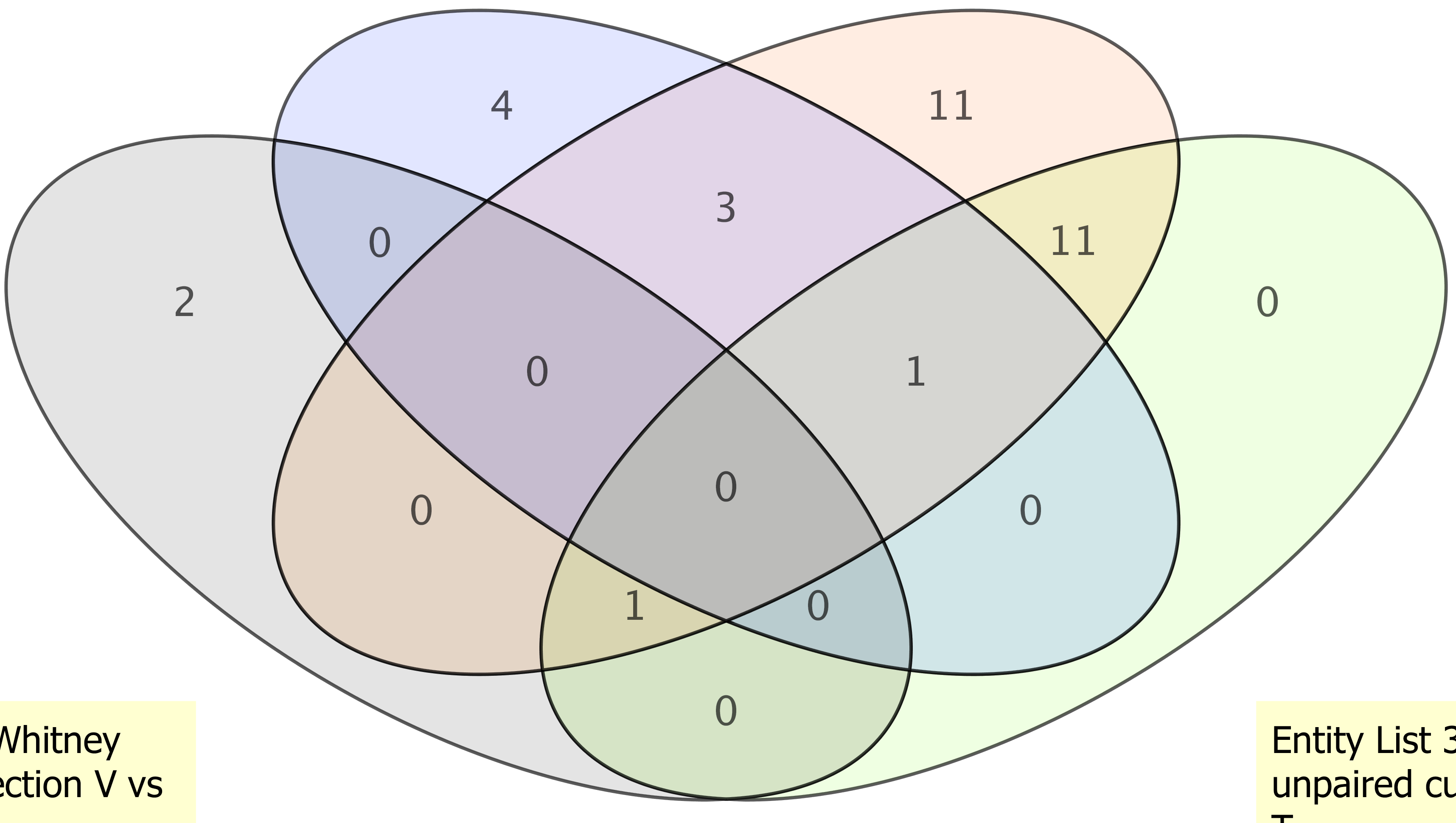


Mann Witney U test analyses (unpaired, no multiple testing correction, at a fold-change cut off >1.5 and p ≤ 0.05) of the comparison between control and test groups for IgG recognition of *B. anthracis* proteins on the array. (i) Control vs BWS (ii) Control vs AN IVDU (iii) Control vs AP IVDU (iv) Control vs TCA (v) Control vs AVPV (vi) Venn diagram depiction of shared and unique entities between the control and three infected or vaccinated groups (* represents where only one of two duplicate entities are represented) (v) list of *B. anthracis* protein entities (excluding toxin components) unique to the AP IVDA and BWS groups. All group specific and shared entity features depicted in Figure A (vi) are listed below in Table 1.

**(B)**

**(i) (ii) (iii) (iv)**


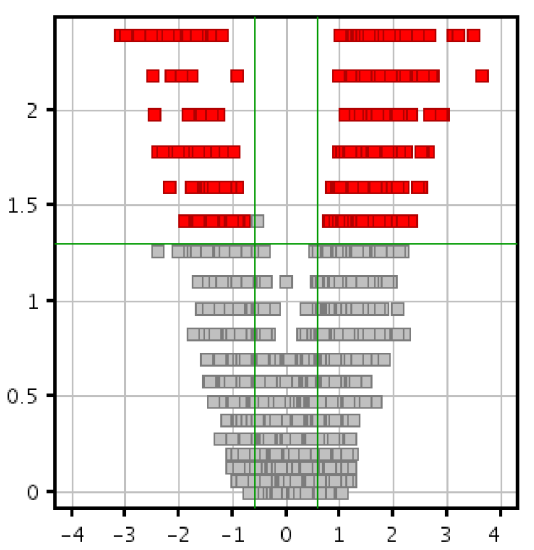

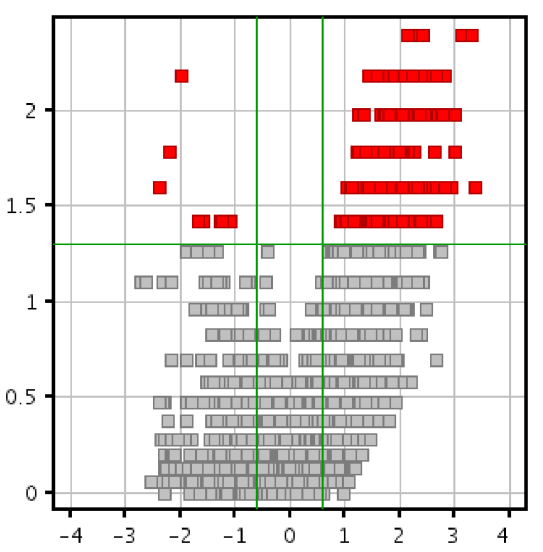

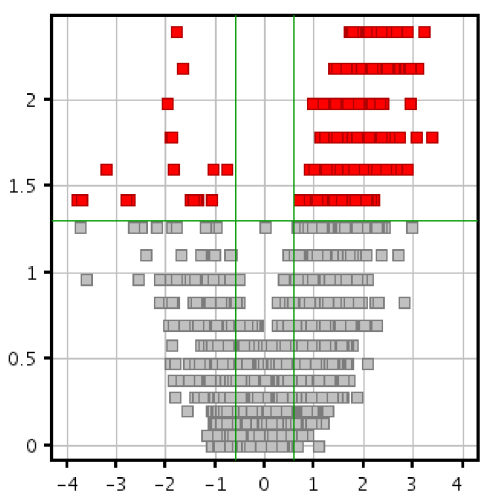

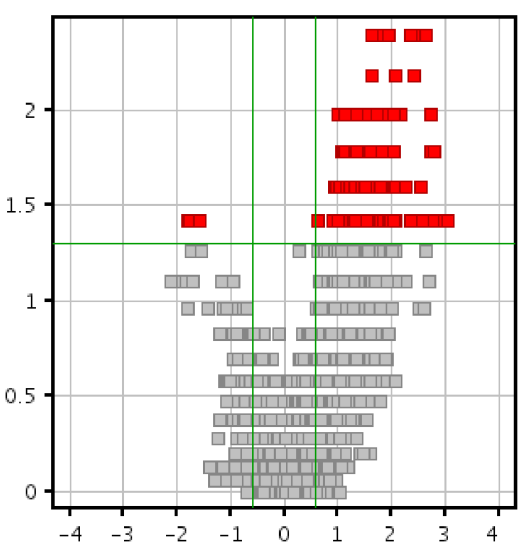


(e)

(c)

(b)

(a)

**(v) (vi)**

(d)

(a)


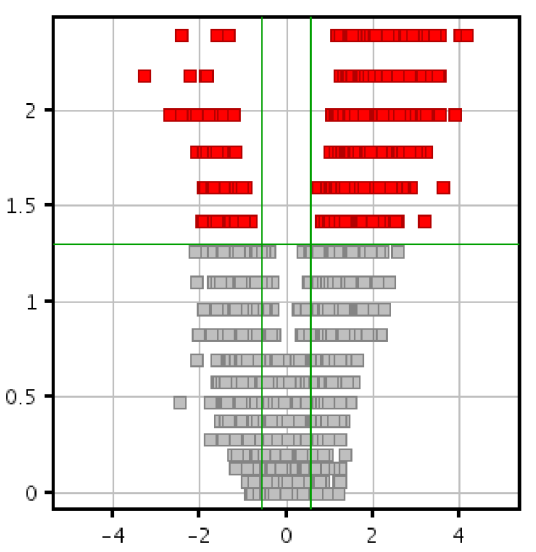


(d)

(c)

(b)


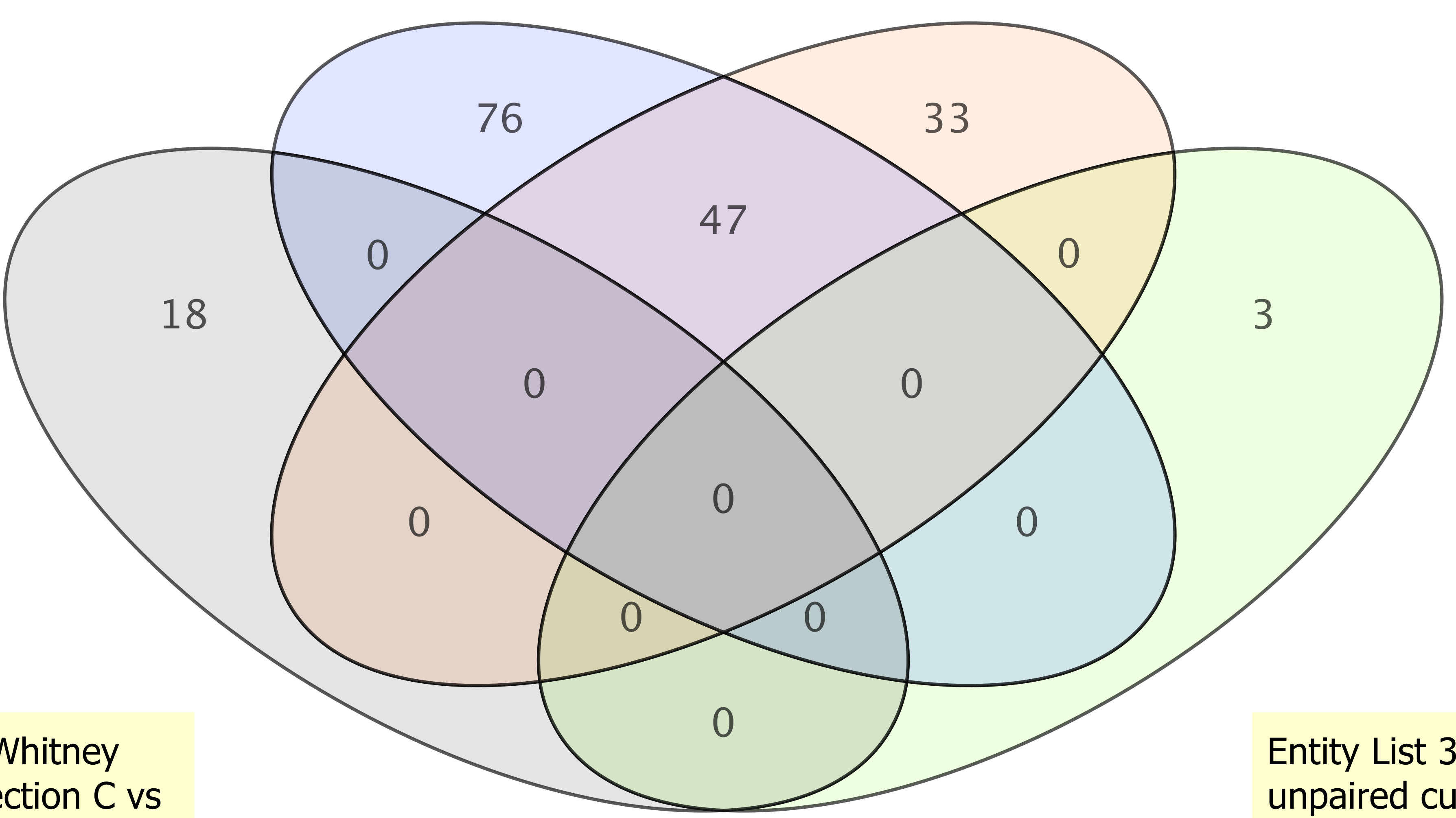


Mann Witney U test analyses (unpaired, no multiple testing correction, at a fold-change cut off >1.5 and p ≤ 0.05) of the comparison between control and test groups for IgA recognition of *B. anthracis* proteins on the array. (i) Control vs BWS (ii) Control vs AN IVDU (iii) Control vs AP IVDU (iv) Control vs TCA (v) Control vs AVPV (vi) Venn diagram depiction of shared and unique entities between the control and three infected groups All group-specific and shared entity features depicted in Figure B (vi) are listed below in Table 2.

**Table 1**

| **Group Specificity** | **Protein**  **Name** | **Venn Diagram Segment Identifier** | **P value** | **Group Specificity** | **Protein**  **Name** | **Venn Diagram Segment Identifier** | **P value** |
| --- | --- | --- | --- | --- | --- | --- | --- |
| AP IVDU | BA4182***** | (b) | 1.63 x 10^-2^ | AN IVDU | BA2377***** | (e) | 2.50 x 10^-2^ |
| AP IVDU/ TCA/AVPV | PA 200 ng/ul | (b)/(c)/(d) | 2.5 x 10^-2^ /2.5 x 10^-2^ /1.03 x 10^-2^ | AVPV/TCA | LF 1.5625 ng/ul | (c)/(d) | 3.88 x 10^-3^ |
| BWS | BA0973 | (a) | 2.5 x 10^-2^ | AVPV/TCA | LF 0.78125 ng/ul | (c)/(d) | 3.88 x 10^-3^ |
| BWS | BA1930 | (a) | 2.5 x 10^-2^ | AVPV/TCA | PA 100 ng/ul | (c)/(d) | 3.88 x 10^-3^ |
| BWS/ AN IVDU | BA2389 | (a)/(e) | 2.5 x 10^-2^/3.74 x 10^-2^ | AVPV/TCA | PA 50 ng/ul | (c)/(d) | 3.88 x 10^-3^ |
| BWS | PA .0.390625 ng/ul | (a) | 2.5 x 10^-2^ | AVPV/TCA | PA 25 ng/ul | (c)/(d) | 3.88 x 10^-3^ |
| BWS/AVPV | PA 6.25 ng/ul | (a)/(d) | 3.95 x 10^-3^/3.88 x 10^-3^ | AVPV | PA 1.5625 ng/ul | (d) | 1.03 x 10-2 |
| BWS/AVPV | PA 12.5 ng/ul | (a)/(d) | 3.74 x 10^-2^/3.88 x 10^-3^ | AVPV | PA 3.125 ng/ul | (d) | 6.39 x 10^-3^ |
| BWS/AVPV | LF .0.390625 ng/ul | (a)/(d) | 6.49 x 10^-3^/3.88 x 10^-3^ | AVPV | EF 100 ng/ul | (d) | 3.70 x 10^-2^ |
| BWS/TCA/AVPV | LF .0.390625 ng/ul | (a)/(c)/(d) | 1.63 x 10^-2^/6.49 x 10^-3^ /3.88 x 10^-3^ | AVPV | EF 50 ng/ul | (d) | 1.61 x 10^-2^ |
| AVPV/TCA | LF 200 ng/ul | (c)/(d) | 1.03 x 10^-2^ | AVPV | EF 25 ng/ul | (d) | 1.03 x 10^-2^ |
| AVPV/TCA | LF 100 ng/ul | (c)/(d) | 6.39 x 10^-3^ | AVPV | EF 12.5 ng/ul | (d) | 3.88 x 10^-3^ |
| AVPV/TCA | LF 50 ng/ul | (c)/(d) | 6.39 x 10^-3^ | AVPV | EF 6.25 ng/ul | (d) | 3.88 x 10^-3^ |
| AVPV/TCA | LF 25 ng/ul | (c)/(d) | 6.39 x 10^-3^ | AVPV | EF 3.125 ng/ul | (d) | 6.39 x 10^-3^/1.03 x 10^-2^ |
| AVPV/TCA | LF 12.5 ng/ul | (c)/(d) | 3.88 x 10^-3^ | AVPV | EF 1.5625 ng/ul | (d) | 3.88 x 10^-3^/1.03 x 10^-2^ |
| AVPV/TCA | LF 6.25 ng/ul | (c)/(d) | 3.88 x 10^-3^ | AVPV | EF 0.78125 ng/ul | (d) | 3.88 x 10^-3^ |
| AN IVDU | BA5591 | (e) | 2.50 x 10^-2^ | AVPV | EF 0.390625 ng/ul | (d) | 3.88 x 10^-3^ |

(***** represents where all replicates for those entity features are represented in the group sectors)

(* represents where only one of two duplicate entities are represented)

Table 2

| **Group Specificity** | **Protein**  **Name** | **Venn Diagram Segment Identifier** | **P value** | **Group Specificity** | **Protein**  **Name** | **Venn Diagram Segment Identifier** | **P value** |
| --- | --- | --- | --- | --- | --- | --- | --- |
| AP IVDU | BA1147***** | (b) | 3.74E-02/2.50E-02 | BWS/AVPV | BA3391 | (a)/(d) | 3.95E-03/6.49E-03 |
| AP IVDU | BA1289***** | (b) | 3.95E-03/6.49E-03 | BWS/AVPV | BA3694 | (a)/(d) | 1.04E-02/2.50E-02 |
| AP IVDU | BA1554***** | (b) | 1.63E-02/1.63E-02 | BWS/AVPV | BA3818 | (a)/(d) | 3.95E-03/6.49E-03 |
| AP IVDU | BA3041***** | (b) | 3.74E-02/3.74E-02 | BWS/AVPV | BA3839 | (a)/(d) | 2.50E-02/3.74E-02 |
| AP IVDU | BA4182***** | (b) | 3.74E-02/3.74E-02 | BWS/AVPV | BA3990***** | (a)/(d) | 6.49E-03/3.95E-03 |
| AP IVDU | BA4334***** | (b) | 3.74E-02/3.74E-02 | BWS/AVPV | BA4124 | (a)/(d) | 2.50E-02/1.04E-02 |
| AP IVDU | BA4778***** | (b) | 3.74E-02/3.74E-02 | BWS/AVPV | BA4779 | (a)/(d) | 2.50E-02/1.63E-02 |
| AP IVDU | BA4844***** | (b) | 1.04E-02/2.50E-02 | BWS/AVPV | BA5051***** | (a)/(d) | 6.49E-03/1.63E-02 |
| AP IVDU | BA5355 | (b) | 2.50E-02 | BWS/AVPV | BA5684 | (a)/(d) | 6.49E-03/3.74E-02 |
| AP IVDU | PA 50ng/ul | (b) | 2.50E-02 | BWS/AVPV | BXA0026 | (a)/(d) | 3.74E-02/3.74E-02 |
| TCA | BA2967 | (c) | 3.74E-02 | BWS | BA0483 | (a) | 3.74E-02 |
| TCA | BA3953 | (c) | 3.74E-02/3.74E-02 | BWS | BA0639 | (a) | 1.63E-02 |
| AVPV | BA0107 | (d) | 2.50E-02 | BWS | BA0771 | (a) | 1.04E-02 |
| AVPV | BA0126 | (d) | 3.74E-02 | BWS | BA0936***** | (a) | 2.50E-02/3.74E-02 |
| AVPV | BA0172 | (d) | 3.74E-02 | BWS | BA1013 | (a) | 3.95E-03 |
| AVPV | BA1048 | (d) | 3.74E-02 | BWS | BA1044 | (a) | 3.95E-03 |
| AVPV | BA1644 | (d) | 3.74E-02 | BWS | BA1135 | (a) | 1.63E-02 |
| AVPV | BA2256 | (d) | 3.74E-02 | BWS | BA1166 | (a) | 3.74E-02 |
| AVPV | BA2276 | (d) | 2.50E-02 | BWS | BA1242 | (a) | 2.50E-02 |
| AVPV | BA2287 | (d) | 2.50E-02 | BWS | BA1258 | (a) | 3.74E-02 |
| AVPV | BA2402 | (d) | 3.95E-03 | BWS | BA1284 | (a) | 3.74E-02 |
| AVPV | BA2454 | (d) | 2.50E-02 | BWS | BA1580 | (a) | 3.95E-03 |
| AVPV | BA2482 | (d) | 1.63E-02 | BWS | BA1730 | (a) | 3.74E-02 |
| AVPV | BA2707 | (d) | 2.50E-02 | BWS | BA1733 | (a) | 3.74E-02 |
| AVPV | BA2717 | (d) | 3.74E-02 | BWS | BA1788 | (a) | 3.74E-02 |
| AVPV | BA3258 | (d) | 2.50E-02 | BWS | BA1797 | (a) | 1.63E-02 |
| AVPV | BA3659 | (d) | 2.50E-02 | BWS | BA1877 | (a) | 3.74E-02 |
| AVPV | BA3752 | (d) | 3.74E-02 | BWS | BA2114 | (a) | 1.04E-02 |
| AVPV | BA3842 | (d) | 3.74E-02 | BWS | BA2119 | (a) | 2.50E-02 |
| AVPV | BA3860 | (d) | 3.74E-02 | BWS | BA2187 | (a) | 2.50E-02 |
| AVPV | BA3903 | (d) | 3.74E-02 | BWS | BA2196 | (a) | 3.74E-02 |
| AVPV | BA3918 | (d) | 2.50E-02 | BWS | BA2246 | (a) | 2.50E-02 |
| AVPV | BA3945 | (d) | 2.50E-02 | BWS | BA2344 | (a) | 1.04E-02 |
| AVPV | BA3948 | (d) | 3.74E-02 | BWS | BA2353 | (a) | 3.74E-02 |
| AVPV | BA4015 | (d) | 3.74E-02 | BWS | BA2389 | (a) | 1.63E-02 |
| AVPV | BA4369 | (d) | 3.74E-02 | BWS | BA2500***** | (a) | 1.63E-02/1.63E-02 |
| AVPV | BA4384 | (d) | 2.50E-02 | BWS | BA2541***** | (a) | 2.50E-02/3.74E-02 |
| AVPV | BA4761 | (d) | 3.74E-02 | BWS | BA2554 | (a) | 1.63E-02 |
| AVPV | BA5207 | (d) | 2.50E-02 | BWS | BA2668***** | (a) | 1.63E-02/1.63E-02 |
| AVPV | BA5327***** | (d) | 3.74E-02/2.50E-02 | BWS | BA2691 | (a) | 3.74E-02 |
| AVPV | BA5436 | (d) | 2.50E-02 | BWS | BA2817 | (a) | 1.63E-02 |
| AVPV | BXA0178 | (d) | 2.50E-02 | BWS | BA3088 | (a) | 2.50E-02 |
| AVPV | EF 0.78125 ng/ul | (d) | 3.95E-03 | BWS | BA3136 | (a) | 3.74E-02 |
| AVPV | LF 0.390625 ng/ul | (d) | 3.74E-02 | BWS | BA3209 | (a) | 3.74E-02 |
| BWS/AVPV | BA0107 | (a)/(d) | 1.63E-02/1.63E-02 | BWS | BA3461 | (a) | 3.74E-02 |
| BWS/AVPV | BA1044 | (a)/(d) | 3.74E-02/2.50E-02 | BWS | BA3514 | (a) | 3.74E-02 |
| BWS/AVPV | BA1135 | (a)/(d) | 1.63E-02/1.04E-02 | BWS | BA3580 | (a) | 1.63E-02 |
| BWS/AVPV | BA1292 | (a)/(d) | 2.50E-02/3.74E-02 | BWS | BA3810 | (a) | 3.74E-02 |
| BWS/AVPV | BA1311 | (a)/(d) | 3.95E-03/3.74E-02 | BWS | BA3813 | (a) | 2.50E-02 |
| BWS/AVPV | BA1398 | (a)/(d) | 1.04E-02/1.04E-02 | BWS | BA3844 | (a) | 2.50E-02 |
| BWS/AVPV | BA1458 | (a)/(d) | 3.74E-02/3.74E-02 | BWS | BA3979 | (a) | 3.74E-02 |
| BWS/AVPV | BA1534 | (a)/(d) | 3.95E-03/1.04E-02 | BWS | BA4124 | (a) | 2.50E-02 |
| BWS/AVPV | BA1563 | (a)/(d) | 3.95E-03/6.49E-03 | BWS | BA4326 | (a) | 3.74E-02 |
| BWS/AVPV | BA1788 | (a)/(d) | 6.49E-03/1.04E-02 | BWS | BA4373 | (a) | 2.50E-02 |
| BWS/AVPV | BA1797 | (a)/(d) | 3.95E-03/1.63E-02 | BWS | BA4565 | (a) | 2.50E-02 |
| BWS/AVPV | BA1851 | (a)/(d) | 2.50E-02/2.50E-02 | BWS | BA4672 | (a) | 3.74E-02 |
| BWS/AVPV | BA1929 | (a)/(d) | 3.95E-03/3.74E-02 | BWS | BA4681 | (a) | 3.74E-02 |
| BWS/AVPV | BA1930 | (a)/(d) | 1.04E-02/1.63E-02 | BWS | BA4720 | (a) | 3.74E-02 |
| BWS/AVPV | BA2187 | (a)/(d) | 2.50E-02/2.50E-02 | BWS | BA4722 | (a) | 3.74E-02 |
| BWS/AVPV | BA2196 | (a)/(d) | 1.63E-02/3.74E-02 | BWS | BA4732 | (a) | 3.74E-02 |
| BWS/AVPV | BA2217 | (a)/(d) | 1.63E-02/1.04E-02 | BWS | BA4740 | (a) | 3.74E-02 |
| BWS/AVPV | BA2342 | (a)/(d) | 2.50E-02/1.04E-02 | BWS | BA4769 | (a) | 6.49E-03 |
| BWS/AVPV | BA2353 | (a)/(d) | 2.50E-02/1.63E-02 | BWS | BA4825 | (a) | 3.74E-02 |
| BWS/AVPV | BA2386 | (a)/(d) | 1.63E-02/3.74E-02 | BWS | BA4955***** | (a) | 3.74E-02/3.74E-02 |
| BWS/AVPV | BA2401 | (a)/(d) | 3.74E-02/1.63E-02 | BWS | BA5002 | (a) | 3.95E-03 |
| BWS/AVPV | BA2431 | (a)/(d) | 2.50E-02/1.63E-02 | BWS | BA5135***** | (a) | 3.74E-02/3.74E-02 |
| BWS/AVPV | BA2525 | (a)/(d) | 1.63E-02/2.50E-02 | BWS | BA5144***** | (a) | 3.74E-02/3.74E-02 |
| BWS/AVPV | BA2611 | (a)/(d) | 3.95E-03/6.49E-03 | BWS | BA5179 | (a) | 3.74E-02 |
| BWS/AVPV | BA2626 | (a)/(d) | 3.95E-03/1.04E-02 | BWS | BA5200 | (a) | 2.50E-02 |
| BWS/AVPV | BA2693 | (a)/(d) | 1.63E-02/2.50E-02 | BWS | BA5241 | (a) | 3.74E-02 |
| BWS/AVPV | BA2717 | (a)/(d) | 1.04E-02/1.04E-02 | BWS | BA5255 | (a) | 3.74E-02 |
| BWS/AVPV | BA2738 | (a)/(d) | 3.95E-03/1.04E-02 | BWS | BA5287 | (a) | 3.74E-02 |
| BWS/AVPV | BA2750 | (a)/(d) | 6.49E-03/1.63E-02 | BWS | BA5345 | (a) | 3.74E-02 |
| BWS/AVPV | BA2804 | (a)/(d) | 2.50E-02/1.63E-02 | BWS | BXA0048 | (a) | 3.74E-02 |
| BWS/AVPV | BA2817 | (a)/(d) | 3.95E-03/6.49E-03 | BWS | BXA0060 | (a) | 1.63E-02 |
| BWS/AVPV | BA2884 | (a)/(d) | 3.74E-02/1.04E-02 | BWS | BXA0112 | (a) | 3.74E-02 |
| BWS/AVPV | BA2917 | (a)/(d) | 1.63E-02/2.50E-02 | BWS | BXA0121 | (a) | 3.74E-02 |
| BWS/AVPV | BA2919 | (a)/(d) | 1.04E-02/1.63E-02 | BWS | BXA0169 | (a) | 3.74E-02 |
| BWS/AVPV | BA3321 | (a)/(d) | 2.50E-02/3.74E-02 |  |  |  |  |

(* represents where only one of two duplicate entities are represented)
