# Supplementary material for: Whole genome protein microarrays for serum profiling of immunodominant antigens of Bacillus anthracis
Source: Front Microbiol. 2015 Aug 13;6:747. doi: 10.3389/fmicb.2015.00747 (PMC4534840; doi:10.3389/fmicb.2015.00747)
Supplement: Supplementary file 5 [file DataSheet5.DOCX]

**Supplementary Information S5:** (a) Boxplot graphical depiction of IgG recognition of *B. anthracis* entity BA1482 across all human data sets (b) Boxplot graphical depiction of IgA recognition of entity BA1482 across all human data sets

**Normalised Intensity Values**

**A**


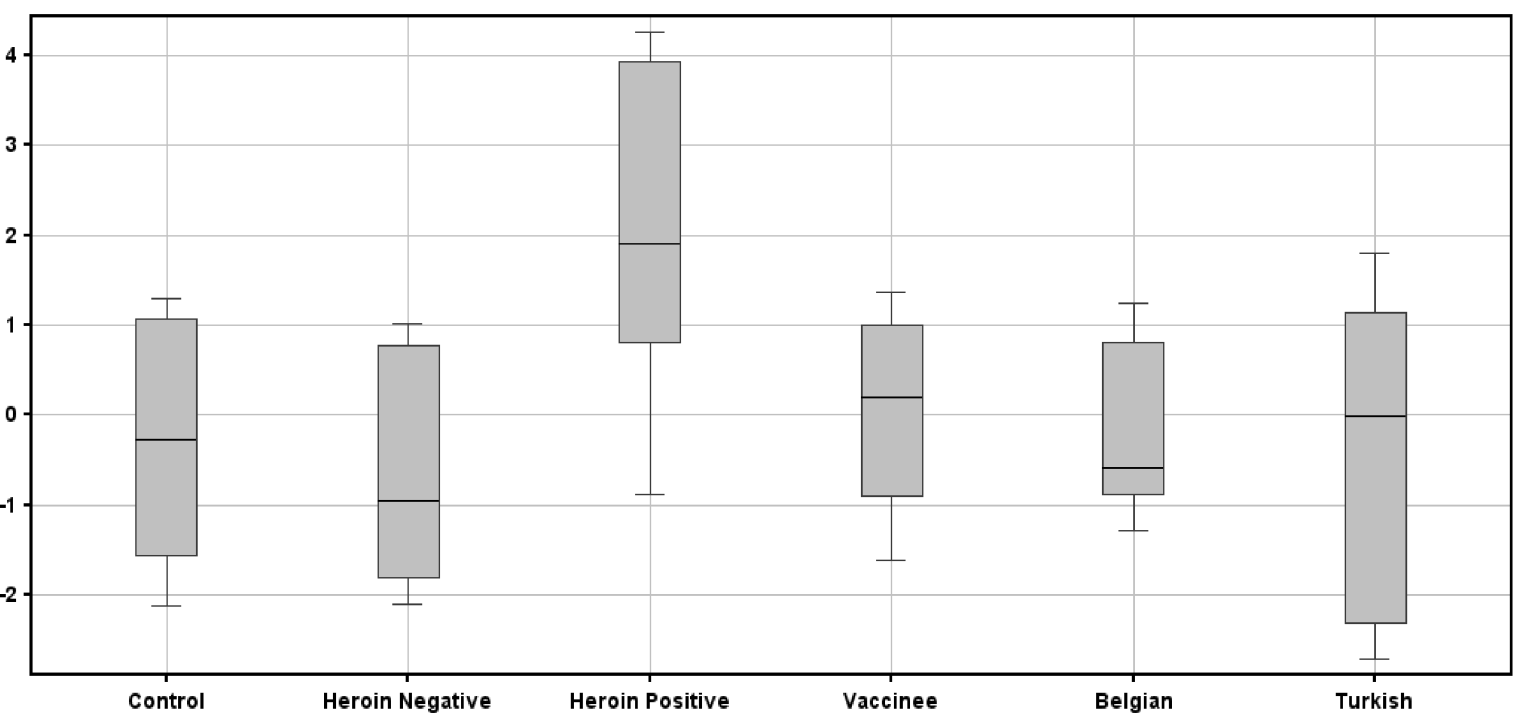


**Control AN IVDU AP IVDU AVPV BWS TCA**

**Group**

**Normalised Intensity Values**

**B**


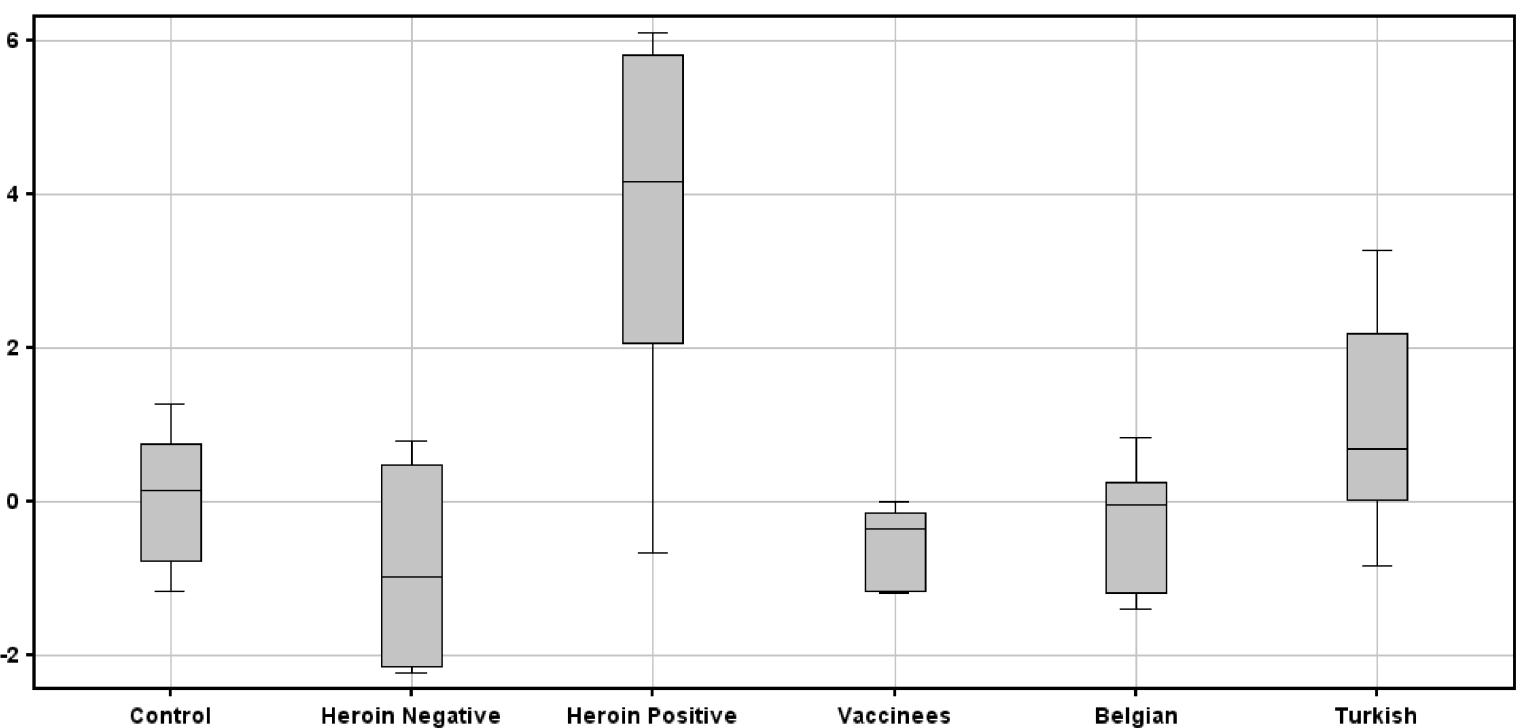


**Control AN IVDU AP IVDU AVPV BWS TCA**

**Group**
