# Supplementary material for: Whole genome protein microarrays for serum profiling of immunodominant antigens of Bacillus anthracis
Source: Front Microbiol. 2015 Aug 13;6:747. doi: 10.3389/fmicb.2015.00747 (PMC4534840; doi:10.3389/fmicb.2015.00747)
Supplement: Supplementary file 6 [file DataSheet6.DOCX]

**Supplementary information S6**

**Table 1** Immunodominant protein entities identified as more highly recognised by sera from either the AVP vaccinated or Belgian Woolsorters groups, with equivalent or greater fluorescence binding intensities to the various toxin components

| **Gene**  **Name** | **Name** | **Peptide Sequence/**  **Alignment to Nearest Neighbour** | **% Conserved Identity** | **Ig Positivity** | **p value**  **Human**  **IgG** | **p value**  **Human**  **IgA** |
| --- | --- | --- | --- | --- | --- | --- |
|  |  |  | **Nearest Neighbour** | **Group** |  |  |
| BA3810 | Conserved domain hypothetical protein (bacillus specific), 46% similar to the *B. thuringiensis* group family transcriptional regulator, autolysin regulatory protein (*ArpU*) | MREIK IPQLNENATKKKVLIAFAI - - YRSF I K------------------ NKNKT-----------KDRIDYINDMNVALQK  M LNIELPVLNKEATKENVLK- -AIKKYRLFMKCNYLNETILSNEN I LKENAKEERINYI I AMNKGLE K  LVNKEDKKIIQEYMLREKVNRFRV I RELN ISEGS YYR I RNKAFYNFAYVFGIAVEK  LD I ESDRI IIQKYL LKNRVNRYEVMKELNLSEGDYYRKRNTAFYNYAYAL GIEVEE | 46 | IgG  IgA | 3.97 x 10^-3^ | 7.21 x 10^-3^ |
|  |  |  | *B. cereus*  WP_000931948.1 | AVP vaccinated humans and rabbits |  |  |
| BA0973 | Hypothetical protein | MILGISYFNIFCIVVIILFFFIFWRLHVWYNKKHNVPKVFQWFPRKWGKRKVSEHLSQLNEKLEAEGK MILGISYFNIFCIVVIILFFFIFWRLHVWYNKKHNVPKVFQWFPRKWGKRKVSEHLSQLNEKLAAEGK  GIWVTIYAIDLYIVTIPCTVKKYKS  GIWVTIYAIDLYIVTIPCTVKKYKS | 99 | IgG  IgA | 3.48 x 10^-3^ | 4.52 x 10^-2^ |
|  |  |  | *B. thuringiensis*  EEM61245.1 | Belgian Woolsorters |  |  |
| BA1930 | 3-oxoacyl-ACP synthase (Bacillus cereus group) | MNIGIEATGVFFPKDVETAVDLSKKTGIPENIIIEKFGLYEKHVADEMMHASDLAIAAAKPILLQVNPQS MNIGIEATGVFFPKDVETAVDLSKKTGIPENIIIEKFGLYEKHVADEMMHASDLAIAAAKPILLQVNPQS  IDVVIYFGSPHKDYHVWSSAPKIQHELGLKNAYAFEIMNVSSCFPIALKVAKDMLYSDNSIENILLVGG  IDVVIYFGSPHKDYHVWSSAPKIQHELGLKNAYAFEIMNVSSCFPIALKVAKDMLYSDNSIENILLVGG  CKESQIVDYDNPRSRFMFNFADGGSAALVKKDAKNGEILGSAIITDGSFHEDVRIPAGGSKQVASYD  CKESQIVDYDNPRSRFMFNFADGGSAALVTKDAKNGEILGSAIITDGSFHEDVRIPAGGSKQVASYD  TVENRQHYIDVIDPNSMKERLDPISIPNFDKVIREALRKSGYTPKDIKVLLPLHTKRSMLIELIQGLGLTE  TVENRQHYIDVIDPNSMKERLDPISIPNFDKVIREALRKSGYTPKDIKVLLPLHTKRSMLIELIQGLGLTE  EQVVYLDHYGHMSALDPCIGLHFANEQGKLQAGDIAVVVSAGTGYTWAATVIRW  EQVVYLDHYGHMSALDPCIGLHF ANEQGKLQAGDIAVVVSAGTGYTWAATVIRW | 99 | IgG | 2.01x 10^-3^ | 6.21 x 10^-3^ |
|  |  |  | *B. cereus*  WP_001020502.1 | Belgian Woolsorters |  |  |

**Table 2** Immunodominant protein entities identified as more highly recognised by sera from either the IVDU Anthrax positive (some also recognised by the IVDU Anthrax negative group) or the Turkish cutaneous Anthrax patient groups, with equivalent or greater fluorescence binding intensities to the various toxin components

| **Gene**  **Name** | **Name** | **Peptide Sequence/**  **Alignment to Nearest Neighbour** | **% Conserved Identity** | **Ig Positivity** | **p value**  **Human**  **IgG** | **p value**  **Human**  **IgA** |
| --- | --- | --- | --- | --- | --- | --- |
|  |  |  | **Nearest Neighbour** | **Group** |  |  |
| **A**: BA0007 | Conserved hypothetical protein (pseudogene, GTG start codon – valine) | C I KH I V LGSN*VSSFHL KMYNV I LPVVMKNPPYKMLKKSFENCYPFIYYLEHGKNYYEL  MHQTHC TWQQ - LS FFF-SSQNV QRY - - LARCYE KSSIQ DAEKKSFENCYPFIYYLEHGKNYYEL  YKVAPFSIQPMLLFYGISQLFKACLLTIDPNYPESTTVLAHGVTTRKRKKQGYQFLEDEVKVQKNGLF  YKVAPFSIQPMLLFYGISQLFKACLLTIDPNYPESTTVLAHGVTTRKRKKQGYQFLEDEVKVQKNGLF  THVAEQLFHMKHLESEKFNMLDLMGNIPELQNLFRYSQRGATLYKIDSPNTNELSFSVNILDRLHMT  THVAEQLFHMKHLESEKFNMLDLMGNIPELQNLFRYSQRGATLYKIDSPNTNELSFSVNILDRLHMT  TERFSRYIESICKHLSIQHVPRKTSASNLLFTAPIQSWNPIYSTPLYYEYLADTYYLPLTTDPRNPKPAL  TERFSRYIESICKHLSIQHVPRKTSASNLLFTAPIQSWNPIYSTPLYYEYLADTYYLPLTNDPRNPKPAL  PELLVHYLLLYNLSMISRYETDWWYDLLGSYGSEDY PFIYQFLTISAQKVPYYISSFLLAEPGLFHGK  PELLVHYLLLYNLSMISRYETDWWYDLLGSYGSEDY PFIYQFLTISAQKVPYYISSFLLAEPGLFHGK | 93 | IgG  IgA | 1.63 x 10^-2^ | 1.04 x 10^-2^ |
|  |  |  |  | Under  represented in AP and AN IVDU |  |  |
| **B**: BA3106 | conserved hypothetical protein, multidrug MFS transporter [Bacillus cereus group] | MKENKKSKKRRIFQVFLLMICSAILYVSYAAYDIWSYRFKANDDVKTDAGIVLGAASWNGKPSPVFKE  RINHAISLYKNGNIKKIIFTGGTKFEAELEEARTARVYAMKQGVKEEDILIETKSLFTEENLKNAKQVGI  ENGIQTYTIVSDPLHMKRAMRIAKHINIEAYASPTPTSAYKTLDTEIPFFFKELFSYIGYVTSLPLKALKG  D | 100 | IgG | 2.5 x 10^-2^ | NA |
|  |  |  | NA | Under  represented in AP IVDU |  |  |

| **C**: BA3828 | hypothetical protein  DNA binding protein HTH_XRE superfamily. belonging to the xenobiotic response element family of transcriptional regulators | MKYSSFGIEVRKVLLERDLTLTVLASELKISVSYLSDILKGSRKGKKQKKAIVELLGLDMCEEDLK  MKYSSFGIEVRKVLLERDLTLTVLASELKISVSYLSDILRGSRKGKKQKKAIVELLGLDMCEEDLK | 98 | IgG | 3.95 x 10^-3^ | NA |
| --- | --- | --- | --- | --- | --- | --- |
|  |  |  | *B. cereus*  WP_000877761.1 | Under  represented in AP and AN IVDU |  |  |
| **D/L**: BA4182  (different between C vs AP IVDU and TCA vs AP IVDU IgG) | pyruvate dehydrogenase (*pdhC*) complex E2 component, dihydrolipoyl  lysine-residue acetyl  transferase | MAFEFKLPDIGEGIHEGEIVKWFIKPGDEVNEDDVLLEVQNDKAVVEIPSPVKGKVLEVLVEEGTVAV  MAFEFKLPDIGEGIHEGEIVKWFIKPGDEVNEDDVLLEVQNDKAVVEIPSPVKGKVLEVLVEEGTVA I  VGDTLIKFDAPGYENLKFKGDDHDEAPKAEATPAATAEVVNERVIAMPSVRKYARENGVDIHKVAGS  VGDTLIKFDAPGYENLKFKGDDHDEAPAAEATPAATAEVVNERVIAMPSVRKYAREKGVDIHKVAGT  GKNGRIVKADIDAFANGGQAVAATEAPAAVEATPAAAKEEAPKAQPIPAGEYPETREKMSGIRKAIA  GKNGRIVKADIDAFANGGQ TVAATEAPAAVEATPAAAKEEAPKAQPIPAGEYPETREKMSGIRKAIA  KAMVNSKHTAPHVTLMDEVDVTELVAHRKKFKAVAADKGIKLTYLPYVVKALTSALREYPMLNTSLD  KAMVNSKHTAPHVTLMDEVDVTELVAHRKKFKAVAADKGIKLTYLPYVVKALTSALREYPMLNTSLD  DASQEVVHKHYFNIGIAADTDKGLLVPVVKDTDRKSIFTISNEINDLAGKAREGRLAPAEMKGASCTIT  DASQEVVHKHYFNIGIAADTDKGLLVPVVKDTDRKSIFTISNEINDLAGKAREGRLAPTEMKGASCTIT  NIGSAGGQWFTPVINHPEVAILGIGRIAEKPVVKNGEIVAAPVLALSLSFDHRLIDGATAQKALNQIKRL  NIGSAGGQWFTPVINHPEVAILGIGRIAEKPVVKNGEIVAAPVLALSLSFDHRLIDGATAQKALNQIKRL  LNDPQLLVMEA  LNDPQLLVMEA | 99 | IgG  IgA | 5.47 x 10^-2^ | 5.47 x 10^-2^ |
|  |  |  | *B. cereus*  WP_016080446.1 | AP IVDU |  |  |
| **E**: BA4815 | peptidase, M42 family | MTKLDETLTMLKELTDARGIAGNEREPREVMKKYIEPFADELSTDNLGSLVAKKVGEENGPKIMVAG  MTKLDETLTMLKELTDARGIAGNEREPREVMKKYIEPFADELSTDNLGSLVAKKVGEENGPKIMVAG  HLDEVGFMITQIDDKGFLRFQTVGGWWSQVMLAQRVTIVTRKGDVTGVIGSKPPHILPPEARKKPVE  HLDEVGFMITQIDDKGFLRFQTVGGWWSQVMLAQRVTIVTRKGDVTGVIGSKPPHILPPEARKKPVE  IKDMFIDIGASSQEEAMEWG I RPGDQVVPYFEFQVMKNEKMLLAKAWDNRIGCAIAIDVLKQLKNEK  IKDMFIDIGASSQEEAMEWGVRPGDQVVPYFEFQVMKNEKMLLAKAWDNRIGCAIAIDVLKQLKNEK  HPNVVYGVGTVQEEVGLRGAKTSANYIKPDIAFAVDVGIAGDTPGVTSKEAQSKMGDGPQIILYDAS  HPNVVYGVGTVQEEVGLRGAKTSANYIKPDIAFAVDVGIAGDTPGVTSKEAQSKMGDGPQIILYDAS  VIGHTGLRDFVVDVADELQIPYQYDSVAGGGTDAGAIHIAVNGIPSMAITIATRYIHSHAAMLHRDDYE  VIGHTGLRDFVVDVADELQIPYQYDSVAGGGTDAGAIHIAVNGIPSMAITIATRYIHSHAAMLHRDDYE  NAVKLIVEVIKRLDKEAVHNITFN  NAVKLIVEVIKRLDKEAVHNITFN | 99 | IgA | NA |  |
|  |  |  | *B. cereus*  WP_000163569.1 | AP  IVDU |  |  |
| **F/N**: BA4334 | 6,7-dimethyl-8-ribityllumazine synthase, riboflavin biosynthesis (*RibH*) | MVFEGHLVGTGLKVGVVVGRFNEFITSKLLGGALDGLKRHGVEENDIDVAWVPGAFEIPLIAKKMAN  MVFEGHLVGTGLKVGVVVGRFNEFITSKLLGGALDGLKRHGVEENDIDVAWVPGAFEIPLIAKKMAN  SGKYDAVITLGTVIRGATTHYDYVCNEVAKGVASLSLQTDIPVIFGVLTTETIEQAIERAGTKAGNKGY  SGKYDAVITLGTVIRGATTHYDYVCNEVAKGVASLSLQTDIPVIFGVLTTETIEQAIERAGTKAGNKGY  ESAVAAIEMAHLSKHWA  ESAVAAIEMAHLSKQWA | 99 | IgG  IgA | 1.04 x 10^-2^ | 1.63 x 10^-2^ |
|  |  |  | *B. cereus*  EJQ58563.1 | AP  IVDU |  |  |
| **G**: BA0472 | hypothetical protein; prophage pi2 protein 37  GTG start | VASIDSLANDIARELQRYGKEVEEKLEVEKEEVANNLVSDLKENSPKNTGKYAKGWRKKKEGNGFIV  VASIDSLANDIARELQRYSNVVEEDLEVAKEEVATDLVNELKQKSPKKTGKYGKGWRKKKDGSA I IV  HNALKPQLTHLLEKGHAQVNGGRVPAKVHIAPAEEKAENEFLERVERAIQQ  YNALKPQLTHLLEKGHAK ANGGRVAAKVHIAPAEEKA INEL I ERVERAIQQ | 79 | IgG | 3.95 x 10^-3^ | NA |
|  |  |  | *B. weihenstephanensis*  WP_012260128.1 | AP and AN IVDU |  |  |
| **H**: BA0391 | DNA Binding protein, PbsX family transcriptional regulator | MKNQIYELRTENNISQGALADKCKVSRQTINAIENNKYDPSLALAFRLAEVLGTTVDKLFLYKQ | 100 | IgG  IgA | 3.95 x 10^-3^ | 2.5 x 10^-2^ |
|  |  |  | NA | AP IVDU |  |  |
| **I**: BA3824 | conserved hypothetical protein , bacillus phage lambda | MNLVGIENLVLPEDAELAKSLRNKKENYIKNQFLLTRIASKKNVEGKTKEFYEACKEYEACGEKAKEC  MNLVGIENLVLPEDAELAKSLRNKKENYIKNQFLLTRIASKKNVEGKTKEFYETCKEYEACGEKAKEC  DKQLKELIFKKKENDRVQHVVERMREVGIKEDVIQKVLYK  DKQLKELIFKKKENDRVQHVVERMRE IGIKDDVIQKVLYK | 97 | IgG | 3.95 x 10^-3^ | NA |
|  |  |  | *B. cereus*  CP000001.1 | AP and AN IVDU |  |  |
| **J**: BA3953 | N utilization substance protein A (*NusA*) | MSTELLDALLVLESEKGISKDIIIDAIEAALISAYKRNFNQAQNVRVSFNPQVGTIQVLARKDVVDNVFD  MSTELLDALLVLESEKGISKDIIIDAIEAALISAYKRNFNQAQNVRVSFNPQVGTIQVLARKDVVDNVFD  PRLEISVEEARQINPNYQDGDVLEIEVTPKDFGRIAAQTAKQVVTQRVREAERGVIYSEFSDREEDIM  PRLEISVEEARQINPNYQDGDVLEIEVTPKDFGRIAAQTAKQVVTQRVREAERGVIYSEFSDREEDIM  VGIVQRQDARFIYVSLGKVEALLPVSEQMPNEQYKPHDRIRVFITKVEKTTKGPQIYVSRTHPGLLKR  VGIVQRQDARFIYVSLGKVEALLPVSEQMPNEQYKPHDRIRVFITKVEKTTKGPQIYVSRTHPGLLKR  LFEMEVPEIYDGTVEIRSVAREAGDRSKISVHAENIDVDPVGSCVGPKGQRVQRIVDELKGEKIDIVR  LFEMEVPEIYDGTVEIRSVAREAGDRSKISVHAENIDVDPVGSCVGPKGQRVQRIVDELKGEKIDIVR  WSNDPVEYVANALSPSQVVKVLVDEEEKATTVVVPDHQLSLAIGKRGQNARLAAKLTGWKIDIKSES  WSNDPVEYVANALSPSQVVKVLVDEEEKATTVVVPDHQLSLAIGKRGQNARLAAKLTGWKIDIKSES  DAKQLGIVTEEDSVVAFGFDSVEDEIE  DAKQLGIVTEEDSVVAFGFDS IEDEIE | 99 | IgG  IgA | 3.95 x 10^-3^ | 6.49 x 10^-3^ |
|  |  |  | *B. cereus*  WP_000102608.1 | AP  IVDU |  |  |
| **K**: BA4424 | conserved hypothetical protein | MSQEQLGTRNFVQIGLFGGLFWGGIWYFLHIFSFTEAGPNYLLLPFAFGSWKEGVWGNVLGIVCMA  MSQEQLGTRNFVQIGLFGGI FWGGIWYFLHIFSFTEAGPNYLLLPFAFGSWKEGVWGNVLGIVCMA  LLSILIAFLYKAFLAKFEGILPGMIYGLFWWALLFFGVGLIAPAIKSALHLPKETIVTTICIFILYGVFIAYSV  LLSILIAFLYKAFLAKFEGILPGMIYGLFWWALLFFGVGLIAPAIKSALHLPKETIVTTICIFILYGVFIAYSV  SYAVNTNKAEREGEEKTNYSNK  SYAVNTNKAEREGEEKTNYSNK | 99 | IgA | NA | 1.63 x 10^-2^ |
|  |  |  | *B. cereus*  WP_000076889.1 | AP  IVDU |  |  |
| **L:** BA1949 | microcin immunity protein MccF | MPLPKSLKYGDTIGIYSPSSPVTYTSPKRFERAKSYLLQKGFHILEGSLTGRYDYYRSGSIQERAKEL  NALIRNPNVSCIMSTIGGMNSNSLLPYIDYDAFQNNPKIMIGYSDATALLLGIYAKTGIPTFYGPALVPS  FGEFEPFVDDTYKYFLETLLHDQALPYNIKQPLFWSDEFINWEEKTKEKELRPNNWISVTNGQATGR  V IGGNLNTIQGIWGSPYMPCIQEGDILFIEDSSKDAATIERSFSFLKINGVFDKVSGIILGKHEQFDDC  GTNRKPYEILLEVLQNQRIPLLADFDCCHTHPMITMPIGVQVKMDATNKTIHILEKWKI | 100 | IgG  IgA | 1.04 x 10^-2^ | 2.5 x 10^-2^ |
|  |  |  | NA | AP and AN IVDU |  |  |
| **M**: BA3589 | hypothetical protein | MYPYNPYNPYNQNGYQPQYDLQAQYQQYIDTAEQVNPYDQNRQFQLPISFPGAGNRQLERRVNEL  EQRVRQLENTVERHTRRLNRLNQRLRTIENRLNIPFSALEDGF | 100 | IgG  IgA | 6.49 x 10^-3^ | 2.5 x 10^-2^ |
|  |  |  | NA | HP |  |  |
| **O:** BA4324 | putative hydrolase, alpha/beta fold family | MLFRSYTPQFYNENKQPIPNSIATMESVMINNRKQTLLIRGQNVEQPILLCCHGGPGMAQIGFIRHFQ  KELEKHFIVINWDQRGAGKSFSTKDFGANFTIEQFISDAKEVIQYVLKKFSKQKLFLAGHSWGSIIGLNI  AHQYPQYIEAYIGIGQIVHMKQNEELLYQHLIRSAKKHDHKKALASLLKLGKPPFLDTRRLIIQRKWLG  TFGGAIQNGSSFSFIRKGFFSPEYTLLDWFKFLAGNLKSGVLWEEMLTIDFFSSITSLSIPVYFCSGRY  DYQTPYALVQEYCDVIEAPIKKMI WFPNSAHSPDLEEPELFAHSLQSIKQELAFQH | 100 | IgG  IgA | 6.49 x 10^-3^ | 2.5 x 10^-2^ |
|  |  |  | NA |  |  |  |
