# Supplementary material for: Whole genome protein microarrays for serum profiling of immunodominant antigens of Bacillus anthracis
Source: Front Microbiol. 2015 Aug 13;6:747. doi: 10.3389/fmicb.2015.00747 (PMC4534840; doi:10.3389/fmicb.2015.00747)
Supplement: Supplementary file 7 [file DataSheet7.DOCX]

**Supplementary Information S7**

**Anti-toxin component Ig responses in Vaccinated Rabbits; Table 1 – IgG, Table 2 – IgA**

**Table 1**

| Toxin Component | Antigen  Concentration  (ng/ul) | Fold Change  Spore vs Control | Regulation  Spore vs Control | Fold Change  UK AVP vs Control | Regulation  UK AVP vs Control | IgG Fold Change Difference  Spore vs AVP |
| --- | --- | --- | --- | --- | --- | --- |
| PA | 200 | 18.492619 | up | 87.696495 | up | 0.210870674 |
| PA | 100 | 57.959 | up | 433.27698 | up | 0.133768935 |
| PA | 50 | 78.387726 | up | 769.03143 | up | 0.101930458 |
| PA | 25 | 92.22782 | up | 951.8399 | up | 0.096894257 |
| PA | 12.5 | 37.774727 | up | 468.5911 | up | 0.080613411 |
| PA | 6.25 | 10.641657 | up | 127.94019 | up | 0.083176811 |
| PA | 3.125 | 33.06747 | up | 99.91951 | up | 0.330941074 |
| PA | 1.5625 | 17.183727 | up | 177.80159 | up | 0.096645519 |
| PA | 0.78125 | 9.30365 | up | 100.26688 | up | 0.092788865 |
| PA | 0.390625 | 5.965974 | up | 48.476883 | up | 0.123068432 |
| LF | 200 | 5.4048557 | up | 26.494177 | up | 0.204001645 |
| LF | 100 | 6.315834 | up | 30.994871 | up | 0.203770295 |
| LF | 50 | 6.969614 | up | 33.72635 | up | 0.206651891 |
| LF | 25 | 8.162482 | up | 39.96827 | up | 0.204224051 |
| LF | 12.5 | 15.034971 | up | 64.41444 | up | 0.233409947 |
| LF | 6.25 | 23.906427 | up | 119.39508 | up | 0.200229582 |
| LF | 3.125 | 46.741383 | up | 245.88025 | up | 0.19009816 |
| LF | 1.5625 | 48.891838 | up | 320.58118 | up | 0.152510007 |
| LF | 0.78125 | 47.058334 | up | 400.42722 | up | 0.117520317 |
| LF | 0.390625 | 63.341194 | up | 526.06134 | up | 0.12040648 |
| EF | 200 | 11.676983 | up | 61.23174 | up | 0.190701473 |
| EF | 100 | 20.329407 | up | 131.46497 | up | 0.154637444 |
| EF | 50 | 25.8304 | up | 134.37494 | up | 0.192226318 |
| EF | 25 | 27.821533 | up | 134.1421 | up | 0.20740344 |
| EF | 12.5 | 35.209835 | up | 172.87166 | up | 0.203676155 |
| EF | 6.25 | 28.068531 | up | 126.66317 | up | 0.221599783 |
| EF | 3.125 | 139.03471 | up | 549.80914 | up | 0.252878135 |
| EF | 1.5625 | 68.29495 | up | 317.07483 | up | 0.215390638 |
| EF | 0.78125 | 310.33536 | up | 1558.9526 | up | 0.199066578 |
| EF | 0.390625 | 257.29276 | up | 1630.473 | up | 0.157802527 |

| Toxin Component | Antigen  Concentration  (ng/ul) | Fold Change  Spore vs Control | Regulation  Spore vs Control | Fold Change  UK AVP vs Control | Regulation  UK AVP vs Control | IgA Fold Change Difference  Spore vs AVP |
| --- | --- | --- | --- | --- | --- | --- |
| PA | 200 | 394.59515 | up | 13.868243 | up | 28.45314652 |
| PA | 100 | 185.69582 | up | 5.4165506 | up | 34.28303984 |
| PA | 50 | 191.06346 | up | 5.6656775 | up | 33.72296782 |
| PA | 25 | 90.427826 | up | 2.46029 | up | 36.75494596 |
| PA | 12.5 | 71.21438 | up | 3.0648801 | up | 23.23561695 |
| PA | 6.25 | 31.663311 | up | 2.696407 | up | 11.74277882 |
| PA | 3.125 | 88.97781 | up | 1.3356614 | up | 66.61704082 |
| PA | 1.5625 | 25.503767 | up | 1.3342887 | up | 19.1141295 |
| PA | 0.78125 | 33.34618 | up | -1.0627785 | down | -31.37641569 |
| PA | 0.390625 | ND | ND | ND | ND | ND |
| LF | 200 | 258.47583 | up | 10.813033 | up | 23.90410073 |
| LF | 100 | 329.3774 | up | 13.401043 | up | 24.5784899 |
| LF | 50 | 320.34326 | up | 11.638595 | up | 27.52422092 |
| LF | 25 | 308.92084 | up | 11.356737 | up | 27.20154918 |
| LF | 12.5 | 220.83649 | up | 6.5231433 | up | 33.8543061 |
| LF | 6.25 | 340.22742 | up | 6.7111425 | up | 50.69590163 |
| LF | 3.125 | 325.15936 | up | 5.000997 | up | 65.01890723 |
| LF | 1.5625 | 234.41925 | up | 3.2981284 | up | 71.07644748 |
| LF | 0.78125 | 190.85802 | up | 3.6939745 | up | 51.66738969 |
| LF | 0.390625 | 60.124443 | up | 2.0248075 | up | 29.69390572 |
| EF | 200 | 677.75146 | up | 27.300842 | up | 24.82529513 |
| EF | 100 | 375.2475 | up | 15.691208 | up | 23.91450677 |
| EF | 50 | 461.78882 | up | 16.717848 | up | 27.62250381 |
| EF | 25 | 306.61133 | up | 10.647168 | up | 28.79745393 |
| EF | 12.5 | 425.0698 | up | 13.936853 | up | 30.49969746 |
| EF | 6.25 | 63.14003 | up | 1.7145938 | up | 36.82506609 |
| EF | 3.125 | 324.27628 | up | 6.615519 | up | 49.0175117 |
| EF | 1.5625 | 267.0303 | up | 5.0371995 | up | 53.01165856 |
| EF | 0.78125 | 188.9476 | up | 2.8399398 | up | 66.53225537 |
| EF | 0.390625 | 115.11861 | up | 1.6006153 | up | 71.92147295 |

**Table 2**
